# Chiral Bis-8-Aryl-isoquinoline Bis-alkylamine Iron Catalysts for Asymmetric Oxidation Reactions

**DOI:** 10.1021/acs.orglett.5c00050

**Published:** 2025-01-22

**Authors:** Tomer Mintz, Lei Liu, Doron Pappo

**Affiliations:** † Department of Chemistry, Ben-Gurion University of the Negev, Beer-Sheva 8410501, Israel; ‡ School of Chemistry and Chemical Engineering, Shandong University, Jinan 250100, China

## Abstract

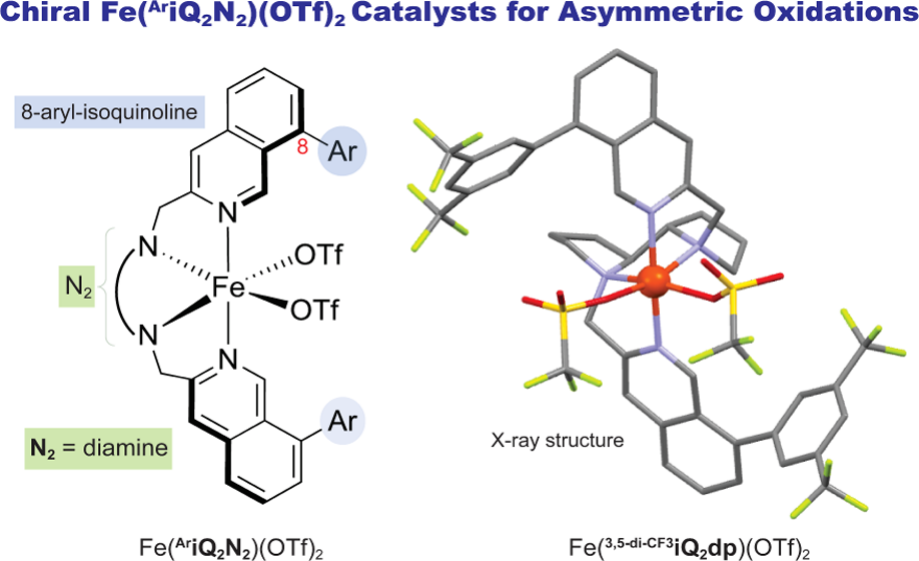

A novel class of bis-8-aryl-isoquinoline (^**Ar**^**iQ**) bis-alkylamine iron complexes, Fe^II^(^**Ar**^**iQ_2_dp**)(OTf)_2_ and Fe^II^(^**Ar**^**iQ_2_mc**)(OTf)_2_ (**dp** = dipyrrolidinyl or **mc** = *N*,*N*′-dimethylcyclohexyl-diamine), for asymmetric oxidation reactions is reported. The scalable divergent synthesis of 8-aryl-3-formylisoquinolines (**8**), the key intermediates in preparing these ligands, enables precise structural and electronic tuning around the metal center. The enantioselective epoxidation and hydroxy carbonylation of conjugated alkenes, mediated by the Fe^II^(^**3,5-di-CF_3_**^**iQ_2_dp**) catalyst with H_2_O_2_ as the oxidant, demonstrates the potential of these redox Fe^II^[N_4_] catalysts in inducing face selection in oxygen transfer transformations.

Chiral iron and manganese complexes with well-defined first coordination sphere tetradentate ligands (N_4_) are privileged catalysts for various oxidation reactions.^1^ In recent years, these highly efficient catalysts were applied in water oxidation,^2^ oxidative coupling,^3^ stereoselective hydroxylation of strong and remote C–H bonds,^4^ and the enantioselective epoxidation and *cis*-dihydroxylation of both electron-deficient and electron-rich olefins.^5^ To further enhance oxidation reactions for preparing optically pure compounds from simple achiral substrates, there is an emerging need to develop innovative N_4_-type ligands that impart distinctive structural and electronic properties to the metal.

Que,^6^ Ménage,^7^ White,^8^ Costas,^5a^ and others demonstrated that Fe and Mn complexes with aminopyridine ligands (Py_2_N_2_, e.g., **1a**–**1c**; Figure 1A-i) serve as highly effective catalysts in oxygen transfer reactions.^1d,6b^ The Py_2_N_2_ ligands are arranged around the metal in a *cis*-α topological configuration, with the two pyridine units positioned *trans* to each other, forming complexes that are resilient to oxidative degradation and demetalation.^9^ The M[Py_2_N_2_] complexes (M = Fe or Mn) facilitate the heterolytic cleavage of H_2_O_2_, resulting in the formation of reactive M[N_2_Py_2_][S]=O species (S = solvent, acid additive, or substrate; Figure 1A-ii) that transfer oxygen atom(s) or accept a hydrogen atom from substrates that are positioned within the active site, near the pyridine rings.^1d,10^ Therefore, the primary approach to regulating the enantioselectivity in these oxidation reactions involves structural modification of the pyridine moieties. A systematic study by the Costas group demonstrated that introducing substituents at the γ-pyridine position significantly alters the electronic configuration of the catalysts, thereby impacting their reactivity and selectivity.^11^ On the other hand, the α-pyridine site that should be most suitable to project the chirality to the incoming substrate is limited to H and Me substituents, as larger substituents tend to destabilize the complex.^12^ As a result, the chiral environment surrounding the metal center is typically regulated from the β-pyridine site.^8a,13^ From the perspective of catalyst design, this presents a constraint given that the orientation of the substituents (e.g., aryl groups) at the β position is directed away from the metal center, thereby rendering the catalytic site relatively exposed (Figure 1B).

**Figure 1:**
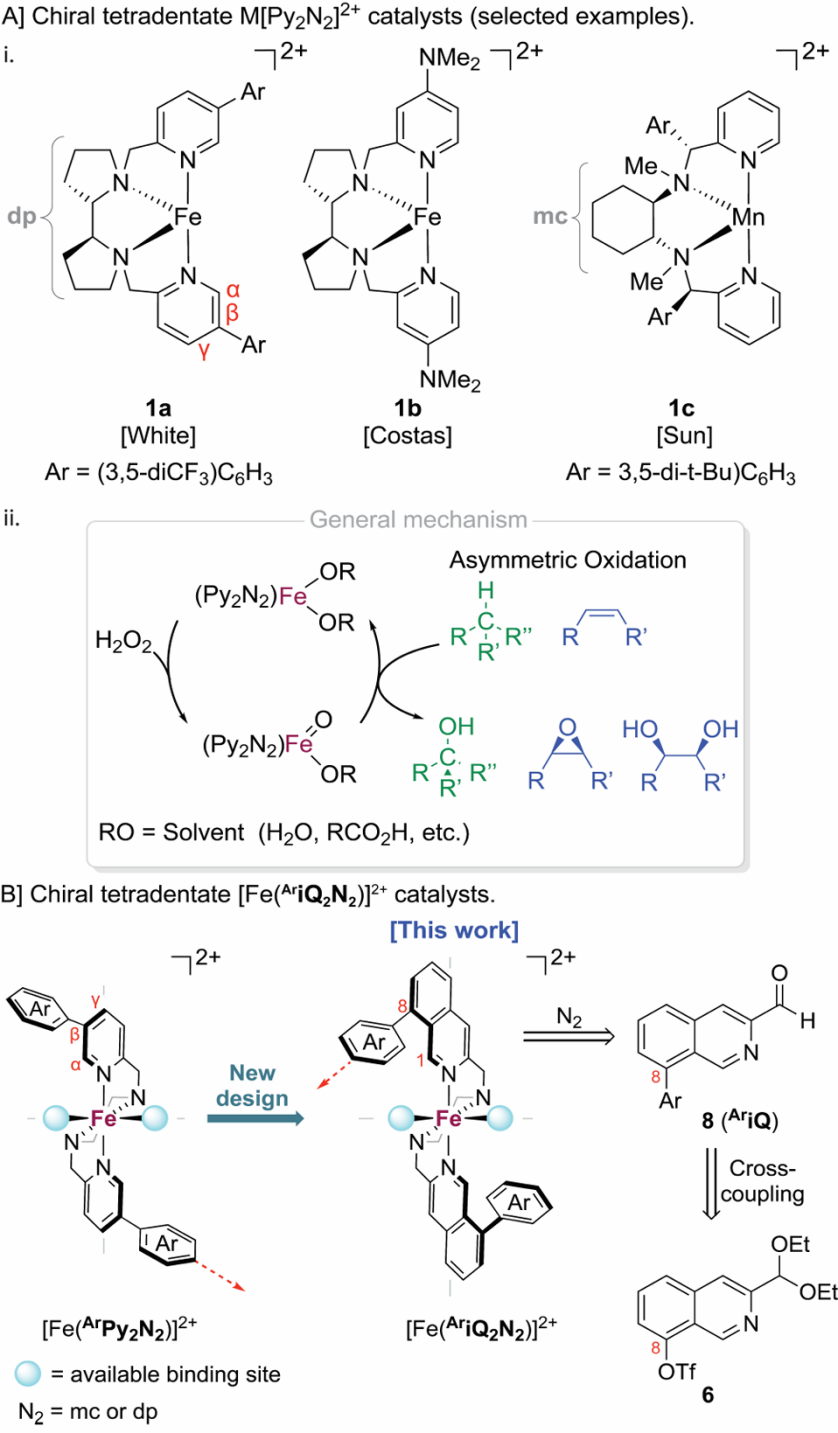
Selected examples of chiral tetradentate M[Py_2_N_2_]^2+^ catalysts and general mechanism of the asymmetric oxidation reaction catalyzed by them.

An additional developing approach to enhance the selectivity of redox M[N_4_] catalysts involves replacing the pyridine unit with alternative heterocycles. Utilizing this methodology, tetradentate iron and manganese complexes incorporating *N*-methylbenzimidazole,^14^ quinoline,^15^ and isoquinoline (iQ)^16^ moieties have been synthesized and effectively employed in a variety of oxidation reactions. The bis-isoquinoline bis-alkylamine iron complex [Fe(iQ_2_N_2_)] (Figure 1B; Ar = H) displayed similar reactivity and selectivity to the [Fe(Py_2_N_2_)] complex in olefin epoxidation and C–H hydroxylation reactions. These promising results inspired us to examine the idea of introducing a bulky group at the remote C-8 position of the isoquinoline units instead of the β-pyridine sites in the Py_2_N_2_ ligands. We hypothesized that the 8-isoquinoline position could be strategically employed to modulate the electronic characteristics of the metal while simultaneously generating a confined metal active site deeply buried within a highly demanding chiral environment (Figure 1B). To make this hypothesis possible, the tetradentate ^**Ar**^**iQ**_**2**_**N**_**2**_ [^**Ar**^**iQ** = 8-arylisoquinoline and **N**_**2**_ = **dp** (dipyrrolidinyl) or **mc** (*N*,*N*′-dimethylcyclohexyl-diamine] ligands along with their corresponding metal complexes had to be prepared (Figure 1B).

**Scheme 1:**
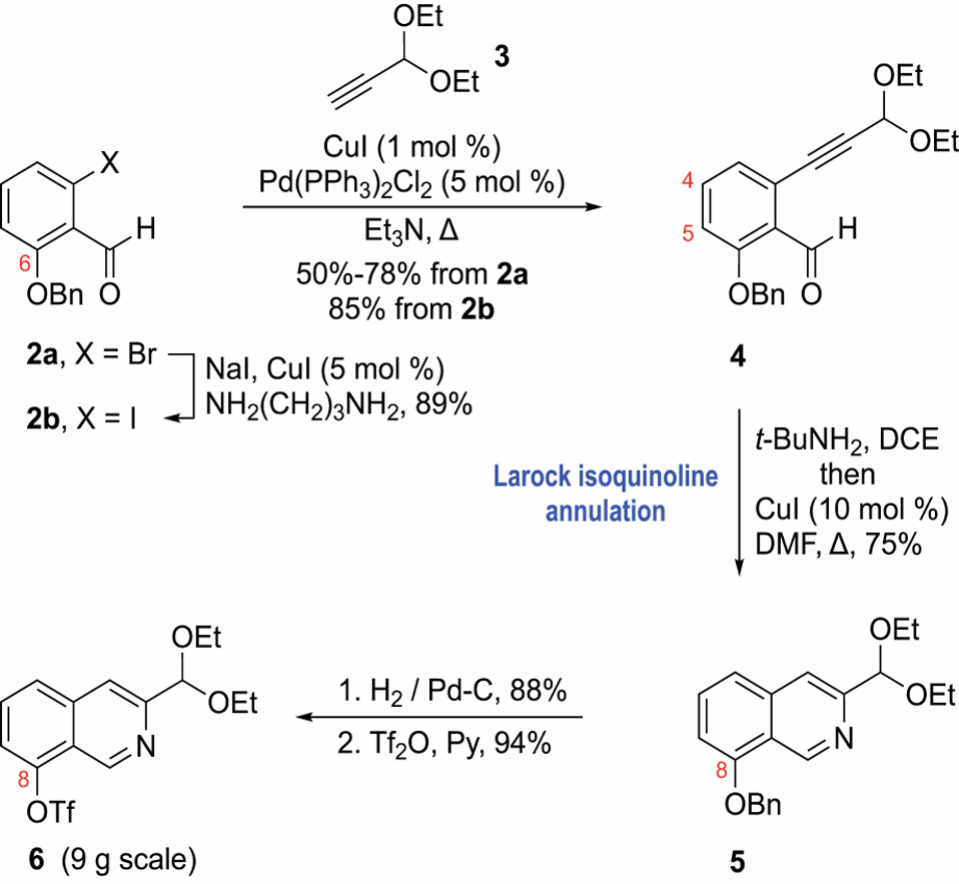
Synthesis of 8-Triflate-3-diethoxymethyl-isoquinoline **6** See the Supporting Information for the exact conditions.

Synthetically, 8-OTf-3-(diethoxymethyl)-isoquinoline product **6**, which can be diversified by Suzuki–Miyaura coupling to different 8-aryl-3-formyl-isoquinoline products **8**, was targeted (Figure 1B). Preparing the ^**Ar**^**iQ**_**2**_**dp** and ^**Ar**^**iQ**_**2**_**mc** ligands from compound **8** and chiral diamines allows further library expansion for structure–activity–selectivity relationship studies. However, a database search revealed no established synthetic pathway existed to prepare 8-substituted-3-formyl-isoquinolines **8**. As a result, a new synthesis had to be developed.

Here within, this section, we describe the synthesis and catalytic activity of a new class of tetradentate Fe(^**Ar**^**iQ**_**2**_**dp**)(OTf)_2_ and Fe(^**Ar**^**iQ**_**2**_**mc**)(OTf)_2_ complexes with tunable electronic and steric properties. The potential of these chiral Earth-abundant metal complexes to promote enantioselective oxygen-transfer reactions is demonstrated for the enantioselective epoxidation and hydroxy carbonylation of conjugated alkenes.

The study began by developing a reliable and scalable synthesis of 8-OTf-3-formyl-isoquinoline **6** starting from 2-bromo-6-benzyloxybenzaldehyde **2** (Scheme 1). We employed the Larock isoquinoline synthesis, which enables the conversion of 2-ethynylbenzaldehydes into isoquinolines in the presence of an amine source, as the key step in our design.^16a,17^ This copper-catalyzed transformation has mainly been utilized for 2-ethynylbenzaldehydes with remote substitutions at the C-4 and C-5 positions. Applying the method for substrates with neighboring C-6 substituents,^18^ such as compound **4** (Scheme 1), is relatively uncommon.

Therefore, to synthesize compound **4**, Sonogashira coupling between 2-benzyloxy-6-bromobenzaldehyde (**2a**), which was prepared by benzylation of 2-hydroxybenzaldehyde, and 3,3-diethoxyprop-1-yne (**3**) [1.3 equiv, Pd(PPh_3_)Cl_2_ (5 mol %), triethanolamine (TEA), 70 °C, 4 h; Scheme 1] was carried out. This reaction afforded 6-benzyloxy-2-ethynylbenzaldehyde **4** in moderate yields ranging between 50 and 78%. To address the inconsistency of the process, a bromide-to-iodine atom-exchange step by aromatic Finkelstein substitution (NaI, CuI, 1,3-diaminopropane, dioxane, 89% yield)^19^ was introduced before the cross-coupling. This tactic turned out to be successful, and the Sonogashira coupling of 2-(benzyloxy)-6-iodobenzaldehyde (**2b**) with alkyne **3** proceeded under milder reaction conditions [Pd(PPh_3_)Cl_2_ (2 mol %), TEA, 50 °C, 1 h], yielding product **4** with an improved 85% yield. The annulation of 2-ethynylbenzaldehyde **4** was best performed when we first ensured the formation of the *N*-*tert*-butyl-phenylmethanimine intermediate [*t*-BuNH_2_, 1,2-dichloroethane (DCE)] before proceeding to the annulation step [CuI, *N*,*N*-dimethylformamide (DMF)]. Under these conditions, the desired product 8-benzyloxy-3-(diethoxymethyl)-isoquinoline **5** was isolated in 75% yield. Selective hydrogenolysis of the benzyl-protecting group (H_2_, Pd/C, 88% yield) and triflation of the 8-OH group (Tf_2_O, pyridine, 94% yield) afforded the key 8-triflate-3-diethoxymethyl-isoquinoline **6** in excellent yields. This robust short synthesis was performed on a practical scale, delivering up to 9 g of compound **6** in a single campaign.

Isoquinoline **6**, which is a stable solid that can be safely stored for a long period in a refrigerator, is an excellent partner in Suzuki–Miyaura cross-coupling reactions. Its reaction with different aryl boronic acids [1.2 equiv, Pd(PPh_3_)_4_ (5 mol %), ethanol, 90 °C, 4 h] affords, after acetal hydrolysis workup (aqueous HCl, dioxane), the target 8-aryl-3-formylisoquinolines **8a**–**8d** and **8f**–**8h** in good to excellent yields (Scheme 2A). Alternatively, isoquinoline **6** can be converted to 8-Bpin-3-(diethoxymethyl)-isoquinoline **7** through the Miyaura borylation method [bis(pinacolato)diboron (B_2_pin_2_, 3 equiv), Pd(dppf)_2_Cl_2_ (3 mol %), KOAc (3 equiv), 1,4-dioxane, 90 °C, 3 h, 84%]^20^ before subjected to coupling with aryl halides. For example, the coupling of compound **7** with 5-bromo-1,3-(bis-trimethylsilane)phenylene and 9-bromoanthracene afforded after acetal hydrolysis isoquinolines **8e** and **8i** in 58 and 74% yields, respectively. The 8-aryl-3-formyl-isoquinoline products **8a**–**8i** highlight the versatility of this synthesis, allowing for the introduction of aryl groups with customizable electronic and steric characteristics.

**Scheme 2:**
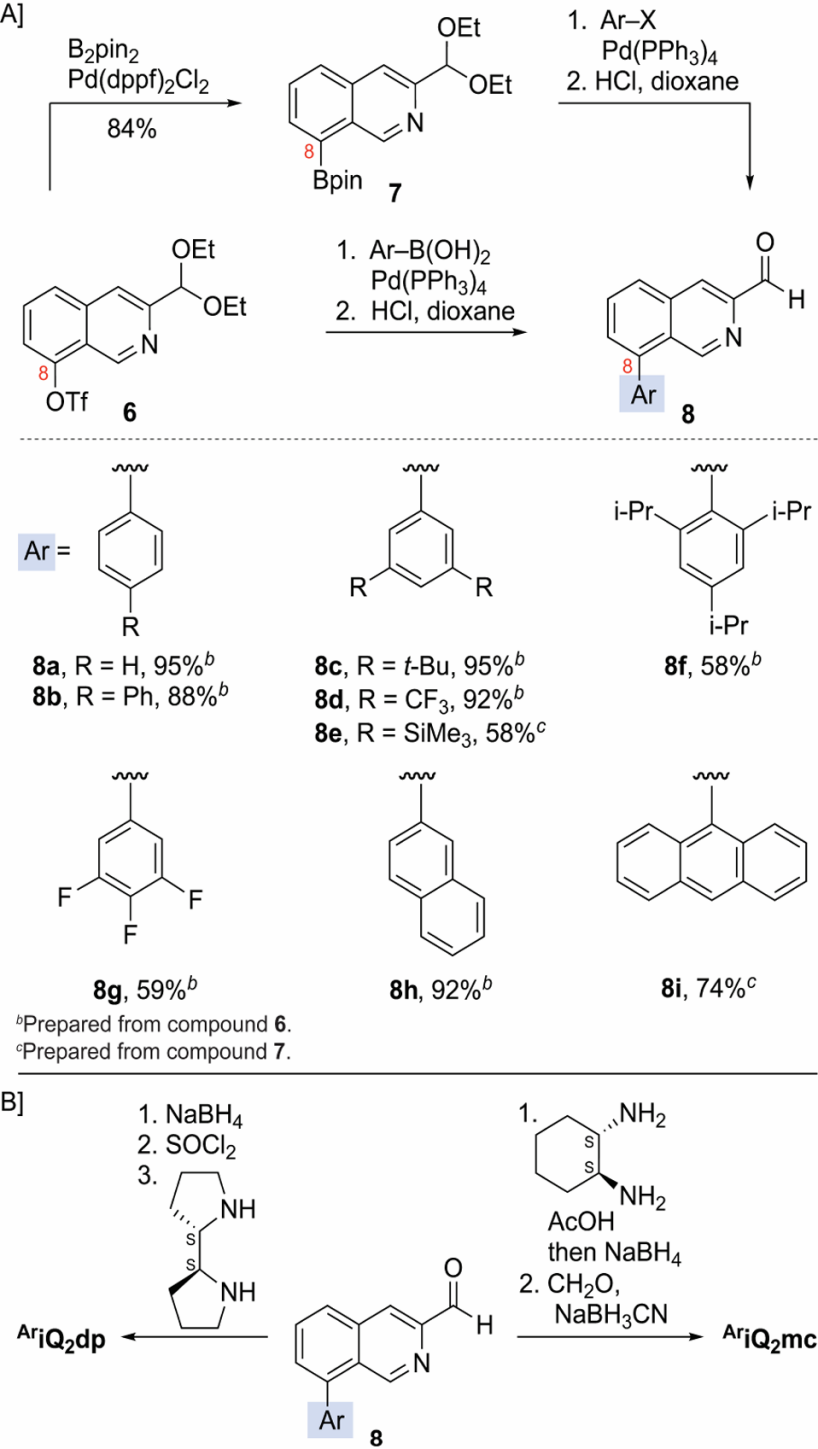
Synthesis of 8-Aryl-3-formylisoquinolines **8a**–**8i** and the ^**Ar**^**iQ**_**2**_**mc** and ^**Ar**^**iQ**_**2**_**dp** Ligands See the Supporting Information for the exact conditions.

Next, the tetradentate ^**Ar**^**iQ**_**2**_**mc** and ^**Ar**^**iQ**_**2**_**dp** ligands were prepared from 8-aryl-3-formylisoquinolines **8** and either (1*S*,2*S*)-cyclohexane-1,2-diamine or (2*S*,2′*S*)-2,2′-bipyrrolidine by employing established methodologies (Scheme 2B). Mixing these strong chelators with freshly prepared Fe(OTf)_2_(CH_3_CN)_2_ in tetrahydrofuran (THF) led to the formation of orange precipitates of Fe^II^(^**Ar**^**iQ**_**2**_**mc**)(OTf)_2_ or Fe^II^(^**Ar**^**iQ**_**2**_**dp**)(OTf)_2_ complexes (Figure 2). The structure of the novel complexes was determined by ^1^H nuclear magnetic resonance (NMR) spectroscopy and high-resolution mass spectrometry (HRMS) analysis and confirmed by securing the crystal structures of Fe(^**3,5-di-CF_3_**^**iQ**_**2**_**dp**) and Fe(^**2,4,6-tri-iPr**^**iQ**_**2**_**dp**) complexes (Figure 2).

**Figure 2:**
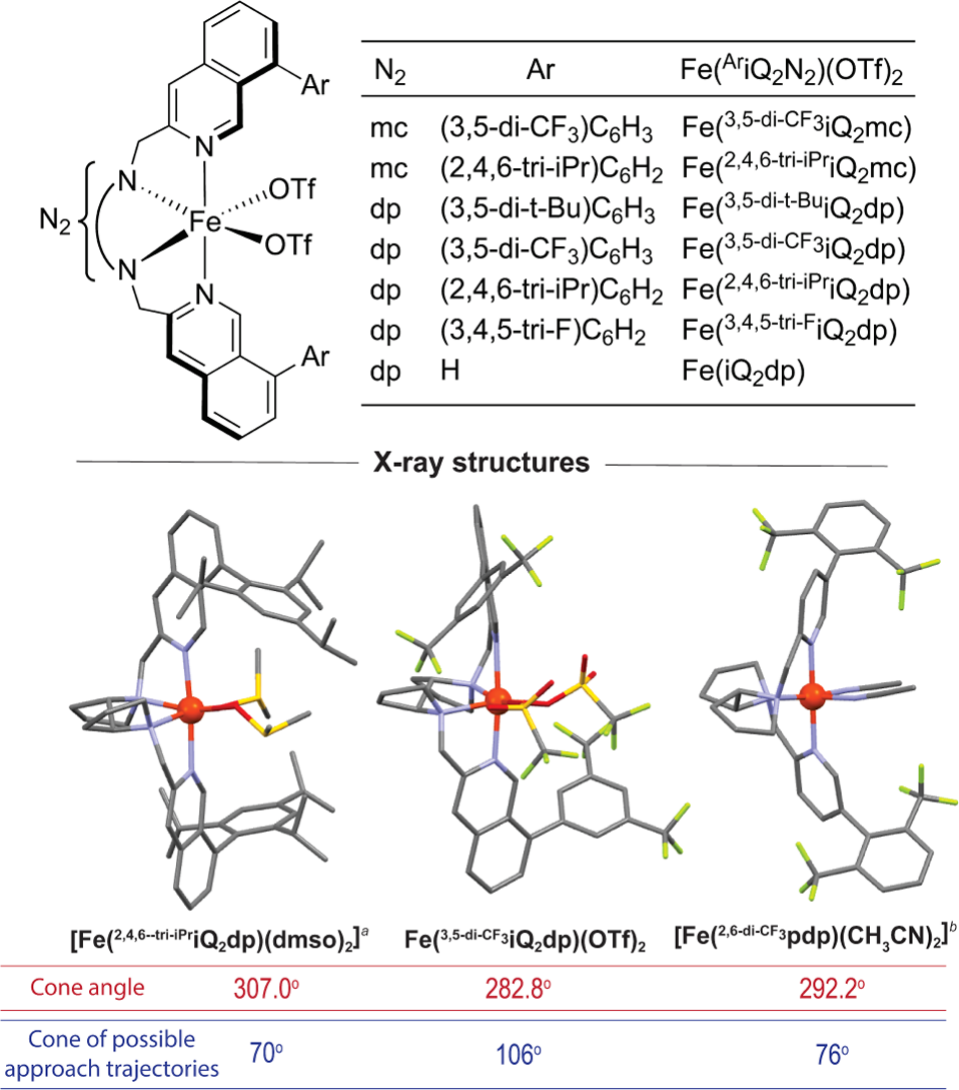
Fe(^Ar^iQ_2_mc)(OTf)_2_ and Fe(^Ar^iQ_2_dp)(OTf)_2_ complexes and the X-ray structure of **Fe(**^**2,4,6-tri-iPr**^**iQ**_**2**_**dp)** (CCDC number 2361551), **Fe(**^**3,5-di-CF_3_**^**iQ**_**2**_**dp)** (CCDC number 2390794), and Fe(^2,6-di-CF_3_^pdp) (**1a**).^4j^^*a*^The hydrogen atoms and outer sphere triflate anions were omitted for clarity. ^*b*^The hydrogen atoms and outer sphere SbF_6_ anions were removed for clarity.

To evaluate the reactivity and selectivity of the new complexes, we examined their catalytic activity for the enantioselective epoxidation of alkyl cinnamates (**10a**–**10i**; Table 1 and Scheme 3A,B). This transformation poses significant challenges for Fe(N_4_) complexes. For example, catalysts **1b** and **1c** (Figure 1A) facilitated the epoxidation of electron-deficient olefin **10a** to produce methyl (2*R*,3*S*)-3-phenyloxirane-2-carboxylate **11a** with a high degree of enantioselectivity yet with moderate yields (entries 1 and 2 in Table 1).^21^ First, we implemented the epoxidation conditions reported by Costas for catalyst **1b**.^5d,10b,11,16b,21^ Under these conditions, the **Fe(**^**3,5-di-CF_3_**^**iQ**_**2**_**dp)** complex [2 mol %, 2-ethyl-hexanoic acid (EHA, 5 mol %) H_2_O_2_ (1.5 equiv), acetonitrile, −40 °C] mediated the formation of the desired epoxide **11a** in a 20% conversion and with 73% enantiomeric excess (ee) (see Table S1 of the Supporting Information). After a short optimization phase, we identified that the use of benzoic acid (50 mol %) as the additive in a mixture of acetonitrile and 2,2,2-trifluoroethanol (TFE) at −40 °C leads to a significant improvement in the results, affording epoxide **11a** in 99% yield and 95% ee (entry 3 in Table 1).

**Table 1:**
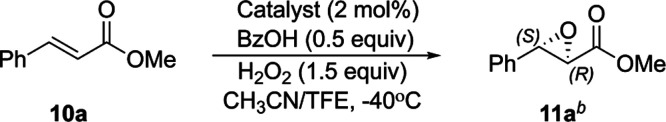
Asymmetric Epoxidation of Methyl Cinnamate **10a**^a^

entry	catalyst	yield (%)^c^	ee (%)^d^
1^e^	**1b**	66	91
2^f^	**1c**	48	95
3	**Fe(**^**3,5-diCF_3_**^**iQ**_**2**_**dp)**	99 (98)^g^	95
4	**Fe(iQ**_**2**_**dp)**	37	57
5	**Fe(**^**3,5-diCF_3_**^**iQ**_**2**_**mc)**	32	55
6	**Fe(**^**3,5-di-***t***-Bu**^**iQ**_**2**_**dp)**	85	69
7	**Fe(**^**3,4,5-tri-F**^**iQ**_**2**_**dp)**	50	60
8	**Fe(**^**2,4,6-tri-iPr**^**iQ**_**2**_**dp)**	0	
9	**Fe(**^**2,4,6-tri-iPr**^**iQ**_**2**_**mc)**	0	

a Reaction conditions: H_2_O_2_ (1.5 equiv) in acetonitrile (0.5 M) is added over 30 min to a TFE (0.25 M) solution of alkyl cinnamate (1 equiv), catalyst (2 mol %), and benzoic acid (0.5 equiv) at −40 °C.

b The absolute configuration of the product was assigned as (−)-(2*R*,3*S*)-**11**.

c High-performance liquid chromatography (HPLC) yield using chlorobenzene as internal standard.

d Enantiomeric excess, determined by HPLC with a chiral stationary phase.

e From ref (11).

f From ref (21).

g Isolated yield.

The cone angle and cone of possible approach trajectories^22^ of the latter complexes and the White catalyst **1a** (Figure 2) suggest that the C-8 substitution has a substantial impact on the environment surrounding the iron and, therefore, should lead to a notable effect on its catalytic performance. Indeed, the **Fe(iQ**_**2**_**dp)** complex, which lacks the C-8 aryl group, yielded epoxide **11a** in 37% yield and 57% ee (entry 4), while **Fe(**^**3,5-diCF_3_**^**iQ**_**2**_**mc**), **Fe(**^**3,5-di-*t*-Bu**^**iQ**_**2**_**dp**) and **Fe(**^**3,4,5-tri-F**^**iQ**_**2**_**dp**) complexes exhibited diminished effectiveness (entries 5–7). The inferior performance of **Fe(**^**3,5-di-*t*-Bu**^**iQ**_**2**_**dp**) compared to **Fe(**^**3,5-di-CF_3_**^**iQ**_**2**_**dp)** (entries 3 and 6) may also be attributed to differences in the ligand electronic properties. The highly sterically demanding **Fe(**^**2,4,6-tri-iPr**^**iQ**_**2**_**dp)** and **Fe(**^**2,4,6-tri-iPr**^**iQ**_**2**_**mc)** complexes left the substrates untouched (entries 8 and 9).

The epoxidation of electron-deficient olefins **10b**–**10f** using the **Fe(**^**3,5-di-CF_3_**^**iQ**_**2**_**dp)** complex further underscores the sensitivity of the reaction to steric changes (compare compounds **11a**, **11b**, and **11c** as well as conjugated ketones **11d** and **11e** to chalcone **11f**; Scheme 3A). Methyl cinnamates with a 4-methyl or 4-bromide substituent afforded the corresponding epoxides **11g** (95% yield, 93% ee) and **11h** (47% yield, 76% ee; Scheme 3A). On the other hand, methyl 4-methoxycinnamate underwent hydroxy carboxylation with the acid additive, affording monoprotected diols **12ia** (benzoic acid, 46%, 37.5% ee), **12ib** (pivalic acid, 62%, 82% ee), and **12ic** [(*S*)-ibuprofen, 46%, 81% diastereomeric excess (de); Scheme 3B].^23^ Previous studies have proposed the involvement of a Fe(V)[N_4_][OC(O)R]=O intermediate, which facilitates the transfer of hydroxyl and carboxylate groups to olefin within the active site.^24^ The hydroxy lactonization of acid (*E*)-5-phenylpent-4-enoic acid (**10j**) by **Fe(**^**2,4,6-tri-iPr**^**iQ**_**2**_**dp)** or **Fe(**^**3,4,5-tri-F**^**iQ**_**2**_**dp)** catalysts to afford lactone **12j** (26% yield and 78% ee or 95% yield and 50% ee, respectively) further supports the role of such a chiral intermediate (Scheme 3C).

**Scheme 3:**
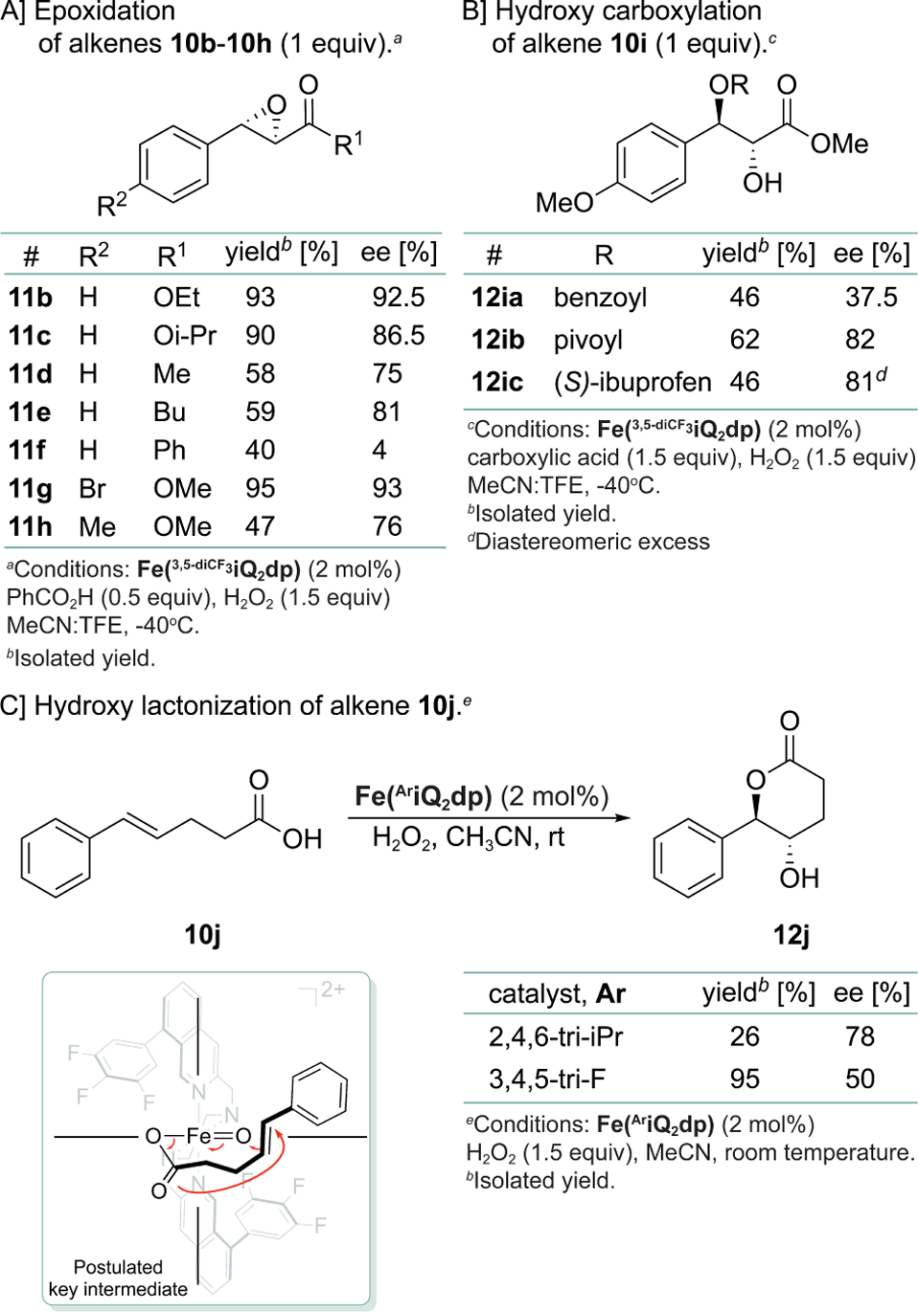
Asymmetric Oxidation of Alkenes

In conclusion, a new class of Fe^II^(^**Ar**^**iQ**_**2**_**dp**)(OTf)_2_ and Fe^II^(^**Ar**^**iQ**_**2**_**mc**)(OTf)_2_ catalysts for asymmetric oxidation reactions was designed and prepared. The divergent synthetic route to 8-aryl-3-formyl-isoquinolines offers an opportunity to explore metal catalysts characterized by a diverse range of structural and electronic attributes. The potential of these tetradentate iron catalysts to promote oxidation reactions is demonstrated for the highly enantioselective epoxidation and hydroxy carbonylation of conjugated alkenes. We intend to further explore the potential of these ligands in Fe- and Mn-catalyzed asymmetric oxidation and oxidative coupling reactions.

## Data Availability

The data underlying this study are available in the published article and its Supporting Information.

## Supporting Information Available

Experimental procedures, characterization data, and NMR spectra (PDF)
