# Supplementary material for: Chiral Bis-8-Aryl-isoquinoline Bis-alkylamine Iron Catalysts for Asymmetric Oxidation Reactions
Source: Org Lett. 2025 Jan 22;27(4):1078–83. doi: 10.1021/acs.orglett.5c00050 (PMC11791880; doi:10.1021/acs.orglett.5c00050)
Supplement: Supplementary file 1 — ol5c00050_si_001.pdf [file ol5c00050_si_001.pdf]

Supporting information

Chiral bis-8-Aryl-isoquinoline bis-Alkylamine-Iron Catalysts for Asymmetric Oxidation Reactions

Tomer Mintz,[a] Lei Liu,[b] and Doron Pappo[a,*]

[a] Department of Chemistry, Ben-Gurion University of the Negev, Beer-Sheva 84105, Israel

[b] School of Chemistry and Chemical Engineering, Shandong University, Jinan 250100, China

Email: pappod@bgu.ac.il

Table of Contents

1. *General information* *S2*

2. *The structures of the* ***Fe(^{Ar}iQ$_2$dp)(OTf)$_2$*** *and* ***Fe(^{Ar}iQ$_2$mc)(OTf)$_2$*** *catalysts* *S3*

3. *Ligands and Complexes preparation and characterization* *S4*

   3.1. Isoquinoline Synthesis S4

   3.2. 8-Aryl-3-formylisoquinolines (**8a-i**) Synthesis S7

   3.3. Ligand Synthesis S13

   3.1. Complex Synthesis S18

4. *Catalytic studies* *S20*

   4.1. Table S1. epoxidation of methyl cinnamate with different acids S20

   4.2. Table S2. epoxidation of alkyl cinnamates using iron ^{Ar}**iQ$_2$dp** and iron ^{Ar}**iQ$_2$mc** complexes S21

   4.3. General Procedure for enantioselective epoxidation S22

   4.4. General Procedure for selective hydroxy carboxylation S22

   4.5. Mechanistic Probing of Selective Hydroxy Carboxylation via Epoxide Testing S22

   4.6. Characterization data S23

5. *Crystallographic Data* *S26*

6. *Spectral (^{1}H & ^{13}C) data.* *S31*

7. *HPLC data analysis* *S144*

8. *References* *S156*

1. General information

All reagents were of reagent-grade quality, purchased commercially from Sigma-Aldrich, Alfa-Aesar, Angene Chemical, Apollo Scientific or Fluka, and used without further purification. Purification by column chromatography was performed on Sigma-Aldrich chromatographic silica gel (40-60 μm). TLC analyses were performed using Merck silica gel glass plates 60 F_{254}. NMR spectra were recorded on Bruker DPX400, or DMX500 instruments; chemical shifts, given in ppm, are relative to Me_4Si as an internal standard or to the residual solvent peak. HR-MS data were obtained using Thermo Scientific™ Q Exactive™ Hybrid Quadrupole-Orbitrap Mass Spectrometer. HPLC analysis was carried out on Agilent 1260 instrument equipped with a G4212-60008 photodiode array detector and an Agilent reverse phase ZORBAX Eclipse plus C18 3.5 μm column (4.6 X 100 mm) and Phenomenex chiral stationary phase Lux® 5 μm Cellulose-1 column (4.6 X 250 mm). The cone angles of the iron complexes were calculated using UCSF ChimeraX,[1] in conjunction with the SEQCROW plugin.[2] The cone of possible approach trajectories was calculate according to White (2013).[3] Olefins **10e**[4] and **10j**,[5] Fe(**iQ$_2$dp**)[6] were prepared according to literature procedures.

2. The structures of the Fe(^{Ar}iQ$_2$dp)(OTf)$_2$ and Fe(^{Ar}iQ$_2$mc)(OTf)$_2$ catalysts

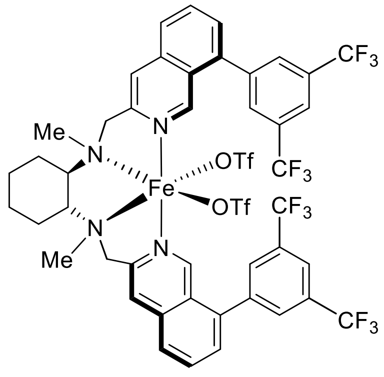

Fe($^{3,5\text{-di-}CF_3}$**iQ$_2$mc**)

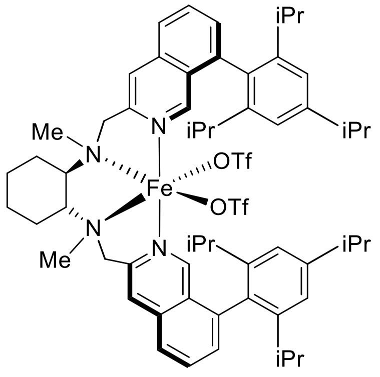

Fe($^{2,4,6\text{-tri-iPr}}$**iQ$_2$mc**)

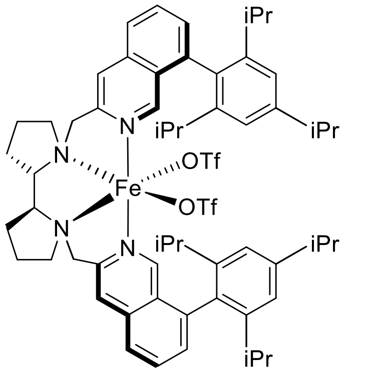

Fe($^{2,4,6\text{-tri-iPr}}$**iQ$_2$dp**)

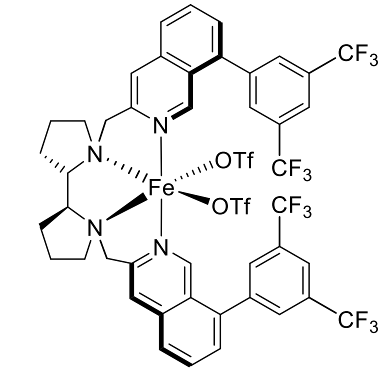

Fe($^{3,5\text{-di-}CF_3}$**iQ$_2$dp**)

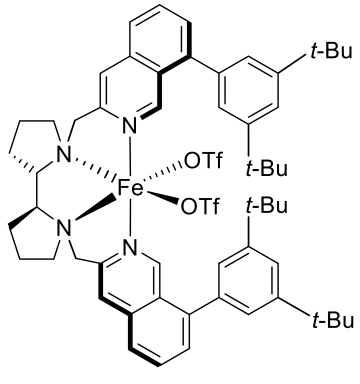

Fe($^{3,5\text{-di-}t\text{-Bu}}$**iQ$_2$dp**)

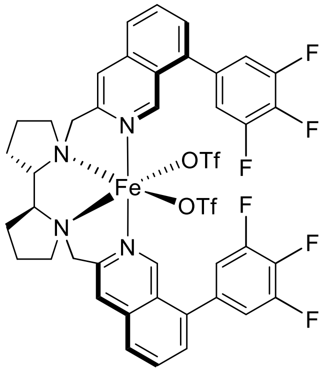

Fe($^{3,4,5\text{-tri-F}}$**iQdp**)

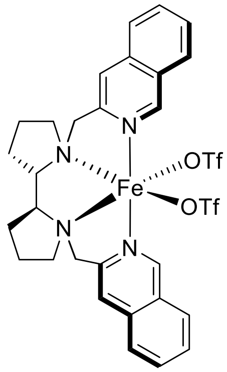

Fe(**iQ$_2$dp**)

3. Ligands and Complexes preparation and characterization

3.1. Isoquinoline Synthesis

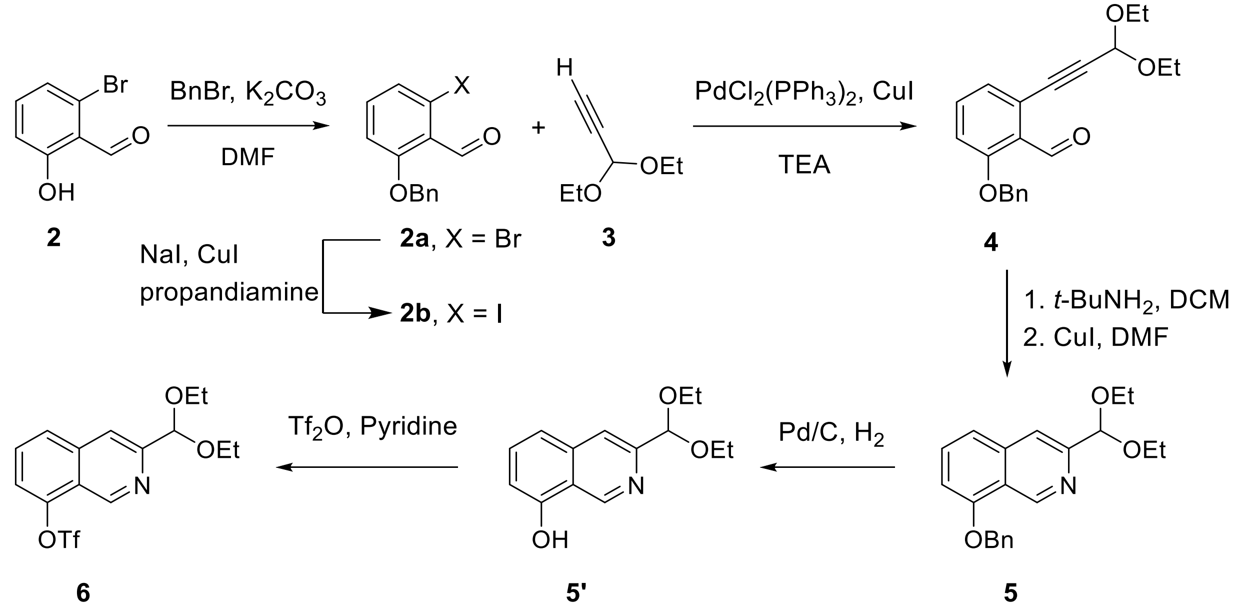

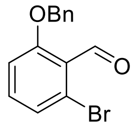

2a

Compound 2a: Benzyl bromide (1.1 equiv., 2.5 mL) was added to a solution of 2-bromo-6-hydroxybenzaldehyde (20 mmol, 4.04 g) and K_2CO_3 (2 equiv., 5.5 g) in DMF (1M, 20 mL). The reaction mixture was stirred at room temperature for 3h until complete consumption of the starting material was observed by TLC (ethyl-acetate/hexane 5:95). The mixture was diluted with water (50 mL) and extracted with diethyl ether (20 mL x3). The combined organic phase was washed with brine, dried with Na_2SO_4, and removed under reduced pressure, affording compound **2a** without further purification in quantitative yield as a light-yellow solid.
Characterization data of compound **2a**: **m.p.** 50-55°C. **^{1}H NMR (400 MHz, CDCl$_3$)** δ 10.48 (s, 1H), 7.47 – 7.24 (m, 7H), 7.00 (dd, J = 7.7, 1.7 Hz, 1H), 5.19 (s, 2H). **^{13}C{^{1}H} NMR (100 MHz, CDCl$_3$)** δ 190.2, 161.3, 135.8, 134.7, 128.9, 128.4, 127.2, 127.0, 124.4, 124.2, 112.7, 71.1. **HRMS (ESI) m/z:** $[M+H]^+$ calcd for $C_{14}H_{12}{}^{79}BrO_2$ 291.0015, found 291.0013.

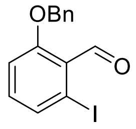

2b

Compound 2b: Compound **2b** was synthesized according to a modified procedure reported by Shin.[7] Note: NaI must be dehydrated before subjected to this reaction. Dehydration procedure: NaI was dried by sequential addition of toluene and evaporation of its water azeotrope under reduced pressure (2-3 times). The solid was kept under high vacuum for 24 hours, resulting in a fine white powder. The anhydrous NaI (4 equiv., 8.4 g), CuI (5 mol %, 140 mg), and 1,3-diaminopropane (10 mol %, 120 µL) were added to a solution of compound **2a** (14 mmol, 4.1 g) in dry 1,4-dioxane (1M, 14 mL). The reaction mixture was stirred in an oil bath at 90°C until complete consumption of the starting material was observed (2-5h, monitored by HPLC). The volatiles were removed under reduced pressure, and the crude residue was dissolved in ethyl acetate and filtered through a plug of celite. The filtrate was washed with aq. $NaHCO_3$, 1M HCl solution, and brine. The organic phase was dried with Na_2SO_4 and removed under reduced pressure. The crude product was purified by column chromatography (ethyl acetate/hexane 5:95), affording compound **2b** (4.2 g, 89% yield) as a light-yellow solid.

Characterization data of compound **2b**: **m.p.** 42-45°C. **^{1}H NMR (400 MHz, CDCl$_3$)** δ 10.32 (s, 1H), 7.61 (d, *J* = 7.5 Hz, 1H), 7.46 – 7.32 (m, 5H), 7.12 – 7.03 (m, 2H), 5.18 (s, 2H). **^{13}C{^{1}H} NMR (100 MHz, CDCl$_3$)** δ 191.3, 161.2, 135.8, 135.1, 134.3, 128.9, 128.5, 127.3, 125.6, 113.5, 95.8, 71.0. **HRMS (ESI) m/z:** $[M+H]^+$ calcd for $C_{14}H_{12}IO_2$ 338.9876, found 338.9874.

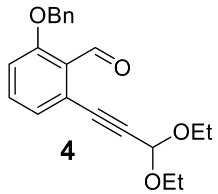

Compound 4 from **2a**: 3,3-Diethoxyprop-1-yne (1.3 equiv., 3.4 mL) was added to a solution of compound **2b** (18 mmol, 5.24 g) and Pd(PPh$_3$)$_2$Cl (5 mol %, 631 mg) in dry triethylamine (0.25M, 75 mL) under an argon atmosphere. The resultant mixture was stirred for 15 minutes at room temperature before the addition of CuI (1 mol %, 34 mg). The reaction mixture was then stirred in an oil bath at 70°C until complete consumption of the starting material was observed (4h, monitored by TLC). The mixture was filtered through a plug of celite, and the solvent was removed under reduced pressure. The crude product was purified by column chromatography (ethyl acetate/hexane 10:90), affording compound **4** (4.7-3 g, 50-78% yield) as an orange oil**.**

from **2b**: 3,3-Diethoxyprop-1-yne (1.1 equiv., 2.62 mL) was added to a solution of compound **2b** (16.6 mmol, 5.6 g) and Pd(PPh$_3$)$_2$Cl (2 mol %, 233 mg) in dry triethylamine (0.25M, 66 mL) under an argon atmosphere. The resultant mixture was stirred for 15 minutes at room temperature before the addition of CuI (1 mol %, 32 mg). The reaction mixture was then stirred in an oil bath at 50°C until complete consumption of the starting material was observed (1-3h, monitored by TLC). The mixture was filtered through a plug of celite, and the solvent was removed under reduced pressure. The crude product was then purified by column chromatography (ethyl acetate/hexane 10:90), affording compound **4** (4.7 g, 85% yield) as an orange oil**.** Characterization data of compound **4**: **^{1}H NMR (400 MHz, CDCl$_3$)** δ 10.63 (s, 1H), 7.56 – 7.26 (m, 6H), 7.19 (dd, *J* = 7.7, 0.9 Hz, 1H), 7.03 (dd, *J* = 8.5, 1.0 Hz, 1H), 5.53 (s, 1H), 5.19 (s, 2H), 3.87 (dq, *J* = 9.4, 7.1 Hz, 2H), 3.76 – 3.55 (m, 2H), 1.28 (t, *J* = 7.1 Hz, 6H). **^{13}C{^{1}H} NMR (100 MHz, CDCl$_3$)** δ 189.4, 160.3, 136.0, 134.3, 128.8, 128.3, 127.2, 126.1, 124.5, 114.0, 91.9, 90.4, 82.7, 70.8, 61.3, 15.3. **HRMS (ESI) m/z:** $[M+H]^+$ calcd for $C_{21}H_{22}O_4$ 339.1591, found 339.1588.

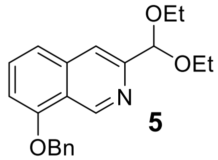

Compound 5: According to a modified procedure reported by Larock.[8] *tert*-Butylamine (4 equiv., 6 mL) was added to a stirred solution of compound **4** (14 mmol, 4.8 g) in DCE (0.5 M, 30 mL). The reaction mixture was stirred in an oil bath at 50°C under argon for 1.5h, and the solvent was then removed under reduced pressure**.** To a stirred solution of the afforded residue in dry DMF (0.1 M, 140 mL) was added CuI (10 mol %, 267 mg), and the reaction was stirred in an oil bath at 100°C under argon. After the complete consumption of the starting material (1-3h, monitored by TLC) the mixture was cooled to room temperature, diluted with aq. $NaHCO_3$ and extracted with diethyl ether (20 mL x3). The combined organic phase was washed with aq. NH_4Cl and brine, dried with Na_2SO_4, and the volatiles were removed under reduced pressure. The crude product was then purified by column chromatography (ethyl acetate/hexane 10:90), affording compound **5** (3.5 g, 75% yield) as a light-yellow oil. Characterization data of compound **5**: **^{1}H NMR (400 MHz, CDCl$_3$)** δ 9.72 (s, 1H), 7.89 (s, 1H), 7.64 – 7.23 (m, 7H), 6.93 (d, *J* = 7.8 Hz, 1H), 5.69 (s, 1H), 5.28 (s, 2H), 4.11 – 3.51 (m, 4H), 1.28 (t, *J* = 7.0 Hz, 6H). **^{13}C{^{1}H} NMR (100 MHz, CDCl$_3$)** δ 155.6, 152.2, 147.6, 137.6, 136.6, 131.1, 128.8, 128.2, 127.3, 120.8, 119.5, 117.2, 106.9, 102.5, 70.3, 62.1, 15.4. **HRMS (ESI) m/z:** $[M+H]^+$ calcd for $C_{21}H_{24}NO_3$ 338.1751, found 338.1745.

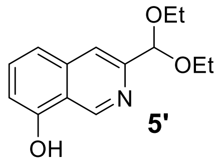

Compound 5': Methanol (0.03M, 180 mL) was added to a round flask containing compound **5** (6 mmol, 2 g) and Pd\C (10 mol %, 600 mg). After evacuation of the air atmosphere and back-filling the flask with hydrogen (1 atm, three times) the mixture was vigorously stirred in an oil bath at 30°C until complete consumption of the starting material (3-5h, monitored by TLC). The mixture was filtered through a celite bed and the solvent was removed under reduced pressure to afford compound **5'** (1.3 g, 88% yield) as a white solid.

Characterization data of compound **5'**: **m.p.** 145-150°C. **^{1}H NMR (400 MHz, CDCl$_3$)** δ 9.75 (s, 1H), 7.91 (s, 1H), 7.48 (t, *J* = 7.8 Hz, 1H), 7.29 (d, *J* = 8.2 Hz, 1H), 7.12 (s, 1H), 5.76 (s, 1H), 3.82 – 3.50 (m, 4H), 1.22 (t, *J* = 7.0 Hz, 6H). **^{13}C{^{1}H} NMR (100 MHz, CDCl$_3$)** δ 156.0, 149.8, 147.8, 137.9, 132.8, 120.6, 118.2, 117.4, 111.2, 101.8, 62.4, 15.3. **HRMS (ESI) m/z:** [M+]$^+$ calcd for $C_{14}H_{18}NO_3$ 248.1281, found 248.1278.

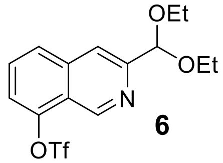

Compound 6: Triflic anhydride (1.2 equiv., 3.2 mL) in DCM (15 mL) was added dropwise at 0°C to a stirred solution of compound **5'** (15.5 mmol, 3.85 g) and pyridine (2 equiv., 2.5 mL) in DCM (0.1M, 140 mL). The reaction mixture was stirred at 0°C for 30 minutes, and at room temperature for another 30 minutes until complete consumption of the starting material was observed (monitored by TLC). The mixture was diluted with water and extracted with DCM (100 mL x3), the organic phase was washed with aq. NH_4Cl, brine and aq. $NaHCO_3$, dried with Na_2SO_4, and the volatiles were removed under reduced pressure. The compound was purified by column chromatography (ethyl acetate/hexane 13:87), affording compound **6** (5.5 g, 94% yield) as a light-yellow oil.

Characterization data of compound **6**: **m.p** 20-25°C. **^{1}H NMR (400 MHz, CDCl$_3$)** δ 9.53 (s, 1H), 8.04 (s, 1H), 7.91 (d, *J* = 8.5 Hz, 1H), 7.77 – 7.68 (m, 1H), 7.56 (dd, *J* = 7.8, 1.0 Hz, 1H), 5.71 (s, 1H), 3.95 – 3.47 (m, 4H), 1.29 (t, *J* = 7.1 Hz, 6H). **^{13}C{^{1}H} NMR (100 MHz, CDCl$_3$)** δ 153.6, 145.9, 145.8, 137.9, 130.3, 127.9, 121.3, 119.4, 118.83 (q, *J* = 323.1 Hz), 117.4, 102.1, 62.4, 15.4. **^{19}F NMR (376 MHz, CDCl$_3$)** δ -72.9. **HRMS (ESI) m/z:** [M+H]$^+$ calcd for $C_{15}H_{17}F_3NO_5S$ 380.0774, found 380.0769.

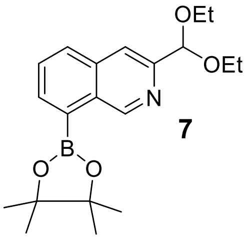

Compound 7: A solution of Compound **6** (2 mmol, 720 mg) in dioxane (20 mL) was added to a stirred solution of bis(pinacolato)diboron (3 equiv., 1.5 g), $Pd(dppf)_2Cl_2$ (3 mol %, 49 mg), and KOAc (3 equiv., 589 mg) in dioxane (20 mL) under air atmosphere. The mixture was stirred in an oil bath at 90 °C until complete consumption of the starting material was observed (1-3h, monitored by TLC). The mixture was cooled to room temperature, diethyl ether was added, the mixture was filtered and the volatiles were removed under reduced pressure. The crude product was then purified by column chromatography (ethyl acetate/hexane 14:86), affording compound **7** (598 mg, 84% yield) as a colorless oil.

Characterization data of compound **7**: **^{1}H NMR (400 MHz, CDCl$_3$)** δ 10.14 (s, 1H), 8.12 (d, *J* = 6.9 Hz, 1H), 7.93 – 7.89 (m, 2H), 7.65 (ddd, *J* = 8.4, 6.8, 1.6 Hz, 1H), 5.69 (s, 1H), 3.78 – 3.60 (m, 4H), 1.39 (d, *J* = 1.5 Hz, 12H), 1.27 (td, *J* = 7.1, 1.3 Hz, 6H). **^{13}C{1H} NMR (101 MHz, CDCl$_3$)** δ 152.7, 150.4, 136.3, 135.5, 131.2, 130.0, 129.0, 117.4, 101.9, 83.8, 61.5, 24.5, 14.9. **HRMS (ESI) m/z:** [M+H]$^+$ calcd for $C_{20}H_{28}{}^{11}BNO_4$ 358.2184, found 358.2183.

3.2. 8-Aryl-3-formylisoquinolines (**8a-i**) Synthesis

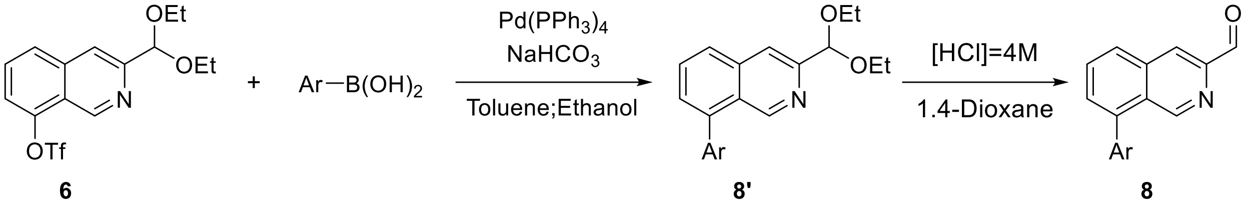

General procedure for Suzuki coupling: A solution of substituted aryl boronic acid (1 equiv.) in ethanol (0.3 M, 3 mL) was added to a stirred solution of compound **6** (1 equiv. 1 mmol, 380 mg), $Pd(PPh_3)_4$ (5 mol %, 60 mg) and aqueous 2M $NaHCO_3$ (2 equiv., 1 mL) in toluene (0.1 M, 10 mL) at room temperature under argon atmosphere. The mixture was stirred for 5 min before being heated in an oil bath to 80–90°C. The reaction was allowed to proceed for 2–4 hours, or until complete consumption of the starting materials (monitored by TLC). The mixture was cooled to room temperature and extracted with ethyl acetate. The organic layer was washed with water and brine, dried with Na_2SO_4, and removed under reduced pressure. The crude product was purified by column chromatography affording compounds **8'**.

General procedure for the acetal hydrolysis: Compounds **8'** (1 equiv.) were dissolved in dioxane (5 mL/mmol) and 4 M aqueous HCl solution (15 mL/mmol) in a 1:3 ratio. The mixture was stirred at room temperature overnight. After full deprotection (indicated by HPLC) the mixture was neutralized by the addition of saturated $NaHCO_3$ solution. The mixture was extracted with ethyl acetate, the organic layer was dried with Na_2SO_4 and the volatiles were removed under reduced pressure, affording compounds **8**.

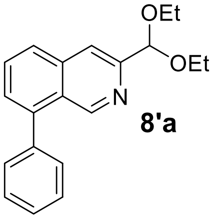

8'a

Compound 8'a: phenylboronic acid (122 mg) was reacted according to the Suzuki coupling general procedure. The compound was purified by column chromatography (ethyl acetate/hexane 10:90), affording compound **8'a** (292 mg, 95% yield) as a white amorphous solid.

Characterization data of compound **8'a**: **^{1}H NMR (400 MHz, $CDCl_3$)** δ 9.32 (s, 1H), 8.00 (s, 1H), 7.87 (d, *J* = 8.3 Hz, 1H), 7.73 (dd, *J* = 8.3, 7.1 Hz, 1H), 7.61 – 7.38 (m, 6H), 5.70 (s, 1H), 3.94 – 3.48 (m, 4H), 1.29 (t, *J* = 7.0 Hz, 6 H). **^{13}C{^{1}H} NMR (101 MHz, $CDCl_3$)** δ 151.3, 151.0, 141.2, 138.8, 136.8, 130.2, 128.6, 128.6, 128.5, 128.1, 126.8, 126.5, 117.9, 102.4, 62.2, 15.4. **HRMS (ESI) m/z:** $[M+H]^+$ calcd for $C_{20}H_{22}NO_2$ 308.1645, found 308.1644.

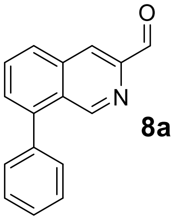

8a

Compound 8a: Compound **8'a** (377 mg, 0.9 mmol) was reacted according to the acetal hydrolysis general procedure, affording compound **8a** in quantitative yield as a white amorphous solid.

Characterization data of compound **8a**: **^{1}H NMR (400 MHz, $CDCl_3$)** δ 10.27 (s, 1H), 9.43 (s, 1H), 8.43 (s, 1H), 8.02 (d, *J* = 8.2 Hz, 1H), 7.84 (dd, *J* = 8.3, 7.2 Hz, 1H), 7.70 (dd, *J* = 7.1, 1.2 Hz, 1H), 7.59 – 7.44 (m, 5H). **^{13}C{^{1}H} NMR (101 MHz, $CDCl_3$)** δ 193.6, 152.1, 146.8, 141.6, 138.1, 135.9, 131.2, 131.0, 130.2, 128.8, 128.7, 128.4, 128.2, 121.6. **HRMS (ESI) m/z:** $[M+H]^+$ calcd for $C_{16}H_{12}NO$ 234.0913, found 234.0912.

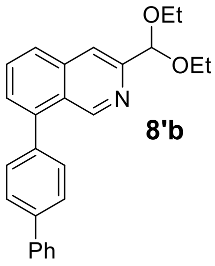

8'b

Compound 8'b: [1,1'-biphenyl]-4-ylboronic acid (198 mg) was reacted according to the Suzuki coupling general procedure. The compound was purified by column chromatography (ethyl acetate/hexane 10:90), affording compound **8'b** (337 mg, 88% yield) as a white amorphous solid.

Characterization data of compound **8'b**: **^{1}H NMR (400 MHz, CDCl$_3$)** δ 9.41 (s, 1H), 8.02 (s, 1H), 7.88 (d, *J* = 8.3 Hz, 1H), 7.80 – 7.65 (m, 5H), 7.64 – 7.55 (m, 3H), 7.49 (t, *J* = 7.5 Hz, 2H), 7.45 – 7.35 (m, 1H), 5.72 (s, 1H), 3.83 – 3.64 (m, 4H), 1.30 (t, *J* = 7.0 Hz, 6H). **^{13}C{^{1}H} NMR (101 MHz, CDCl$_3$)** δ 151.3, 150.8, 140.9, 140.6, 140.6, 137.6, 136.7, 130.5, 130.1, 128.9, 128.4, 127.6, 127.2, 127.2, 126.7, 126.4, 117.8, 102.3, 62.1, 15.3. **HRMS (ESI) m/z:** [M+H]$^+$ calcd for $C_{26}H_{26}NO_2$ 384.1958, found 384.1952.

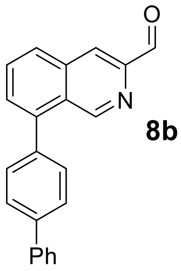

8b

Compound 8b: Compound **8'b** (307 mg, 0.8 mmol) reacted according to the acetal hydrolysis general procedure affording compound **8b** in quantitative yield as a white amorphous solid.

Characterization data of compound **8b: ^{1}H NMR (400 MHz, CDCl$_3$)** δ 10.29 (s, 1H), 9.52 (s, 1H), 8.45 (s, 1H), 8.04 (d, *J* = 9.3 Hz, 1H), 7.91 – 7.83 (m, 1H), 7.83 – 7.74 (m, 3H), 7.71 (dd, *J* = 7.3, 1.1 Hz, 2H), 7.66 – 7.58 (m, 2H), 7.51 (t, *J* = 7.9 Hz, 2H), 7.41 (t, *J* = 7.4 Hz, 1H). **^{13}C{^{1}H} NMR (101 MHz, CDCl$_3$)** δ 193.6, 152.1, 146.8, 141.4, 141.2, 140.5, 137.0, 136.0, 131.2, 131.1, 130.6, 129.1, 128.7, 128.3, 127.8, 127.5, 127.3, 121.7. **HRMS (ESI) m/z:** [M+H]$^+$ calcd for $C_{22}H_{16}NO$ 310.1226, found 310.1223.

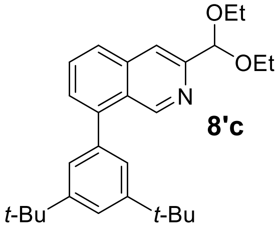

8'c

Compound 8'c: (3,5-Di-*tert*-butylphenyl)boronic acid (234 mg) was reacted according to the Suzuki coupling general procedure. The compound was purified by column chromatography (ethyl acetate/hexane 5:95), affording compound **8'c** (400 mg, 95% yield) as a white amorphous solid.

Characterization data of compound **8'c**: **^{1}H NMR (500 MHz, CDCl$_3$)** δ 9.36 (s, 1H), 8.02 (s, 1H), 7.87 (d, *J* = 8.2 Hz, 1H), 7.77 – 7.69 (m, 1H), 7.58 (dd, *J* = 7.1, 1.0 Hz, 1H), 7.54 (t, *J* = 1.8 Hz, 1H), 7.37 (d, *J* = 1.8 Hz, 2H), 5.72 (s, 1H), 3.81 – 3.61 (m, 4H), 1.40 (s, 18H), 1.31 (t, *J* = 7.1 Hz, 6H). **^{13}C{^{1}H} NMR (125 MHz, CDCl$_3$)** δ 151.3, 151.1, 151.0, 142.3, 137.9, 136.7, 130.1, 128.4, 126.7, 126.4, 124.5, 121.9, 117.8, 102.4, 62.1, 35.0, 31.6, 31.5, 31.5, 15.4. **HRMS (ESI) m/z:** [M+H]$^+$ calcd for $C_{28}H_{38}NO_2$ 420.2897, found 420.2892.

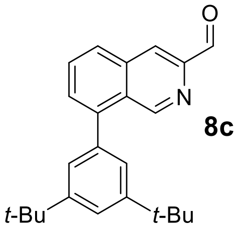

8c

Compound 8c: Compound **8'c** (378 mg, 0.9 mmol) reacted according to the acetal hydrolysis general procedure, affording compound **8c** quantitative yield as a white amorphous solid.

Characterization data of compound **8c**: **^{1}H NMR (400 MHz, CDCl$_3$)** δ 10.27 (s, 1H), 9.44 (s, 1H), 8.42 (d, *J* = 0.9 Hz, 1H), 8.00 (d, *J* = 8.2 Hz, 1H), 7.84 (dd, *J* = 8.2, 7.2 Hz, 1H), 7.73 (dd, *J* = 7.1, 1.2 Hz, 1H), 7.56 (t, *J* = 1.8 Hz, 1H), 7.34 (d, *J* = 1.9 Hz, 2H), 1.39 (s, 18H). **^{13}C{^{1}H} NMR (100 MHz, CDCl$_3$)** δ 193.4, 152.3, 151.2, 146.6, 142.7, 137.2, 135.8, 131.1, 130.9, 128.8, 127.8, 124.4, 122.3, 121.9, 35.0, 31.5. **HRMS (ESI) m/z:** [M+H]$^+$ calcd for $C_{24}H_{28}NO$ 346.2165, found 346.2161.

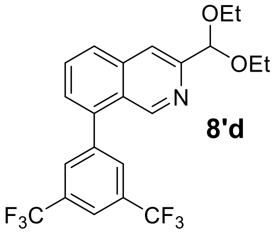

8'd

Compound 8’d: (3,5-Bis(trifluoromethyl)phenyl)boronic acid (258 mg) was reacted according to the Suzuki coupling general procedure. The compound was purified by column chromatography (ethyl acetate/hexane 5:95), affording compound **8’d** (410 mg, 92% yield) as a white amorphous solid.

Characterization data of compound **8’d**: **m.p.** 83-85°C. **^{1}H NMR (400 MHz, $CDCl_3$)** δ 9.17 (s, 1H), 8.05 (s, 1H), 8.01 – 7.97 (m, 3H), 7.96 (s, 1H), 7.78 (dd, *J* = 8.2, 7.2 Hz, 1H), 7.55 (dd, *J* = 7.1, 0.9 Hz, 1H), 5.70 (s, 1H), 4.10 – 3.39 (m, 4H), 1.30 (t, *J* = 7.1 Hz, 6H). **^{13}C{^{1}H} NMR (100 MHz, $CDCl_3$)** δ 152.2, 149.6, 140.9, 137.6, 136.9, 132.2 (q, *J* = 33.6 Hz), 130.5 – 130.1 (m), 130.1, 128.9, 128.4, 122.9 (q, *J* = 276.3 Hz), 122.2 – 122.0 (m), 118.1, 102.3, 62.3, 15.4. **HRMS (ESI) m/z:** $[M+H]^+$ calcd for $C_{22}H_{20}F_6NO_2$ 444.1393, found 444.1388.

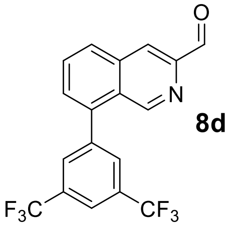

8d

Compound 8d: Compound **8’d** (399 mg, 0.9 mmol) was reacted according to the acetal hydrolysis general procedure, affording compound **8d** quantitative yield as a white solid.

Characterization data of compound **8d**: **m.p.** 135-140°C. **^{1}H NMR (400 MHz, $CDCl_3$)** δ 10.28 (s, 1H), 9.28 (s, 1H), 8.48 (s, 1H), 8.14 (d, *J* = 8.3 Hz, 1H), 8.05 (s, 1H), 8.00 (s, 2H), 7.91 (t, *J* = 7.7 Hz, 1H), 7.74 (d, *J* = 6.9 Hz, 1H). **^{13}C{^{1}H} NMR (100 MHz, $CDCl_3$)** δ 193.1, 150.6, 147.2, 140.2, 137.9, 136.0, 132.4 (q, *J* = 33.0 Hz), 131.4, 130.9, 130.2 – 130.0 (m), 129.7, 128.0, 123.1 (q, *J* = 273.2 Hz), 122.3 (p, *J* = 3.8 Hz), 121.4. **^{19}F NMR (376 MHz, $CDCl_3$)** δ -62.8. **HRMS (ESI) m/z:** $[M+H]^+$ calcd for $C_{18}H_{10}F_6NO$ 370.0661, found 370.0656.

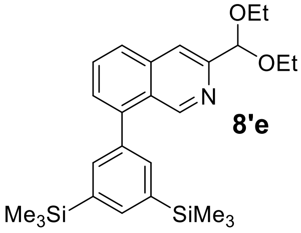

8'e

Compound 8’e: A solution of compound **7** (0.1 mmol, 36 mg) in ethanol (0.3 mL) was added at room temperature under an argon atmosphere to a stirred solution of (5-bromo-1,3-phenylene)bis(trimethylsilane) (1.1 equiv., 34 mg), $Pd(PPh_3)_4$ (5 mol %, 6 mg) and aq. 2M $NaHCO_3$ (2 equiv., 100 µL) in toluene (0.1 M, 1 mL). The mixture was stirred for 10 min before being heated in an oil bath to 80–90 °C until the starting materials were fully consumed (3h, monitored by HPLC). The mixture was cooled to room temperature and extracted with ethyl acetate. The organic layer was washed with water, brine, dried with Na_2SO_4 and the volatiles were removed under reduced pressure. The crude residue was purified by column chromatography (ethyl acetate/hexane 4:96), affording compound **8’e** (26 mg, 58% yield) as a white amorphous solid.

Characterization data of compound **8’e**: **^{1}H NMR (500 MHz, $CDCl_3$)** δ 9.30 (s, 1H), 8.01 (s, 1H), 7.88 (d, *J* = 8.3 Hz, 1H), 7.81 – 7.71 (m, 2H), 7.63 (d, *J* = 1.1 Hz, 2H), 7.54 (dd, *J* = 7.1, 0.9 Hz, 1H), 5.70 (s, 1H), 3.84 – 3.61 (m, 4H), 1.29 (t, *J* = 7.1 Hz, 6H), 0.32 (s, 18H). **^{13}C{^{1}H} NMR (126 MHz, $CDCl_3$)** δ 151.2, 151.1, 141.9, 140.0, 137.8, 137.2, 136.8, 135.4, 130.2, 128.7, 126.7, 126.7, 118.0, 102.4, 62.2, 15.4, -0.9. **HRMS (ESI) m/z:** $[M+H]^+$ calcd for $C_{26}H_{38}NO_2Si_2$ 452.2436, found 452.2433.

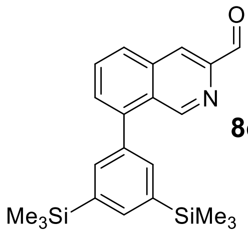

8e

Compound 8e: Compound **8'e** (23 mg, 0.05 mmol) was reacted according to the acetal hydrolysis general procedure, affording compound **8e** in quantitative yield as a white amorphous solid.

Characterization data of compound **8e**: **^{1}H NMR (400 MHz, $CDCl_3$)** 10.28 (s, 1H), 9.42 (s, 1H), 8.45 (s, 1H), 8.03 (d, *J* = 8.2 Hz, 1H), 7.92 – 7.84 (m, 1H), 7.79 (t, *J* = 1.2 Hz, 1H), 7.73 (dd, *J* = 7.2, 1.2 Hz, 1H), 7.64 (d, *J* = 1.2 Hz, 2H), 0.33 (s, 18H). **$^{13}C\{^1H\}$ NMR (100 MHz, $CDCl_3$)** δ 193.0, 151.7, 146.2, 141.8, 139.8, 137.6, 136.1, 135.4, 134.9, 130.8, 130.5, 128.3, 127.5, 121.4, -1.5. **HRMS (ESI) m/z:** $[M+H]^+$ calcd for $C_{22}H_{28}NOSi_2$ 378.1704, found 378.1700.

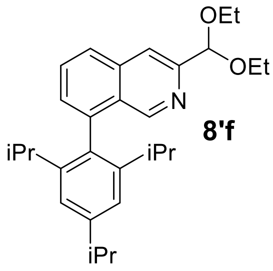

8'f

Compound 8'f: Argon gas was purged through dry toluene (4 mL, 0.25 M) for 30 minutes before being added under argon atmosphere to a flask containing compound **6** (1 equiv., 1 mmol, 380 mg), (2,4,6-triisopropylphenyl)boronic acid (1.5 equiv., 380 mg), $Pd(PPh_3)_4$ (10 mol %, 120 mg) and Cs_2CO_3 (2 equiv., 660 mg). The mixture was stirred for 10 min at room temperature before being heated in an oil bath to 100 °C until complete consumption of compound **6** was observed (24h, monitored by TLC). The mixture was cooled to room temperature and extracted with ethyl acetate. The organic layer was washed with water and brine, dried with Na_2SO_4 and the volatiles were removed under reduced pressure. The crude product was purified by column chromatography (ethyl acetate/hexane 4:96), affording compound **8'f** (250 mg, 58% yield) as a white amorphous solid.

Characterization data of compound **8'f**: **^{1}H NMR (500 MHz, $CDCl_3$)** δ 8.80 (s, 1H), 8.02 (s, 1H), 7.87 (d, *J* = 8.3 Hz, 1H), 7.72 (dd, *J* = 8.2, 7.1 Hz, 1H), 7.43 – 7.39 (m, 1H), 7.14 (s, 2H), 5.68 (s, 1H), 3.82 – 3.65 (m, 4H), 3.00 (hept, *J* = 6.9 Hz, 1H), 2.28 (hept, *J* = 6.8 Hz, 2H), 1.35 (d, *J* = 6.9 Hz, 6H), 1.29 (t, *J* = 7.1 Hz, 6H), 1.05 (d, *J* = 6.8 Hz, 6H), 0.97 (d, *J* = 6.9 Hz, 6H). **$^{13}C\{^1H\}$ NMR (125 MHz, $CDCl_3$)** δ 151.5, 151.1, 149.0, 147.3, 139.7, 136.4, 132.3, 130.0, 129.0, 128.2, 126.3, 120.9, 117.7, 102.6, 62.3, 34.5, 30.7, 24.9, 24.2, 23.8, 15.4. **HRMS (ESI) m/z:** $[M+Na]^+$ calcd for $C_{29}H_{39}NO_2Na$ 456.2873, found 456.2867.

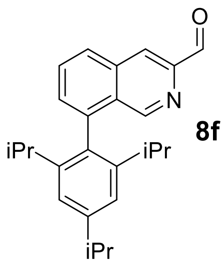

8f

Compound 8f: Compound **8'f** (217 mg, 0.5 mmol) was reacted according to the acetal hydrolysis general procedure, affording compound **8f** in quantitative yield as a white solid.

Characterization data of compound **8f**: **m.p.** 117-120°C. **^{1}H NMR (400 MHz, $CDCl_3$)** δ 10.23 (s, 1H), 8.92 (s, 1H), 8.43 (d, *J* = 1.0 Hz, 1H), 8.02 (d, *J* = 8.3 Hz, 1H), 7.83 (dd, *J* = 8.3, 7.1 Hz, 1H), 7.57 (dd, *J* = 7.0, 1.2 Hz, 1H), 7.14 (s, 2H), 2.99 (hept, *J* = 6.9 Hz, 1H), 2.22 (hept, *J* = 6.8 Hz, 2H), 1.34 (d, *J* = 6.9 Hz, 6H), 1.04 (d, *J* = 6.8 Hz, 6H), 0.95 (d, *J* = 6.9 Hz, 6H). **$^{13}C\{^1H\}$ NMR (100 MHz, $CDCl_3$)** δ 193.6, 152.1, 149.4, 147.2, 146.9, 140.2, 135.5, 131.6, 130.9, 130.2, 127.7, 121.5, 121.1, 34.4, 30.8, 24.7, 24.1, 23.8. **HRMS (ESI) m/z:** $[M+H]^+$ calcd for $C_{25}H_{30}NO$ 360.2322, found 360.2315.

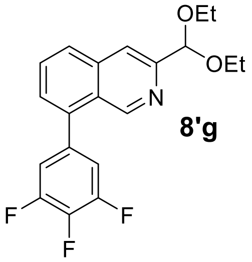
8'g

Compound 8’g: (3,4,5-trifluorophenyl)boronic acid (176 mg) was reacted according to the Suzuki coupling general procedure. The compound was purified by column chromatography (ethyl acetate/hexane 10:90), affording compound **8’g** (213 mg, 59% yield) as a white amorphous solid.

Characterization data of compound **8’g**: **^{1}H NMR (400 MHz, CDCl$_3$)** δ 9.24 (s, 1H), 8.01 (s, 1H), 7.91 (d, *J* = 8.3 Hz, 1H), 7.72 (dd, *J* = 8.3, 7.1 Hz, 1H), 7.47 (dd, *J* = 7.1, 1.2 Hz, 1H), 7.15 (dd, *J* = 8.2, 6.4 Hz, 2H), 5.69 (s, 1H), 3.79 – 3.65 (m, 4H), 1.29 (t, *J* = 7.1 Hz, 6H). **^{13}C{^{1}H} NMR (100 MHz, CDCl$_3$)** δ 152.0, 151.7 (ddd, *J* = 251.8, 10.0, 4.2 Hz), 149.9, 139.8 (dt, *J* = 253.9, 15.2 Hz), 137.8, 136.8, 134.7 (td, *J* = 7.5, 4.6 Hz), 130.0, 128.5, 128.0, 126.0, 118.0, 114.4 (dd, *J* = 15.7, 6.2 Hz), 102.3, 62.3, 15.4. **^{19}F NMR (376 MHz, CDCl$_3$)** δ -133.76 (d, *J* = 20.5 Hz), -160.94 (t, *J* = 20.5 Hz). **HRMS (ESI) m/z:** $[M+H]^+$ calcd for $C_{20}H_{19}F_3NO_2$ 362.1362, found 362.1356.

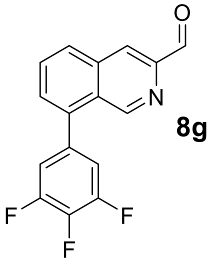
8g

Compound 8g: Compound **8’g** (180 mg, 0.5 mmol) reacted according to the acetal hydrolysis procedure, affording compound **8g** in quantitative yield as a white amorphous solid.

Characterization data of compound **8g**: **^{1}H NMR (400 MHz, CDCl$_3$)** δ 10.27 (s, 1H), 9.36 (s, 1H), 8.45 (s, 1H), 8.08 (d, *J* = 8.3 Hz, 1H), 7.90 – 7.81 (m, 1H), 7.66 (d, *J* = 7.2 Hz, 1H), 7.17 (dd, *J* = 7.7, 6.3 Hz, 2H). **^{13}C{^{1}H} NMR (100 MHz, CDCl$_3$)** δ 193.4, 151.5 (ddd, *J* = 252.6, 9.8, 3.7 Hz), 151.0, 147.2, 140.1 (dt, *J* = 254.5, 14.3 Hz), 138.2, 136.0, 134.1 (t, *J* = 5.9 Hz), 131.1, 130.9, 129.4, 128.2, 121.4, 114.5 (dd, *J* = 15.7, 6.1 Hz). **^{19}F NMR (376 MHz, CDCl$_3$)** δ -133.1 (d, *J* = 20.3 Hz), -160.1 (t, *J* = 21.0 Hz). **HRMS (ESI) m/z:** $[M+H]^+$ calcd for $C_{16}H_9F_3NO$ 288.0631, found 288.0626.

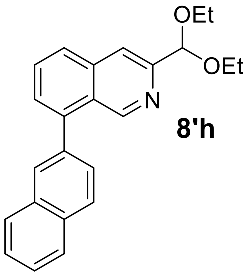
8'h

Compound 8’h: naphthalen-2-ylboronic acid (172 mg) was reacted according to the Suzuki coupling general procedure. The compound was purified by column chromatography (ethyl acetate/hexane 10:90), affording compound **8’h** (329 mg, 92% yield) as a white amorphous solid.

Characterization data of compound **8’h:** **^{1}H NMR (400 MHz, CDCl$_3$)** δ 9.40 (s, 1H), 8.03 (s, 1H), 8.02 – 7.84 (m, 5H), 7.77 (dd, *J* = 8.3, 7.1 Hz, 1H), 7.70 – 7.60 (m, 2H), 7.64 – 7.50 (m, 2H), 5.72 (s, 1H), 3.83 – 3.64 (m, 4H), 1.30 (t, *J* = 7.0 Hz, 6H). **^{13}C{^{1}H} NMR (101 MHz, CDCl$_3$)** δ 151.4, 151.0, 141.1, 136.9, 136.3, 133.5, 133.0, 130.2, 129.2, 128.9, 128.3, 128.2, 128.2, 127.9, 126.9, 126.7, 126.6, 126.6, 117.9, 102.4, 62.3, 15.4. **HRMS (ESI) m/z:** $[M+H]^+$ calcd for $C_{24}H_{24}NO_2$ 358.1802, found 358.1797.

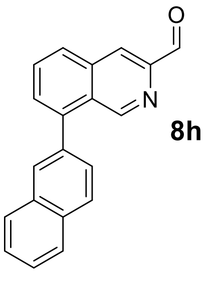
8h

Compound 8h: Compound **8’h** (322 mg, 0.9 mmol) was reacted according to the acetal hydrolysis general procedure, affording compound **8h** in quantitative yield as a white amorphous solid.

Characterization data of compound **8h**: **^{1}H NMR (400 MHz, CDCl$_3$)** δ 10.29 (s, 1H), 9.50 (s, 1H), 8.46 (s, 1H), 8.13 – 7.75 (m, 7H), 7.65 (dd, *J* = 8.4, 1.6 Hz, 1H), 7.59 (dd, *J* = 6.3, 3.3 Hz, 2H). **^{13}C{^{1}H} NMR (101 MHz, CDCl$_3$)** δ 193.6, 152.2, 146.8, 141.5, 136.0, 135.6, 133.4, 133.0, 131.5, 131.1, 129.4, 128.8, 128.5, 128.3, 128.3, 128.0, 127.9, 127.0, 126.8, 121.7. **HRMS (ESI) m/z:** $[M+H]^+$ calcd for $C_{20}H_{14}NO$ 284.1070, found 284.1065.

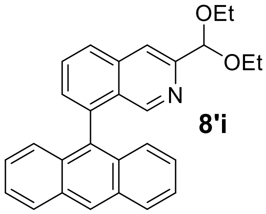

8'i

Compound 8’i: A solution of compound **7** (0.1 mmol, 36 mg) in ethanol (0.3 mL) was added at room temperature under an argon atmosphere to a stirred solution of 9-bromoanthracene (1.1 equiv., 28 mg), $Pd(PPh_3)_4$ (5 mol %, 6 mg) and aq. 2M $NaHCO_3$ (2 equiv., 100 µL) in toluene (0.1 M, 1 mL). The mixture was stirred for 10 min at room temperature before being heated in an oil bath to 80–90 °C until complete consumption of the starting materials (3h, monitored by HPLC). The mixture was cooled to room temperature and extracted with ethyl acetate. The organic layer was washed with water, brine, dried with Na_2SO_4 and the volatiles were removed under reduced pressure. The crude product was purified by column chromatography (ethyl acetate/hexane 4:96), affording compound **8’i** (30 mg, 73% yield) as a white amorphous solid.

Characterization data of compound **8’i**: **^{1}H NMR (400 MHz, CDCl$_3$)** δ 8.61 (s, 1H), 8.44 (s, 1H), 8.21 – 8.02 (m, 4H), 7.93 – 7.85 (m, 1H), 7.63 (d, *J* = 7.0 Hz, 1H), 7.45 (ddd, *J* = 8.4, 6.2, 1.6 Hz, 2H), 7.35 – 7.21 (m, 4H), 5.60 (s, 1H), 3.81 – 3.61 (m, 4H), 1.27 (t, *J* = 7.0 Hz, 6H). **^{13}C{^{1}H} NMR (101 MHz, CDCl$_3$)** δ 151.8, 151.4, 137.7, 136.7, 131.4, 131.2, 130.7, 130.3, 128.7, 128.5, 127.7, 127.4, 126.6, 126.4, 126.0, 125.4, 117.8, 102.8, 62.6, 15.4. **HRMS (ESI) m/z:** $[M+H]^+$ calcd for $C_{28}H_{26}NO_2$ 408.1958, found 408.1954.

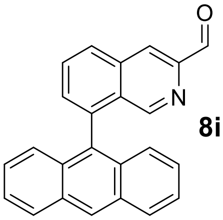

8i

Compound 8i: Compound **8’i** (20 mg, 0,05 mmol) was reacted according to the acetal hydrolysis general procedure, affording compound **8i** in quantitative yield as a white amorphous solid.

Characterization data of compound **8i: ^{1}H NMR (400 MHz, CDCl$_3$)** δ 10.20 (s, 1H), 8.66 (s, 1H), 8.60 (s, 1H), 8.55 (s, 1H), 8.22 (d, *J* = 8.4 Hz, 1H), 8.13 (d, *J* = 9.0 Hz, 2H), 8.01 (dd, *J* = 8.3, 7.0 Hz, 1H), 7.81 (dd, *J* = 7.0, 1.2 Hz, 1H), 7.53 – 7.42 (m, 2H), 7.29 (d, *J* = 3.1 Hz, 4H). **^{13}C{^{1}H} NMR (101 MHz, CDCl$_3$)** δ 193.7, 152.4, 147.0, 138.2, 135.9, 133.5, 131.6, 131.4, 131.3, 131.2, 130.5, 128.9, 128.8, 128.2, 126.4, 126.1, 125.5, 121.4. **HRMS (ESI) m/z:** $[M+H]^+$ calcd for $C_{24}H_{16}NO$ 334.1226, found 334.1225.

3.3. Ligand Synthesis

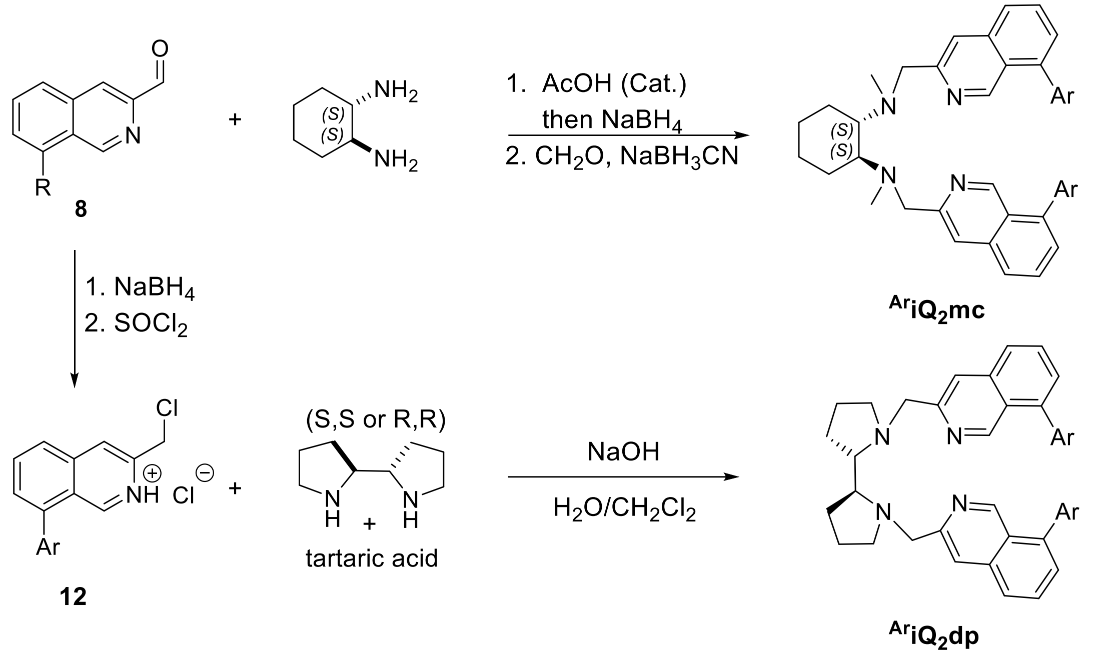

3.3.1. General Procedure for the Preparation of ^{Ar}iQ$_2$mc Ligands: A solution of compound **8** (2 equiv., 0.5 mmol) in methanol (1 M, 0.5 mL) was added to (1*S*,2*S*)-cyclohexane-1,2-diamine (1 equiv., 0.25 mmol, 28.5 mg) and acetic acid (5 mol %, 1 µL) in methanol (0.5 M, 0.5 mL) at 0°C. The mixture was stirred at room temperature overnight. Volatiles were removed under reduced pressure, and the crude product was dissolved in methanol (0.1 M, 2.5 ml) and stirred at 0°C. Sodium borohydride (2.5 equiv., 24 mg) was added in portions, and the mixture was stirred at room temperature for 2h, then quenched with 2M aq. NaOH and extracted with dichloromethane. The organic phase was dried with Na_2SO_4 and the volatiles were removed under reduced pressure. A mixture of the residue and acetic acid (5 mol %, 1 µL) in acetonitrile (0.25 M, 1 mL) was treated with 30% aqueous formaldehyde solution (8 equiv., 200 µL) and stirred at room temperature for 2 hours. Sodium cyanoborohydride (4 equiv., 63 mg) was then added, and the mixture was stirred overnight. The reaction was quenched with 2M aqueous NaOH and extracted with dichloromethane, dried with Na_2SO_4, and the solvent was removed under reduced pressure. The residue was purified by column chromatography affording the **^{Ar}iQ$_2$mc** ligands.

3.3.2. General Procedure for the Preparation of 3-(chloromethyl)isoquinolines (12): Sodium borohydride (2 equiv., 60 mg) was added in portions to a solution of compound **8** (1 equiv., 0.8 mmol) in ethanol (0.25 M, 3 mL). The mixture was stirred at room temperature until the starting materials were fully consumed (1-3h, monitored by TLC). The mixture was diluted with water and extracted with dichloromethane, the organic layer was dried over Na_2SO_4. The volatiles were removed under reduced pressure, and the residue was purified by column chromatography to afford the alcohol intermediate.

Thionyl chloride (5 equiv., 290 µL) was added slowly to a stirred solution of the alcohol intermediate in dichloromethane (0.05 M, 16 mL) at 0°C. The reaction mixture was then stirred at room temperature overnight. Volatiles were removed under reduced pressure to yield the crude 3-(chloromethyl)isoquinoline hydrochlorides **12**, which were used in the next step without further purification.

3.3.3. General Procedure for the Preparation of ^{Ar}iQ$_2$dp ligands: The synthesis was carried out according to a procedure reported by Costas.[9] A solution of (*S,S*)-2,2'-bipyrrolidine D-tartrate (1 equiv., 0.5 mmol, 144 mg) and NaOH (6.5 equiv., 130 mg) in H_2O (0.5 M, 1 mL) was added to compound **12** (2.1 equiv., 1.05 mmol) dissolved in dichloromethane (0.5 M, 1 mL). The mixture was stirred overnight at room temperature. The aqueous phase was extracted with dichloromethane and the combined organic layers were dried over Na_2SO_4, and the volatiles were removed under reduced pressure, affording the crude product that was purified by column chromatography.

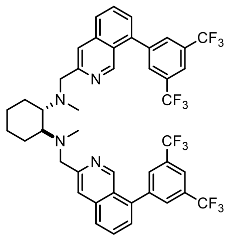

$^{3,5\text{-(di-CF3)}}$iQ$_2$mc

(*S,S*)-$^{3,5\text{-(di-CF3)}}$**iQ$_2$mc Ligand:** Compound **8d** (184 mg) reacted according to the General Procedure for the Preparation of ^{Ar}**iQ$_2$mc** Ligands. The crude product was purified by column chromatography (MeOH/$CHCl_3$ 2:98 to TEA/MeOH/$CHCl_3$ 1:2:97), affording ligand (*S,S*)-$^{3,5\text{-(di-CF3)}}$**iQ$_2$mc** (105 mg, 50% yield) as a light yellow amorphous solid.

Characterization data of ligand (*S,S*)-$^{3,5\text{-(di-CF3)}}$**iQ$_2$mc**: $[\alpha]_D^{23} = -26$ (c = 0.03 , DCM); **^{1}H NMR (400 MHz, $CDCl_3$)** δ 9.09 (s, 2H), 7.99 (d, *J* = 7.0 Hz, 8H), 7.68 – 7.51 (m, 4H), 7.45 (dd, *J* = 5.5, 2.7 Hz, 2H), 4.17 (d, *J* = 15.1 Hz, 2H), 4.03 (d, *J* = 15.2 Hz, 2H), 2.81 (q, *J* = 10.1 Hz, 2H), 2.44 (s, 6H), 2.15 – 2.02 (m, 2H), 1.84 – 1.77 (m, 2H), 1.52 – 1.31 (m, 2H), 1.27 – 1.16 (m, 2H). **^{13}C{^{1}H} NMR (101 MHz, $CDCl_3$)** δ 155.4, 149.2, 141.2, 137.6, 137.2, 132.2 (q, *J* = 33.6 Hz), 130.4 – 129.9 (m), 129.6, 128.0, 127.9, 125.2, 123.4 (q, *J* = 273.1 Hz), 121.9 (q, *J* = 3.4 Hz), 118.9, 64.9, 60.2, 37.1, 26.1, 26.0. **^{19}F NMR (376 MHz, $CDCl_3$)** δ -62.8. **HRMS (ESI) m/z:** $[M+H]^+$ calcd for $C_{44}H_{37}F_{12}N_4$ 849.2821, found 849.2816.

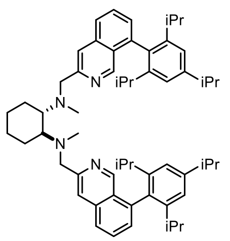

$^{2,4,6\text{-(tri-iPr)}}iQ_2$mc

(*S,S*)-$^{2,4,6\text{-(tri-iPr)}}$**iQ$_2$mc Ligand:** Compound **8f** (180 mg) was reacted according to General Procedure for the Preparation of ^{Ar}**iQ$_2$mc** Ligands. The product was purified by column chromatography (MeOH/$CHCl_3$ 2:98 to TEA/MeOH/$CHCl_3$ 1:2:97)) affording ligand (*S,S*)-$^{2,4,6\text{-(tri-iPr)}}$**iQ$_2$mc** (81 mg, 42% yield) as a light yellow amorphous solid.

Characterization data of ligand (*S,S*)-$^{2,4,6\text{-(tri-iPr)}}$**iQ$_2$mc**: $[\alpha]_D^{23} = -40$ (c = 0.001 , DCM); **^{1}H NMR (400 MHz, $CDCl_3$)** δ 8.72 (s, 2H), 7.92 (s, 2H), 7.62 – 7.50 (m, 4H), 7.32 (d, *J* = 6.5 Hz, 2H), 7.12 (s, 4H), 4.11 (d, *J* = 15.1 Hz, 2H), 3.97 (d, *J* = 15.1 Hz, 2H), 2.99 (hept, *J* = 6.8 Hz, 2H), 2.78 (q, *J* = 8.3 Hz, 2H), 2.43 (s, 6H), 2.30 (dp, *J* = 24.5, 6.7 Hz, 4H), 2.11 – 2.00 (m, 4H), 1.84 – 1.69 (m, 2H), 1.34 (d, *J* = 7.0 Hz, 12H), 1.18 (t, *J* = 9.9 Hz, 2H), 1.05 (d, *J* = 10.7 Hz, 6H), 1.03 (d, *J* = 10.9 Hz, 6H), 0.94 (d, *J* = 9.7 Hz, 6H), 0.93 (d, *J* = 9.8 Hz, 6H). **^{13}C{^{1}H} NMR (101 MHz, $CDCl_3$)** δ 154.6, 150.8, 148.9, 147.4, 147.3, 139.7, 136.8, 132.7, 129.6, 128.0, 127.4, 125.8, 121.0, 118.5, 64.7, 60.1, 37.0, 34.5, 30.8, 26.0, 25.9, 25.0, 24.3, 24.0, 23.9. **HRMS (ESI) m/z:** $[M+H]^+$ calcd for $C_{58}H_{77}N_4$ 829.6143, found 829.6132.

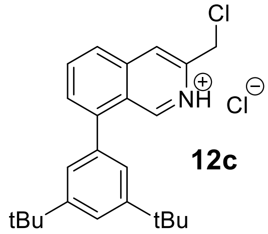

12c

Compound 12c: Compound **8c** (460 mg, 1.33 mmol) was reacted according to the General Procedure for the Preparation of3-(chloromethyl)isoquinolines, affording compound **12c** (495 mg, 90% yield) as a white amorphous solid.

Characterization data of compound **12c:** **^{1}H NMR (500 MHz, $CDCl_3$)** δ 9.30 (s, 1H), 8.36 (s, 1H), 8.19 – 8.08 (m, 2H), 7.87 (dd, *J* = 7.0, 1.2 Hz, 1H), 7.62 (t, *J* = 1.8 Hz, 1H), 7.20 (d, *J* = 1.8 Hz, 2H), 5.27 (s, 2H), 1.37 (s, 18H). **^{13}C{^{1}H} NMR (126 MHz, $CDCl_3$)** δ 152.2, 145.7, 145.5, 141.6, 139.4, 136.5, 135.2, 131.7, 126.4, 126.0, 124.7, 124.1, 123.9, 40.0, 35.2, 31.5. **HRMS (ESI) m/z:** $[M-Cl]^+$ calcd for $C_{24}H_{29}ClN$ 366.1983, found 366.1979.

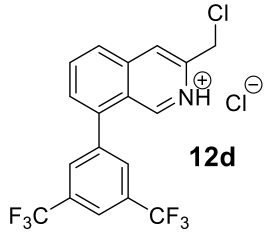

12d

Compound 12d: Compound **8d** (396 mg, 1.0 mmol) reacted according to General Procedure for the Preparation of 3-(chloromethyl)isoquinolines, affording compound **12d** (399 mg, 94% yield) as a white amorphous solid.

Characterization data of compound **12d**: **^{1}H NMR (400 MHz, DMSO)** δ 9.12 (s, 1H), 8.31 (s, 2H), 8.27 (s, 1H), 8.25 (s, 1H), 8.19 (d, *J* = 8.4 Hz, 1H), 8.00 (td, *J* = 7.8, 1.8 Hz, 1H), 7.82 (d, *J* = 7.4 Hz, 1H), 5.03 (s, 2H). **^{13}C{^{1}H} NMR (101 MHz, DMSO)** δ 149.1, 148.4 – 147.6 (m), 140.3, 137.4, 136.7, 131.9, 130.9 – 130.7 (m), 130.7 (q, *J* = 32.9 Hz), 130.4, 127.9, 124.9, 123.8 (q, *J* = 273.3 Hz), 122.2 – 121.8 (m), 121.0, 45.6. **^{19}F NMR (376 MHz, DMSO)** δ -61.1. **HRMS (ESI) m/z:** $[M-Cl]^+$ calcd for $C_{18}H_{11}ClF_6N$ 390.0479, found 390.0477.

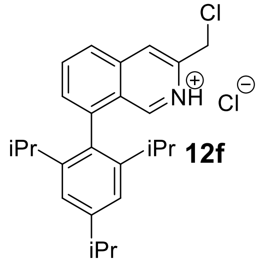

12f

Compound 12f: Compound **8f** (290 mg, 0.8 mmol) reacted according to the General Procedure for the Preparation of 3-(chloromethyl)isoquinolines, affording compound **12f** (289 mg, 86% yield) as a white amorphous solid.

Characterization data of compound **12f**: **^{1}H NMR (500 MHz, $CDCl_3$)** δ 8.90 (s, 1H), 8.41 (s, 1H), 8.19 – 8.10 (m, 2H), 7.72 (dd, *J* = 6.1, 2.1 Hz, 1H), 7.15 (s, 2H), 5.33 (s, 2H), 3.00 (hept, *J* = 6.9 Hz, 1H), 2.03 (hept, *J* = 6.8 Hz, 2H), 1.36 (d, *J* = 7.0 Hz, 6H), 1.01 (d, *J* = 6.9 Hz, 6H), 0.96 (d, *J* = 6.9 Hz, 6H). **^{13}C{^{1}H} NMR (126 MHz, $CDCl_3$)** δ 150.9, 147.1, 144.2, 143.5, 142.3, 139.4, 136.4, 132.7, 129.0, 127.4, 126.6, 125.0, 121.9, 40.0, 34.6, 31.1, 25.0, 24.1, 23.8. **HRMS (ESI) m/z:** $[M-Cl]^+$ calcd for $C_{25}H_{31}ClN$ 380.2139, found 380.2136.

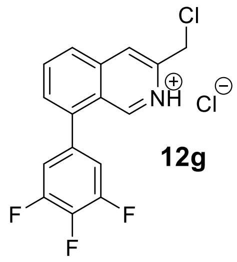

12g

Compound 12g: Compound **8g** (330 mg, 1.15 mmol) reacted according to General Procedure for the Preparation of 3-(chloromethyl)isoquinolines, affording compound **12g** (245 mg, 62% yield) as a white amorphous solid.

Characterization data of compound **12g**: **^{1}H NMR (400 MHz, $CDCl_3$)** δ 9.26 (s, 1H), 8.40 (s, 1H), 8.23 – 8.12 (m, 2H), 7.82 (dd, *J* = 6.6, 1.0 Hz, 1H), 7.12 (dd, *J* = 7.3, 6.0 Hz, 2H), 5.26 (s, 2H). **^{13}C{^{1}H} NMR (101 MHz, $CDCl_3$)** 151.8 (ddd, *J* = 253.5, 10.1, 3.1 Hz), 144.6, 143.0, 140.8 (dt, *J* = 256.8, 13.7 Hz), 140.5, 139.5, 136.5 – 136.2 (m), 136.1, 131.9, 128.1, 125.3, 124.9, 114.6 (dd, *J* = 15.6, 7.3 Hz), 40.3. **^{19}F NMR (376 MHz, $CDCl_3$)** δ -130.9 (d, *J* = 20.5 Hz), -157.8 (t, *J* = 20.4 Hz). **HRMS (ESI) m/z:** $[M-Cl]^+$ calcd for $C_{16}H_{10}ClF_3N$ 308.0446, found 308.0446.

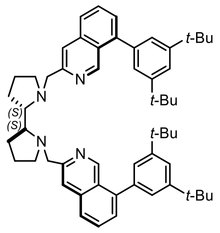

$^{3,5\text{-(di-t-Bu)}}$**iQ$_2$dp**

(*S,S*)-$^{3,5\text{-(di-}t\text{-Bu)}}$**iQ$_2$dp Ligand:** Compound **12c** (423 mg) and reacted according to the General Procedure for the Preparation of ^{Ar}**iQ$_2$dp** ligands. The product was purified by column chromatography (MeOH/$CHCl_3$ 2:98 to TEA/MeOH/$CHCl_3$ 1:2:97), affording ligand (*S,S*)-$^{3,5\text{-(di-}t\text{-Bu)}}$**iQ$_2$dp** (340 mg, 85% yield) as a light yellow solid.

Characterization data of ligand (*S,S*)-$^{3,5\text{-(di-}t\text{-Bu)}}$**iQ$_2$dp**: $[\alpha]_D^{23} = -44$ (c = 0.05, DCM); **m.p.** 115-120°C. **^{1}H NMR (500 MHz, $CDCl_3$)** δ 9.23 (s, 2H), 7.76 – 7.69 (m, 4H), 7.65 (dd, *J* = 8.2, 7.0 Hz, 2H), 7.51 (t, *J* = 1.8 Hz, 2H), 7.47 (dd, *J* = 7.0, 1.2 Hz, 2H), 7.32 (d, *J* = 1.8 Hz, 4H), 4.44 (d, *J* = 14.6 Hz, 2H), 3.71 (d, *J* = 14.6 Hz, 2H), 3.22 – 3.08 (m, 2H), 3.06 – 2.89 (m, 2H), 2.33 (q, *J* = 8.8 Hz, 2H), 2.00 – 1.83 (m, 4H), 1.82 – 1.68 (m, 4H), 1.38 (s, 36H). **^{13}C{^{1}H} NMR (126 MHz, $CDCl_3$)** δ 153.4, 150.9, 150.9, 142.1, 138.1, 136.9, 129.7, 127.5, 125.8, 125.8, 124.4, 121.8, 118.5, 66.0, 61.2, 55.5, 35.0, 31.6, 26.3, 23.7. **HRMS (ESI) m/z:** $[M+H]^+$ calcd for $C_{56}H_{71}N_4$ 799.5673, found 799.5667.

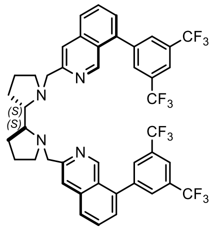

$^{3,5\text{-(di-CF3)}}$**iQ$_2$dp**

(*S,S*)-$^{3,5\text{-(di-CF3)}}$**iQ$_2$dp Ligand:** Compound **12d** (447 mg) and reacted according to the General Procedure for the Preparation of ^{Ar}**iQ$_2$dp** ligands. The product was purified by column chromatography (MeOH/$CHCl_3$ 2:98 to TEA/MeOH/$CHCl_3$ 1:2:97), affording ligand (*S,S*)-$^{3,5\text{-(di-CF3)}}$**iQ$_2$dp** (313 mg, 74% yield) as a light yellow solid.

Characterization data of compound (*S,S*)-$^{3,5\text{-(di-CF3)}}$**iQ$_2$dp**: $[\alpha]_D^{23} = -6$ (c = 0.03, DCM); **m.p.** 110-115°C. **^{1}H NMR (400 MHz, $CDCl_3$)** δ 8.96 (s, 2H), 7.98 (s, 2H), 7.87 (s, 4H), 7.79 (d, *J* = 8.3 Hz, 2H), 7.73 (s, 2H), 7.66 (t, *J* = 7.7 Hz, 2H), 7.40 (d, *J* = 7.1 Hz, 2H), 4.59 (d, *J* = 15.1 Hz, 2H), 3.76 (d, *J* = 15.1 Hz, 2H), 3.21 – 3.12 (m, 2H), 3.00 – 2.91 (m, 2H), 2.38 – 2.28 (m, 2H), 2.03 – 1.91 (m, 2H), 1.90 – 1.72 (m, 6H). **^{13}C{^{1}H} NMR (101 MHz, $CDCl_3$)** δ 154.9 – 153.8 (m), 149.2, 141.1, 137.4, 137.0, 132.1 (q, *J* = 33.7 Hz), 130.1 (q, *J* = 3.5 Hz), 129.7, 128.0, 127.8, 124.8, 123.3 (q, *J* = 272.6 Hz), 121.9 (q, *J* = 3.7 Hz), 118.5, 67.1, 61.7, 55.6, 27.2, 23.9. **^{19}F NMR (376 MHz, $CDCl_3$)** δ -62.8. **HRMS (ESI) m/z:** $[M+H]^+$ calcd for $C_{44}H_{35}F_{12}N_4$ 847.2665, found 847.2659.

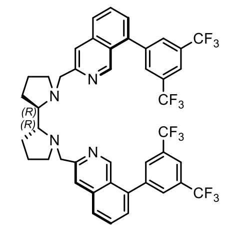

(*R,R*)-$^{3,5\text{-(di-CF3)}}$**iQ$_2$dp**

(*R,R*)-$^{3,5\text{-(di-CF3)}}$**iQ$_2$dp Ligand:** Compound **12d** (447 mg) and (*R,R*)-2,2'-bipyrrolidine L-tartrate reacted according to ^{Ar}iQ$_2$dp ligands general procedure. The product was purified by column chromatography (MeOH/$CHCl_3$ 2:98 to TEA/MeOH/$CHCl_3$ 1:2:97)) affording ligand (*R,R*)-$^{3,5\text{-(di-CF3)}}$**iQ$_2$dp** (228 mg, 54% yield) as a light yellow solid.

Characterization data of compound (*R,R*)-$^{3,5\text{-(di-CF3)}}$**iQ$_2$dp**: $[\alpha]_D^{23} = +6$ (c = 0.05, DCM); **m.p.** 110-115°C. **^{1}H NMR (400 MHz, $CDCl_3$)** δ 8.95 (s, 2H), 7.97 (s, 2H), 7.87 (s, 4H), 7.79 (d, *J* = 8.4 Hz, 2H), 7.73 (s, 2H), 7.66 (tt, *J* = 7.9, 1.6 Hz, 2H), 7.40 (d, *J* = 7.1 Hz, 2H), 4.59 (d, *J* = 15.1 Hz, 2H), 3.75 (d, *J* = 15.1 Hz, 2H), 3.20 – 3.12 (m, 2H), 2.99 – 2.94 (m, 2H), 2.38 – 2.27 (m, 2H), 2.01 – 1.90 (m, 2H), 1.88 – 1.71 (m, 6H). **^{13}C{^{1}H} NMR (101 MHz, $CDCl_3$)** δ 155.5 – 154.3 (m), 149.2, 141.1, 137.4, 137.0, 132.1 (q, *J* = 36.5 Hz), 130.4 – 129.9 (m), 129.6, 127.9, 127.8, 124.8, 123.4 (q, *J* = 273.0 Hz), 121.9 (q, *J* = 2.8 Hz), 118.4, 67.2, 61.8, 55.6, 27.2, 24.0. **^{19}F NMR (376 MHz, $CDCl_3$)** δ -62.8. **HRMS (ESI) m/z:** $[M+H]^+$ calcd for $C_{44}H_{35}F_{12}N_4$ 847.2665, found 847.2661.

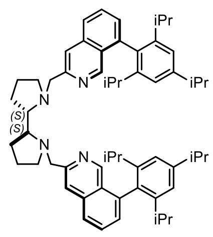

$^{2,4,6\text{-(tri-iPr)}}iQ_2$dp

(*S*,*S*)-**$^{2,4,6\text{-(tri-iPr)}}iQ_2$dp Ligand:** Compound **12f** (437 mg) and reacted according to the General Procedure for the Preparation of **^{Ar}iQ$_2$dp** ligands. The product was purified by column chromatography (MeOH/$CHCl_3$ 2:98 to TEA/MeOH/$CHCl_3$ 1:2:97), affording ligand (*S*,*S*)-**$^{2,4,6\text{-(tri-iPr)}}iQ_2$dp** (318 mg, 77% yield) as a light yellow solid.

Characterization data of compound (*S*,*S*)-**$^{2,4,6\text{-(tri-iPr)}}iQ_2$dp**: $[\alpha]_D^{23} = -24$ (c = 0.05 , DCM); **m.p.** 123-126°C. **^{1}H NMR (500 MHz, $CDCl_3$)** δ 8.72 (s, 2H), 7.78 – 7.73 (m, 4H), 7.66 (t, *J* = 8.2 Hz, 2H), 7.33 (d, *J* = 7.1 Hz, 2H), 7.12 (d, *J* = 1.5 Hz, 4H), 4.33 (d, *J* = 14.5 Hz, 2H), 3.65 (d, *J* = 14.4 Hz, 2H), 3.16 (ddd, *J* = 6.6, 6.5, 3.1 Hz, 2H), 2.98 (hept, *J* = 7.0 Hz, 2H), 2.92 – 2.84 (m, 2H), 2.35 – 2.21 (m, 6H), 1.93 – 1.82 (m, 4H), 1.81 – 1.69 (m, 4H), 1.33 (d, *J* = 6.9 Hz, 12H), 1.04 (d, *J* = 6.9 Hz, 6H), 1.03 (d, *J* = 6.9 Hz, 6H), 0.96 (d, *J* = 6.9 Hz, 6H), 0.94 (d, *J* = 6.9 Hz, 6H). **^{13}C{^{1}H} NMR (126 MHz, $CDCl_3$)** δ 153.7, 151.0, 149.0, 147.3, 139.7, 136.7, 132.6, 129.8, 128.2, 127.4, 125.7, 120.9, 118.7, 65.6, 61.0, 55.6, 34.5, 30.8, 26.0, 25.0, 25.0, 24.3, 24.0, 24.0, 23.7. **HRMS (ESI) m/z:** $[M+H]^+$ calcd for $C_{58}H_{75}N_4$ 827.5986, found 827.5982.

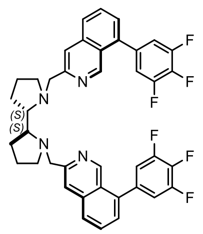

$^{3,4,5\text{-(tri-F)}}$iQ$_2$dp

(*S*,*S*)-**$^{3,4,5\text{-(tri-F)}}$iQ$_2$dp Ligand:** Compound **12g** (361 mg) and reacted according to ^{Ar}iQ$_2$dp ligands general procedure. The product was purified by column chromatography (MeOH/$CHCl_3$ 2:98 to TEA/MeOH/$CHCl_3$ 1:2:97), affording ligand (*S*,*S*)-**$^{2,4,6\text{-(tri-iPr)}}iQ_2$dp** (260 mg, 76% yield) as a light yellow amorphous solid.

Characterization data of compound (*S*,*S*)-**$^{2,4,6\text{-(tri-iPr)}}iQ_2$dp:** $[\alpha]_D^{23} = -21$ (c = 0.02 , DCM); **^{1}H NMR (400 MHz, $CDCl_3$)** δ 9.05 (s, 2H), 7.77 (d, *J* = 8.4 Hz, 2H), 7.72 (s, 2H), 7.64 (dd, *J* = 8.4, 7.0 Hz, 2H), 7.36 (dd, *J* = 7.1, 1.2 Hz, 2H), 7.03 (dd, *J* = 8.0, 6.6 Hz, 4H), 4.55 (d, *J* = 15.0 Hz, 2H), 3.73 (d, *J* = 15.0 Hz, 2H), 3.19 – 3.10 (m, 2H), 3.00 – 2.90 (m, 2H), 2.37 – 2.26 (m, 2H), 2.03 – 1.92 (m, 2H), 1.88 – 1.70 (m, 6H). **^{13}C{^{1}H} NMR (101 MHz, $CDCl_3$)** δ 154.5, 151.2 (ddd, *J* = 250.7, 9.4, 3.5 Hz), 149.5, 139.7 (dt, *J* = 253.8, 15.4 Hz), 137.6, 137.0, 135.2 – 134.5 (m), 129.6, 127.5, 127.4, 124.9, 118.3, 114.3 (dd, *J* = 14.9, 6.0 Hz), 66.8, 61.6, 55.6, 26.9, 23.9. **^{19}F NMR (376 MHz, $CDCl_3$)** δ -134.0 (d, *J* = 20.5 Hz), -161.2 (t, *J* = 20.5 Hz). **HRMS (ESI) m/z:** $[M+H]^+$ calcd for $C_{40}H_{33}F_6N_4$ 683.2604, found 683.2603.

3.1. Complex Synthesis

3.3.4. General procedure for the Preparation of Iron Triflate Complexes: A solution of $Fe(OTf)_2(CH_3CN)_2$ (1 equiv.) (prepared according to a procedure reported by Hagen)[10] in THF (0.2 M) was added to a stirred solution of the ^{Ar}**iQ$_2$dp** or ^{Ar}**iQ$_2$mc** ligand in diethyl ether (0.1 M) under an argon atmosphere. The mixture was stirred at room temperature for 2 hours. The resulting insoluble solid was filtered, dried under vacuum, yielding an air-stable powder of the iron complex.

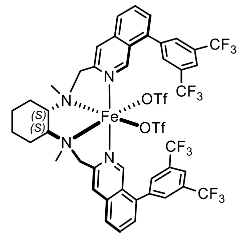

Fe$^{(3,5\text{-(di-CF3)}}$iQ$_2$mc)

(*S*,*S*)-**Fe($^{3,5\text{-(di-CF3)}}$iQ$_2$mc):** Ligand (*S*,*S*)-$^{3,5\text{-(di-CF3)}}$**iQ$_2$mc** (0.024 mmol, 20 mg) was reacted according to the General procedure for the Preparation of Iron Triflate Complexes, affording (*S*,*S*)-**Fe($^{3,5\text{-(di-CF3)}}$iQ$_2$mc)** (25 mg, 86% yield) as a dark orange air stable solid.

Characterization data of (*S*,*S*)-**Fe($^{3,5\text{-(di-CF3)}}$iQ$_2$mc): ^{1}H NMR (500 MHz, CD_2Cl_2)** δ 198.0 (s), 184.0 (s), 162.1 (s), 130.4 (s), 83.1 (s), 46.9 (s), 40.1 (s), 30.0 (s), 23.6 (s), 19.1 – -3.4 (m), -6.0 (s), -22.8 (s). **HRMS (ESI) m/z:** [M-OTf]$^+$ calcd for $C_{45}H_{36}F_{15}FeN_4O_3S$ 1053.1613, found 1053.1617.

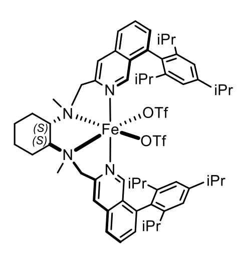

Fe$^{(2,4,6\text{-(tri-iPr)}}iQ_2$mc)

(*S*,*S*)-**Fe($^{2,4,6\text{-(tri-iPr)}}iQ_2$mc):** Ligand (*S*,*S*)-$^{2,4,6\text{-(tri-iPr)}}$**iQ$_2$mc** (0.017 mmol, 14 mg) was reacted according to the General procedure for the Preparation of Iron Triflate Complexes, affording compound (*S*,*S*)-**Fe($^{2,4,6\text{-(tri-iPr)}}iQ_2$mc)** (10.5 mg, 52% yield) as an orange air stable solid.
Characterization data of (*S*,*S*)-**Fe($^{2,4,6\text{-(tri-iPr)}}iQ_2$mc): ^{1}H NMR (500 MHz, CD_2Cl_2)** δ 183.9 (s), 171.4 (s), 156.3 (s), 129.0 (s), 98.5 (s), 79.7 (s), 71.7 (s), 63.3 (s), 49.3 (s), 44.4 (s), 26.9 – -9.3 (m). **HRMS (ESI) m/z:** [M-OTf]$^+$ calcd for $C_{59}H_{76}F_3FeN_4O_3S$ 1033.4934, found 1033.4926.

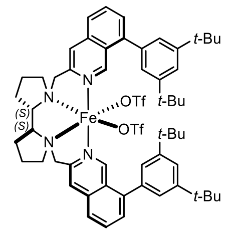

Fe$^{(3,5\text{-(di-t-Bu)}}$iQ^{2}dp)

(*S*,*S*)-**Fe($^{3,5\text{-(di-}t\text{-Bu)}}iQ_2$dp):** Ligand (*S*,*S*)-$^{3,5\text{-(di-}t\text{-Bu)}}$**iQ$_2$dp** (0.1 mmol, 80 mg) was reacted according to the General procedure for the Preparation of Iron Triflate Complexes, affording (*S*,*S*)-**Fe($^{3,5\text{-(di-}t\text{-Bu)}}iQ_2$dp)** (105 mg, 91% yield) as a dark yellow air stable solid.

Characterization data of (*S*,*S*)-**Fe($^{3,5\text{-(di-}t\text{-Bu)}}iQ_2$dp): ^{1}H NMR (500 MHz, CD_2Cl_2)** δ 189.8 (s), 140.5 (s), 95.6 (s), 44.9 (s), 39.8 (s), 32.2 (s), 13.1 (s), 10.7 – -2.7 (m), -6.5 (s), -16.1 (s). **HRMS (ESI) m/z:** [M-OTf]$^+$ calcd for $C_{57}H_{70}F_3FeN_4O_3S$ 1003.4465, found 1003.4467.

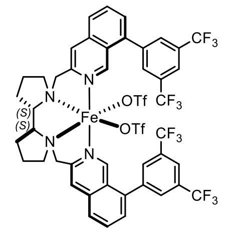

Fe$^{(3,5\text{-(di-CF3)}}$iQ$_2$dp)

(*S*,*S*)-**Fe($^{3,5\text{-(di-CF3)}}$iQ$_2$dp):** Ligand (*S*,*S*)-$^{3,5\text{-(di-CF3)}}$**iQ$_2$dp** (0.106 mmol, 90 mg) was reacted according to the General procedure for the Preparation of Iron Triflate Complexes, affording (*S*,*S*)-**Fe($^{3,5\text{-(di-CF3)}}$iQ$_2$dp)** (95 mg, 75% yield) as an orange air stable solid.

Characterization data of (*S*,*S*)-**Fe($^{3,5\text{-(di-CF3)}}$iQ$_2$dp)**: **^{1}H NMR (400 MHz, CD_2Cl_2)** δ 192.1 (s), 84.7 (s), 47.7 (s), 37.0 (s), 29.9 (s), 20.9 – -1.8 (m), -7.8 (s), -20.0 (s). **HRMS (ESI) m/z:** [M-OTf]$^+$ calcd for $C_{45}H_{34}F_{15}FeN_4O3S$ 1051.1456, found 1051.1458.

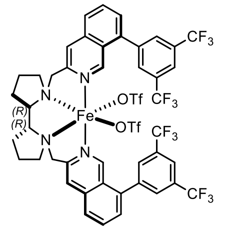

ent-Fe$^{(3,5\text{-(di-CF3)}}$iQ$_2$dp)

***ent*-Fe($^{3,5\text{-(di-CF3)}}$iQ$_2$dp):** Ligand (*R*,*R*)-$^{3,5\text{-(di-CF3)}}$**iQ$_2$dp** (0.078 mmol, 66 mg) reacted according to the General procedure for the Preparation of Iron Triflate Complexes, affording *ent*-**Fe($^{3,5\text{-(di-CF3)}}$iQ$_2$dp)** (73 mg, 78% yield) as an orange air stable solid.

Characterization data of ***ent*-Fe($^{3,5\text{-(di-CF3)}}$iQ$_2$dp)**: **^{1}H NMR (500 MHz, CD_2Cl_2)** δ 191.1 (s), 84.2 (s), 48.2 (s), 35.1 (s), 28.8 (s), 16.9 – 0.7 (m), -8.8 (s), -20.1 (s). **HRMS (ESI) m/z:** [M-OTf]$^+$ calcd for $C_{45}H_{34}F_{15}FeN_4O3S$ 1051.1456, found 1051.1451.

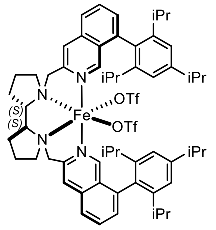

Fe$^{(2,4,6\text{-(tri-iPr)}}iQ_2$dp)

(*S*,*S*)-**Fe($^{2,4,6\text{-(tri-iPr)}}iQ_2$dp):** Ligand (*S*,*S*)-$^{2,4,6\text{-(tri-iPr)}}$**iQ$_2$dp** (0.1 mmol, 83 mg) reacted according to Iron triflate complexation general procedure, affording compound (*S*,*S*)-**Fe($^{2,4,6\text{-(tri-iPr)}}iQ_2$dp)** (76 mg, 64% yield) as a brown air stable solid.

Characterization data of (*S*,*S*)-**Fe($^{2,4,6\text{-(tri-iPr)}}iQ_2$dp):^{1}H NMR (400 MHz, $CDCl_3$)** δ 193.6 (s), 81.7 (s), 46.4 (s), 28.7 (s), 15.8 – -3.1 (m), -6.0 (s), -23.4 (s). **HRMS (ESI) m/z:** [M-OTf]$^+$ calcd for $C_{59}H_{74}F_3FeN_4O_3S$ 1031.4778, found 1031.4779.

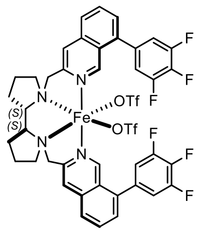

Fe$^{(3,4,5\text{-(tri-F)}}$iQ$_2$dp)

(*S*,*S*)-**Fe($^{3,4,5\text{-(tri-F)}}$iQ$_2$dp):** Ligand (*S*,*S*)-$^{3,4,5\text{-(tri-F)}}$**iQ$_2$dp** (0.16 mmol, 110 mg) reacted according to Iron triflate complexation general procedure, affording compound (*S*,*S*)-**Fe($^{3,4,5\text{-(tri-F)}}$iQ$_2$dp)** (150 mg, 90% yield) as a yellow-orange air stable solid.

Characterization data of (*S*,*S*)-**Fe($^{3,4,5\text{-(tri-F)}}$iQ$_2$dp): ^{1}H NMR (500 MHz, CD_2Cl_2)** δ 193.9 (s), 81.1 (s), 49.0 (s), 33.7 (s), 27.6 (s), 20.3 – -5.0 (m), -9.4 (s), -21.5 (s). **HRMS (ESI) m/z:** [M-OTf]$^+$ calcd for $C_{41}H_{32}F_9FeN_4O_3S$ 887.1395, found 887.1392.

4. Catalytic studies

4.1. Table S1. epoxidation of methyl cinnamate with different acids

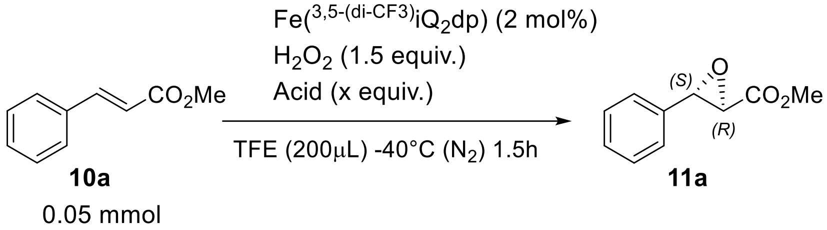

entry	acid (X equiv.)	yield [%][a]	ee [%][b]
1	2-EHA (0.05)	20	73
2	Benzoic acid (0.05)	81	93
3	Benzoic acid (0.5)	99.5	95
4	Benzoic acid (1.5)	97	94.5
5	Acetic acid (0.5)	35	74
6	2-EHA (0.5)	60	90
7	Pivalic acid (0.5)	90	94
8	(*S*)-Ibuprofen	80	97
9	4-Chlorobenzoic acid	20	84
10	4-Methylbenzoic acid	92	94
11[c]	Benzoic acid (0.5)	40	91.5

[a]HPLC yield using chlorobenzene as internal standard; [b]enantiomeric excess determined with HPLC on chiral cellulose column; [c]H_2O_2 (30% in H_2O) added in TFE instead of acetonitrile. Abbreviations: TFE = 2,2,2-trifluoroethanol, 2-EHA = 2-ethylhexanoic acid.

Reaction conditions:

Methyl cinnamate **10a** (1 equiv., 0.05 mmol, 8 mg), Fe(**$^{3,5\text{-di-CF3}}$iQ$_2$dp**)(OTf)$_2$ (2 mol %, 5 mg), acid (X equiv.) and chlorobenzene (1 equiv., 5 µL) were dissolved in TFE (0.25 M, 200 µL) under N_2 atmosphere. The solution was cooled to -40ºC and a solution of H_2O_2 (1.5 equiv., 7.5 µL, 30% in water) in acetonitrile (0.75 M, 100 µL) or TFE was added dropwise by syringe pump over a period of 30 min. The mixture was stirred for an additional 1h at -40ºC. Then 5 µL from the mixture was diluted in 1 mL acetonitrile and injected into HPLC to determine the yield. The remaining mixture was passed through a silica plunge, the solvent was removed under reduced pressure and the remaining residue was sampled for chiral normal phase HPLC analysis to determine the *ee* values.

4.2. Table S2. epoxidation of alkyl cinnamates using iron ^{Ar}**iQ2dp** and iron ^{Ar}**iQ2mc** complexes

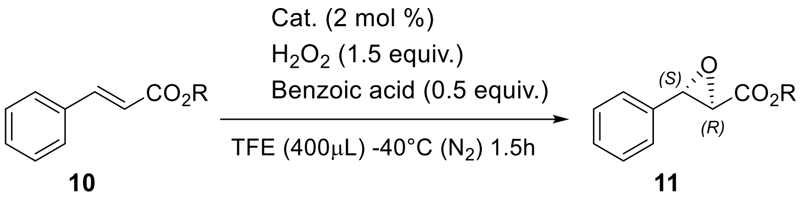

entry	R	Catalyst	Yield (%)[a]	ee (%)[c]
1	Me	Fe(**iQ2dp**)	37	57
2	Me	Fe($^{\mathbf{3,5\text{-}(di\text{-}CF3)}}$**iQ2dp**)	**99 (98[b])**	**95**
3	Me	*ent*-Fe($^{\mathbf{3,5\text{-}(di\text{-}CF3)}}$**iQ2dp**)	100	-94.5
4	Me	Fe($^{\mathbf{2,4,6\text{-}(tri\text{-}iPr)}}$**iQ2dp**)	--	--
5	Me	Fe($^{\mathbf{3,5\text{-}(di\text{-}\mathit{t}\text{-}Bu)}}$**iQ2dp**)	85	69
6	Me	Fe($^{\mathbf{3,4,5\text{-}(tri\text{-}F)}}$**iQ2dp**)	50	60
7	Me	Fe($^{\mathbf{2,4,6\text{-}(tri\text{-}iPr)}}$**iQ2mc**)	32	55
8	Me	Fe($^{\mathbf{2,4,6\text{-}(tri\text{-}iPr)}}$**iQ2mc**)	--	--
9	Et	Fe($^{\mathbf{3,5\text{-}(di\text{-}CF3)}}$**iQ2dp**)	93[b]	92.5
10	iPr	Fe($^{\mathbf{3,5\text{-}(di\text{-}CF3)}}$**iQ2dp**)	90[b]	86.5

[a]HPLC yield using chlorobenzene as internal standard; [b]Isolated yield; [c]enantiomeric excess, determined with HPLC on chiral cellulose column.

Reaction conditions:

Methyl cinnamate **10a** (1 equiv., 0.1 mmol, 16 mg), iron complex (2 mol %), benzoic acid (0.5 equiv., 6 mg) and chlorobenzene (1 equiv., 10 µL) were dissolved in TFE (0.25 M, 400 µL) the solution was cooled to -40ºC and put under N_2 atmosphere. To this solution, H_2O_2 (1.5 equiv., 15 µL, 30% in water) in acetonitrile (0.75 M, 200 µL) was added dropwise by syringe pump over a period of 30 min. The mixture was stirred for an additional 1h at -40ºC, then 5 µL from the mixture were diluted in 1 mL acetonitrile and injected to reverse phase HPLC to determine yield. The remaining mixture passed through a silica plug, the solvent was removed under reduced pressure and the residue sampled for chiral normal phase HPLC analysis to determine the *ee* values.

4.3. General Procedure for enantioselective epoxidation

A solution of olefin **10** (1 equiv., 0.2 mmol), Fe($^{3,5\text{-di-CF3}}$**iQ2dp**) (2 mol %, 5 mg) and benzoic acid (0.5 equiv., 12 mg) in TFE (0.25 M, 800 µL) was cooled to -40ºC, under N_2 atmosphere. To this solution, H_2O_2 (1.5 equiv., 30 µL, 30% in water) in acetonitrile (0.75 M, 400 µL) was added dropwise by syringe pump over a period of 30 min. The mixture was stirred for an additional 1h at -40ºC before it was diluted with aq. $NaHCO_3$ and extracted with dichloromethane. The combined organic phase washed with brine, dried with Na_2SO_4 and passed through a short silica-gel plug. The volatiles were removed under reduced pressure to afford the epoxide product. If needed the residue was further purified by column chromatography.

4.4. General Procedure for selective hydroxy carboxylation

A solution of methyl (E)-3-(4-methoxyphenyl)acrylate **10i** (1 equiv., 0.2 mmol, 38 mg), Fe($^{3,5\text{-di-CF3}}$**iQ2dp**) (2 mol %, 5 mg) and substituted acid (1.5 equiv., 0.3 mmol) in TFE (0.25 M, 800 µL) was cooled to -40ºC, under N_2 atmosphere. To this solution, H_2O_2 (1.5 equiv., 30 µL, 30% in water) in acetonitrile (0.75 M, 400 µL) was added dropwise by syringe pump over a period of 30 min. The mixture was stirred for an additional 1h at -40ºC. The volatiles were removed under reduced pressure, and the residue was purified by column chromatography to afford the affording mono-protected diols product.

4.5. Mechanistic Probing of Selective Hydroxy Carboxylation via Epoxide Testing

To better understand the hydroxy carboxylation of methyl (E)-3-(4-methoxyphenyl)acrylate **10i**. methyl 3-(4-methoxyphenyl)oxirane-2-carboxylate **11i** was synthesized according to established literature conditions.[11] The resulting epoxide was then subjected to the general procedure for selective hydroxy carboxylation using benzoic acid. However, no reaction was observed, and the epoxide **11i** remained intact. This result confirms that the formation of products **12i** does not proceed via initial epoxidation followed by acid-mediated epoxide ring opening. Instead, it suggests that the hydroxyl and carboxylate functionalities are directly transferred to the olefin from a single metal active site.

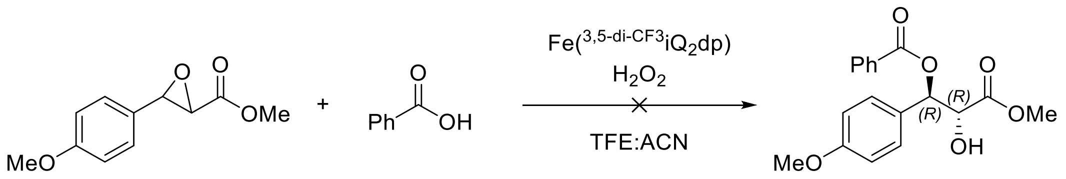

11i **12ia**

4.6. Characterization data

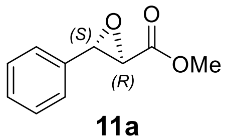
11a

Compound 11a: Methyl cinnamate **10a** (32 mg) reacted according to general procedure for enantioselective epoxidation. The crude product was purified by column chromatography (ethyl acetate/hexane 5:95) affording compound **11a** (35 mg, 98% yield, 95% *ee*) as a colorless oil.

Characterization data of compound **11a**: **^{1}H NMR (500 MHz, $CDCl_3$)** δ 7.40 – 7.34 (m, 3H), 7.32 – 7.26 (m, 2H), 4.10 (d, *J* = 1.8 Hz, 1H), 3.83 (s, 3H), 3.52 (d, *J* = 1.7 Hz, 1H). **$^{13}C\{^{1}H\}$ NMR (126 MHz, $CDCl_3$)** δ 168.8, 135.0, 129.2, 128.8, 126.0, 58.1, 56.8, 52.8. The spectroscopic data for this product match the literature data.[6]
Chiral stationary-phase HPLC analysis [Lux Cellulose-1, hexane/iPrOH 80:20, flow rate 1.0 mL/min, tR 7.8 min (*2R,3S*) and 10.1 min (*2S,3R*), detection at 230 nm];

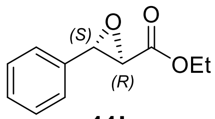
11b

Compound 11b: Ethyl cinnamate **10b** (36 mg) reacted according to general procedure for enantioselective epoxidation. The crude product was purified by column chromatography (ethyl acetate/hexane 5:95) affording compound **11b** (36 mg, 93% yield, 92.5% *ee*) as a colorless oil.

Characterization data of compound **11b**: **^{1}H NMR (500 MHz, $CDCl_3$)** δ 7.39 – 7.33 (m, 3H), 7.29 (dd, *J* = 7.5, 2.1 Hz, 2H), 4.36 – 4.22 (m, 2H), 4.09 (d, *J* = 1.9 Hz, 1H), 3.50 (d, *J* = 1.7 Hz, 1H), 1.33 (t, *J* = 7.2 Hz, 3H). **$^{13}C\{^{1}H\}$ NMR (126 MHz, $CDCl_3$)** δ 168.3, 135.1, 129.1, 128.7, 125.9, 61.9, 58.0, 56.9, 14.2. The spectroscopic data for this product match the literature data.[6]
Chiral stationary-phase HPLC analysis [Lux Cellulose-1, hexane/iPrOH 99:1, flow rate 1.0 mL/min, tR 15.1 min (*2R,3S*) and 16.5 min (*2S,3R*), detection at 230 nm];

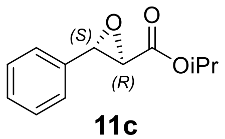
11c

Compound 11c: Isopropyl cinnamate **10c** (38 mg) reacted according to general procedure for enantioselective epoxidation. The crude product was purified by column chromatography (ethyl acetate/hexane 5:95) affording compound **11c** (37 mg, 90% yield, 86.5% *ee*) as a colorless oil.

Characterization data of compound **11c**: **^{1}H NMR (500 MHz, $CDCl_3$)** δ 7.41 – 7.33 (m, 3H), 7.29 (dd, *J* = 7.4, 2.2 Hz, 2H), 5.15 (hept, *J* = 6.2 Hz, 1H), 4.07 (d, *J* = 1.8 Hz, 1H), 3.48 (d, *J* = 1.8 Hz, 1H), 1.32 (d, *J* = 6.2 Hz, 3H), 1.30 (d, *J* = 6.3 Hz, 3H). **$^{13}C\{^{1}H\}$ NMR (126 MHz, $CDCl_3$)** δ 167.9, 135.2, 129.1, 128.8, 126.0, 69.8, 58.0, 57.1, 21.9, 21.9. The spectroscopic data for this product match the literature data.[6]
Chiral stationary-phase HPLC analysis [Lux Cellulose-1, hexane/iPrOH 80:20, flow rate 1.0 mL/min, tR 4.7 min (*2R,3S*) and 4.3 min (*2S,3R*), detection at 230 nm];

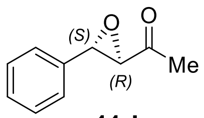
11d

Compound 11d: (E)-4-phenylbut-3-en-2-one **10d** (29 mg) reacted according to general procedure for enantioselective epoxidation. The crude product was purified by column chromatography (ethyl acetate/hexane 2:98) affording compound **11d** (19 mg, 58% yield, 75% *ee*) as a colorless oil.

Characterization data of compound **11d**: **^{1}H NMR (400 MHz, $CDCl_3$)** δ 7.39 – 7.34 (m, 2H), 7.30 – 7.26 (m, 2H), 4.01 (d, *J* = 1.9 Hz, 1H), 3.49 (d, *J* = 1.9 Hz, 1H), 2.20 (s, 3H). **$^{13}C\{^{1}H\}$ NMR (126 MHz, $CDCl_3$)** δ 204.3, 135.2, 129.2, 128.9, 125.8, 63.6, 57.9, 24.9. The spectroscopic data for this product match the literature data.[6]
Chiral stationary-phase HPLC analysis [Lux Cellulose-1, hexane/iPrOH 80:20, flow rate 1.0 mL/min, tR 6.8 min (*2R,3S*) and 6.3 min (*2S,3R*), detection at 220 nm];

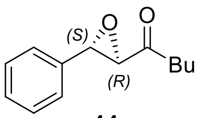

11e

Compound 11e: (E)-1-phenylhept-1-en-3-one **10e** (38 mg) reacted according to general procedure for enantioselective epoxidation. The crude product was purified by column chromatography (ethyl acetate/hexane 2:98) affording compound **11e** (24 mg, 59% yield, 81% *ee*) as a colorless oil.

Characterization data of compound **11e**: **^{1}H NMR (400 MHz, CDCl$_3$)** δ 7.43 – 7.22 (m, 4H), 3.97 (d, *J* = 1.9 Hz, 1H), 3.52 (d, *J* = 1.9 Hz, 1H), 2.62 – 2.37 (m, 2H), 1.70 – 1.54 (m, 2H), 1.36 (h, *J* = 7.4 Hz, 2H), 0.93 (t, *J* = 7.4 Hz, 3H). **^{13}C{^{1}H} NMR (126 MHz, CDCl$_3$)** δ 206.3, 135.4, 129.1, 128.8, 125.8, 63.3, 58.2, 37.7, 25.4, 22.4, 14.0. The spectroscopic data for this product match the literature data.[12]

Chiral stationary-phase HPLC analysis [Lux Cellulose-1, hexane/iPrOH 99.1:0.9, flow rate 1.0 mL/min, tR 11.3 min (*2R,3S*) and 11.9 min (*2S,3R*), detection at 230 nm];

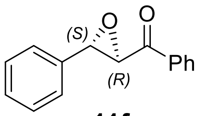

11f

Compound 11f: (E)-chalcone **10f** (42 mg) reacted according to general procedure for enantioselective epoxidation. The crude product was purified by column chromatography (ethyl acetate/hexane 5:95) affording compound **11f** (18 mg, 40% yield, 4% *ee*) as a colorless oil.

Characterization data of compound **11f**: **^{1}H NMR (500 MHz, CDCl$_3$)** δ 8.04 – 7.98 (m, 2H), 7.62 (tt, *J* = 7.2, 1.0 Hz, 1H), 7.53 – 7.46 (m, 2H), 7.44 – 7.35 (m, 5H), 4.30 (d, *J* = 1.8 Hz, 1H), 4.08 (d, *J* = 1.8 Hz, 1H). **^{13}C{^{1}H} NMR (126 MHz, CDCl$_3$)** δ 193.2, 135.6, 134.1, 129.2, 129.0, 129.0, 128.9, 128.5, 125.9, 61.1, 59.5. The spectroscopic data for this product match the literature data.[6]

Chiral stationary-phase HPLC analysis [Lux Cellulose-1, hexane/iPrOH 80:20, flow rate 1.0 mL/min, tR 7.6 min (*2R,3S*) and 6.8 min (*2S,3R*), detection at 240 nm];

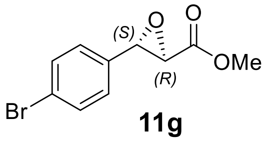

11g

Compound 11g: methyl (E)-3-(4-bromophenyl)acrylate **10g** (48 mg) reacted according to general procedure for enantioselective epoxidation. The crude product was purified by column chromatography (ethyl acetate/hexane 5:95) affording compound **11g** (49 mg, 95% yield, 93% *ee*) as a colorless oil.

Characterization data of compound **11g**: **^{1}H NMR (400 MHz, CDCl$_3$)** δ 7.53 – 7.45 (m, 2H), 7.21 – 7.12 (m, 2H), 4.06 (d, *J* = 1.7 Hz, 1H), 3.83 (s, 3H), 3.46 (d, *J* = 1.7 Hz, 1H). **^{13}C{^{1}H} NMR (126 MHz, CDCl$_3$)** δ 168.4, 134.1, 132.0, 127.5, 123.2, 57.5, 56.7, 52.8. The spectroscopic data for this product match the literature data.[13]

Chiral stationary-phase HPLC analysis [Lux Cellulose-1, hexane/iPrOH 80:20, flow rate 1.0 mL/min, tR 6.3 min (*2R,3S*) and 7.3 min (*2S,3R*), detection at 230 nm];

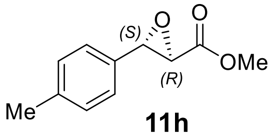

11h

Compound 11h: methyl (E)-3-(p-tolyl)acrylate **10h** (35 mg) reacted according to general procedure for enantioselective epoxidation. The crude product was purified by column chromatography (ethyl acetate/hexane 2:98) affording compound **11h** (18 mg, 47% yield, 76% *ee*) as a colorless oil.

Characterization data of compound **11h**: **^{1}H NMR (400 MHz, CDCl$_3$)** δ 7.18 (s, 4H), 4.06 (d, *J* = 1.8 Hz, 1H), 3.83 (s, 3H), 3.51 (d, *J* = 1.8 Hz, 1H), 2.35 (s, 3H). **^{13}C{^{1}H} NMR (101 MHz, CDCl$_3$)** δ 168.9, 139.2, 132.0, 129.5, 125.9, 58.2, 56.7, 52.7, 21.4 The spectroscopic data for this product match the literature data.[14]

Chiral stationary-phase HPLC analysis [Lux Cellulose-1, hexane/iPrOH 80:20, flow rate 1.0 mL/min, tR 5.4 min (*2R,3S*) and 5.9 min (*2S,3R*), detection at 230 nm];

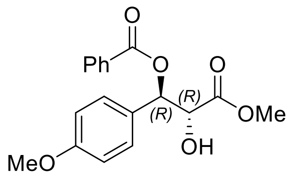

12ia

Compound 12ia: reacted according to general procedure for selective hydroxy carboxylation using benzoic acid (36 mg). The crude product was purified by column chromatography (ethyl acetate/hexane 20:80) affording compound **12ia** (30 mg, 46% yield, 37.5% *ee*) as an amorphous solid.

Characterization data of compound **12ia**: $[\alpha]_D^{24} = -20$ (c = 0.02 , DCM); **^{1}H NMR (400 MHz, $CDCl_3$)** δ 8.06 (d, *J* = 7.2 Hz, 2H), 7.58 (t, *J* = 7.4 Hz, 1H), 7.48 – 7.39 (m, 4H), 6.90 (d, *J* = 8.9 Hz, 2H), 6.25 (d, *J* = 2.9 Hz, 1H), 4.52 (dd, *J* = 6.7, 3.0 Hz, 1H), 3.79 (s, 3H), 3.78 (s, 3H), 3.11 (d, *J* = 6.8 Hz, 1H). **^{13}C{^{1}H} NMR (126 MHz, $CDCl_3$)** δ 172.6, 165.4, 159.9, 133.5, 129.9, 129.8, 128.6, 128.5, 128.4, 114.1, 76.0, 73.8, 55.4, 53.2. **HRMS (ESI) m/z:** $[M+Na]^+$ calcd for $C_{18}H_{18}O_6Na$ 353.0996, found 353.1005. Chiral stationary-phase HPLC analysis [Lux Cellulose-1, hexane/iPrOH 95:5, flow rate 1.0 mL/min, tR 19.3 min (*2R*,*3R*) and 21.3 min (*2S*,*3S*), detection at 220 nm];

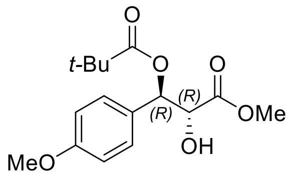

12ib

Compound 12ib: reacted according to general procedure for selective hydroxy carboxylation using pivalic acid (34 µL). The crude product was purified by column chromatography (ethyl acetate/hexane 15:85) affording compound **12ib** (47 mg, 62% yield, 82% *ee*) as an amorphous solid.

Characterization data of compound **12ib**: $[\alpha]_D^{24} = +19$ (c = 0.05 , DCM); **^{1}H NMR (400 MHz, $CDCl_3$)** δ 7.36 – 7.28 (m, 2H), 6.93 – 6.85 (m, 2H), 6.00 (d, *J* = 2.7 Hz, 1H), 4.38 (dd, *J* = 6.2, 2.5 Hz, 1H), 3.80 (s, 3H), 3.79 (s, 3H), 2.93 (d, *J* = 7.0 Hz, 1H), 1.21 (s, 9H). **^{13}C{^{1}H} NMR (126 MHz, $CDCl_3$)** δ 177.1, 172.6, 159.8, 128.7, 128.2, 114.0, 75.0, 73.7, 55.4, 53.0, 27.2, 27.2. **HRMS (ESI) m/z:** $[M+Na]^+$ calcd for $C_{16}H_{22}O_6Na$ 333.1309, found 333.1314.

Chiral stationary-phase HPLC analysis [Lux Cellulose-1, hexane/iPrOH 95:5, flow rate 1.0 mL/min, tR 9.5 min (*2R*,*3S*) and 10.2 min (*2S*,*3R*), detection at 230 nm];

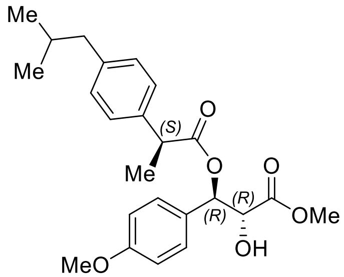

12ic

Compound 12ic: reacted according to general procedure for selective hydroxy carboxylation using (*S*)-ibuprofen (62 mg). The crude product was purified by column chromatography (ethyl acetate/hexane 17:83) affording mixture of diastereomer **12ic** and diastereomer **12ic'** (38 mg, 46% yield, 81% *de*) as an amorphous solid.

Characterization data of diastereomer **12ic** (isolated by carful secondary column chromatography): $[\alpha]_D^{25} = +37$ (c = 0.02 , DCM); **^{1}H NMR (500 MHz, $CDCl_3$)** δ 7.26 (d, *J* = 8.7 Hz, 2H), 7.21 (d, *J* = 8.1 Hz, 2H), 7.11 (d, *J* = 8.1 Hz, 2H), 6.86 (d, *J* = 8.8 Hz, 2H), 5.96 (d, *J* = 2.7 Hz, 1H), 4.30 (dd, *J* = 7.0, 2.7 Hz, 1H), 3.79 (s, 3H), 3.74 (q, *J* = 7.3 Hz, 1H), 3.49 (s, 3H), 2.83 (d, *J* = 7.1 Hz, 1H), 2.45 (d, *J* = 7.2 Hz, 2H), 1.84 (dh, *J* = 13.4, 6.7 Hz, 1H), 1.47 (d, *J* = 7.2 Hz, 3H), 0.90 (dd, *J* = 6.6, 1.3 Hz, 6H). **^{13}C{^{1}H} NMR (126 MHz, $CDCl_3$)** δ 173.5, 172.1, 159.7, 140.9, 137.4, 129.5, 128.5, 128.3, 127.4, 114.0, 75.5, 73.5, 55.4, 52.7, 45.2, 45.1, 30.4, 22.5, 18.1. **HRMS (ESI) m/z:** $[M+Na]^+$ calcd for $C_{24}H_{30}O_6Na$ 437.1935, found 437.1944.

Chiral stationary-phase HPLC analysis [Lux Cellulose-1, hexane/iPrOH 95:5, flow rate 1.0 mL/min, tR 12.0 min (*2R*,*3R*) and 14.0 min (*2S*,*3S*), detection at 230 nm];

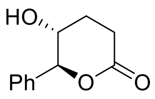
12j

Compound 12j: (E)-5-phenylpent-4-enoic acid **10j** (35 mg, 0.2 mmol), (*S*,*S*)-**Fe($^{3,4,5\text{-(tri-F)}}$iQ$_2$dp)** (2 mol %, 4 mg) were dissolved in acetonitrile (0.2 M, 1 mL). To this solution, H_2O_2 (1.5 equiv., 30 µL, 30% in water) in acetonitrile (0.3 M, 1 mL) was added dropwise by syringe pump over a period of 30 min in room temperature, the mixture was stirred for an additional 1h. The volatiles were removed under reduced pressure, and the residue was purified by column chromatography (ethyl acetate/hexane 40:60) affording (5*R*,6*S*)-5-hydroxy-6-phenyltetrahydro-2H-pyran-2-one **12j** (36 mg, 95% yield, 50% *ee.*) as an amorphous solid.

Characterization data of compound **12j:** $[\alpha]_D^{23} = +32$ (c = 0.02 , DCM);**^{1}H NMR (400 MHz, CDCl$_3$)** δ 7.46 – 7.27 (m, 5H), 4.70 (d, *J* = 6.4 Hz, 1H), 4.65 (q, *J* = 7.0 Hz, 1H), 2.49 – 2.43 (m, 2H), 2.08 (s, 1H), 2.06 – 2.00 (m, 2H).**^{13}C{^{1}H} NMR (126 MHz, CDCl$_3$)** δ 177.1, 138.5, 128.9, 128.8, 127.1, 83.5, 76.6, 28.6, 24.1. **HRMS (ESI) m/z:** $[M+H]^+$ calcd for $C_{11}H_{13}O_3$ 193.0859, found 193.0863.

Chiral stationary-phase HPLC analysis [Lux Cellulose-1, hexane/iPrOH 80:20, flow rate 1.0 mL/min, tR 14.2 min (*2R,3S*) and 13.3 min (*2S,3R*), detection at 230 nm];

5. Crystallographic Data

Data collection, reduction and analysis for **Fe($^{3,5\text{-(di-CF3)}}$iQ$_2$dp)**, **Fe($^{2,4,6\text{-(tri-iPr)}}iQ_2$dp)** were performed with the CrysAlisPro software package (version 1.171.39.22a, Rigaku OD, 2018). was used for the cell determination and intensity data collection. During data collection, the crystal was kept at a steady T = 100(10) K. Structures were solved by intrinsic phasing method and refined using least squares method for F^2 in anisotropic approximation in SHELXTL and Olex2 software [15],[16]. Atomic coordinates and other structural parameters**,** have been deposited with the Cambridge Crystallographic Data Centre: CCDC 2390794 (**Fe($^{3,5\text{-(di-CF3)}}$iQ$_2$dp)**), 2361551 (**Fe($^{2,4,6\text{-(tri-iPr)}}iQ_2$dp)**).

(*S*,*S*)-**Fe(**$^{\mathbf{3,5\text{-}(di\text{-}CF3)}}$**iQ**$_{\mathbf{2}}$**dp):**

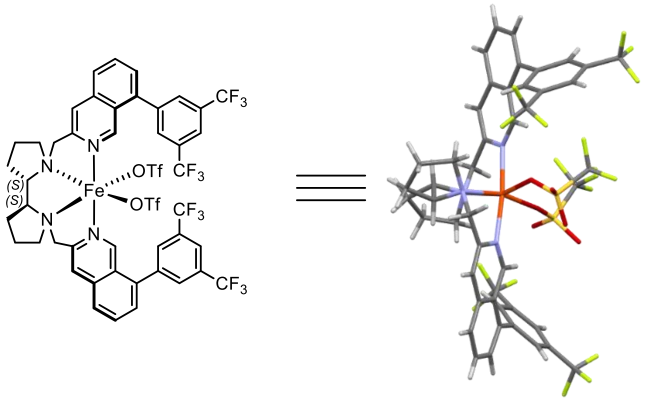

Orange prisms of **Fe(**$^{\mathbf{3,5\text{-}(di\text{-}CF3)}}$**iQ**$_{\mathbf{2}}$**dp)**, suitable for single crystal XRD analysis, were obtained by dissolving 10 mg of the complex in 0.2 mL of DCM in an open 1 mL vial, which was placed inside a closed 20 mL vial containing 1 mL of diethyl ether at room temperature. Crystals formed after 24 hours.

Table S3. Crystal data and structure refinement for **Fe(**$^{\mathbf{3,5\text{-}(di\text{-}CF3)}}$**iQ**$_{\mathbf{2}}$**dp)**.

Empirical formula	$C_{47}H_{36}Cl_2F_{18}FeN_4O_6S_2$
Formula weight	1285.67
Temperature/K	100.00(10)
Crystal system	monoclinic
Space group	$P2_1$
a/Å	8.9842(2)
b/Å	21.1535(3)
c/Å	13.6136(3)
α/°	90
β/°	96.127(2)
γ/°	90
Volume/Å3	2572.45(9)
Z	2
ρcalcg/cm3	1.660
μ/mm-1	5.143
F(000)	1296.0
Crystal size/mm3	0.3 × 0.2 × 0.2
Radiation	CuKα (λ = 1.54184)
2Θ range for data collection/°	6.53 to 136.5
Index ranges	$-9 \le h \le 9$, $-25 \le k \le 25$, $-16 \le l \le 16$
Reflections collected	70699
Independent reflections	8862 [R_{int} = 0.0390, R_{sigma} = 0.0216]
Data/restraints/parameters	8862/13/722
Goodness-of-fit on F2	1.012
Final R indexes [I>=2σ (I)]	R1 = 0.0749, wR2 = 0.1963
Final R indexes [all data]	R1 = 0.0763, wR2 = 0.1976
Largest diff. peak/hole / e Å-3	1.64/-1.03
Flack parameter	0.057(10)

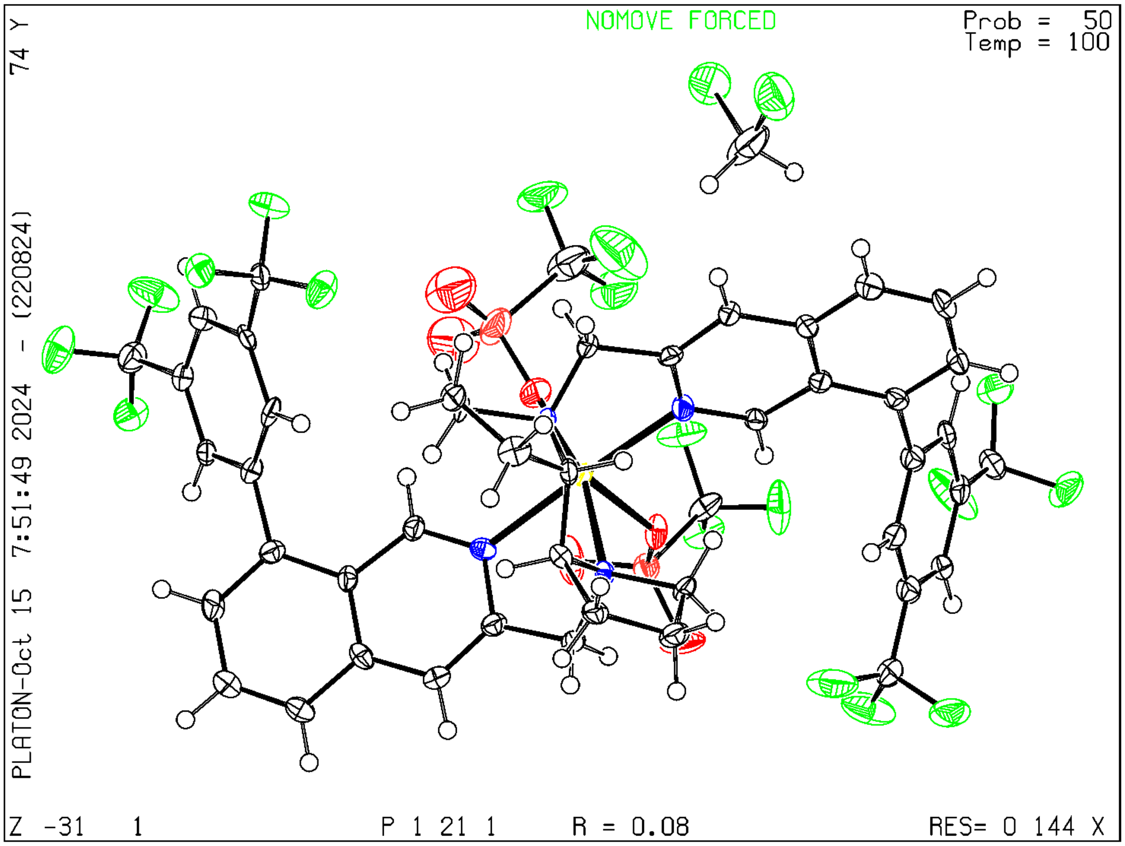

Figure 1. thermal ellipsoid plot of **Fe($^{3,5\text{-(di-CF3)}}$iQ$_2$dp)** showing 50% probability displacement ellipsoids

(*S*,*S*)-**Fe($^{2,4,6\text{-(tri-iPr)}}iQ_2$dp):**

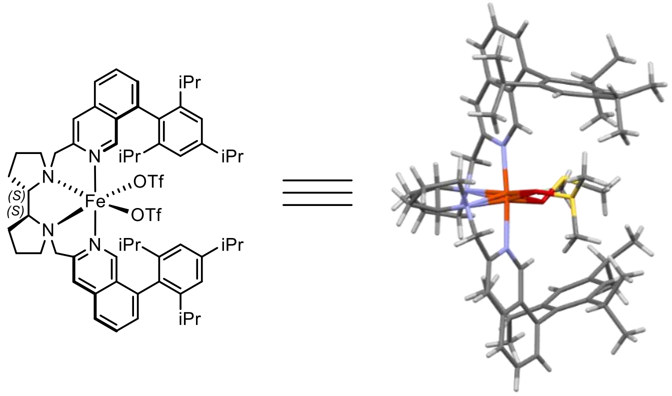

Orange prisms of **Fe($^{2,4,6\text{-(tri-iPr)}}iQ_2$dp)**·2DMSO, suitable for single crystal XRD analysis, were obtained by dissolving 10 mg of the complex **Fe($^{2,4,6\text{-(tri-iPr)}}iQ_2$dp)** in 0.2 mL of DMSO in a 1 mL vial, which was left open to ambient air at room temperature for 72 hours.

Table S4. Crystal data and structure refinement for **Fe($^{2,4,6\text{-(tri-iPr)}}iQ_2$dp)**.

Empirical formula	$C_{64}H_{86}F_6Fe_1N_4OS_4$
Formula weight	1225.5
Temperature/K	100.00(10)
Crystal system	orthorhombic
Space group	$C222_1$
a/Å	15.87570(10)
b/Å	22.7965(2)
c/Å	40.8212(3)
α/°	90
β/°	90
γ/°	90
Volume/Å3	14773.62(19)
Z	8
ρcalcg/cm3	1.243
μ/mm-1	2.934
F(000)	5852.0
Crystal size/mm3	0.29 × 0.22 × 0.18
Radiation	CuKα (λ = 1.54184)
2Θ range for data collection/°	6.784 to 149.83
Index ranges	$-19 \leq h \leq 19$, $-27 \leq k \leq 28$, $-48 \leq l \leq 50$
Reflections collected	72637
Independent reflections	14881 [Rint = 0.0255, Rsigma = 0.0209]
Data/restraints/parameters	14881/0/822
Goodness-of-fit on F2	1.018
Final R indexes [I>=2σ (I)]	R1 = 0.0688, wR2 = 0.1870
Final R indexes [all data]	R1 = 0.0731, wR2 = 0.1914
Largest diff. peak/hole / e Å-3	0.44/-0.40
Flack parameter	0.061(6)

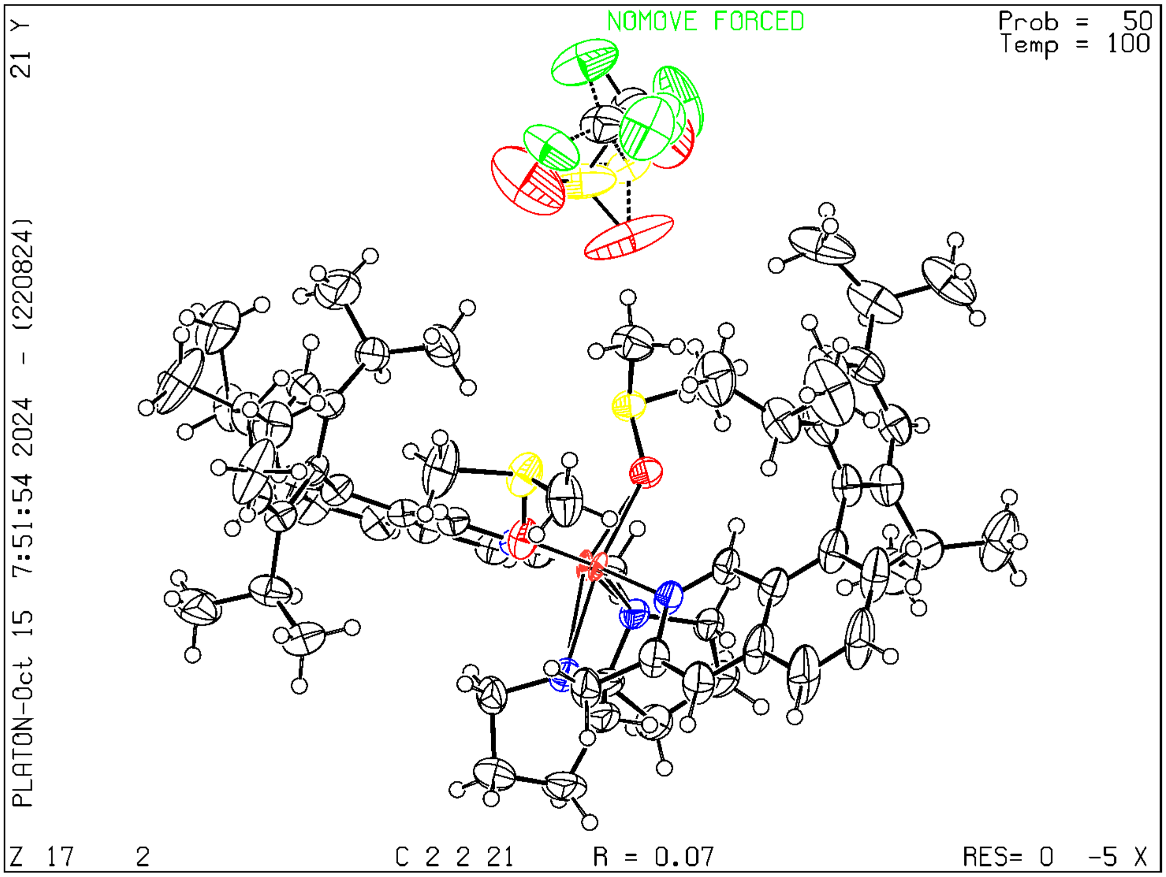

Figure 2. thermal ellipsoid plot of **Fe($^{3,5\text{-(di-CF3)}}$iQ$_2$dp)** showing 50% probability displacement ellipsoids

6. Spectral (^{1}H & ^{13}C) data.

^{1}H-NMR spectrum of compound **2a**: (400 MHz, $CDCl_3$)

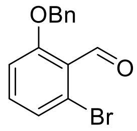

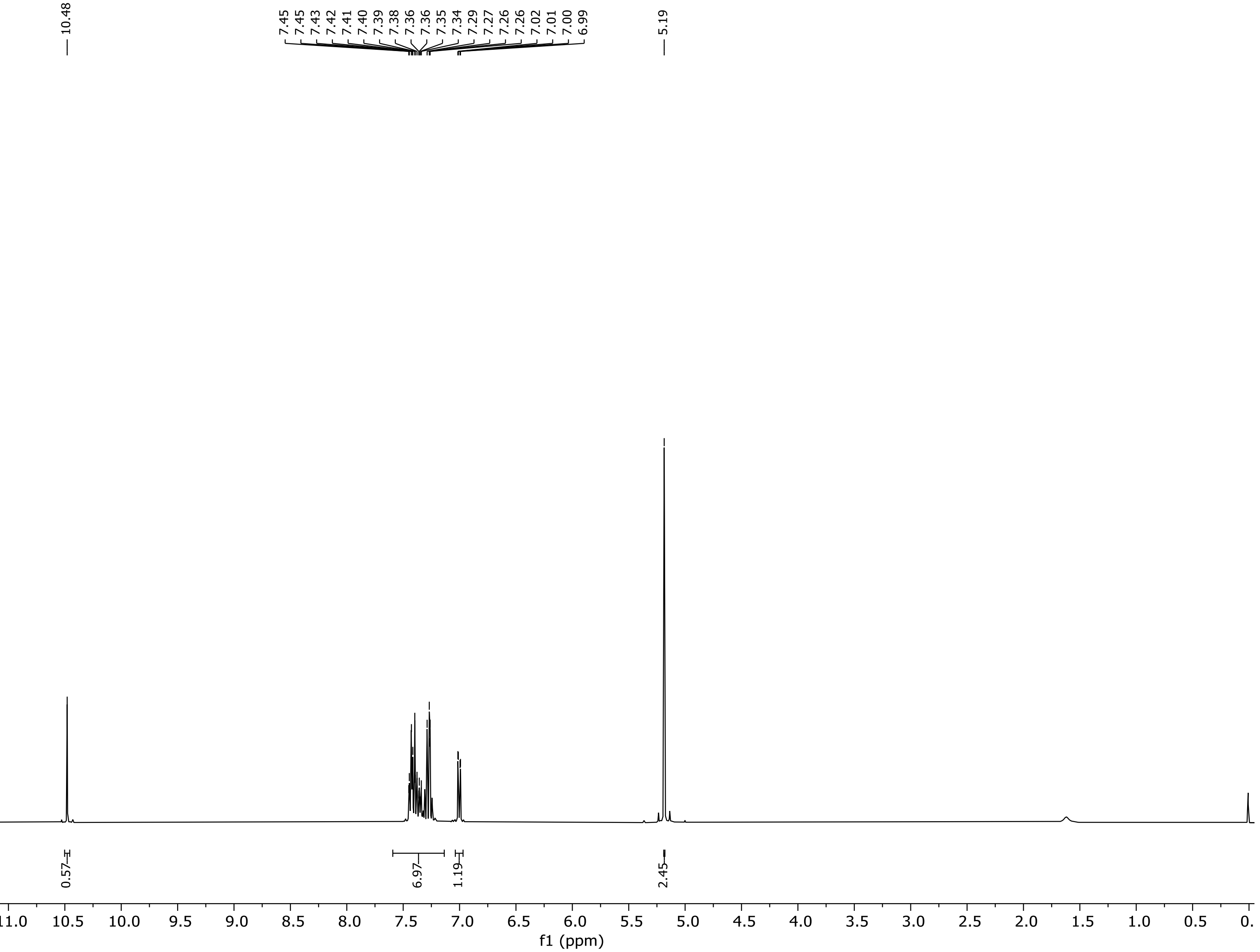

^{13}C-NMR spectrum of compound **2a** (100 MHz, $CDCl_3$)

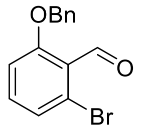

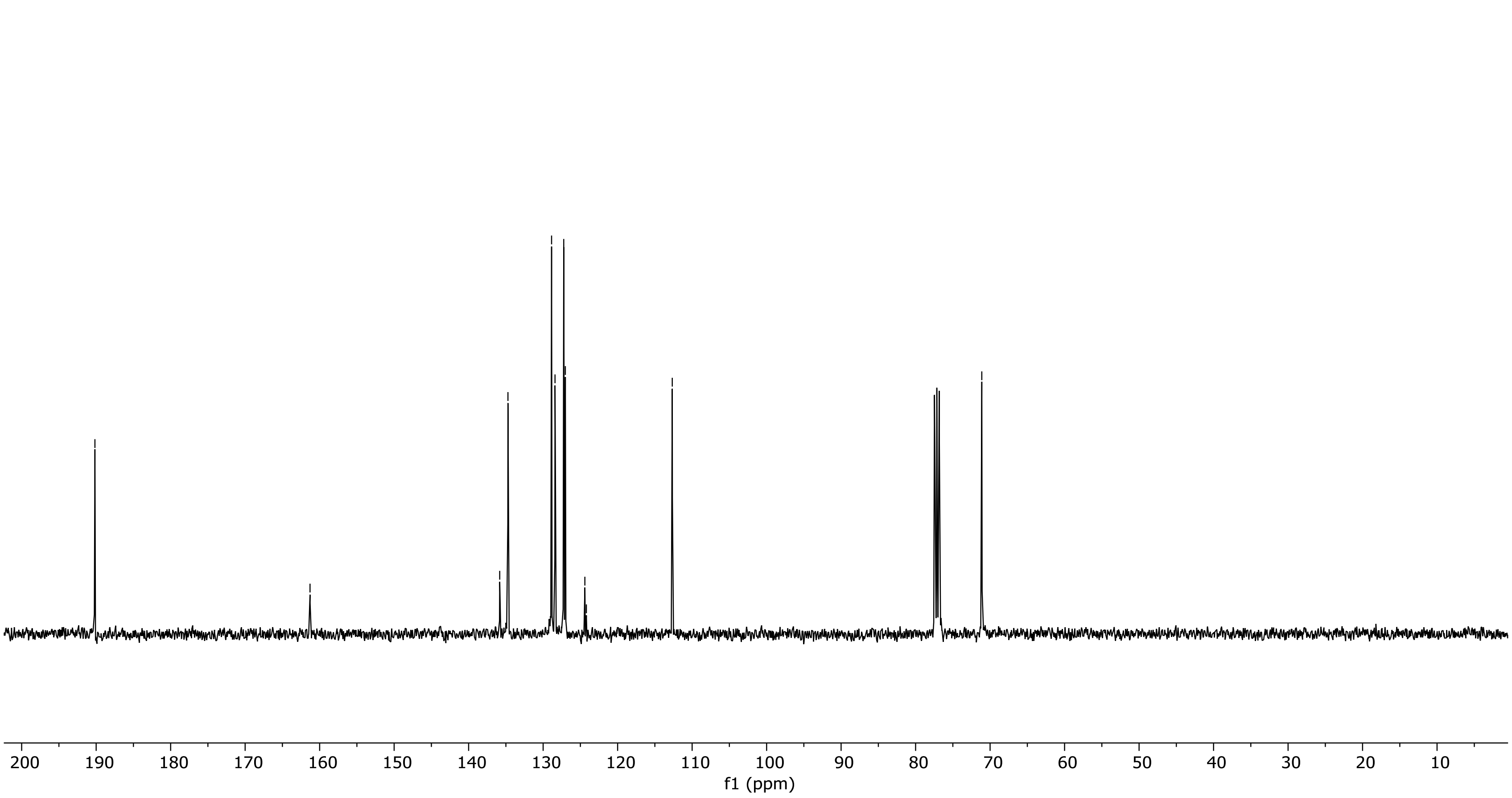

^{1}H-NMR spectrum of compound **2b**: (400 MHz, $CDCl_3$)

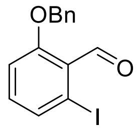

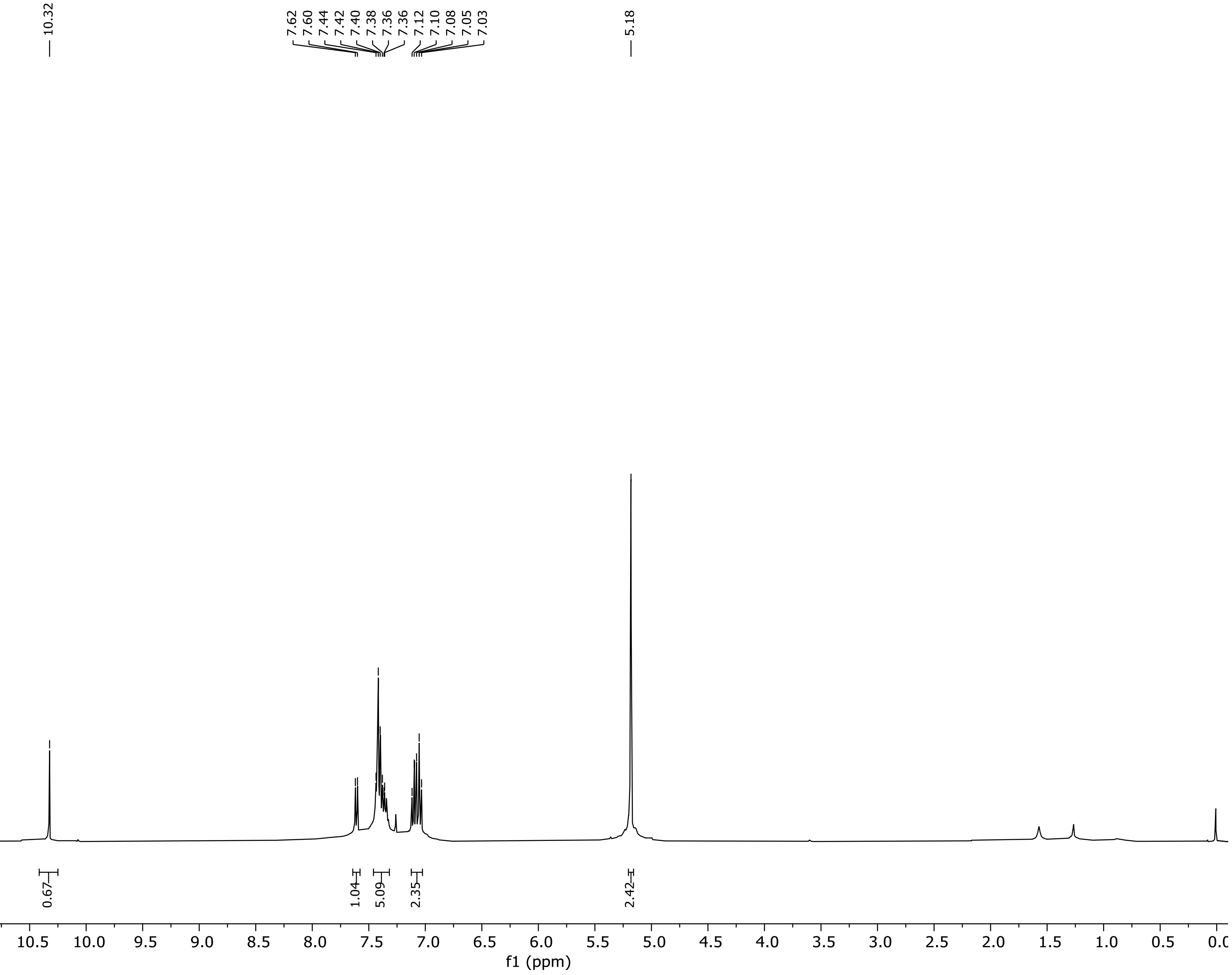

^{13}C-NMR spectrum of compound **2b**: (100 MHz, $CDCl_3$)

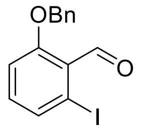

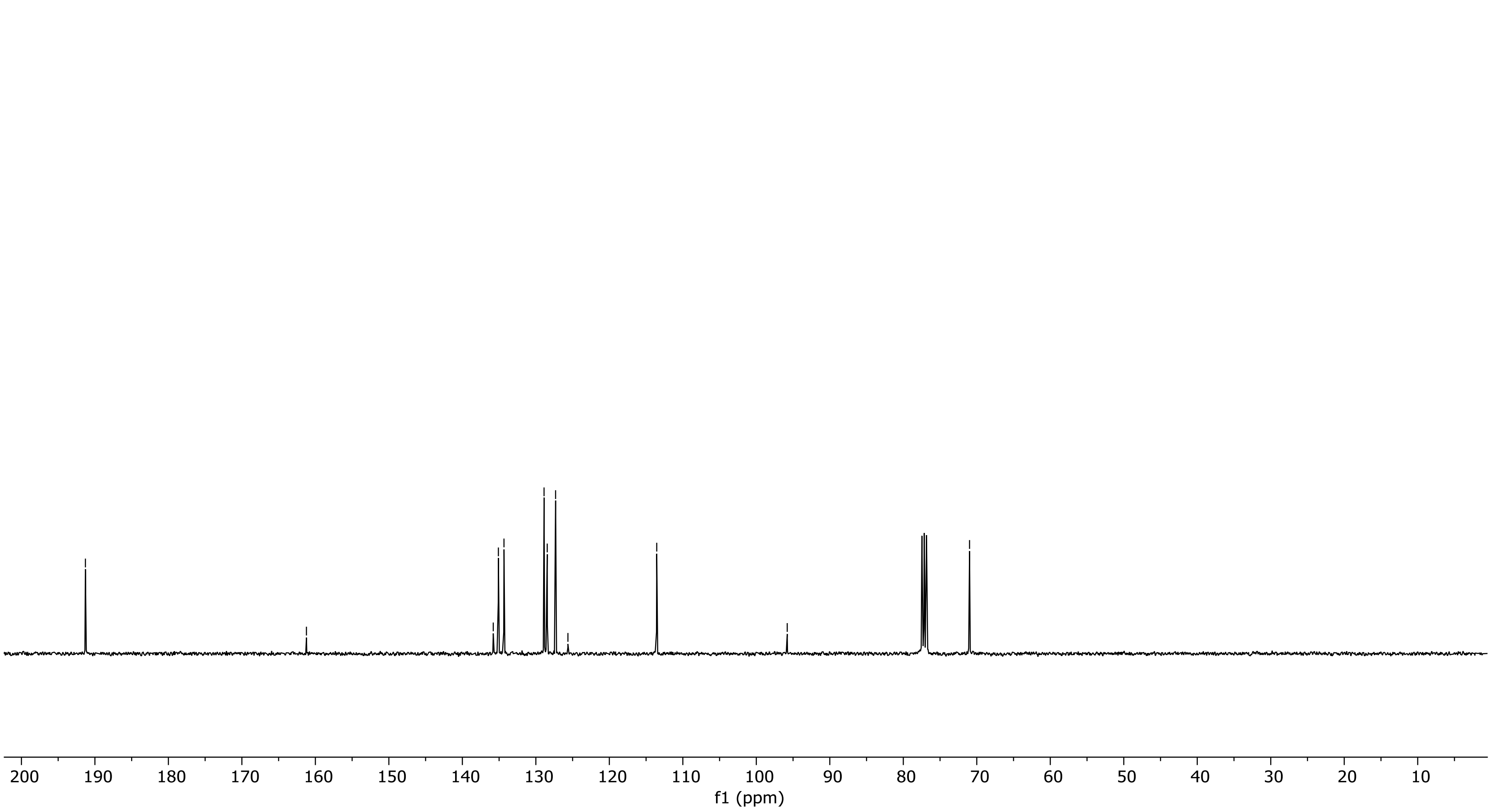

^{1}H-NMR spectrum of compound **4**: (400 MHz, $CDCl_3$)

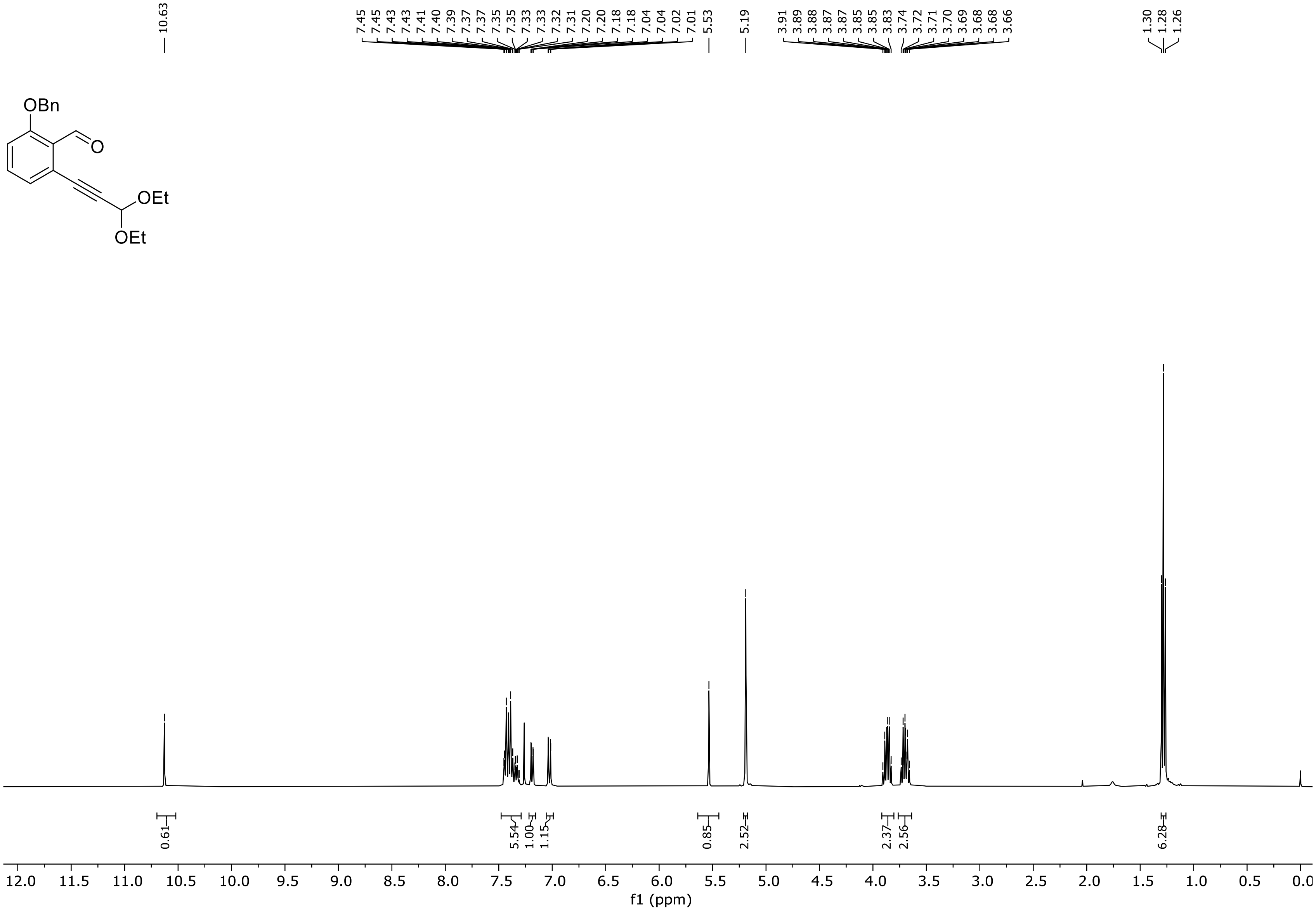

^{13}C-NMR spectrum of compound **4**: (100 MHz, $CDCl_3$)

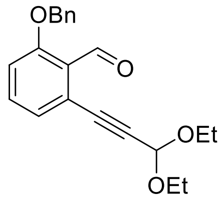

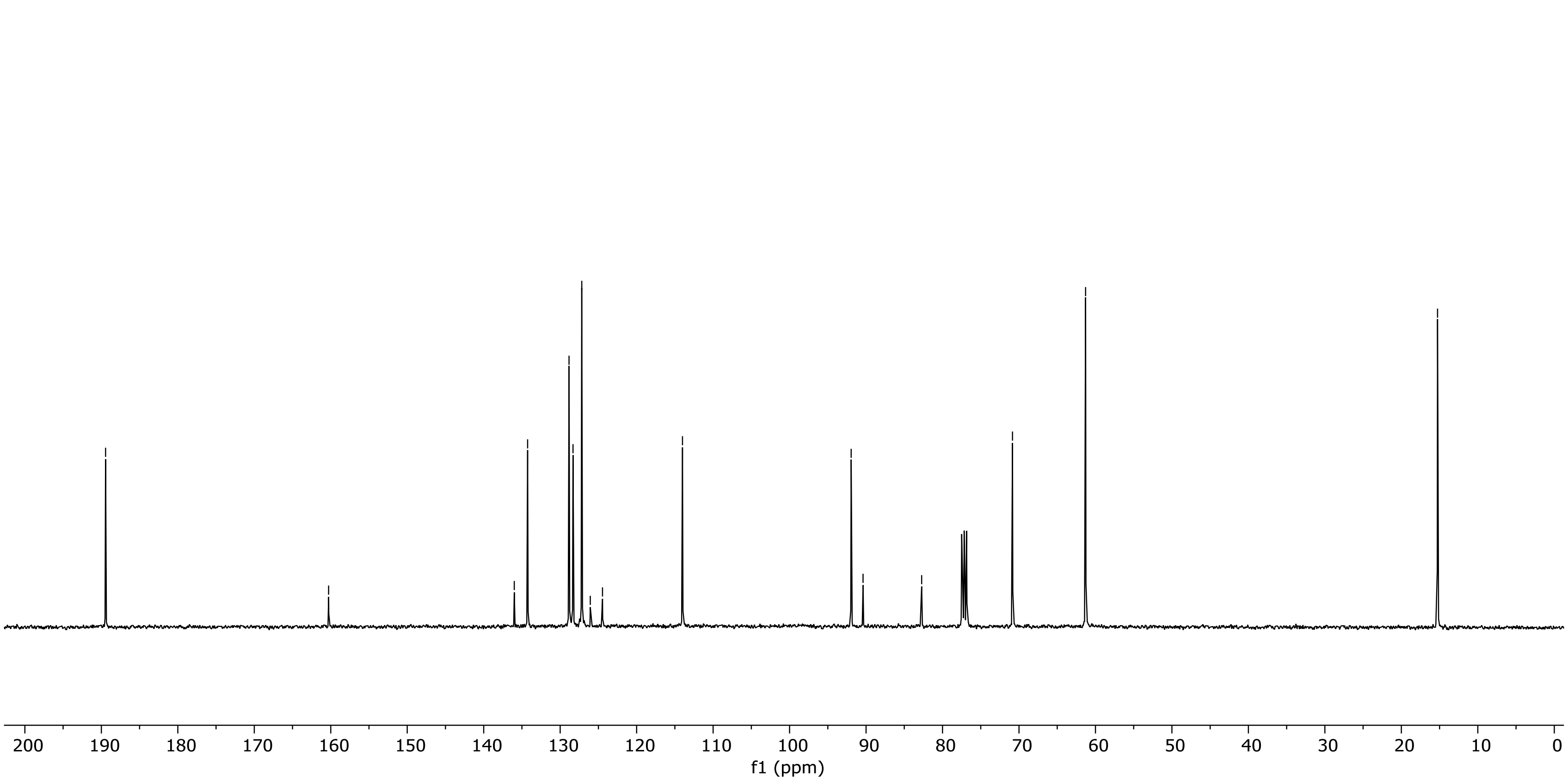

^{1}H-NMR spectrum of compound **5**: (400 MHz, $CDCl_3$)

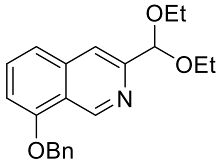

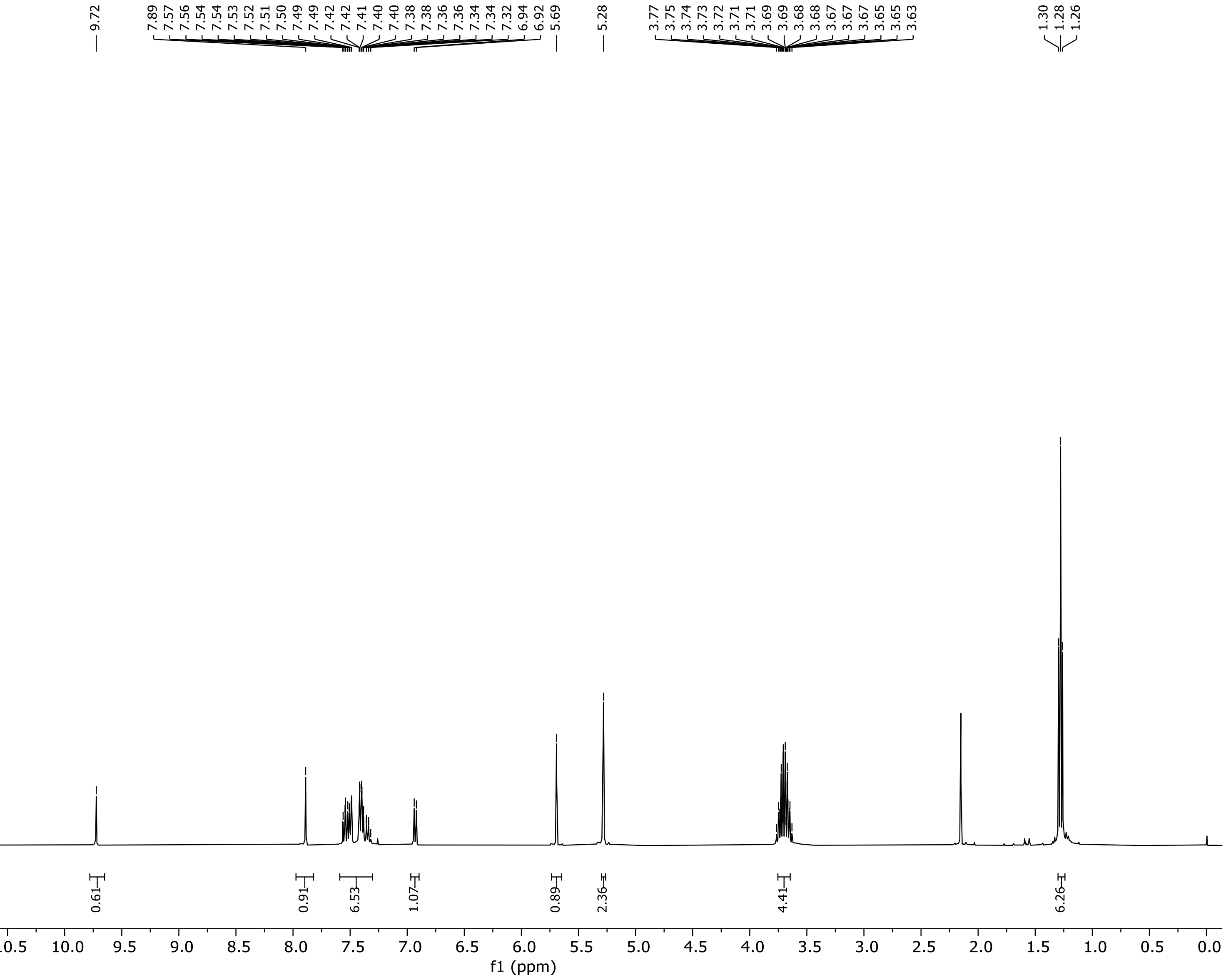

^{13}C-NMR spectrum of compound **5**: (100 MHz, $CDCl_3$)

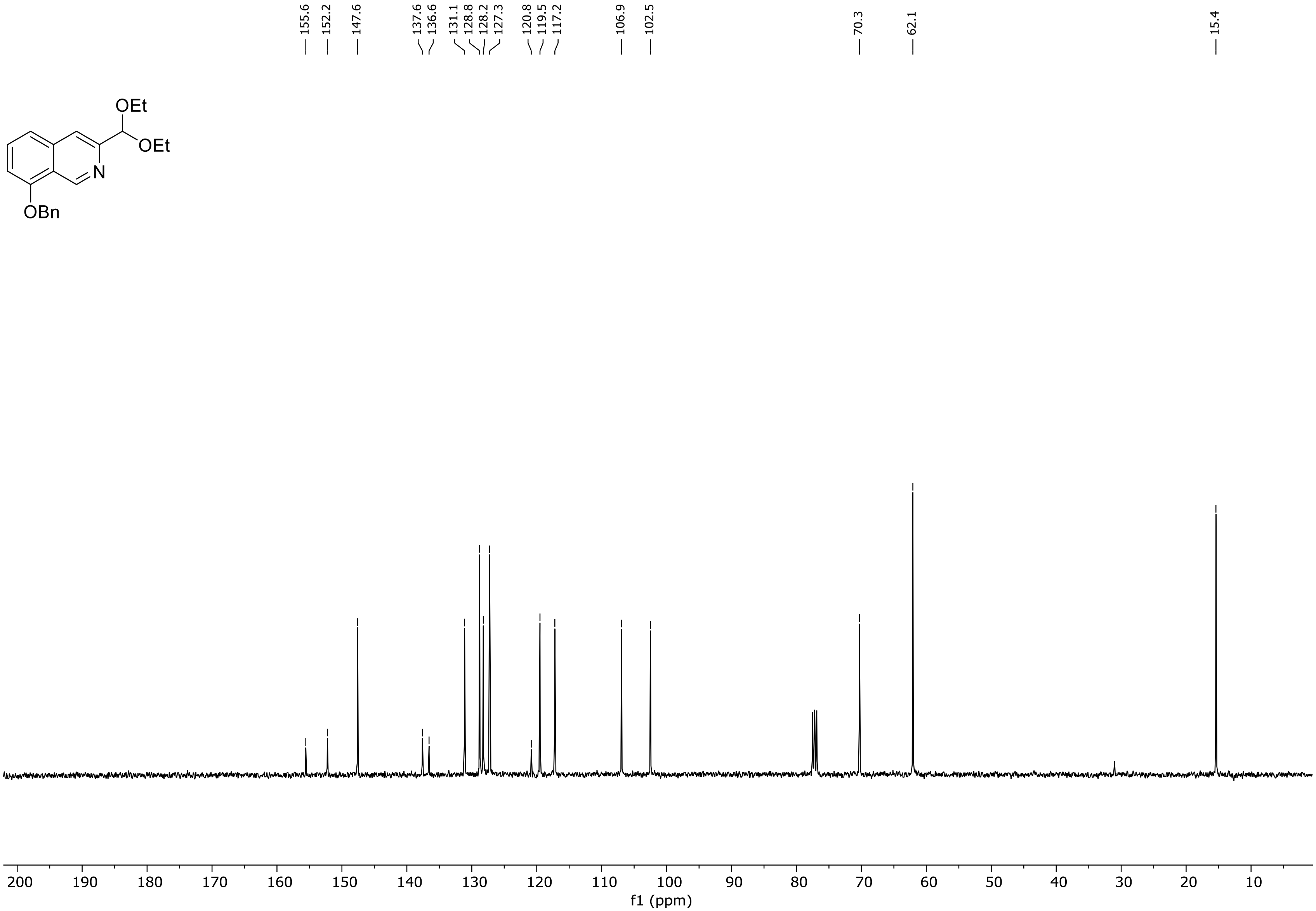

^{1}H-NMR spectrum of compound **5-OH**: (400 MHz, $CDCl_3$)

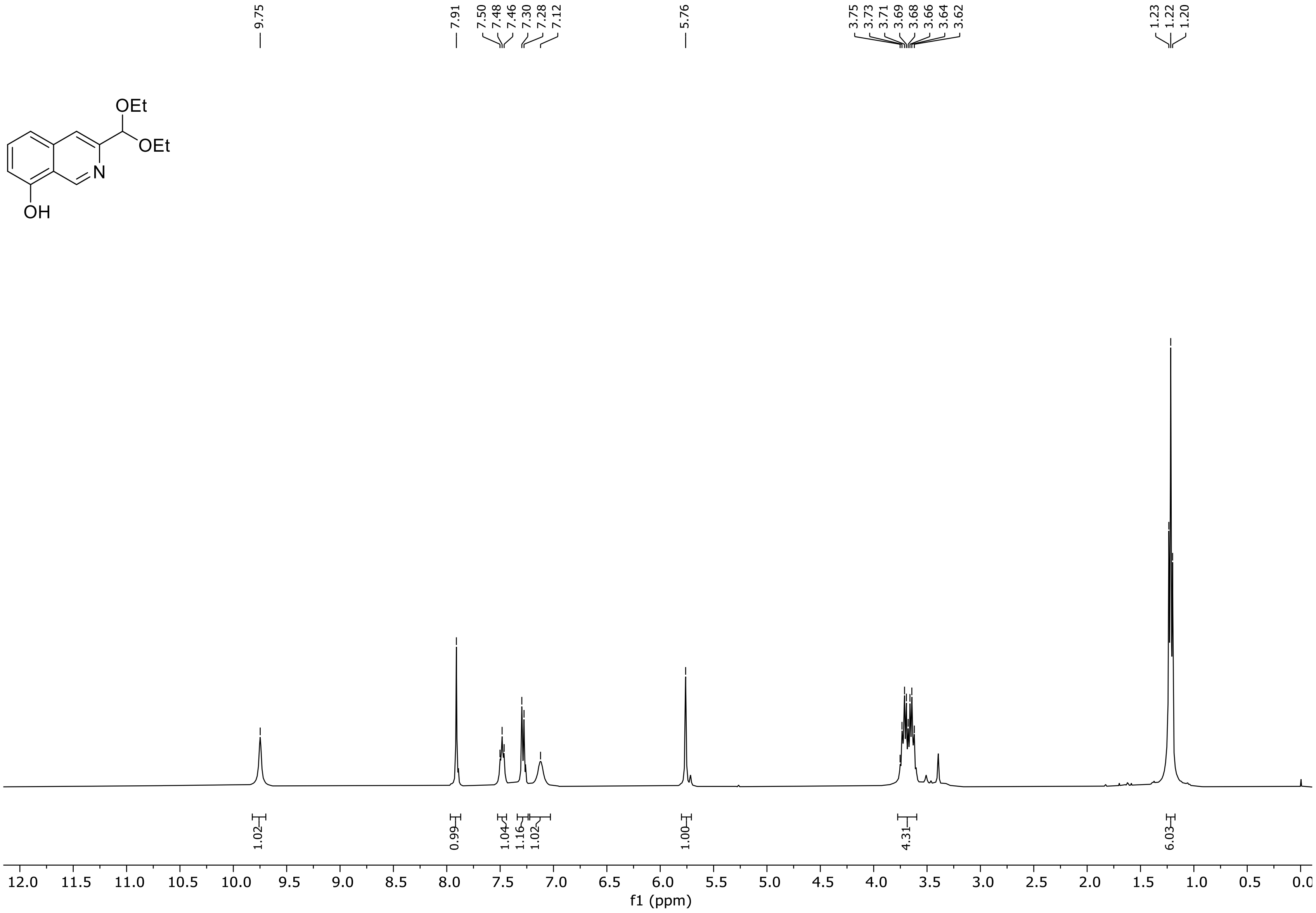

^{13}C-NMR spectrum of compound **5-OH**: (100 MHz, $CDCl_3$)

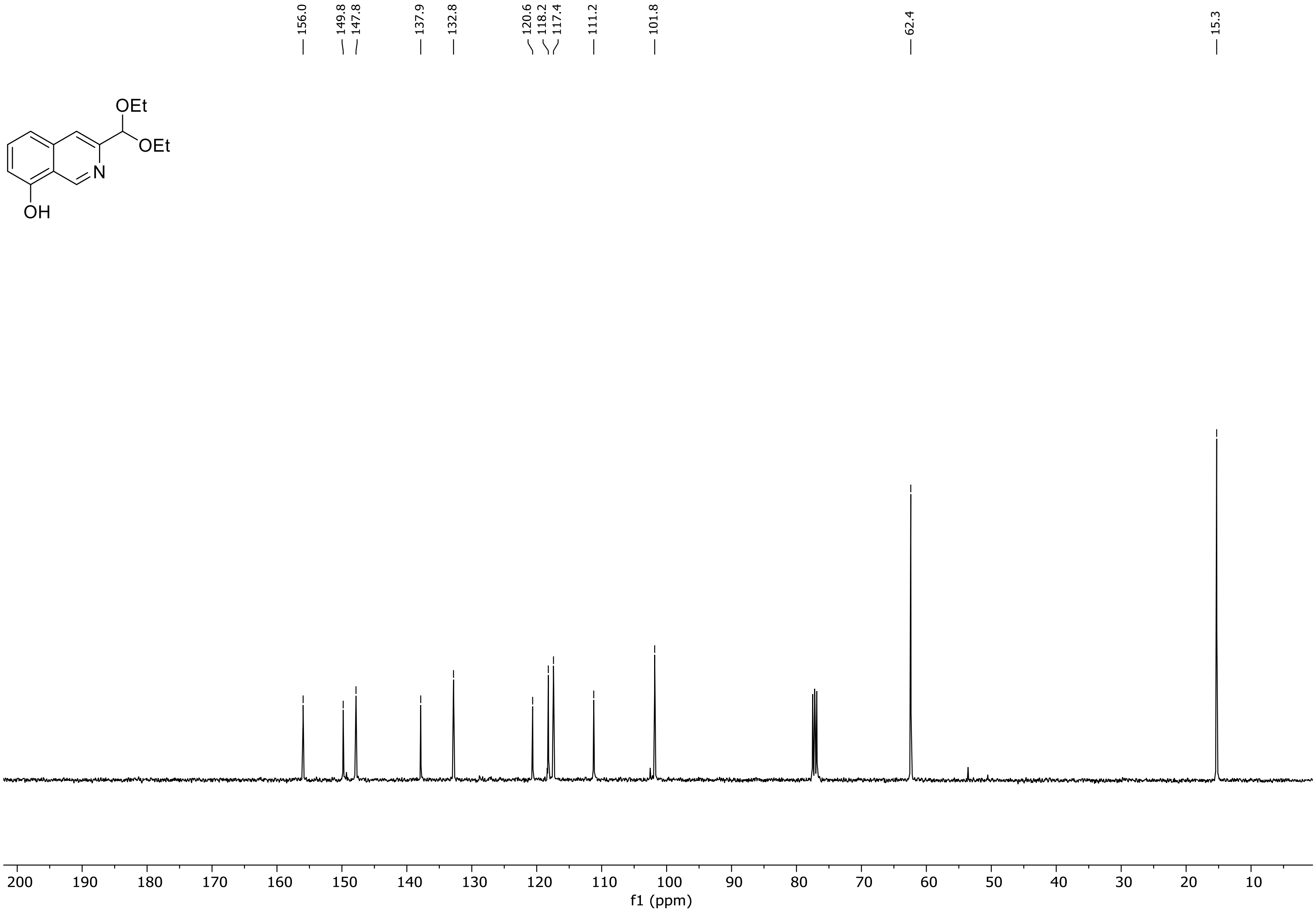

^{1}H-NMR spectrum of compound **6**: (400 MHz, $CDCl_3$)

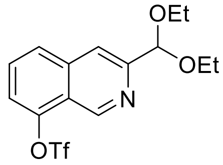

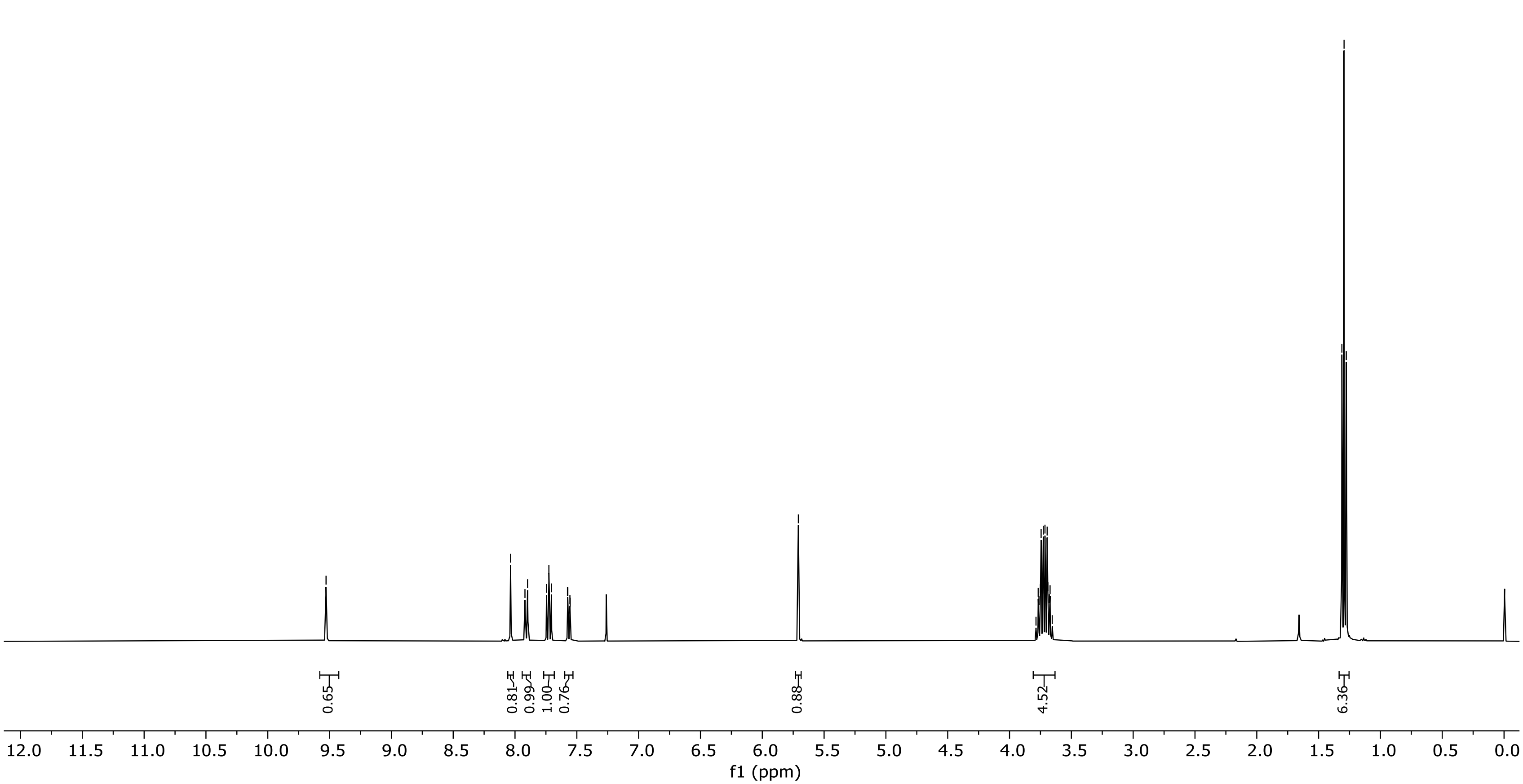

^{13}C-NMR spectrum of compound **6**: (100 MHz, $CDCl_3$)

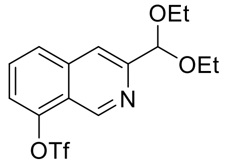

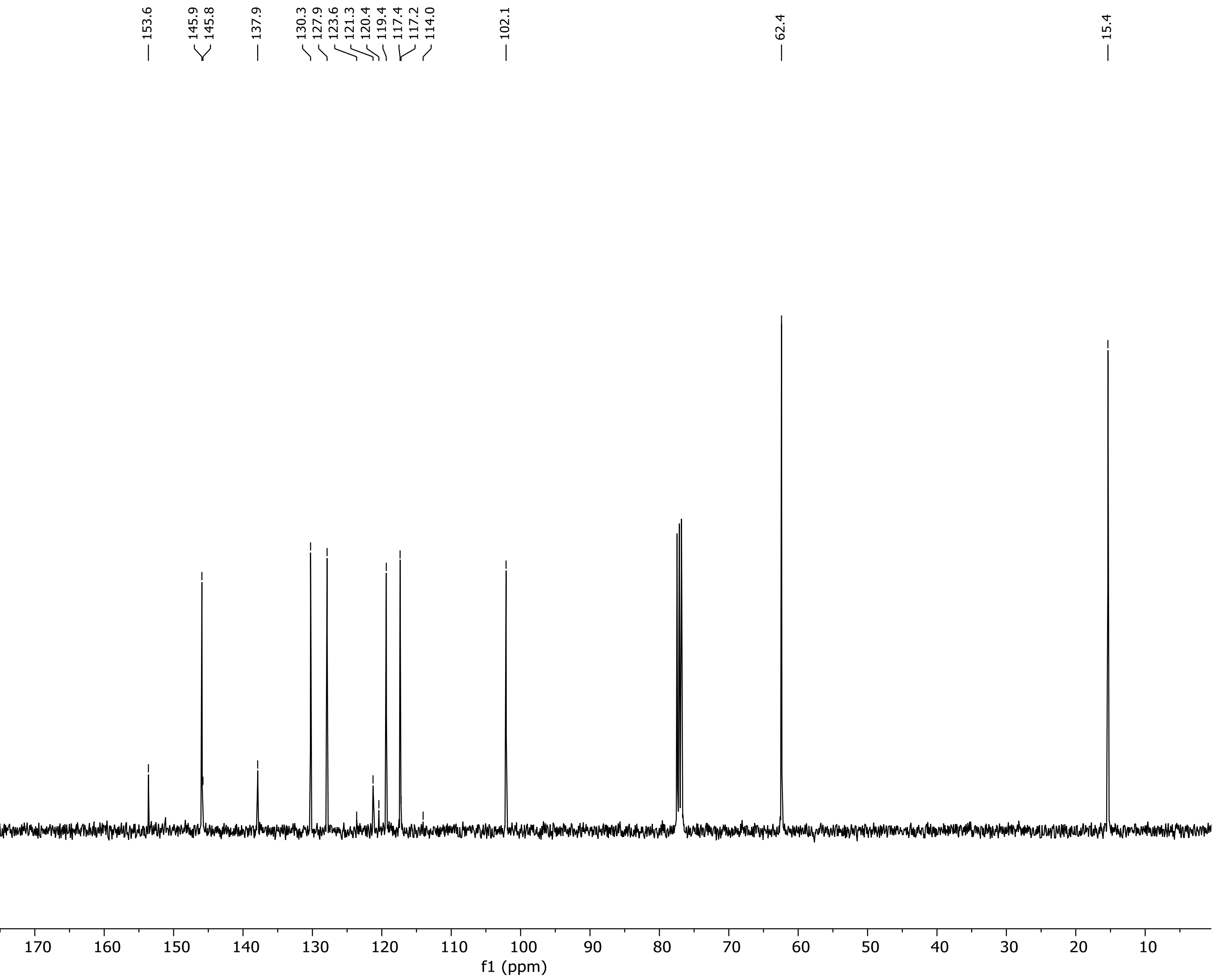

^{19}F-NMR spectrum of compound **6**: (376 MHz, $CDCl_3$)

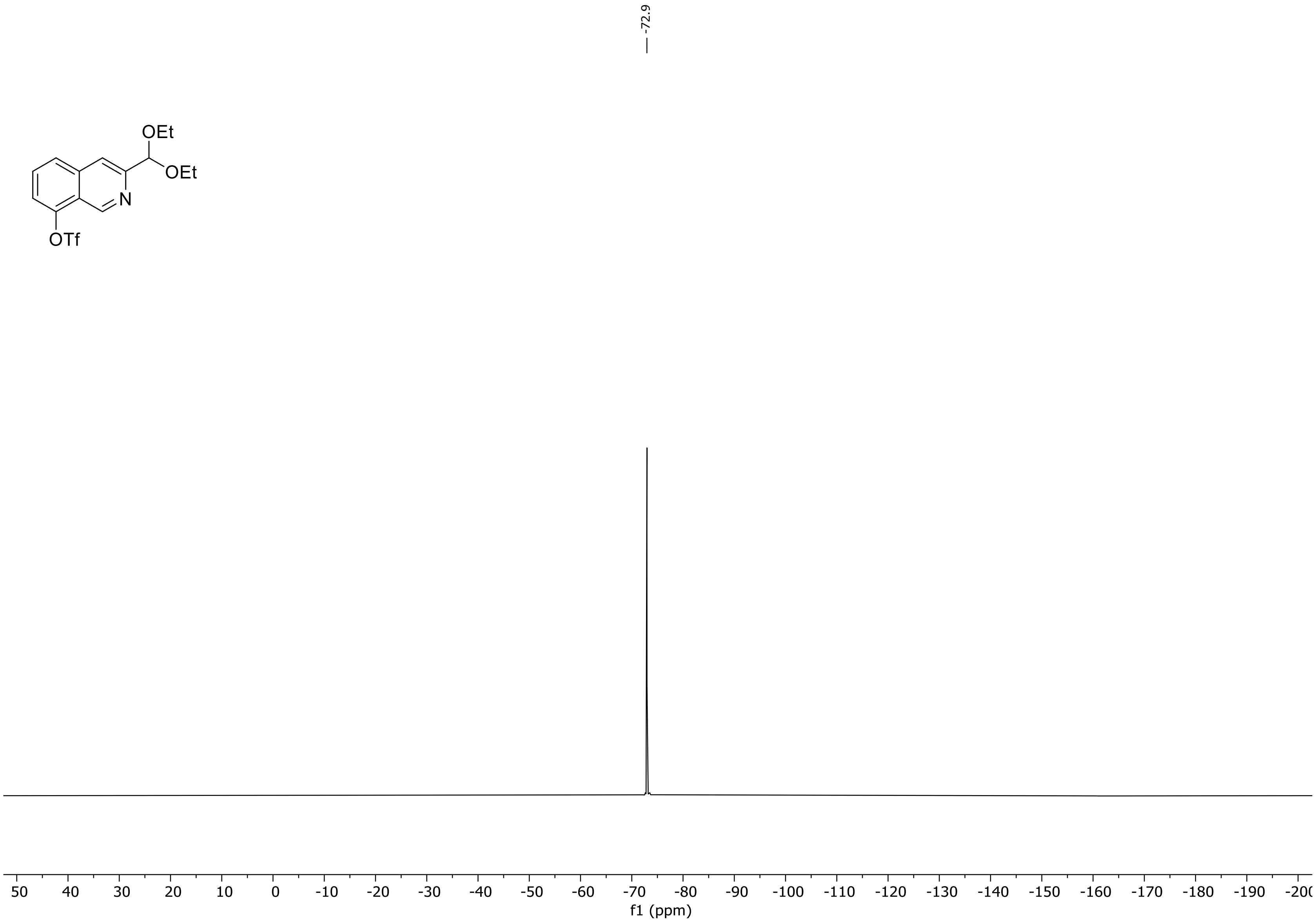

^{1}H-NMR spectrum of compound **7**: (400 MHz, $CDCl_3$)

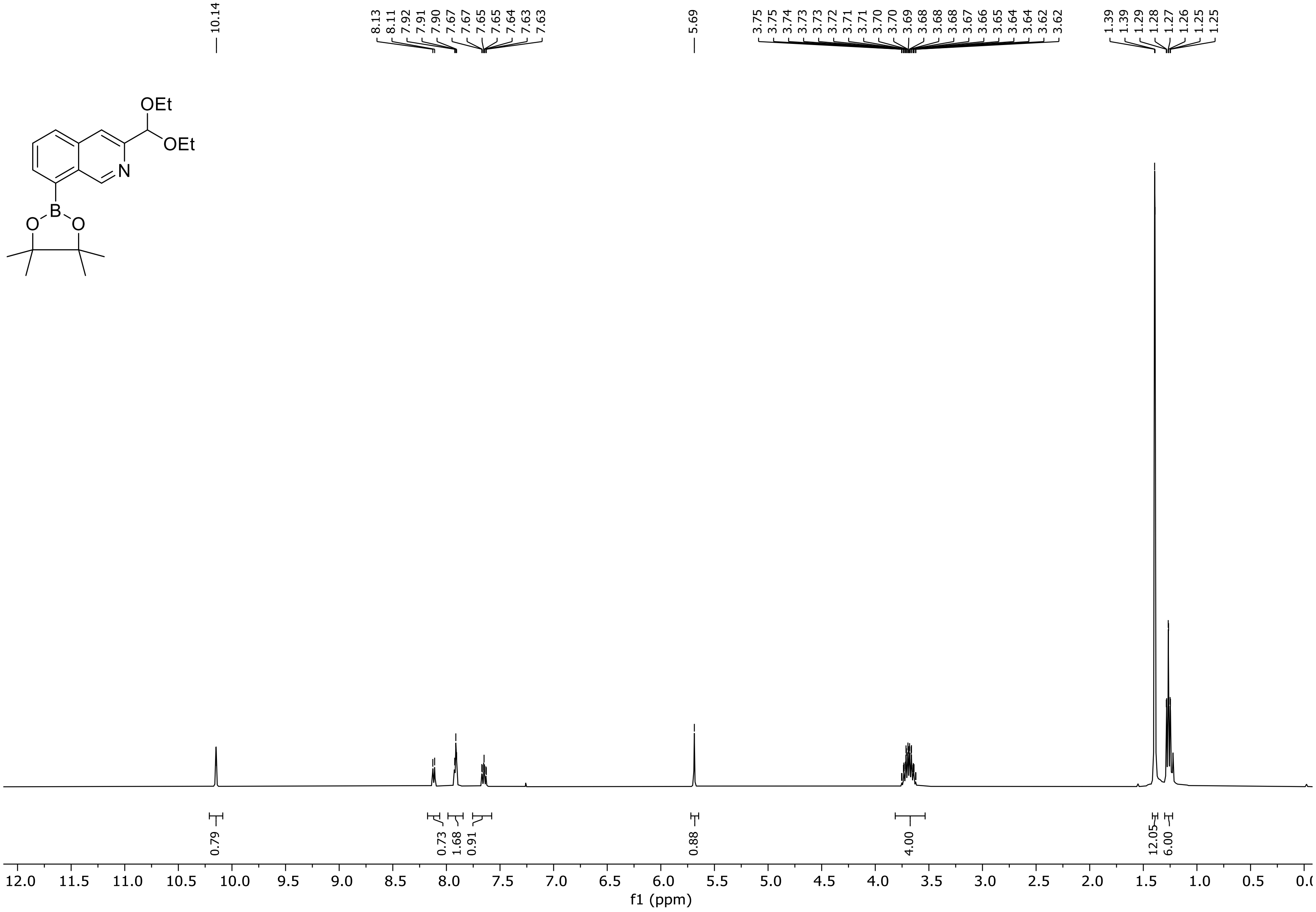

^{13}C-NMR spectrum of compound **7**: (100 MHz, $CDCl_3$)

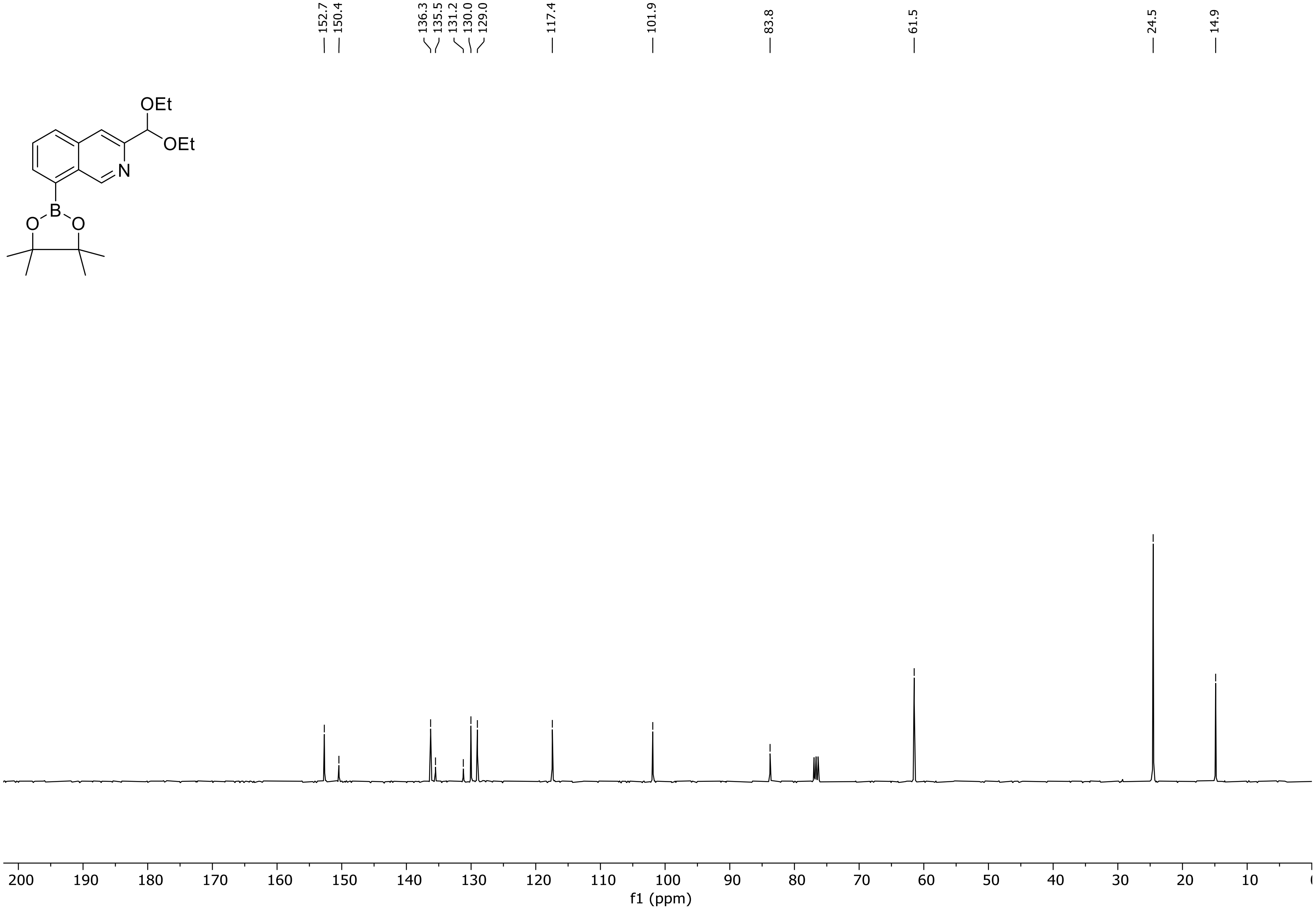

^{1}H-NMR spectrum of compound **8’a**: (400 MHz, $CDCl_3$)

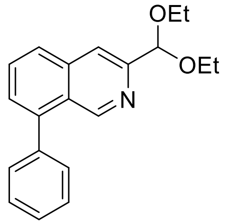

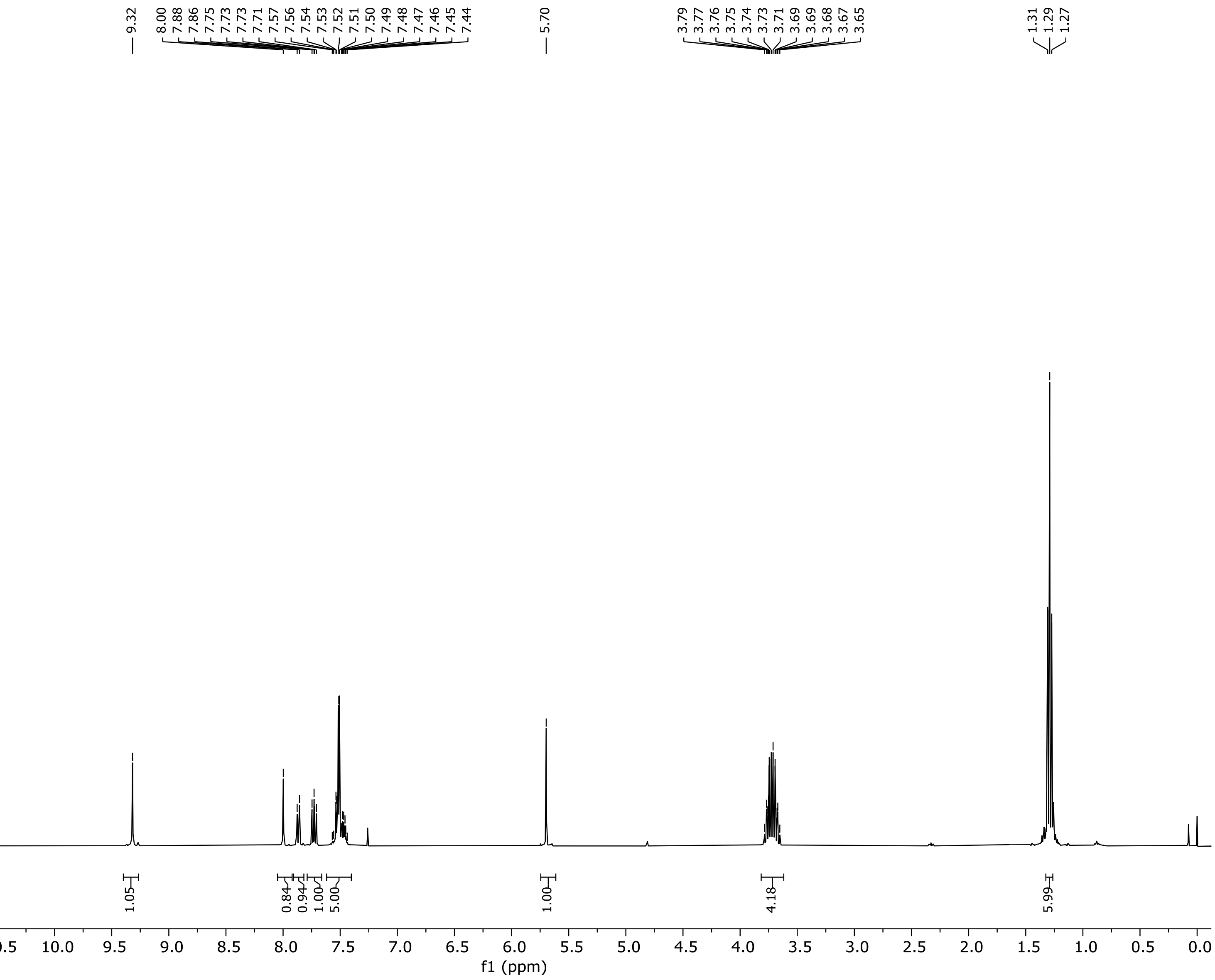

^{13}C-NMR spectrum of compound **8’a**: (100 MHz, $CDCl_3$)

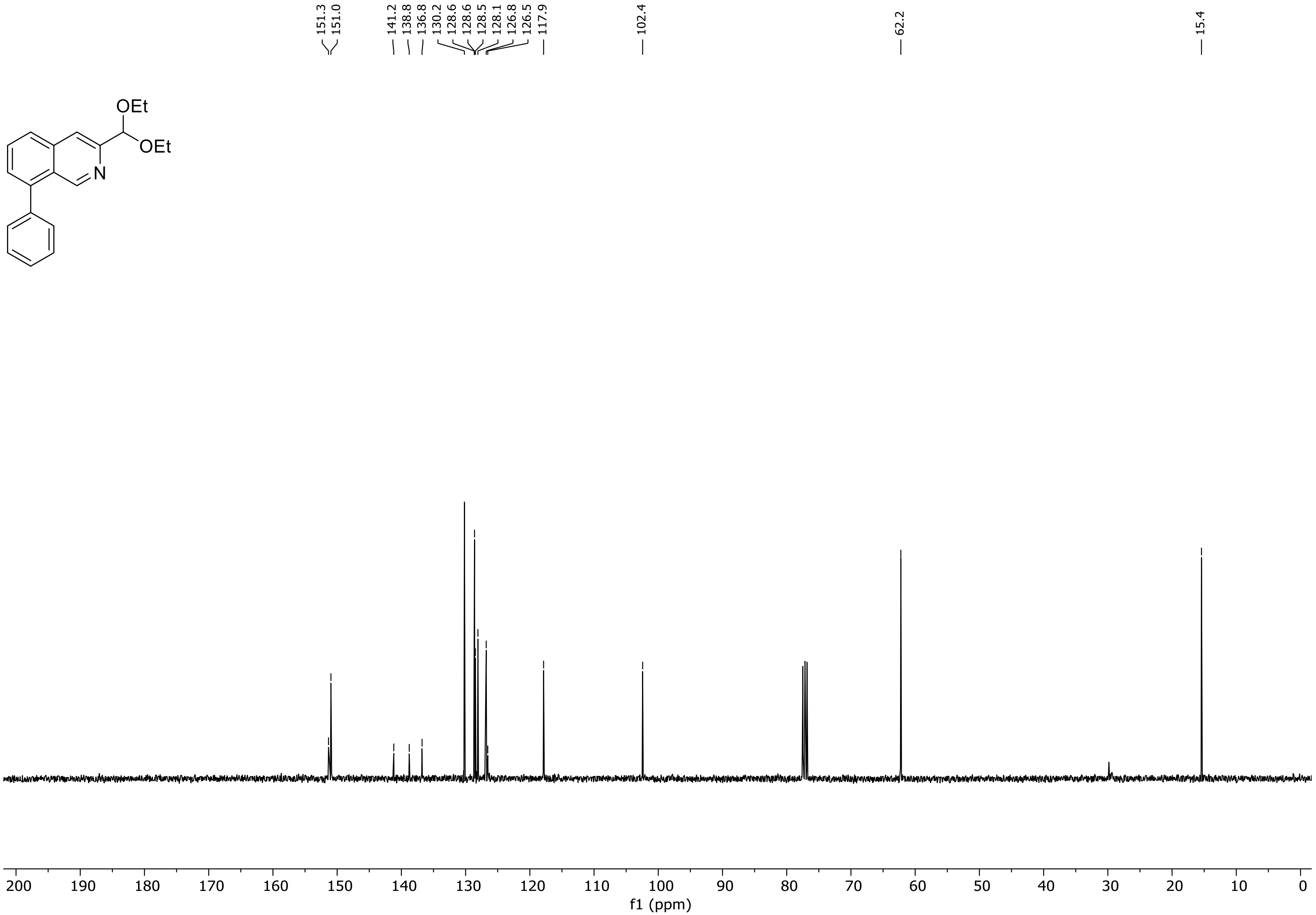

^{1}H-NMR spectrum of compound **8a**: (400 MHz, $CDCl_3$)

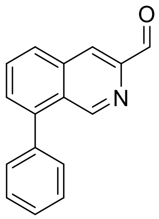

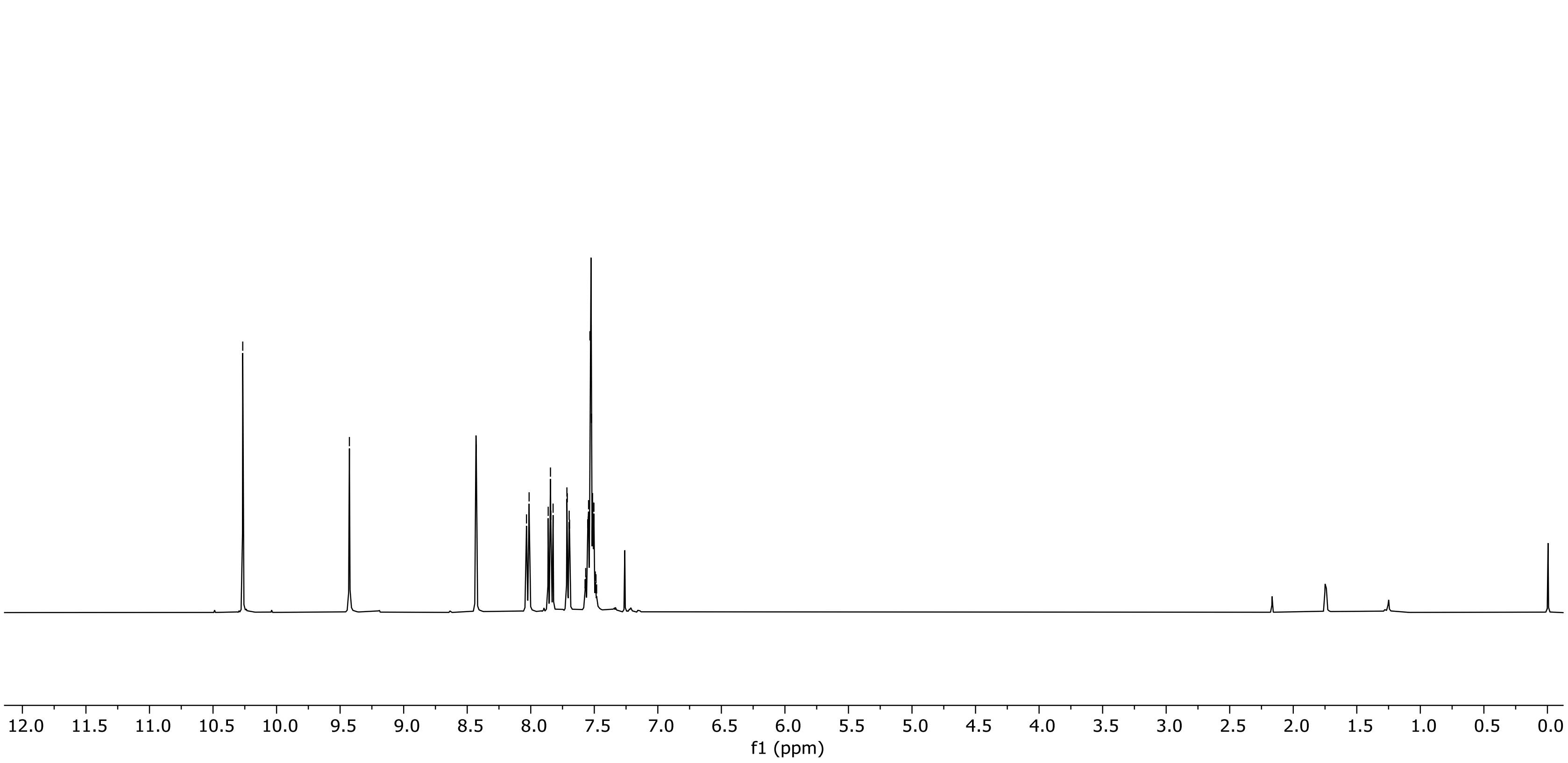

^{13}C-NMR spectrum of compound **8a**: (100 MHz, $CDCl_3$)

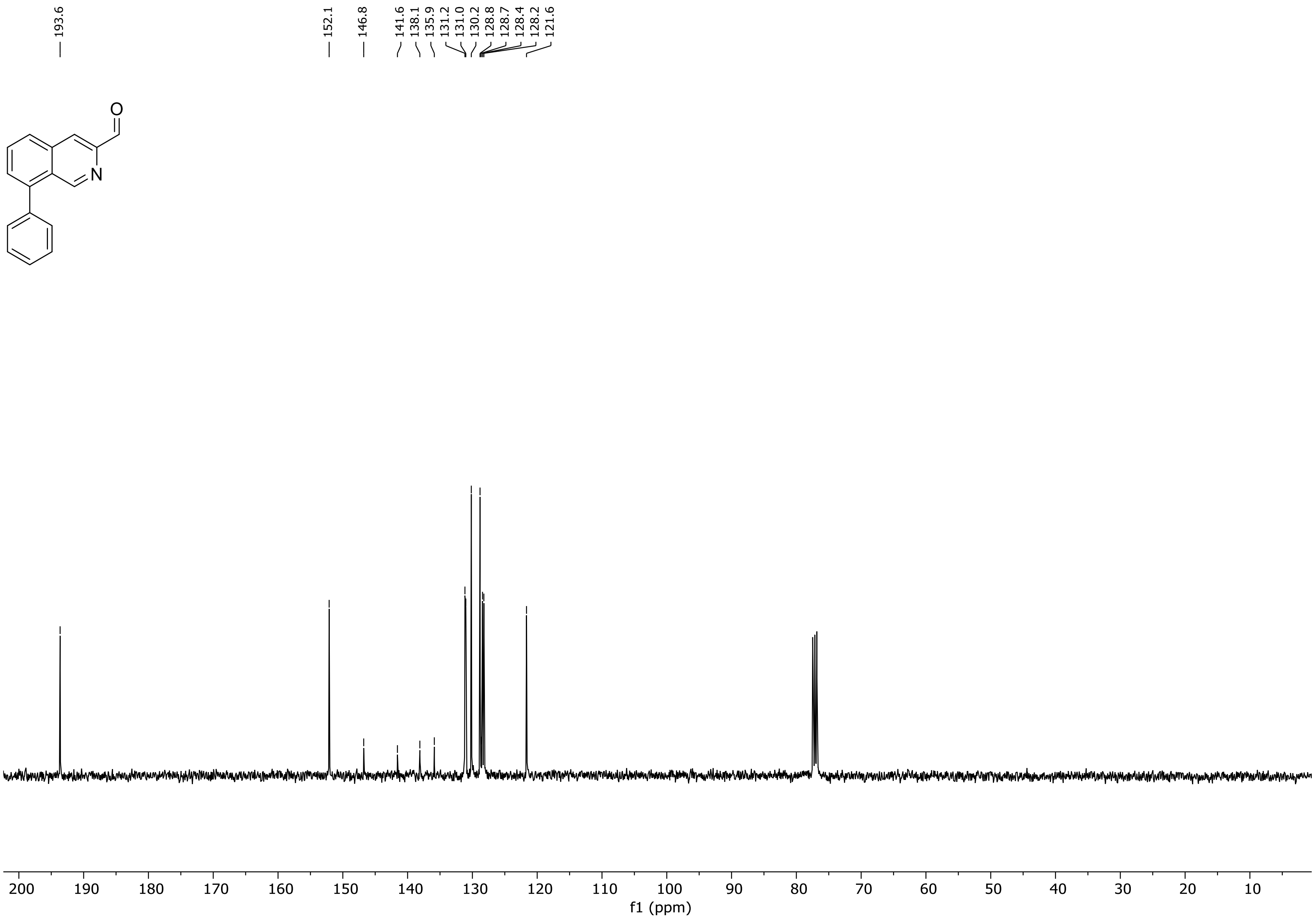

^{1}H-NMR spectrum of compound **8’b**: (400 MHz, $CDCl_3$)

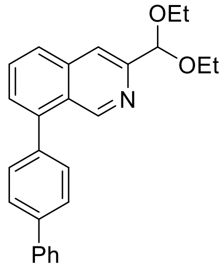

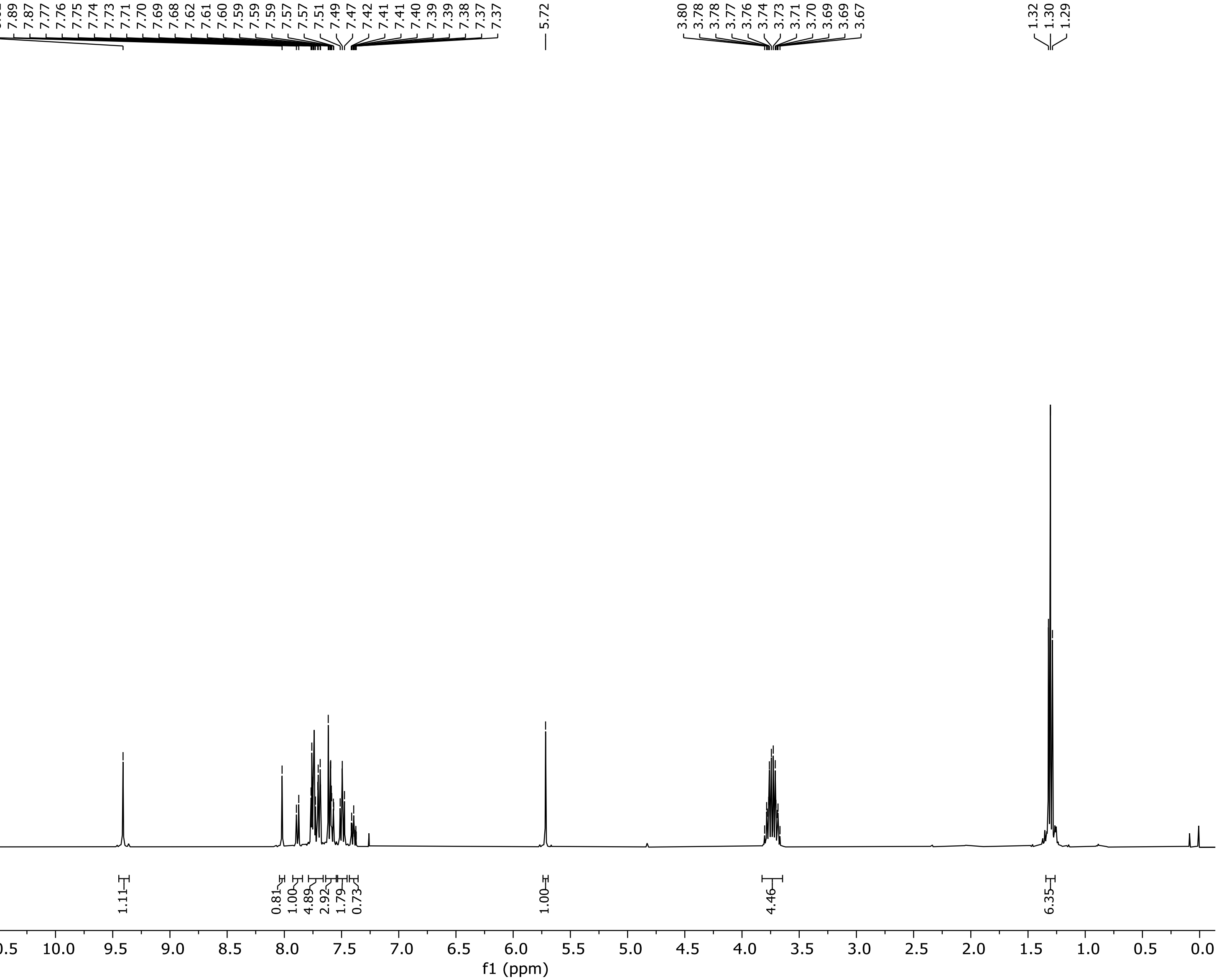

^{13}C-NMR spectrum of compound **8’b**: (100 MHz, $CDCl_3$)

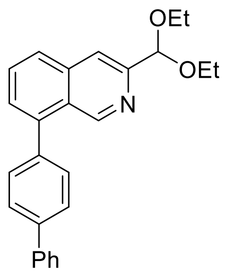

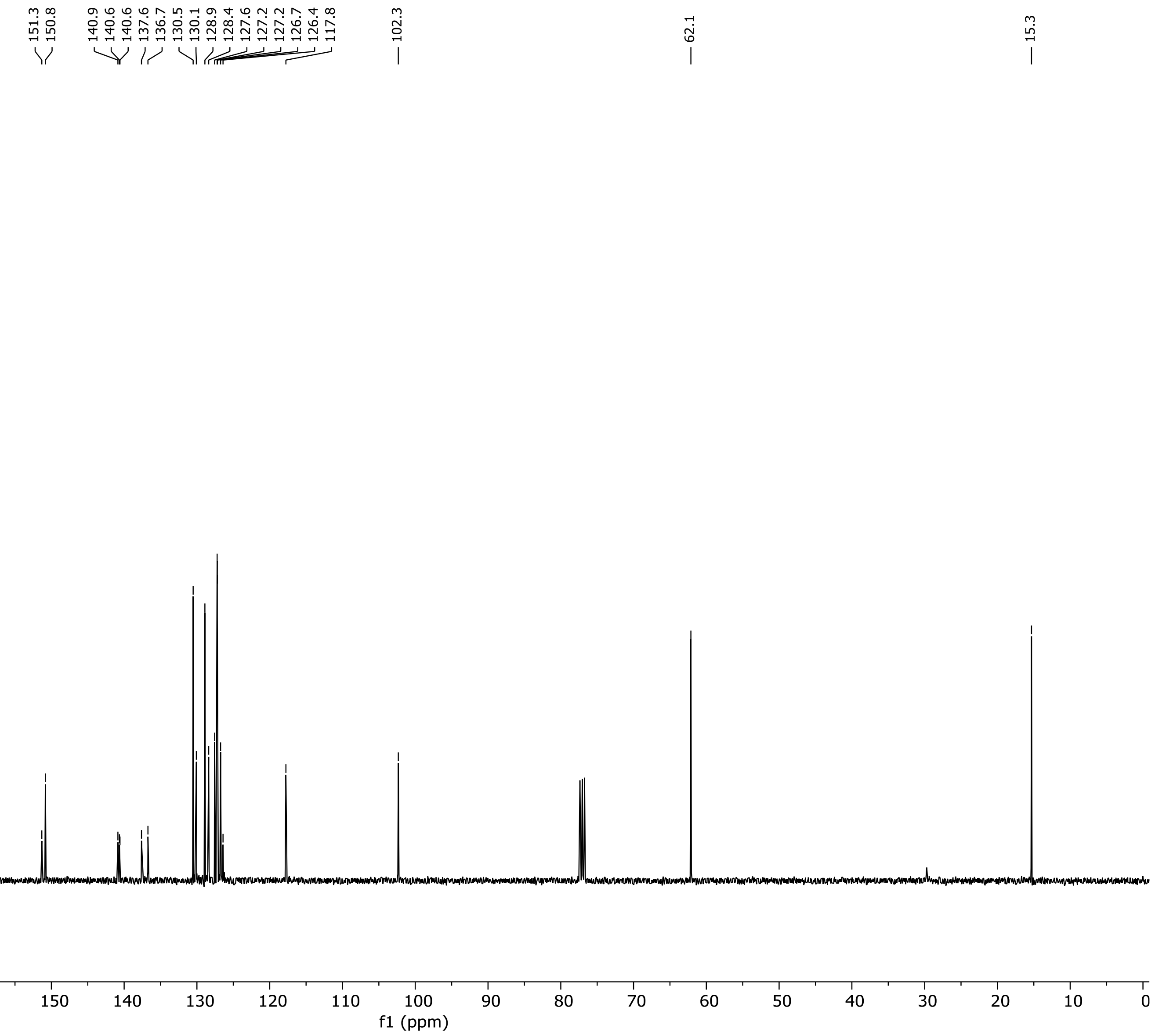

^{1}H-NMR spectrum of compound **8b**: (400 MHz, $CDCl_3$)

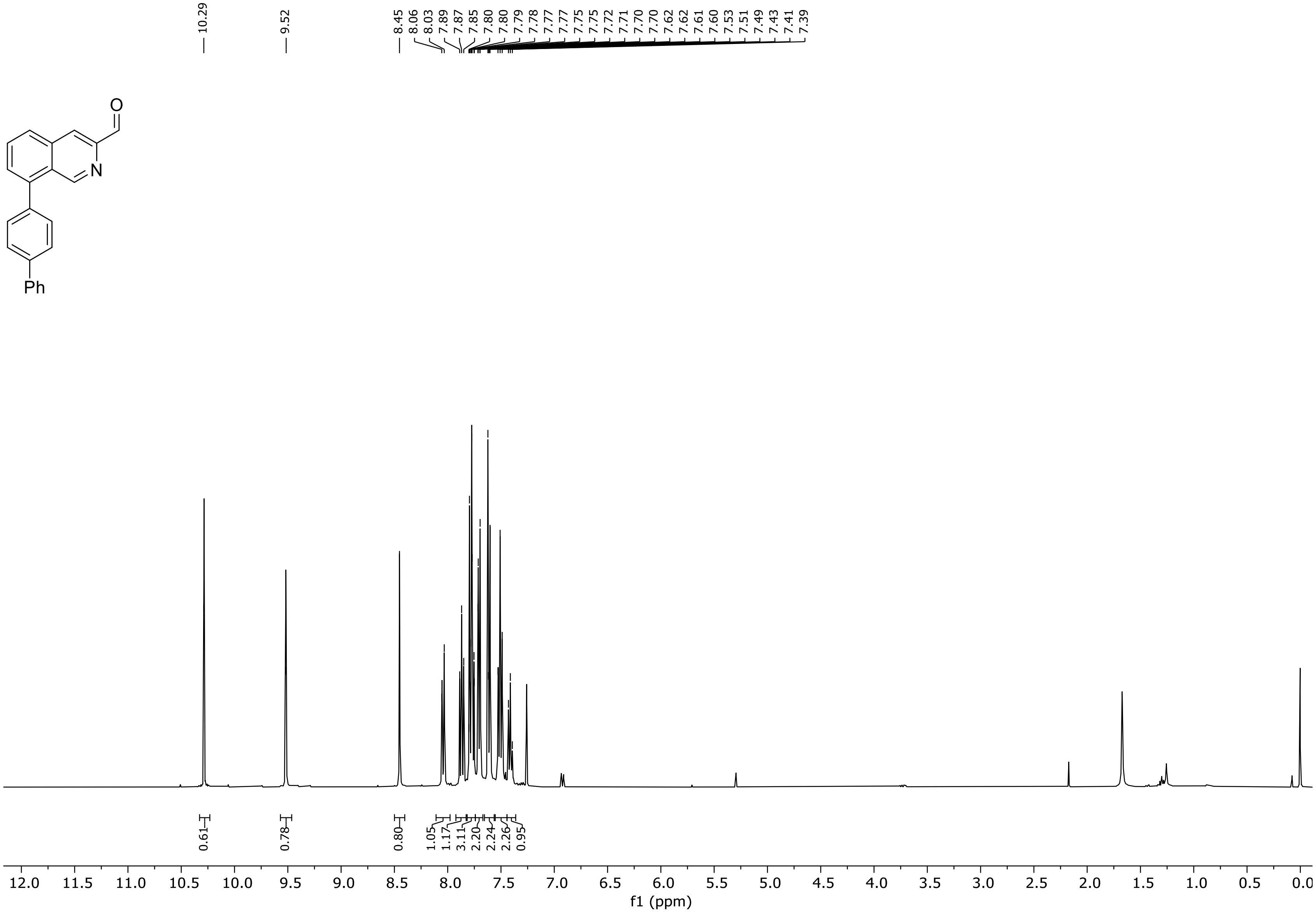

^{13}C-NMR spectrum of compound **8b**: (100 MHz, $CDCl_3$)

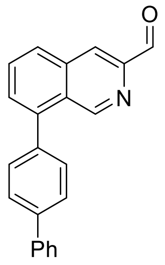

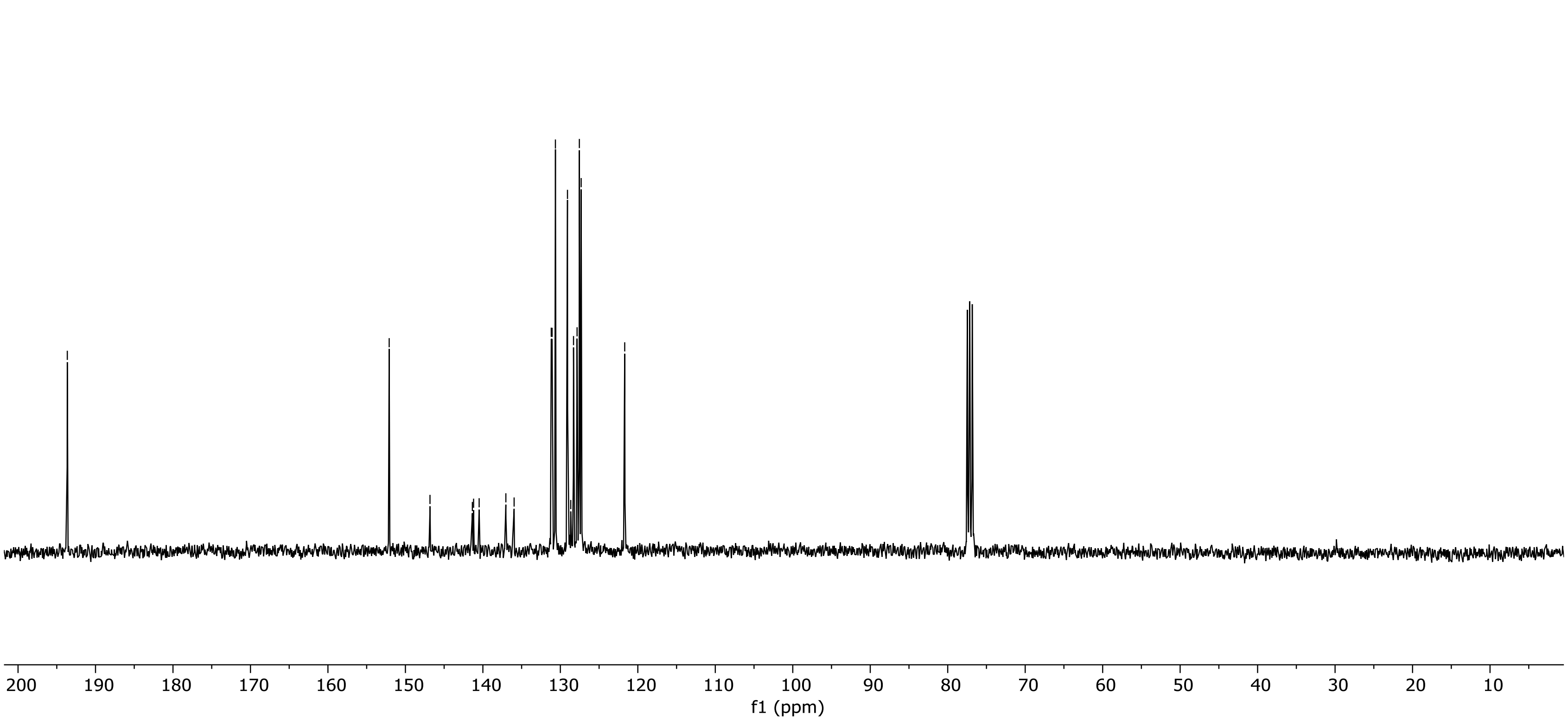

^{1}H-NMR spectrum of compound **8'c**: (500 MHz, $CDCl_3$)

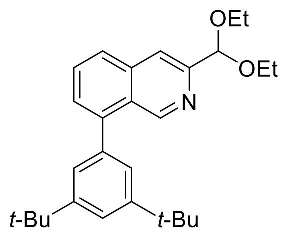

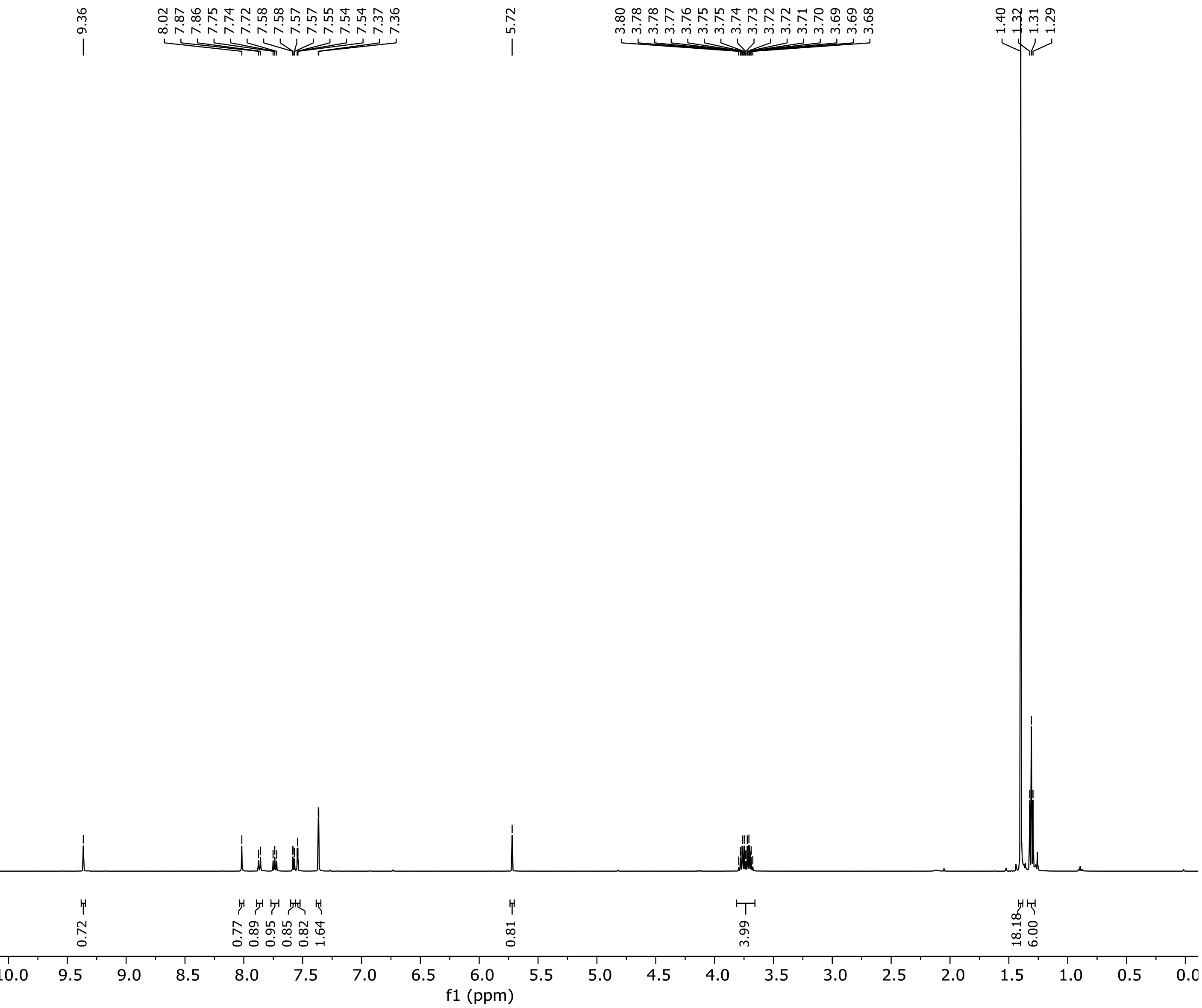

^{13}C-NMR spectrum of compound **8’c**: (125 MHz, $CDCl_3$)

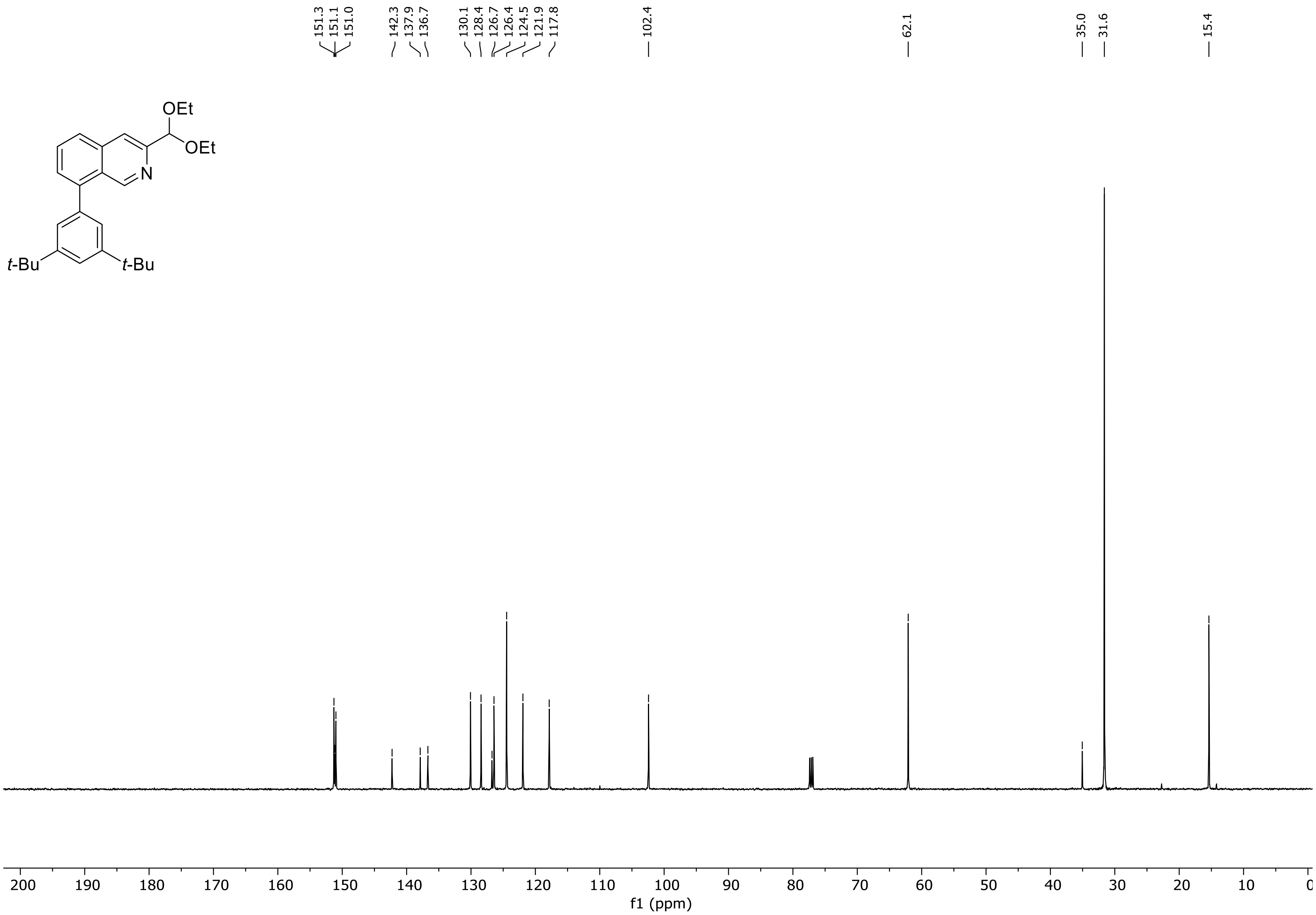

^{1}H-NMR spectrum of compound **8c**: (400 MHz, $CDCl_3$)

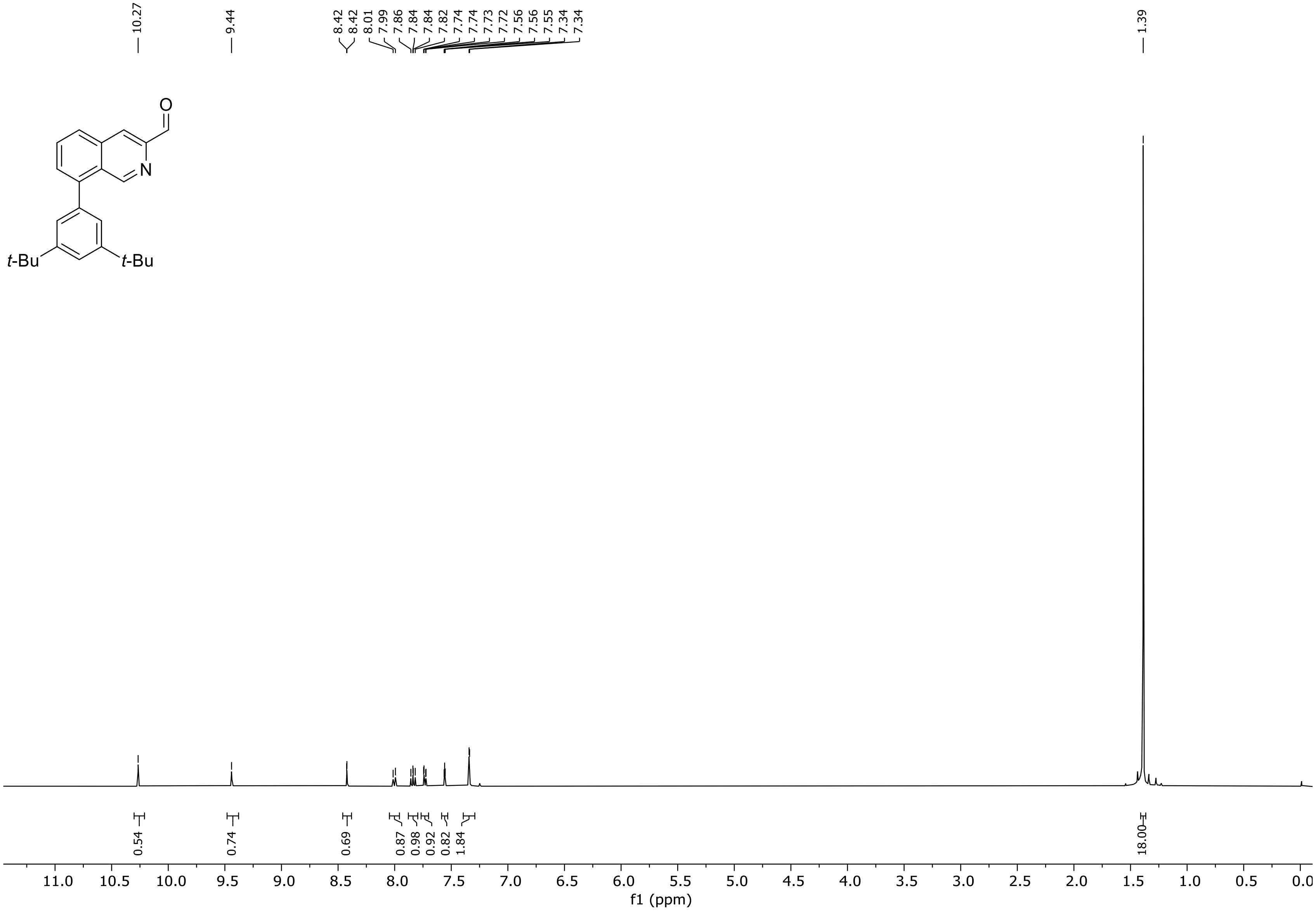

^{13}C-NMR spectrum of compound **8c**: (100 MHz, $CDCl_3$)

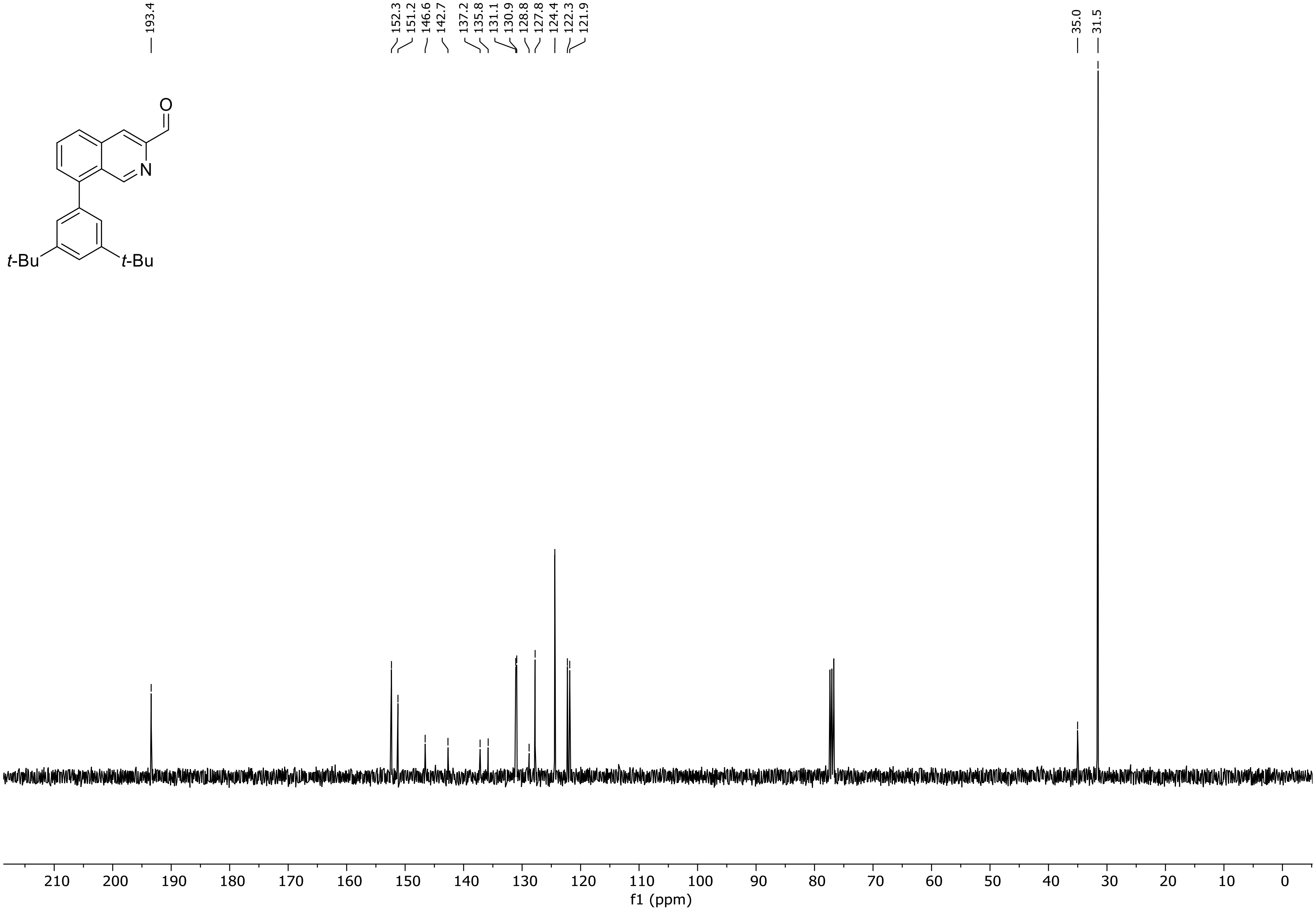

^{1}H-NMR spectrum of compound **8’d**: (400 MHz, $CDCl_3$)

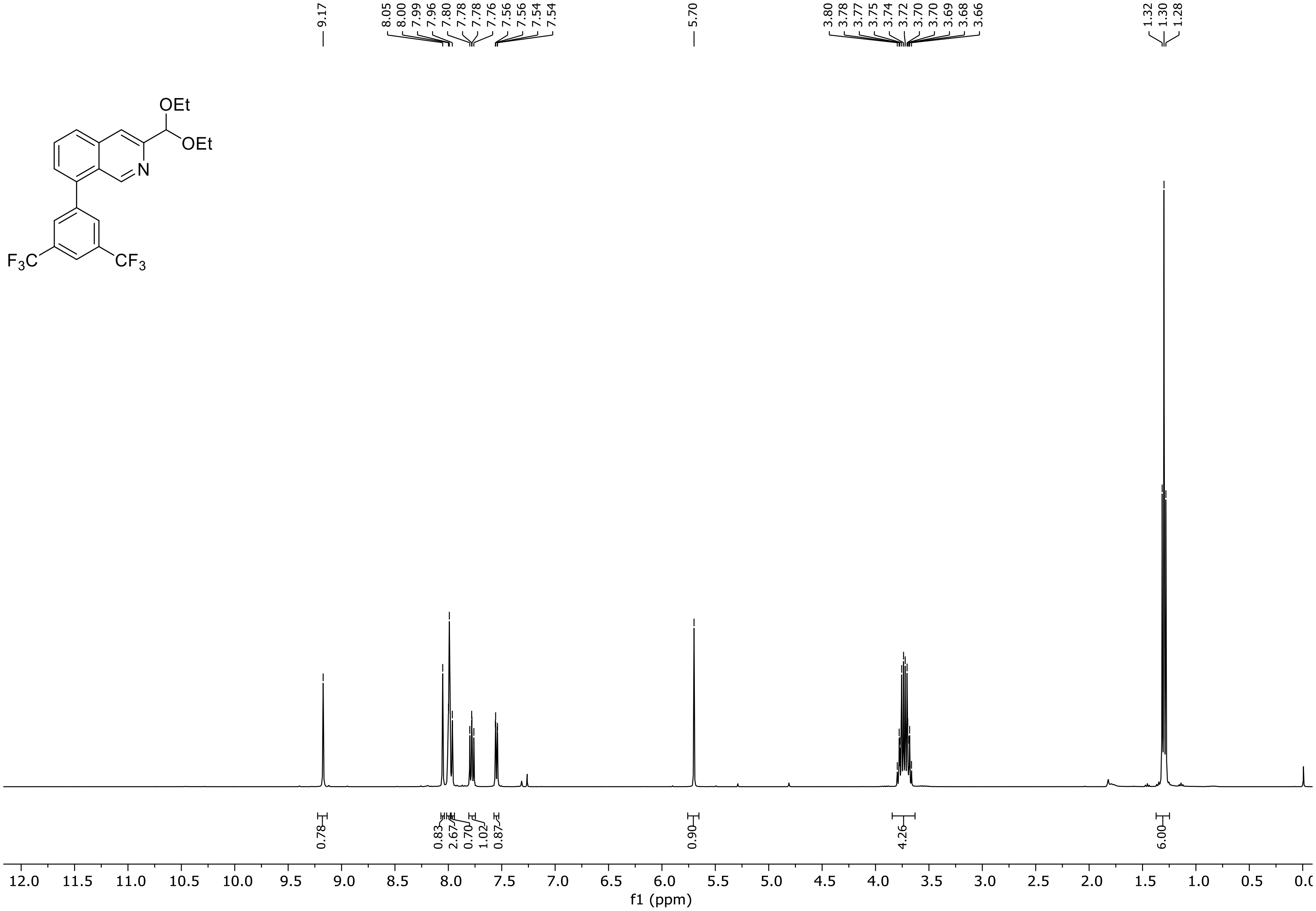

^{13}C-NMR spectrum of compound **8’d**: (100 MHz, $CDCl_3$)

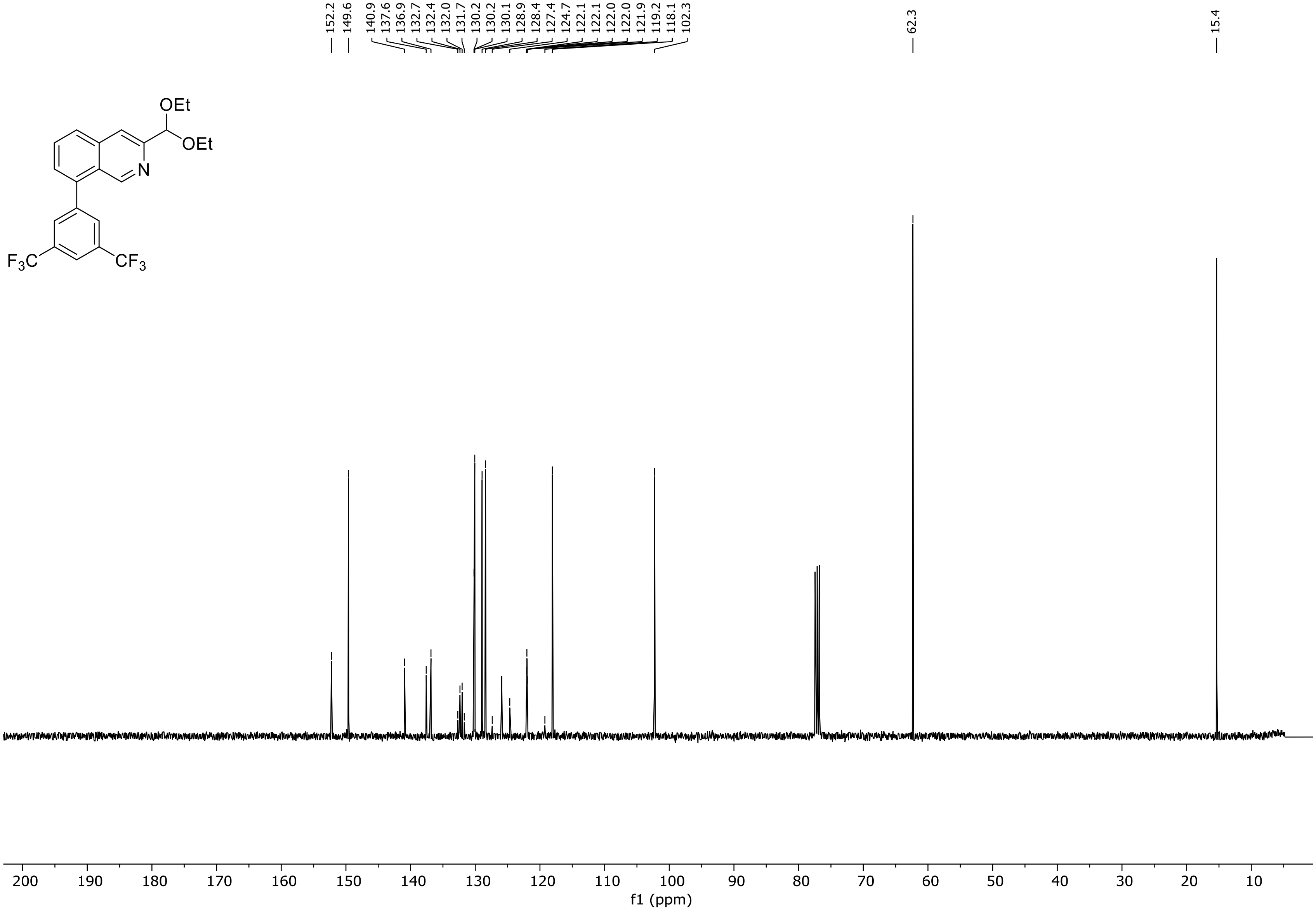

^{1}H-NMR spectrum of compound **8d**: (400 MHz, $CDCl_3$)

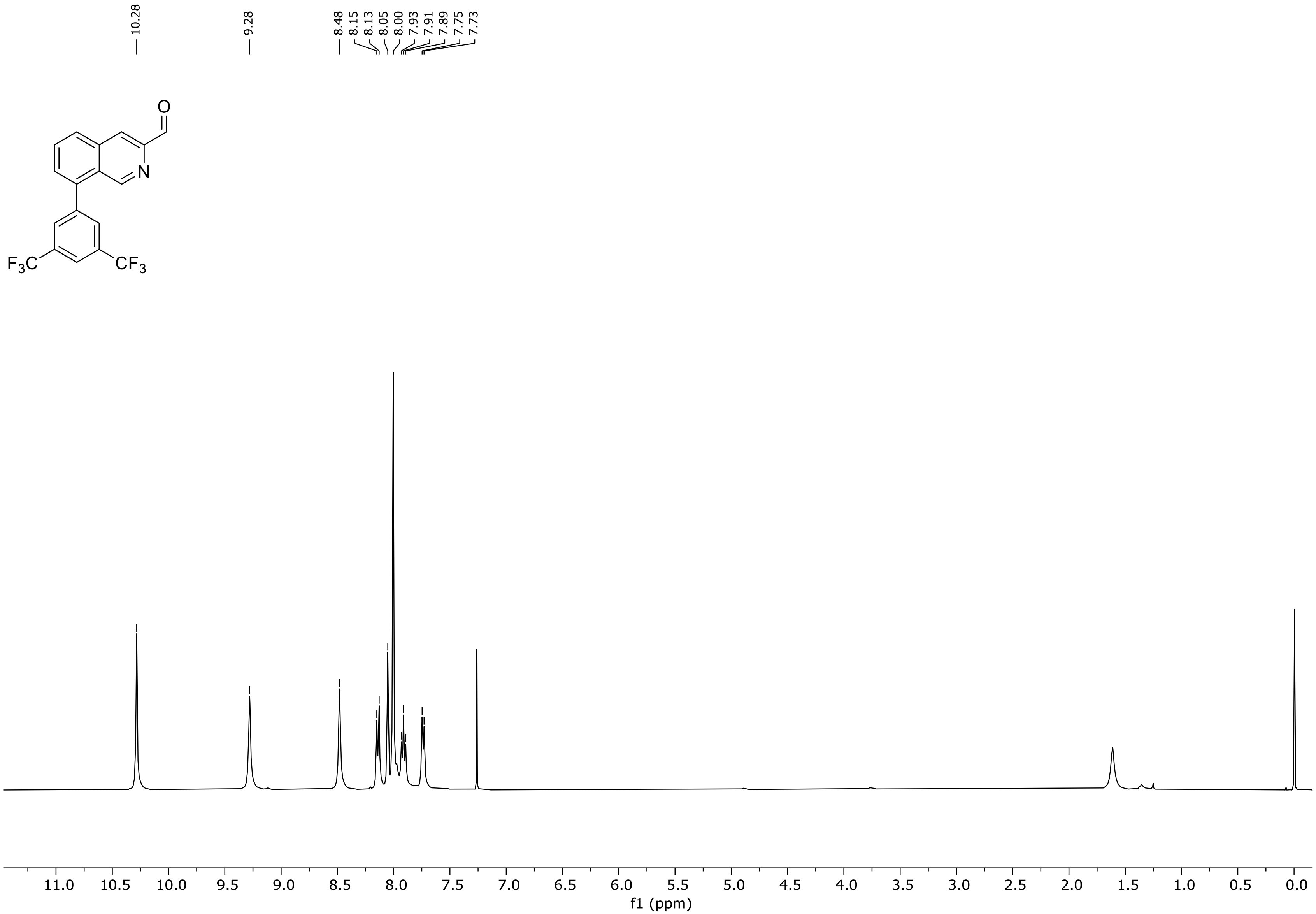

^{13}C-NMR spectrum of compound **8d**: (100 MHz, $CDCl_3$)

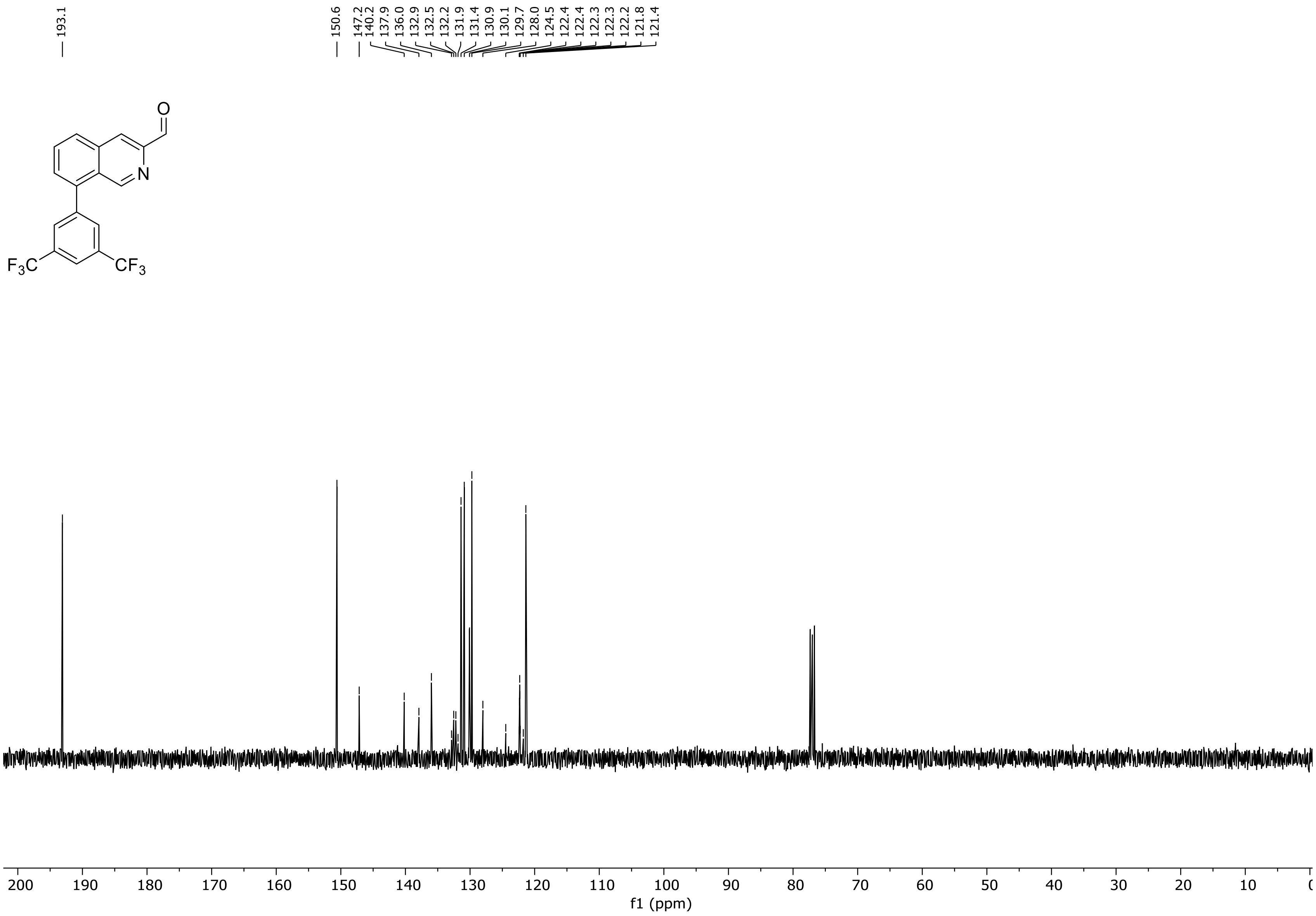

^{19}F-NMR spectrum of compound **8d**: (376 MHz, $CDCl_3$)

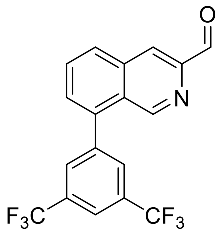

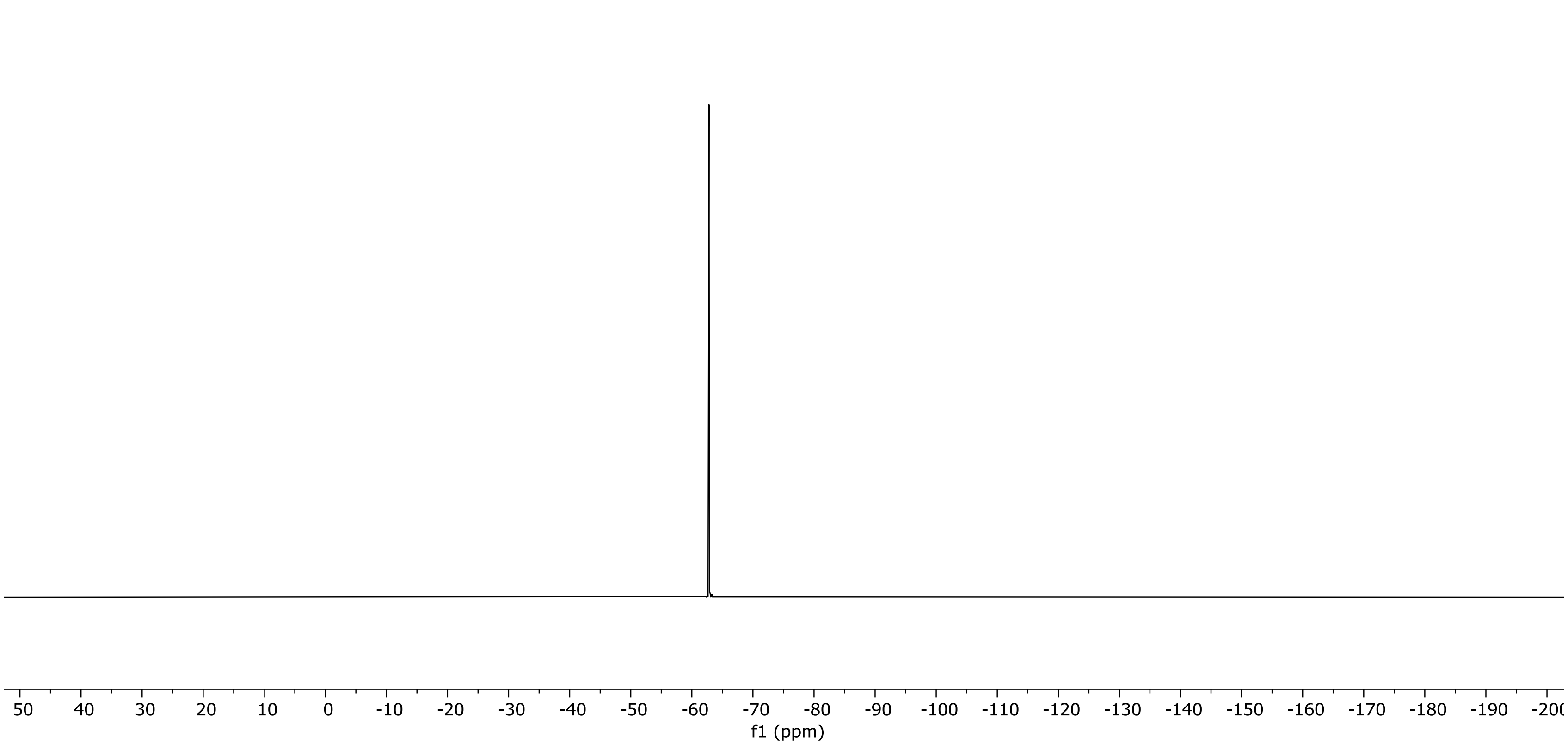

^{1}H-NMR spectrum of compound **8’e**: (500 MHz, $CDCl_3$)

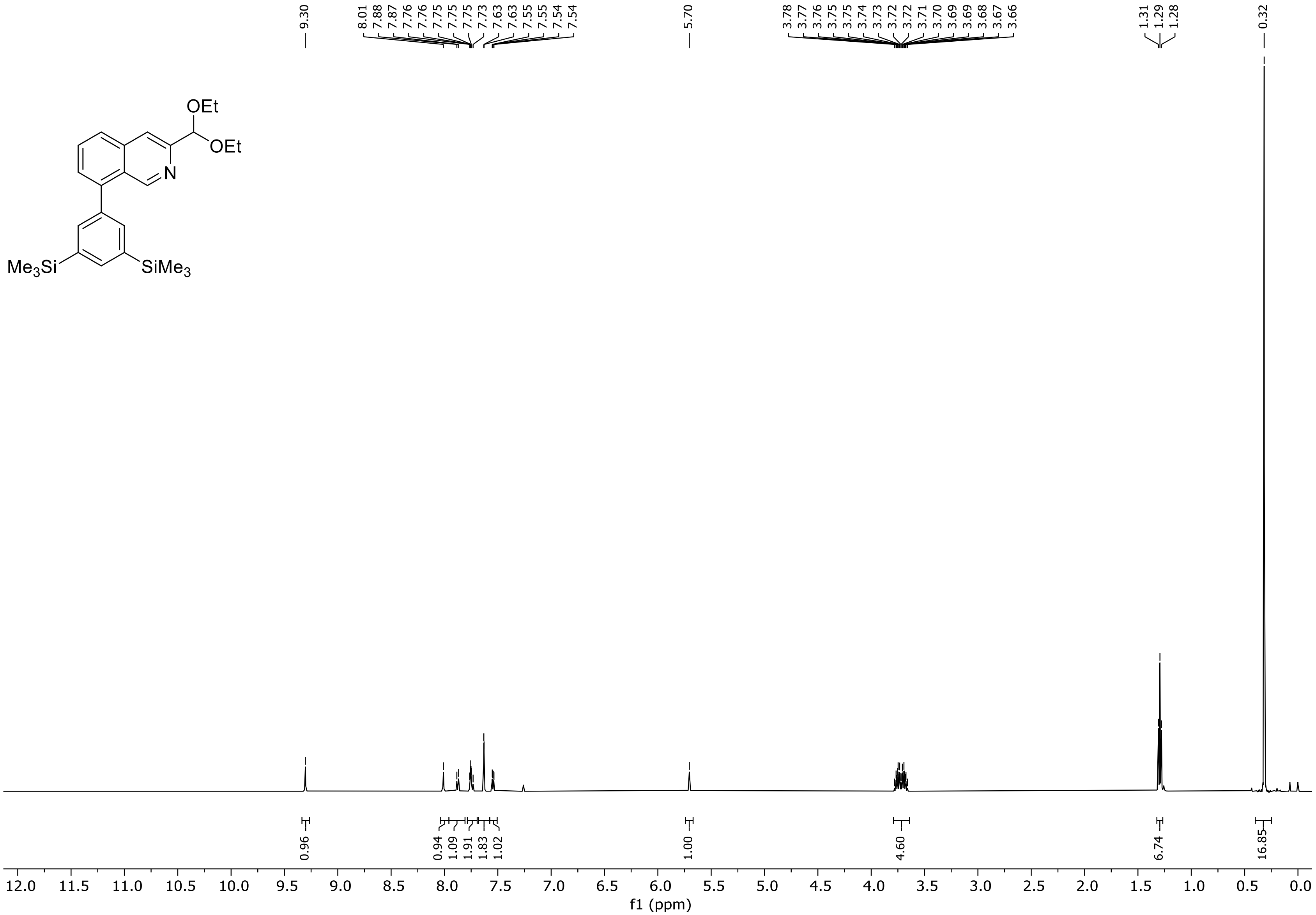

^{13}C-NMR spectrum of compound **8’e**: (126 MHz, $CDCl_3$)

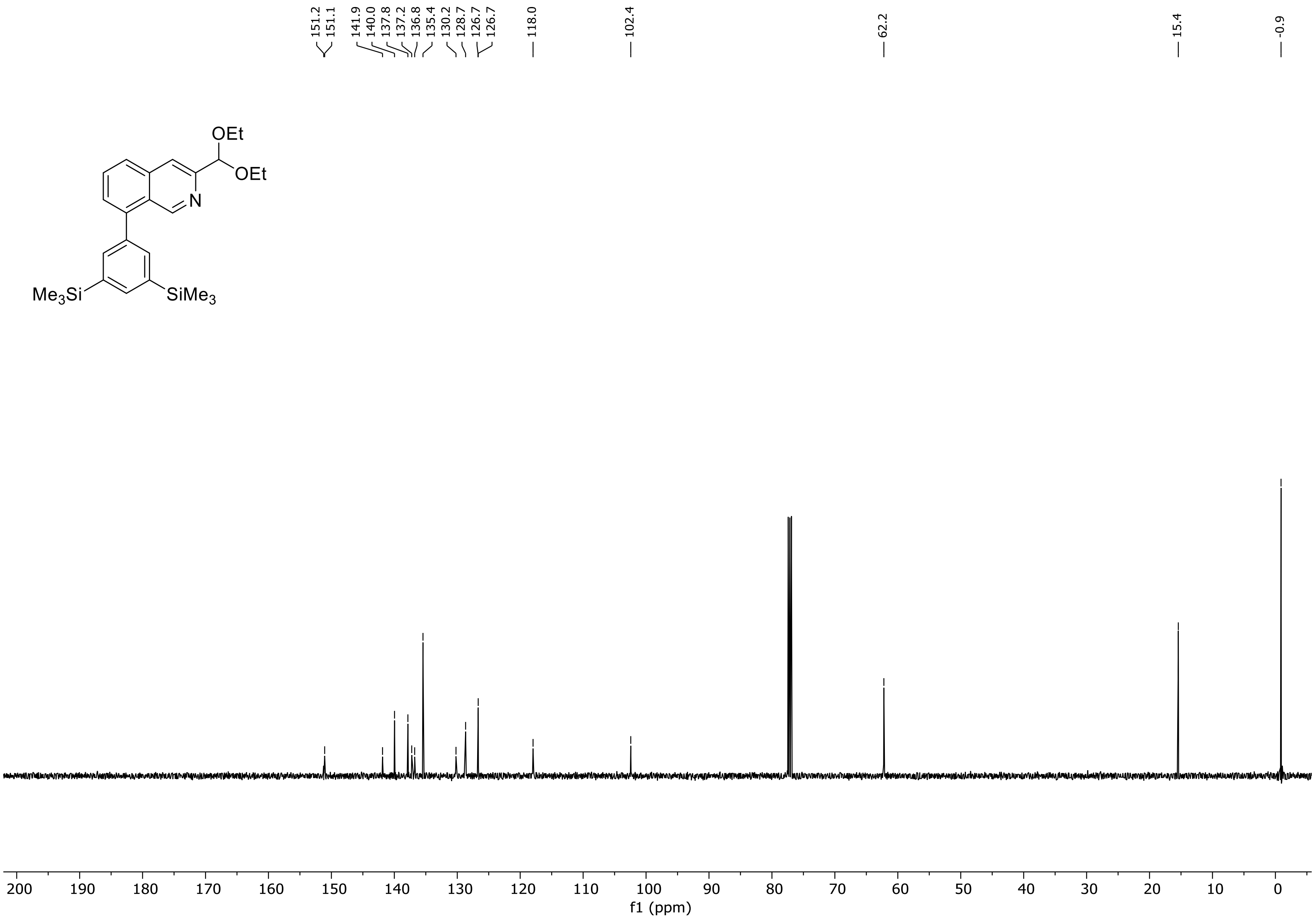

^{1}H-NMR spectrum of compound **8e**: (400 MHz, $CDCl_3$)

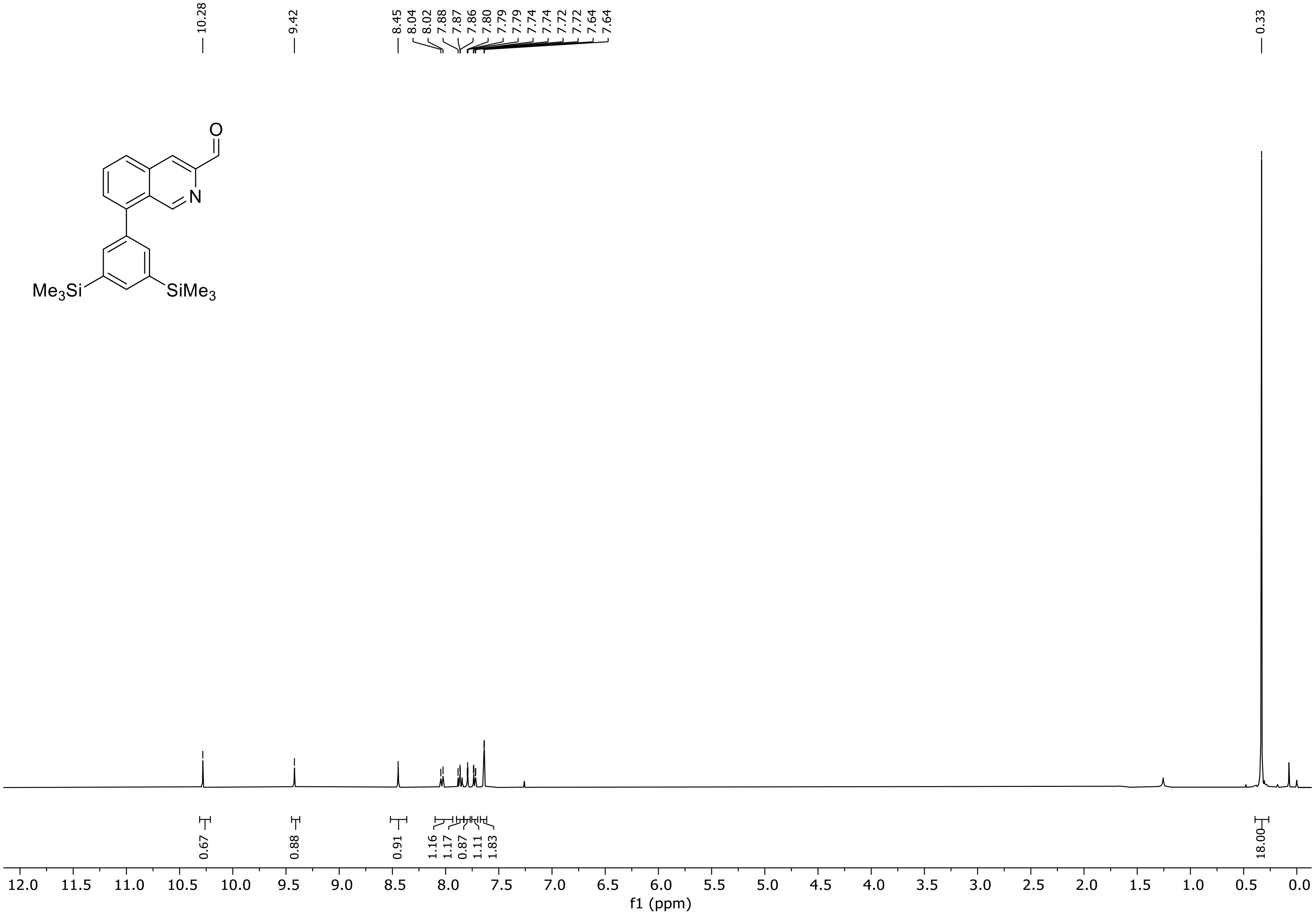

^{13}C-NMR spectrum of compound **8e**: (100 MHz, $CDCl_3$)

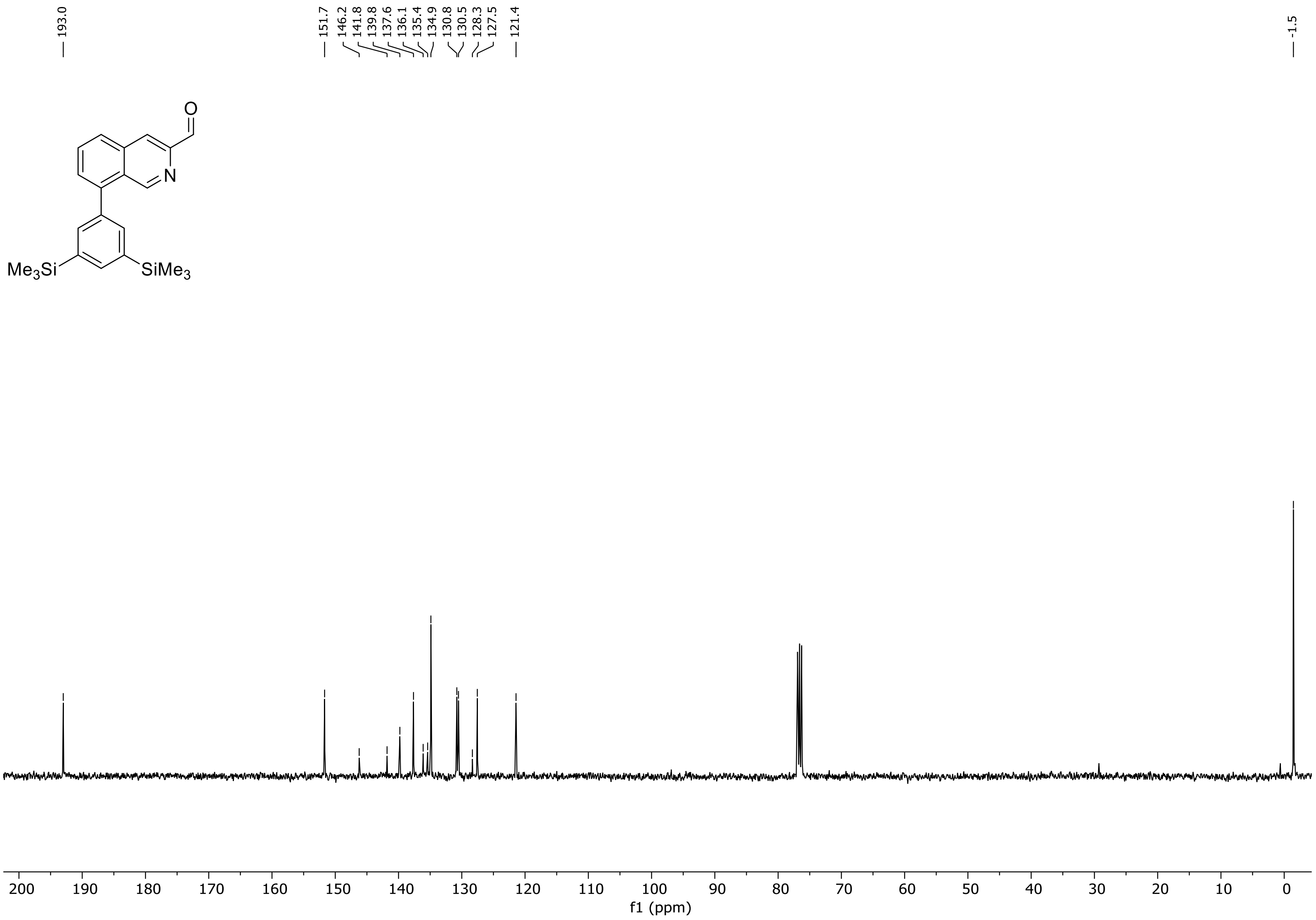

^{1}H-NMR spectrum of compound **8’f**: (500 MHz, $CDCl_3$)

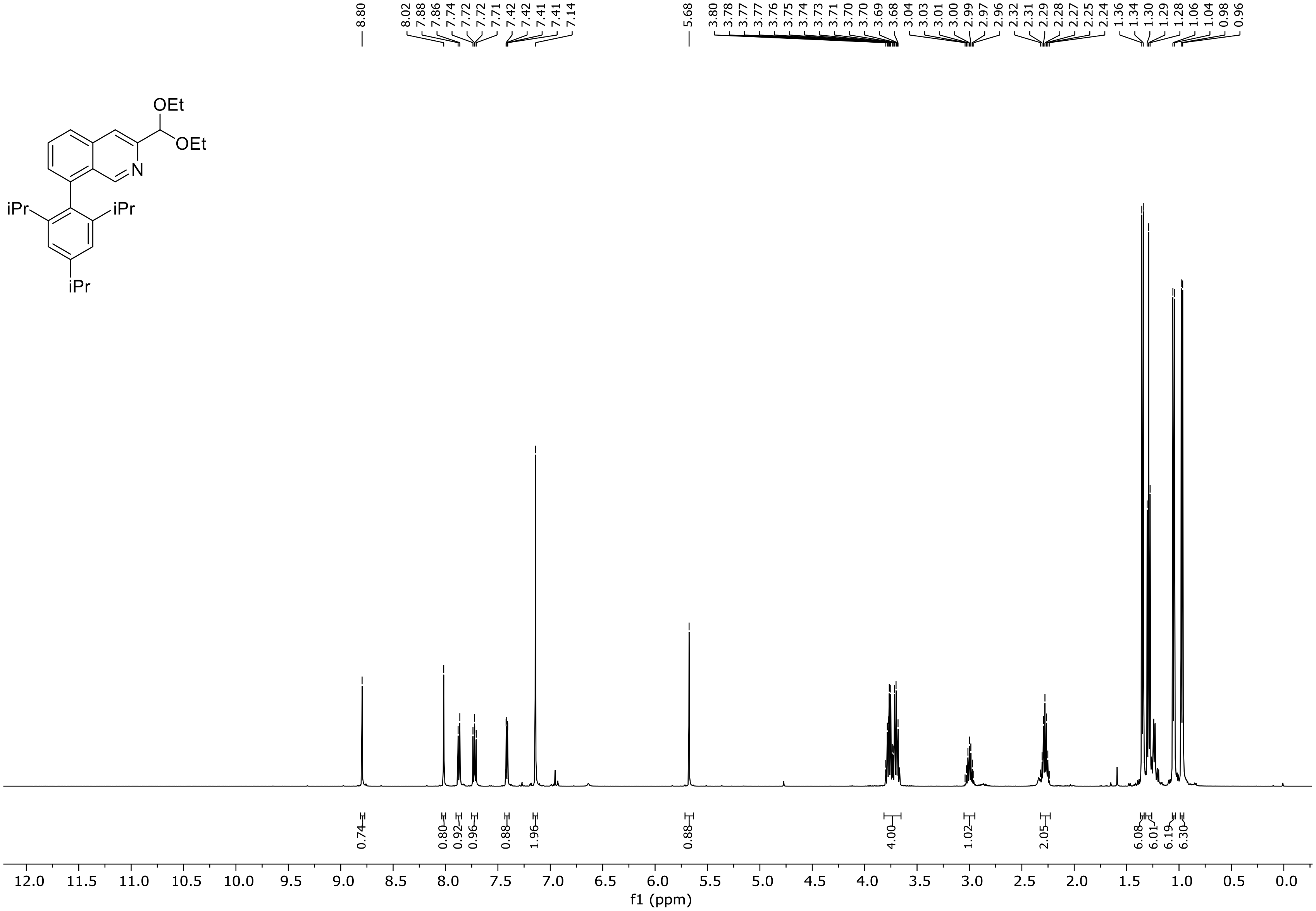

^{13}C-NMR spectrum of compound **8’f**: (125 MHz, $CDCl_3$)

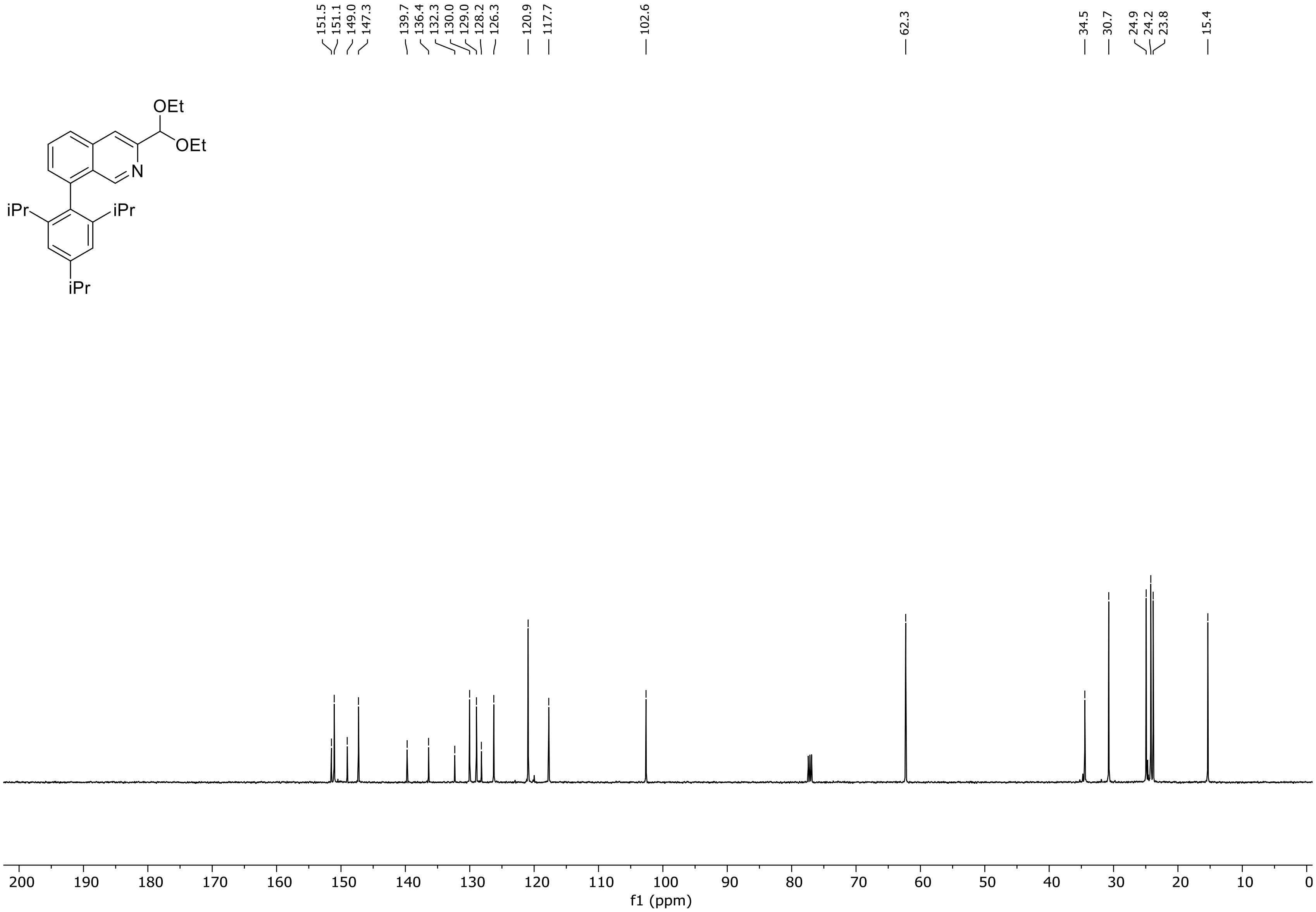

^{1}H-NMR spectrum of compound **8f**: (400 MHz, $CDCl_3$)

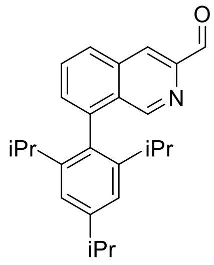

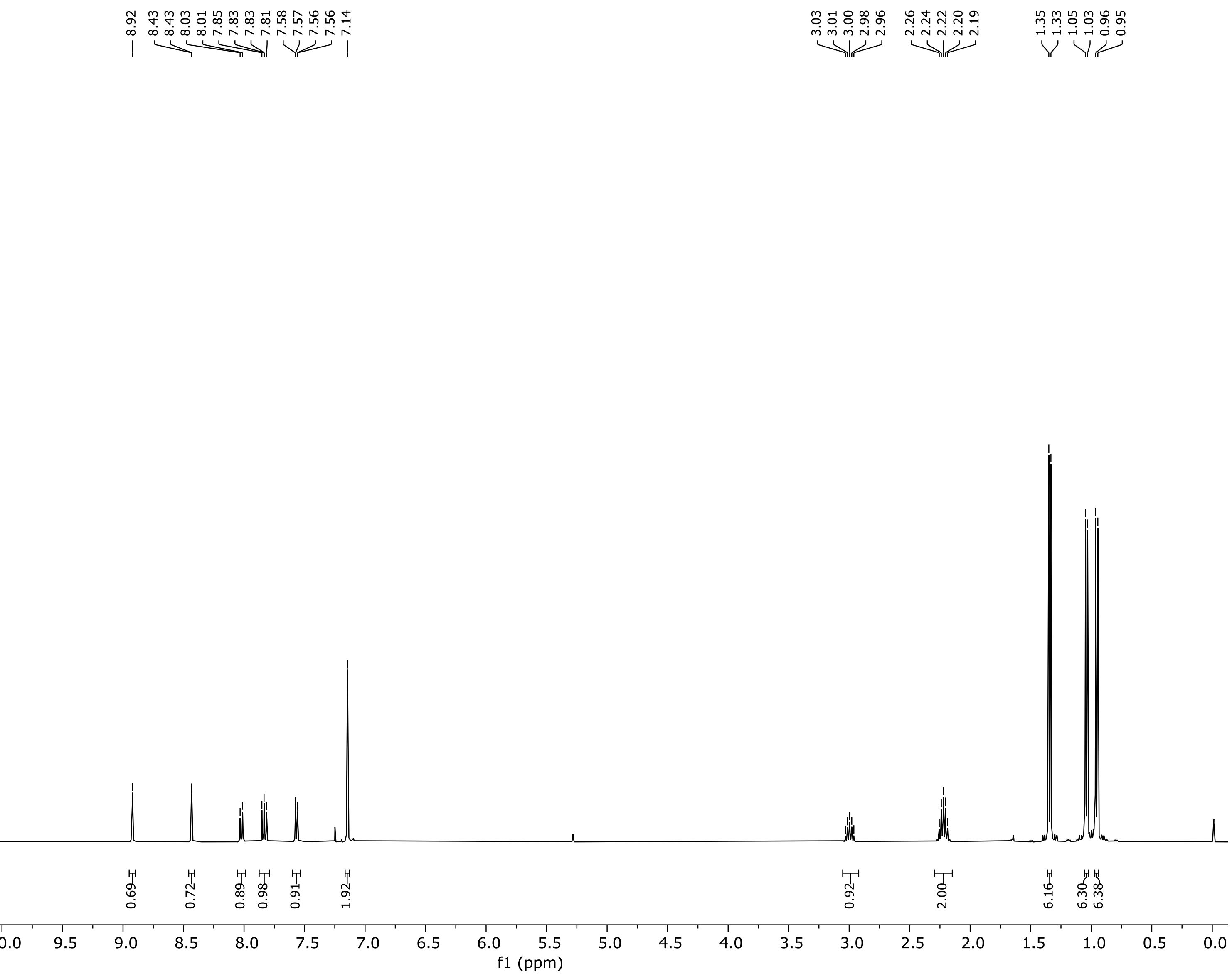

^{13}C-NMR spectrum of compound **8f**: (100 MHz, $CDCl_3$)

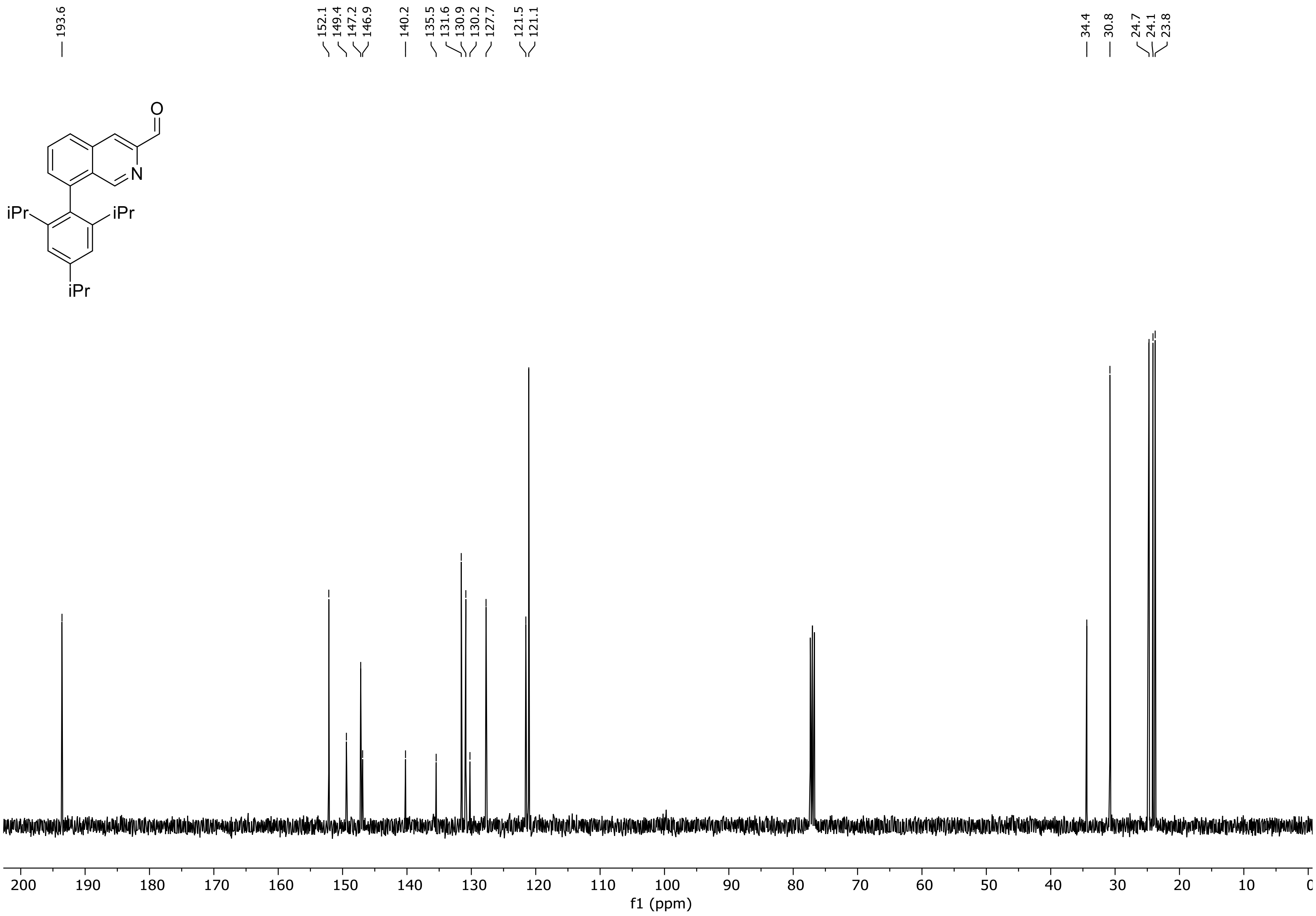

^{1}H-NMR spectrum of compound **8’g**: (400 MHz, $CDCl_3$)

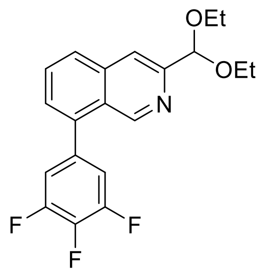

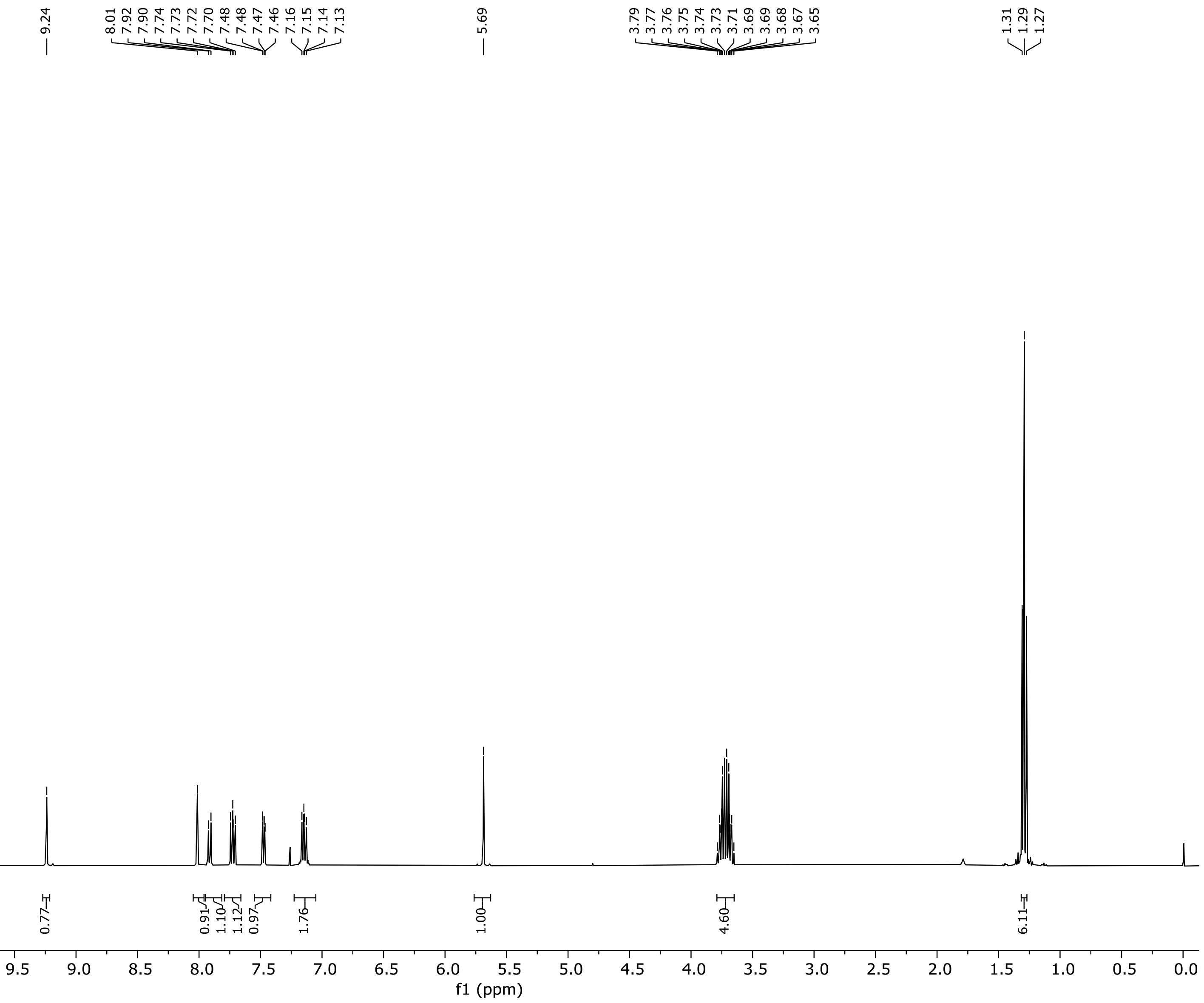

^{13}C-NMR spectrum of compound **8’g**: (100 MHz, $CDCl_3$)

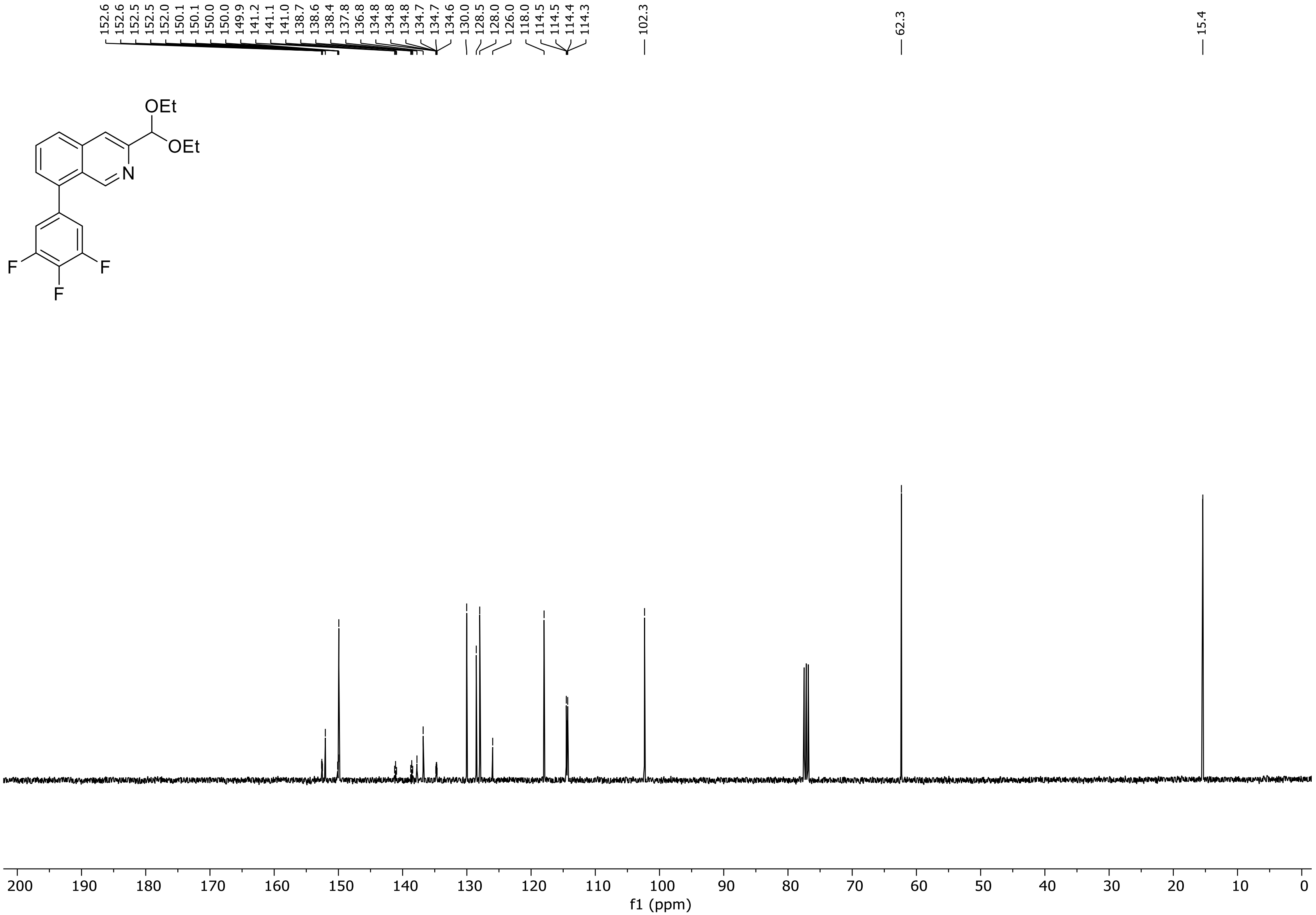

^{19}F-NMR spectrum of compound **8’g**: (376 MHz, $CDCl_3$)

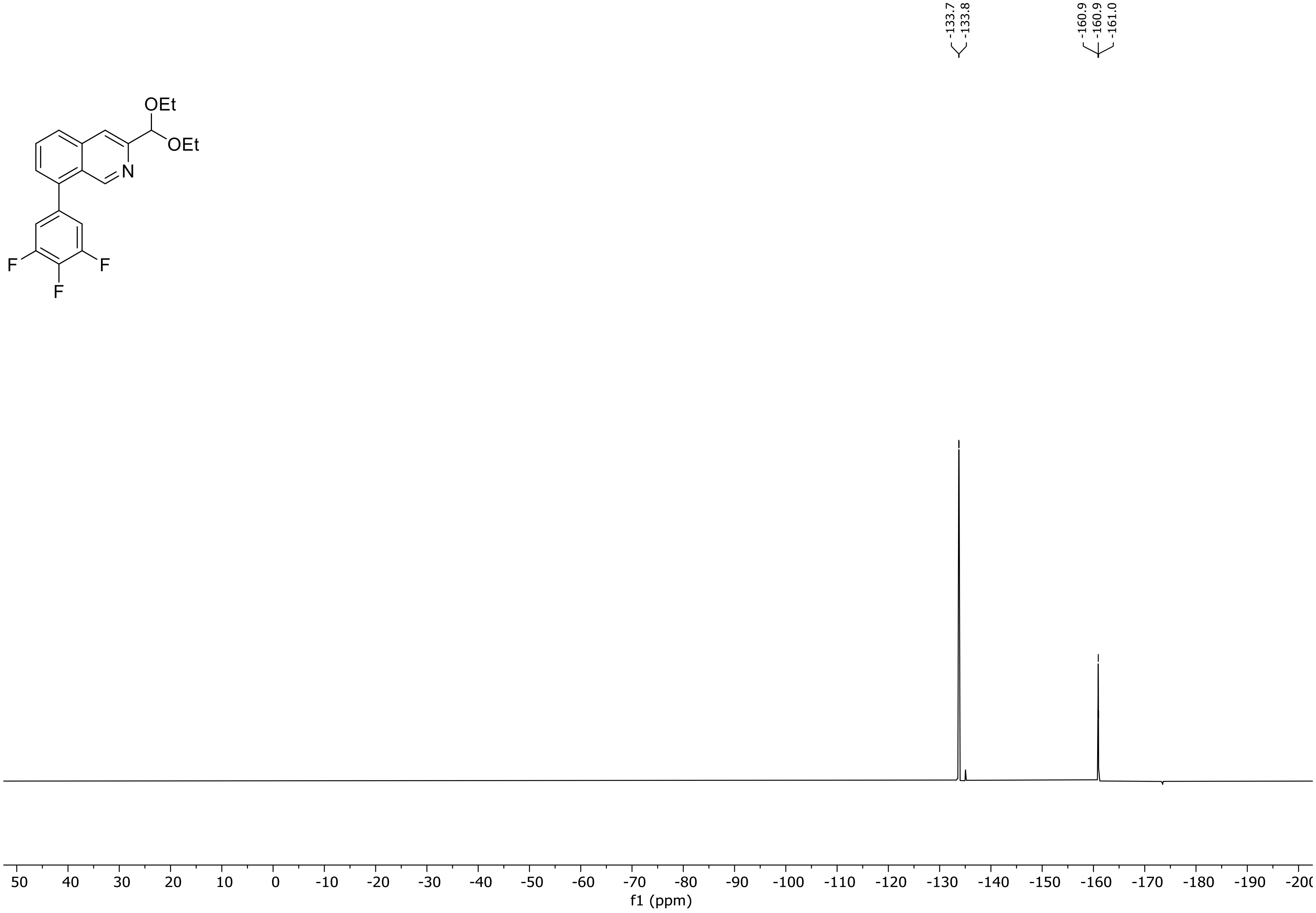

^{1}H-NMR spectrum of compound **8g**: (400 MHz, $CDCl_3$)

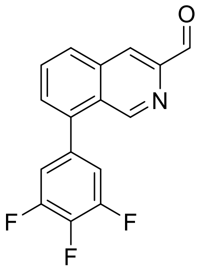

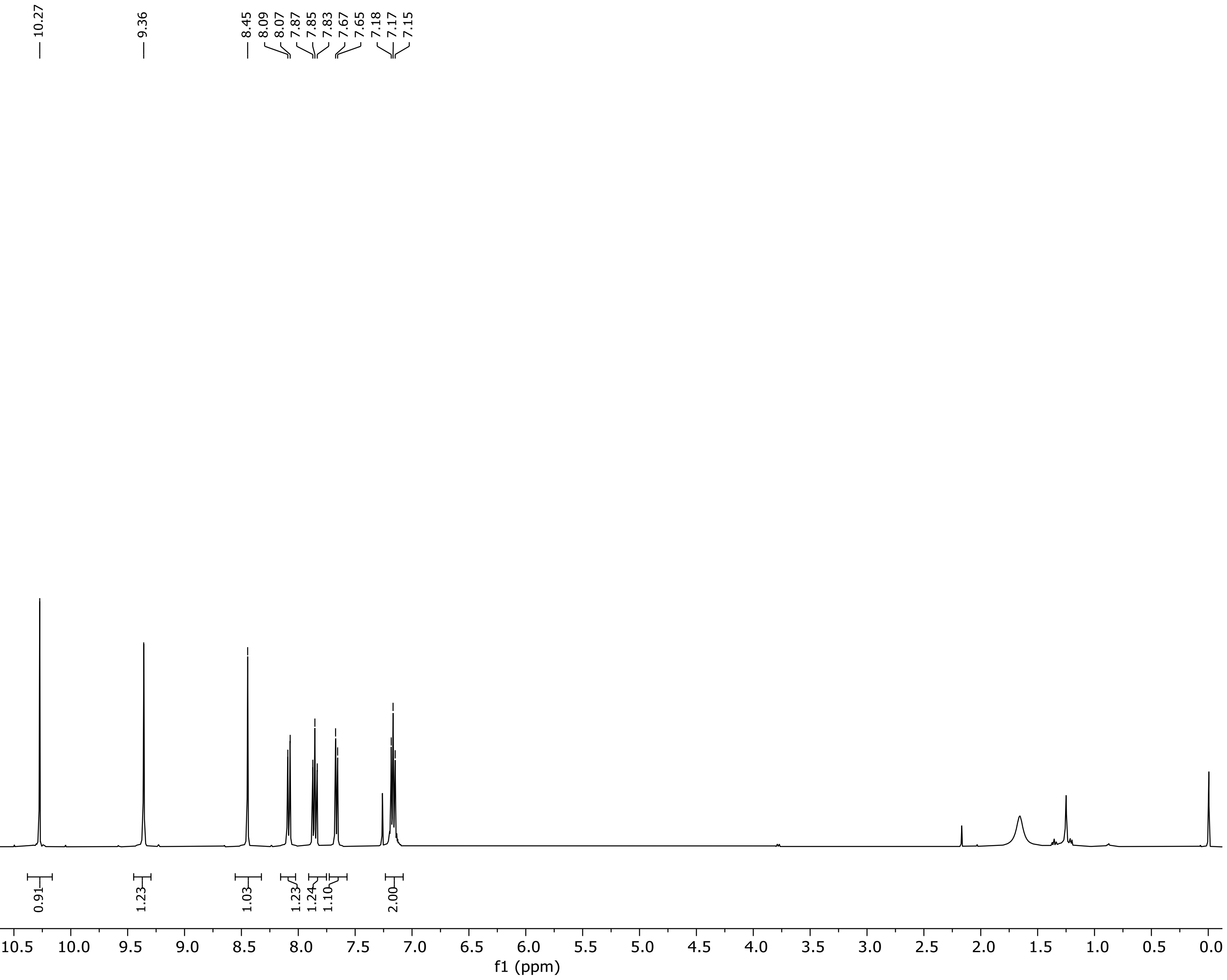

^{13}C-NMR spectrum of compound **8g**: (100 MHz, $CDCl_3$)

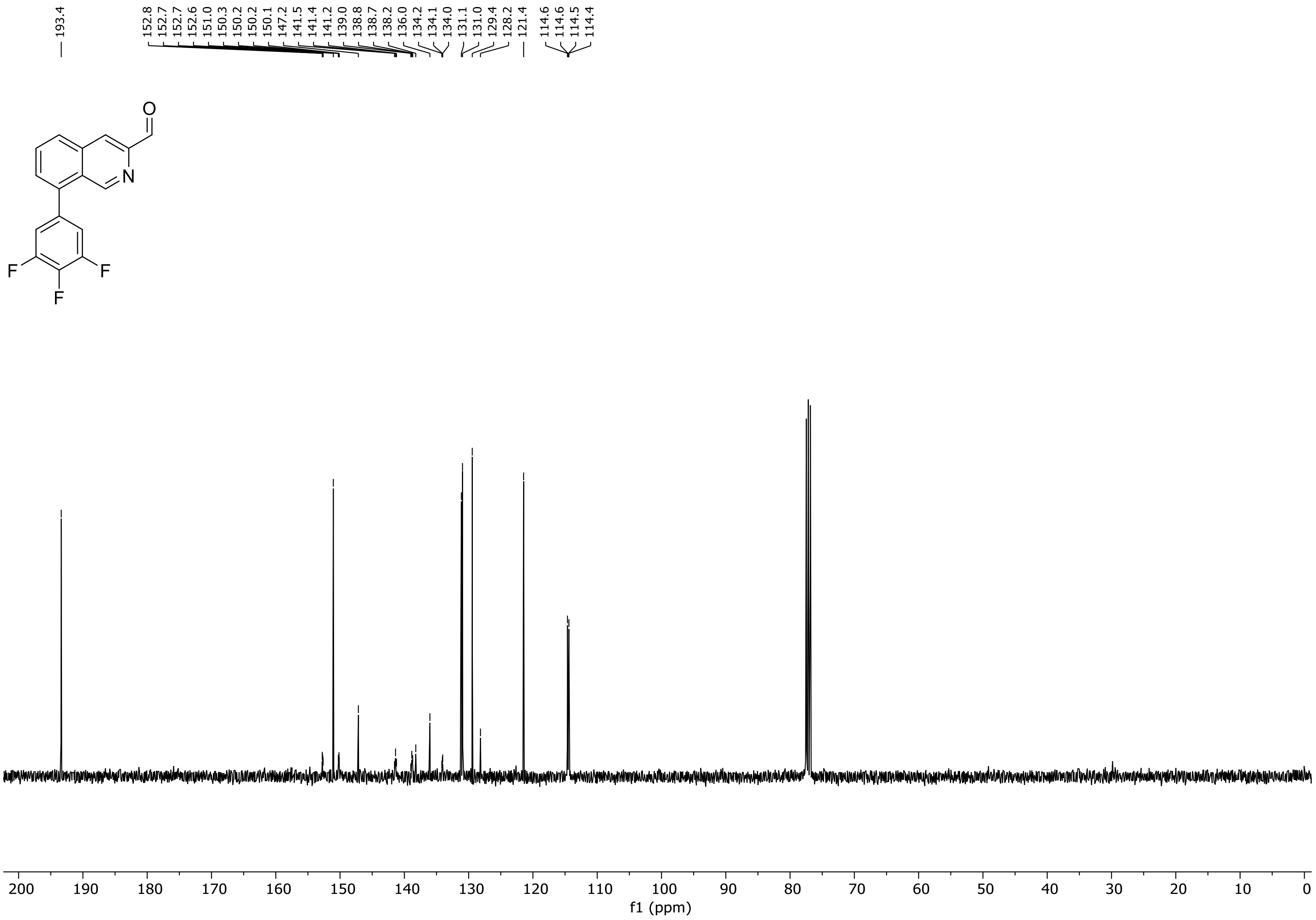

^{19}F-NMR spectrum of compound **8g**: (376 MHz, $CDCl_3$)

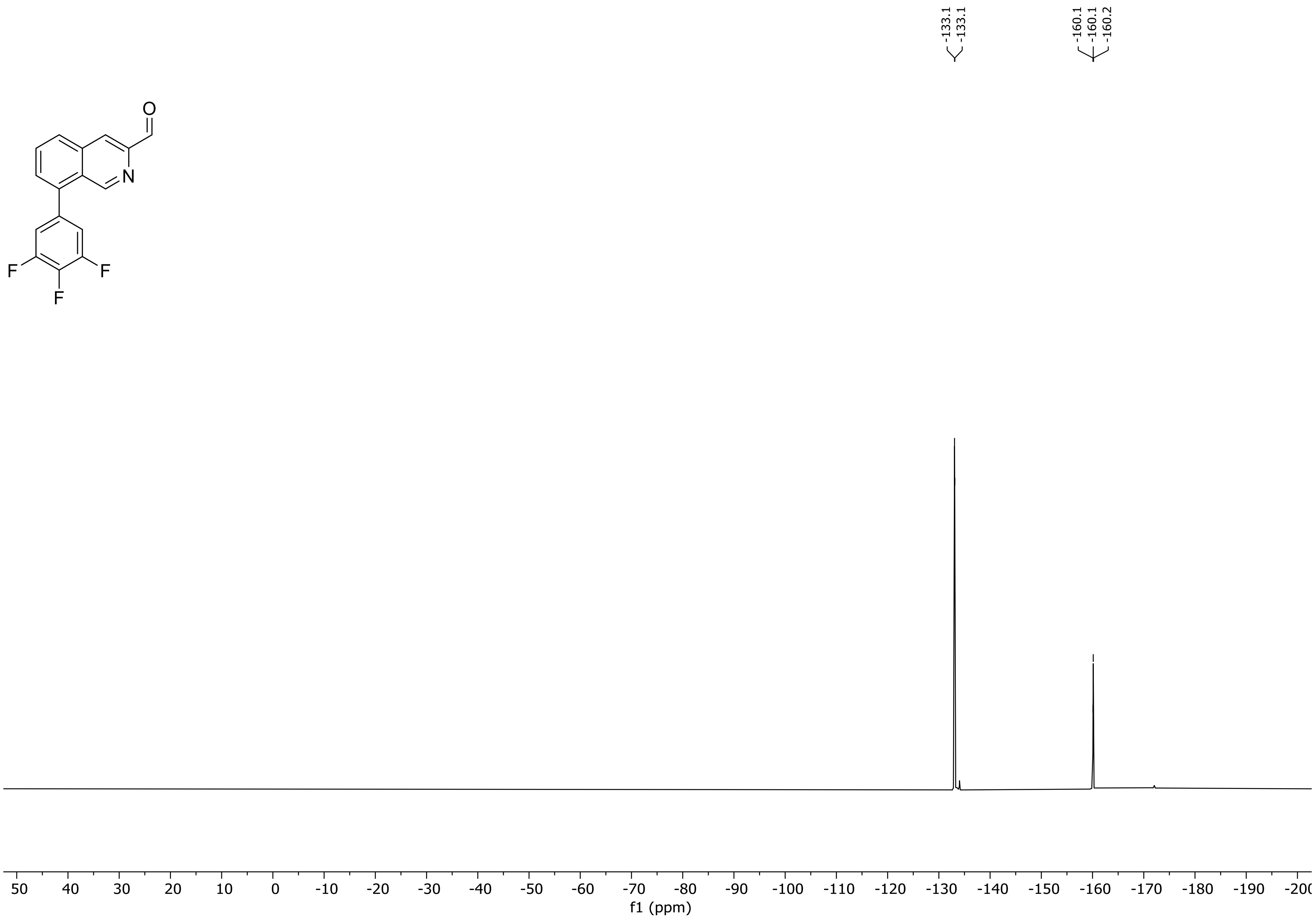

^{1}H-NMR spectrum of compound **8'h**: (400 MHz, $CDCl_3$)

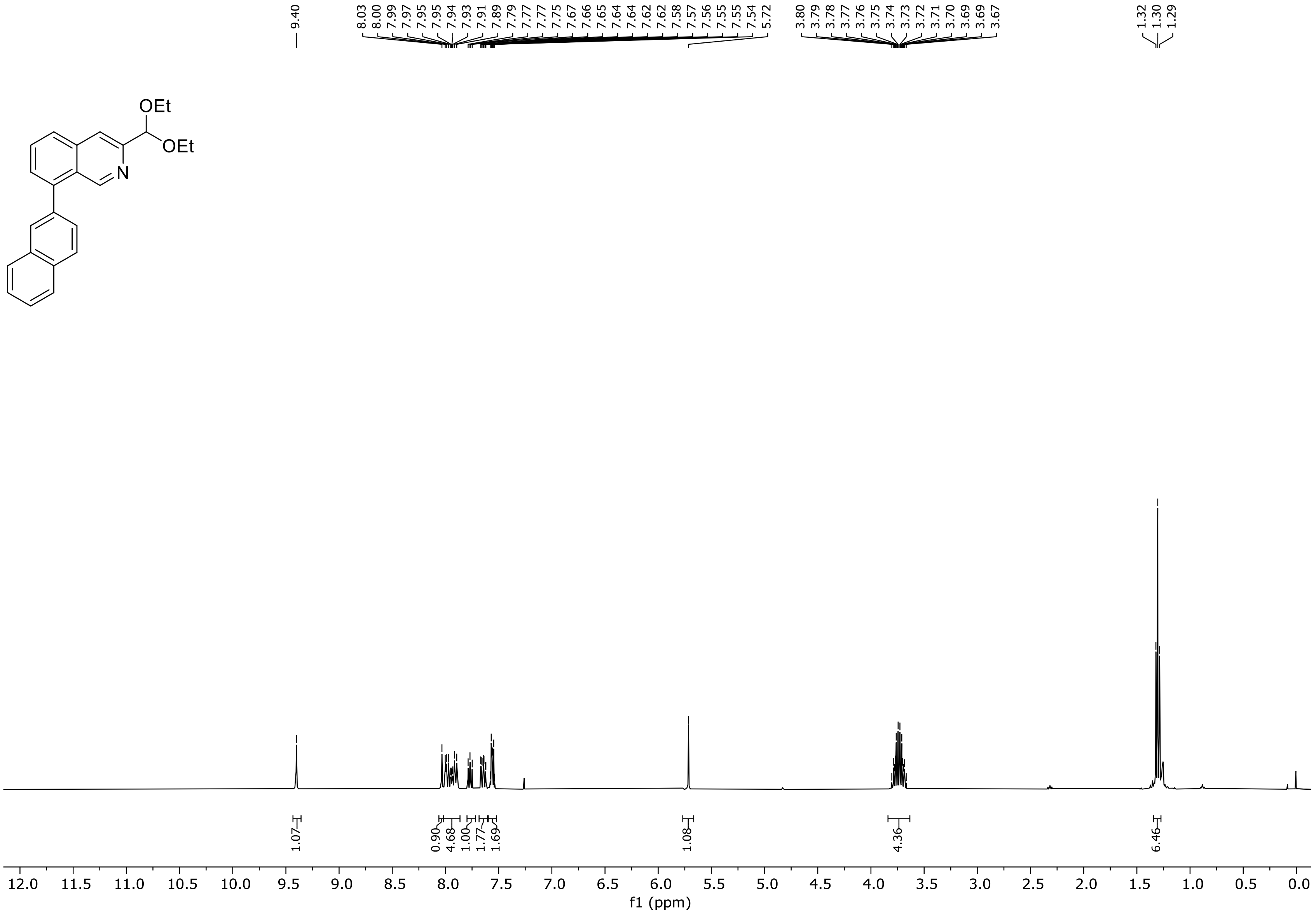

^{13}C-NMR spectrum of compound **8'h**: (100 MHz, $CDCl_3$)

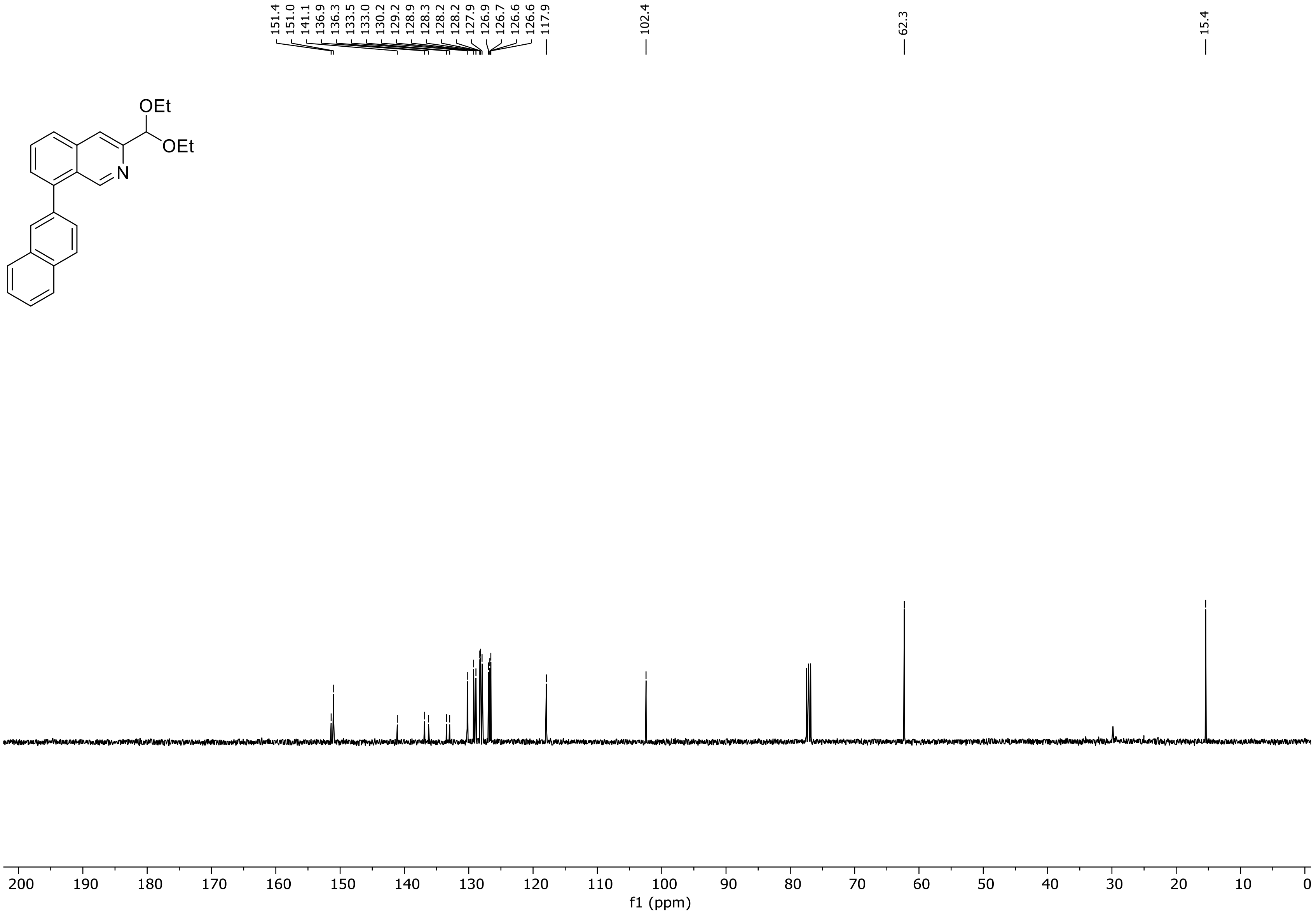

^{1}H-NMR spectrum of compound **8h**: (400 MHz, $CDCl_3$)

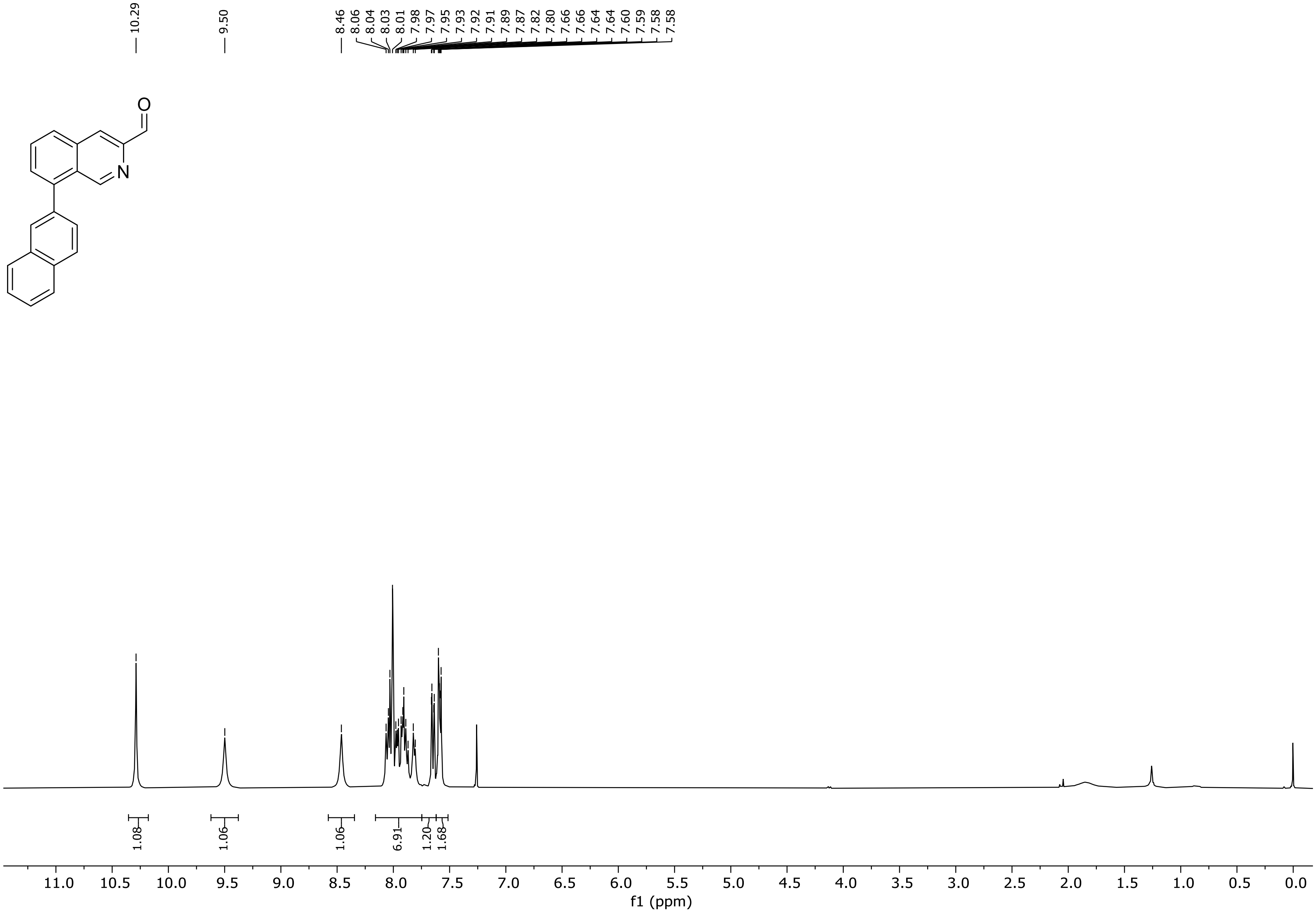

^{13}C-NMR spectrum of compound **8h**: (100 MHz, $CDCl_3$)

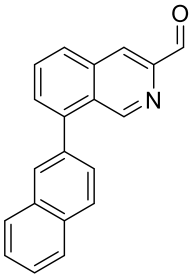

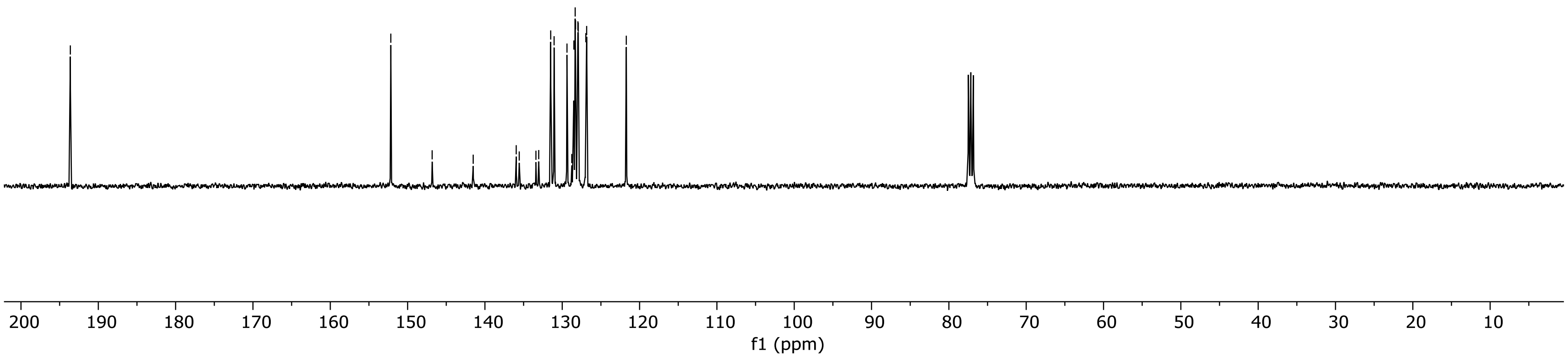

^{1}H-NMR spectrum of compound **8'i**: (400 MHz, $CDCl_3$)

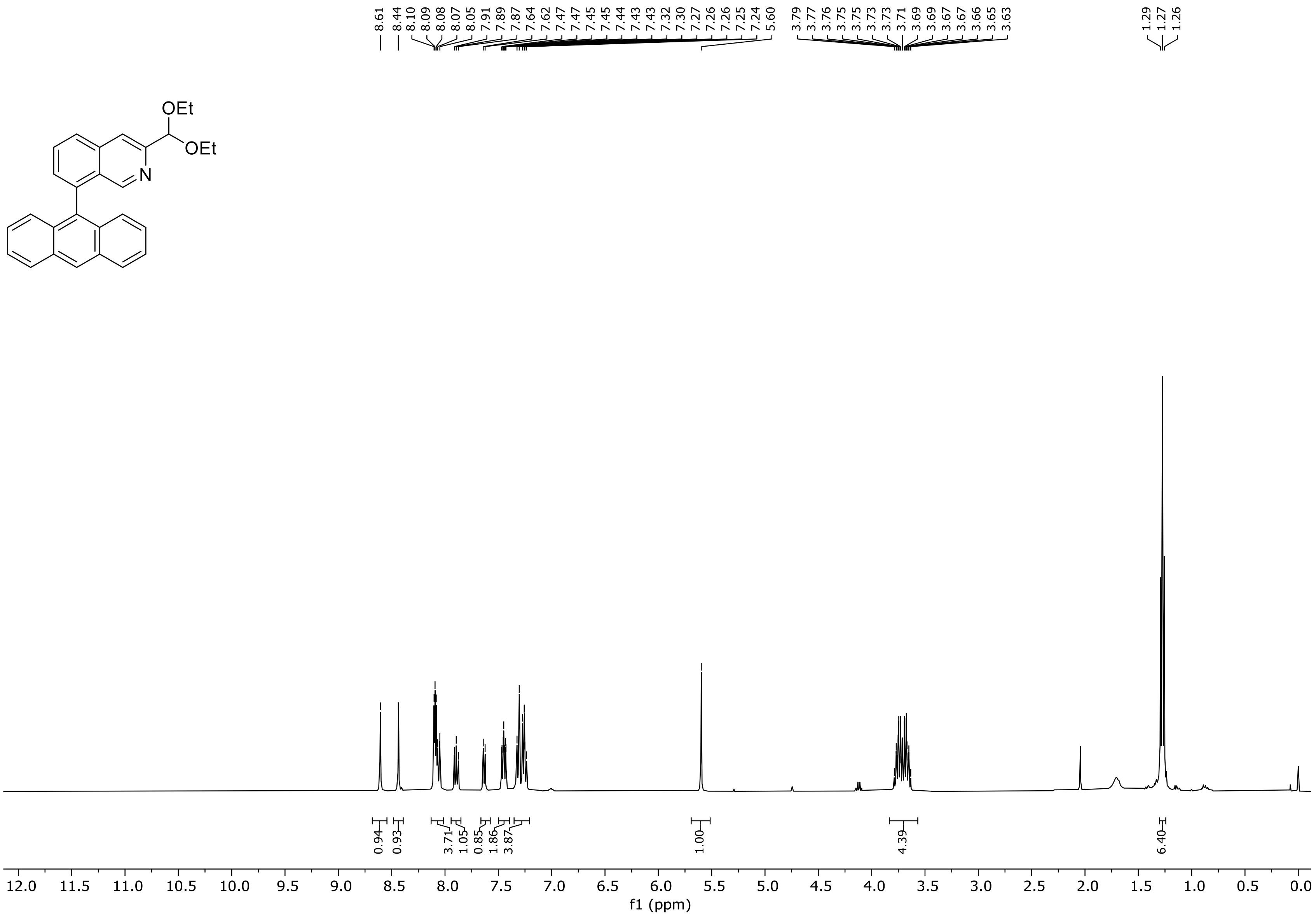

^{13}C-NMR spectrum of compound **8’i**: (100 MHz, $CDCl_3$)

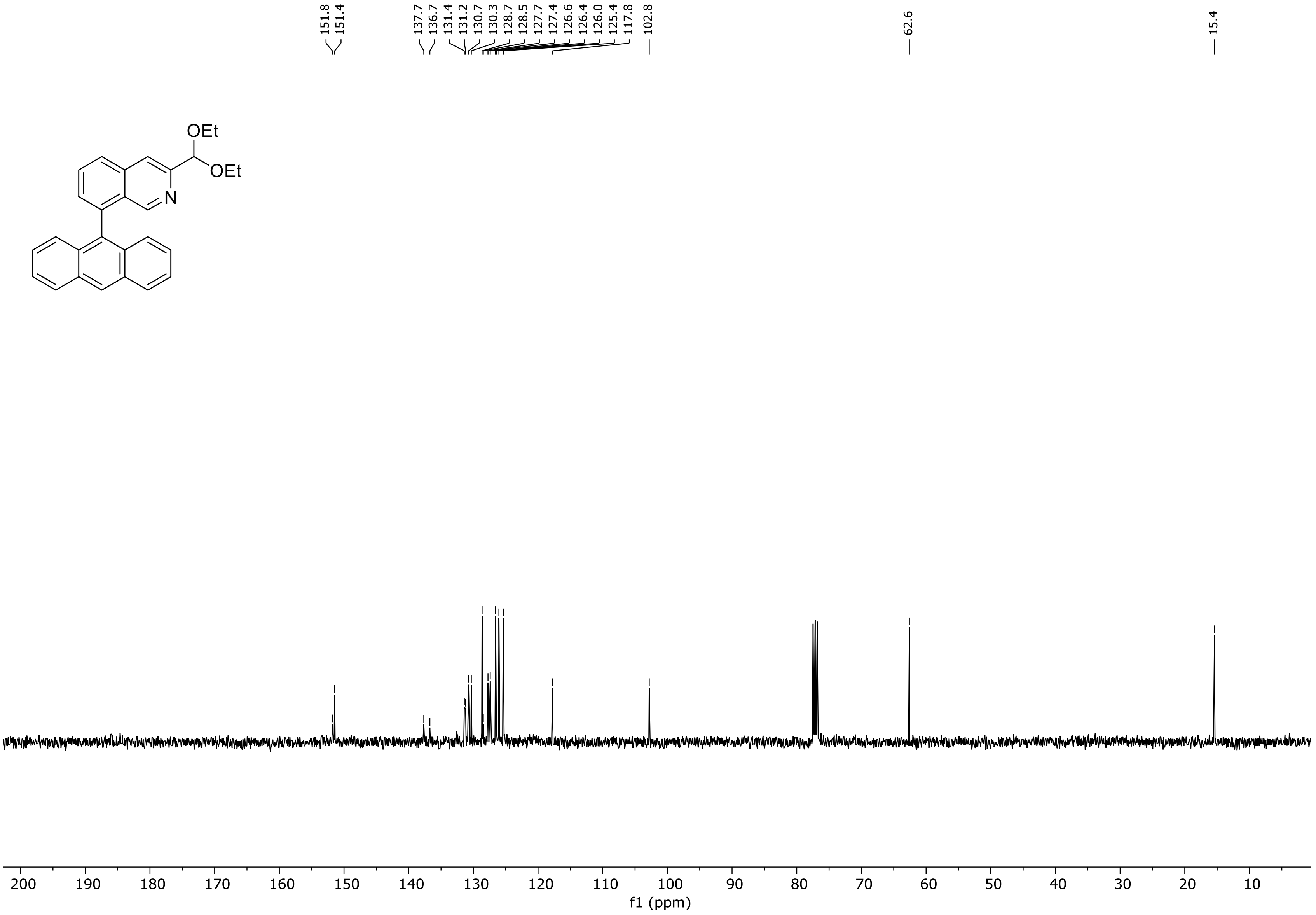

^{1}H-NMR spectrum of compound **8i**: (400 MHz, $CDCl_3$)

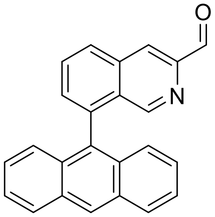

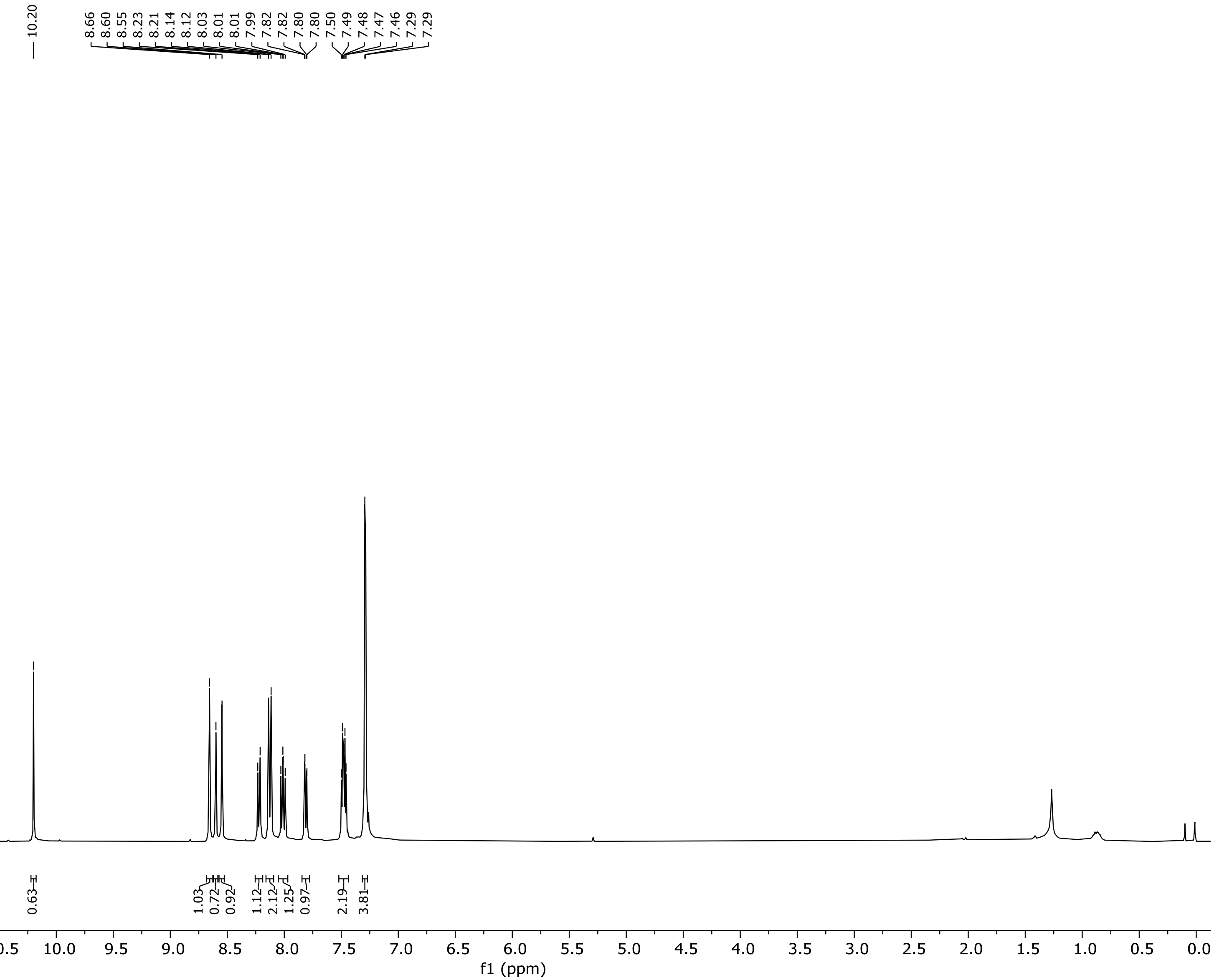

^{13}C-NMR spectrum of compound **8i**: (100 MHz, $CDCl_3$)

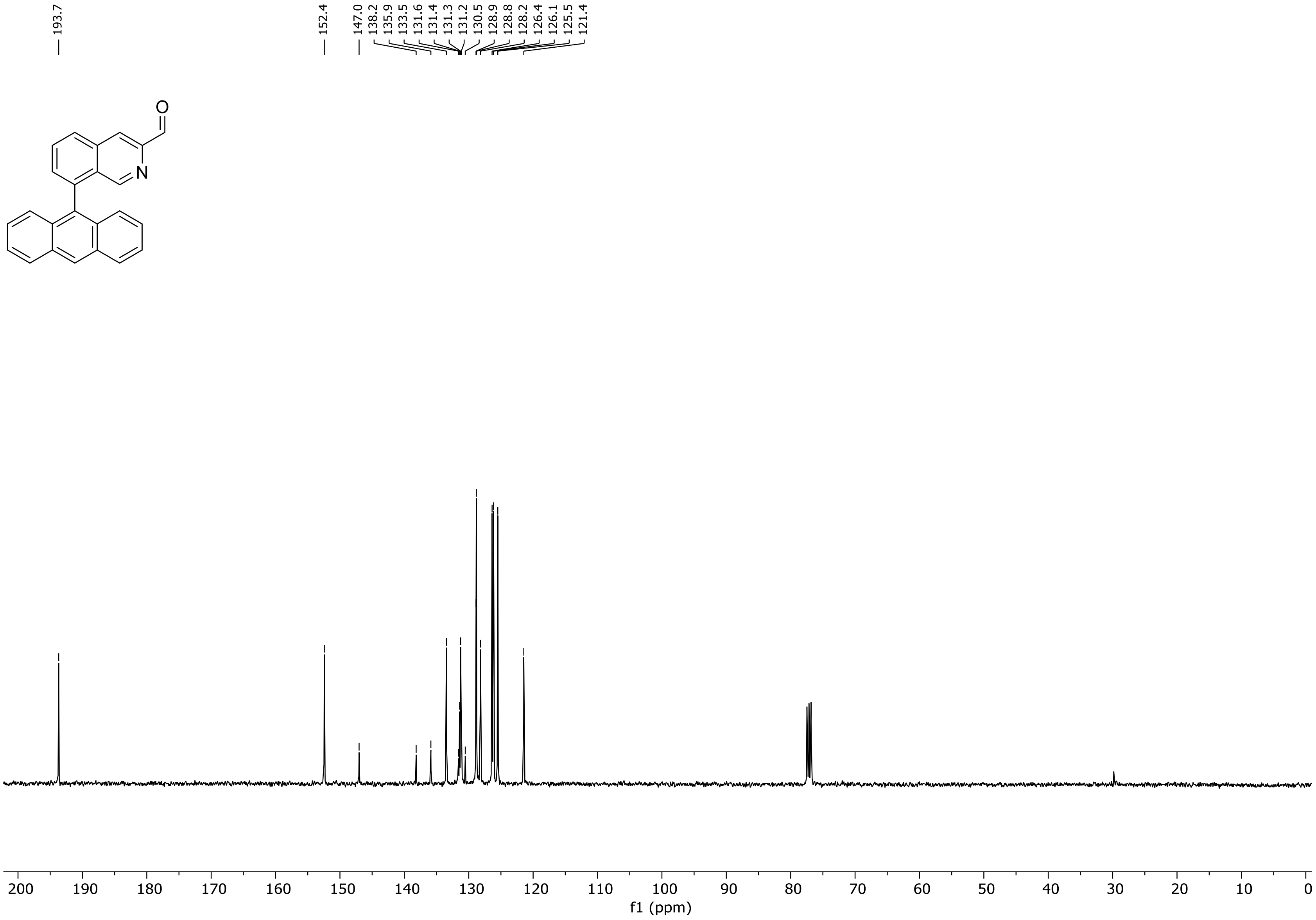

^{1}H-NMR spectrum of compound **12c**: (500 MHz, $CDCl_3$)

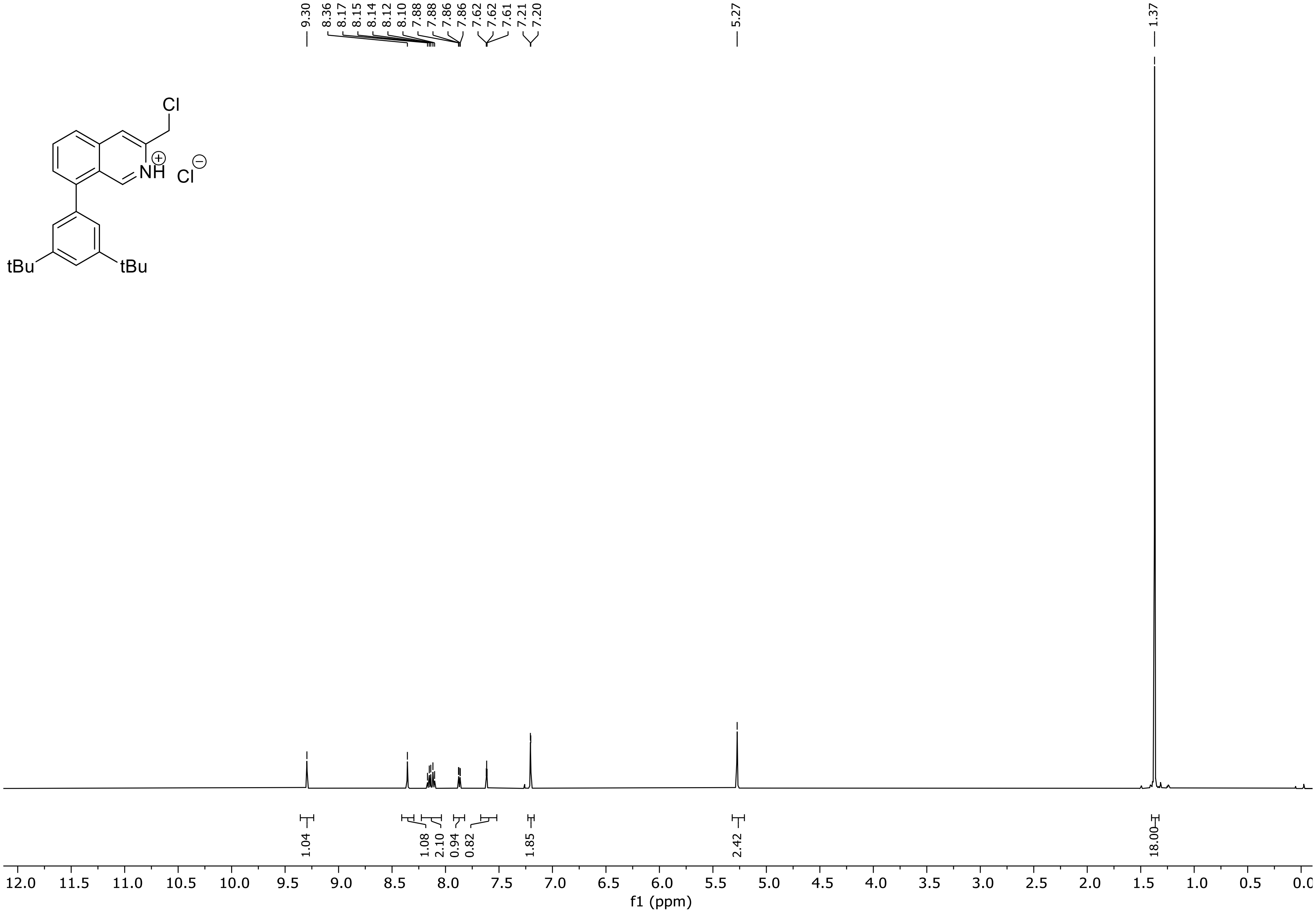

^{13}C-NMR spectrum of compound **12c**: (126 MHz, $CDCl_3$)

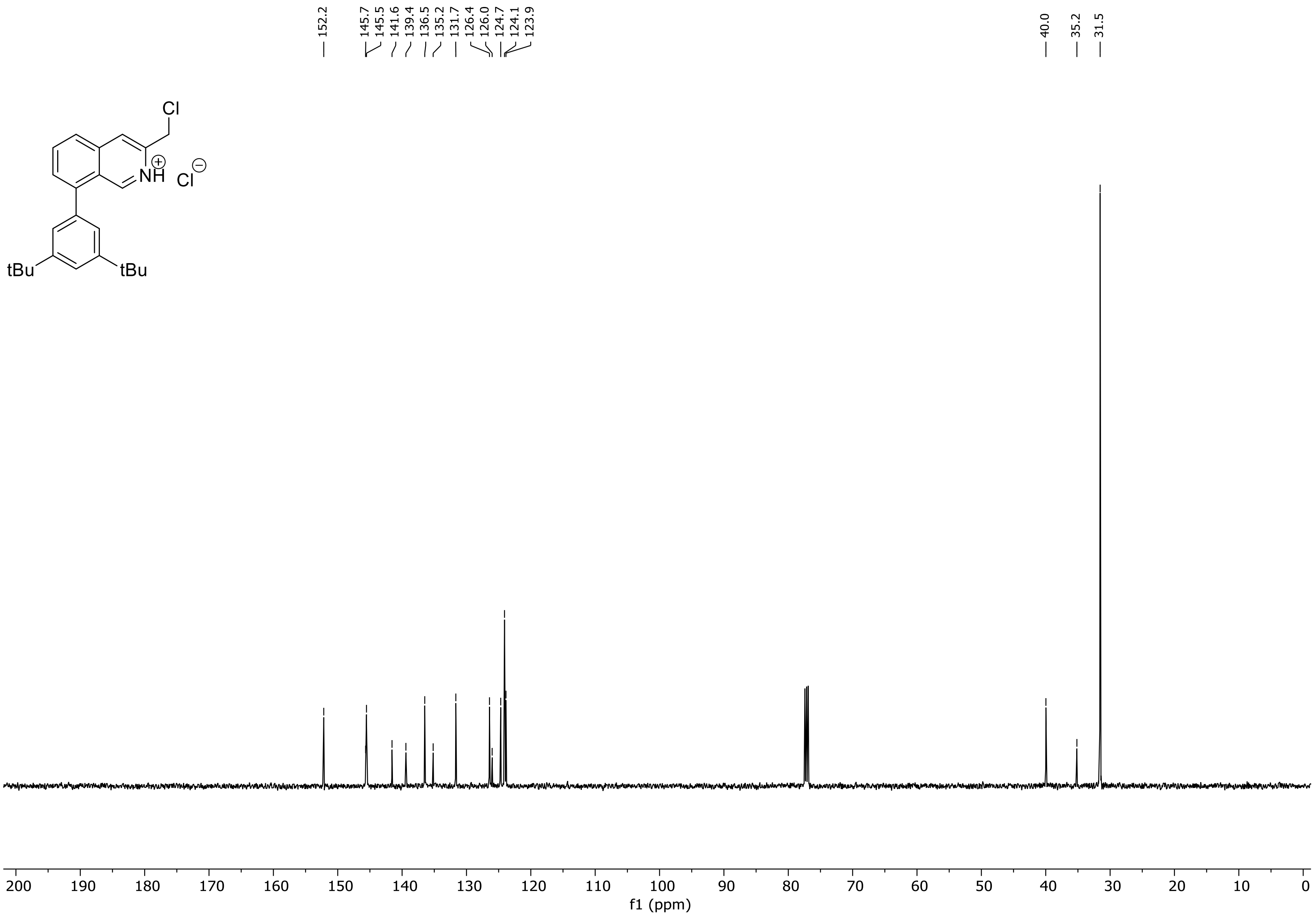

^{1}H-NMR spectrum of compound **12d**: (400 MHz, DMSO)

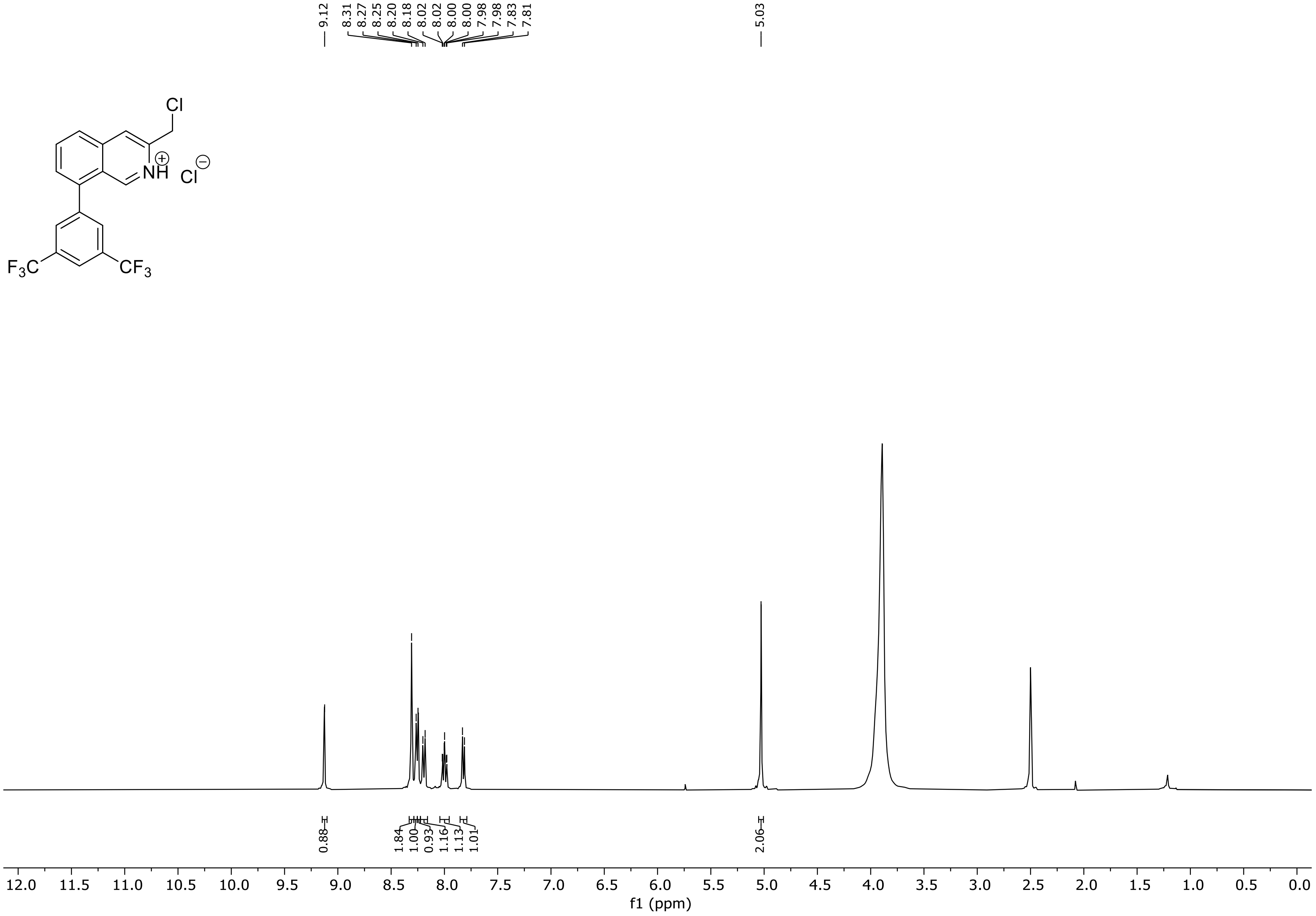

^{13}C-NMR spectrum of compound **12d**: (100 MHz, DMSO)

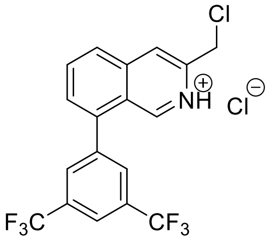

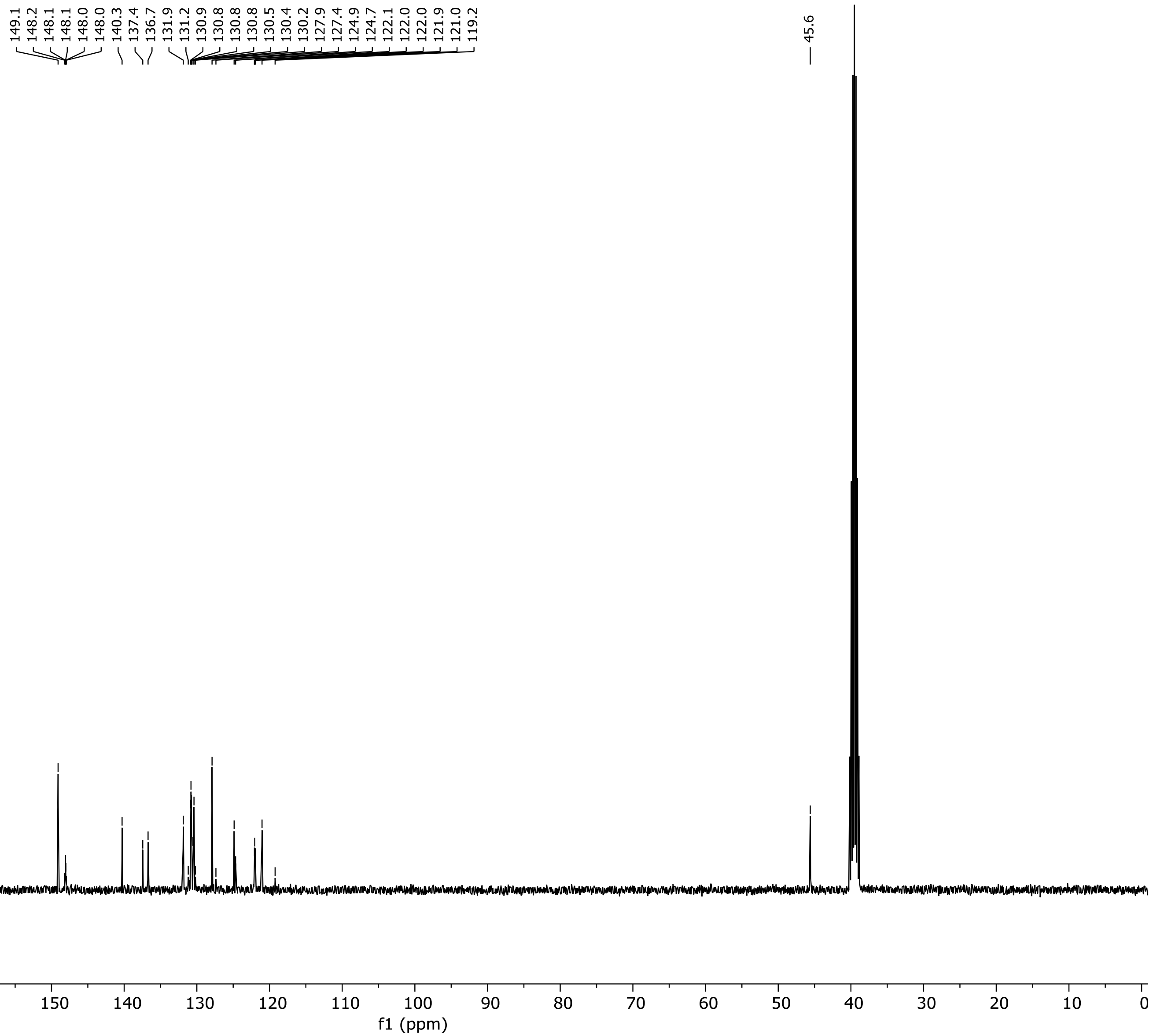

^{19}F-NMR spectrum of compound **12d**: (376 MHz, DMSO)

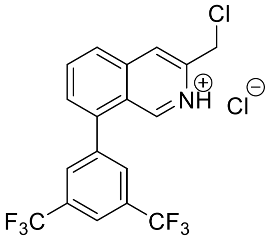

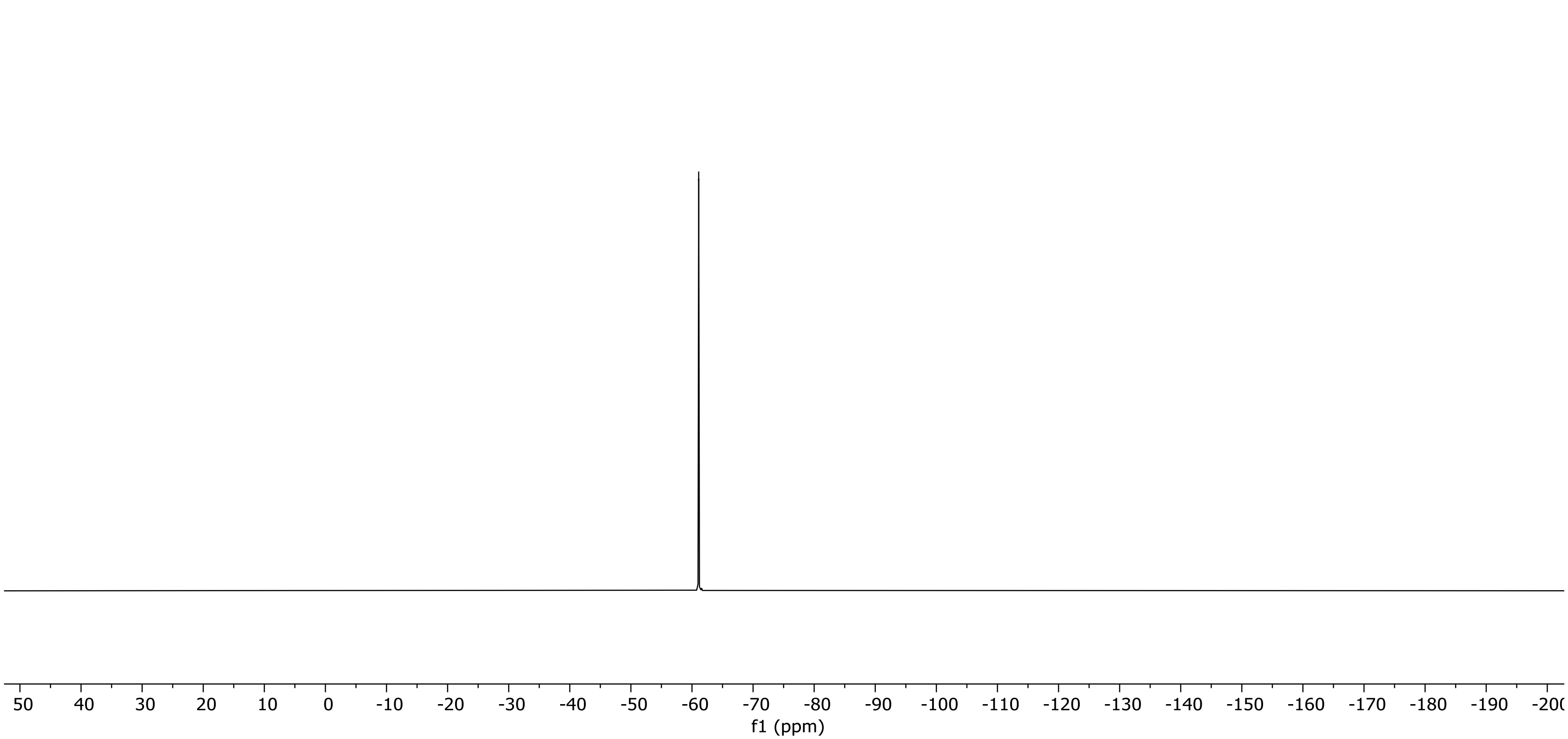

^{1}H-NMR spectrum of compound **12f**: (500 MHz, $CDCl_3$)

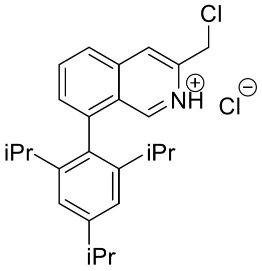

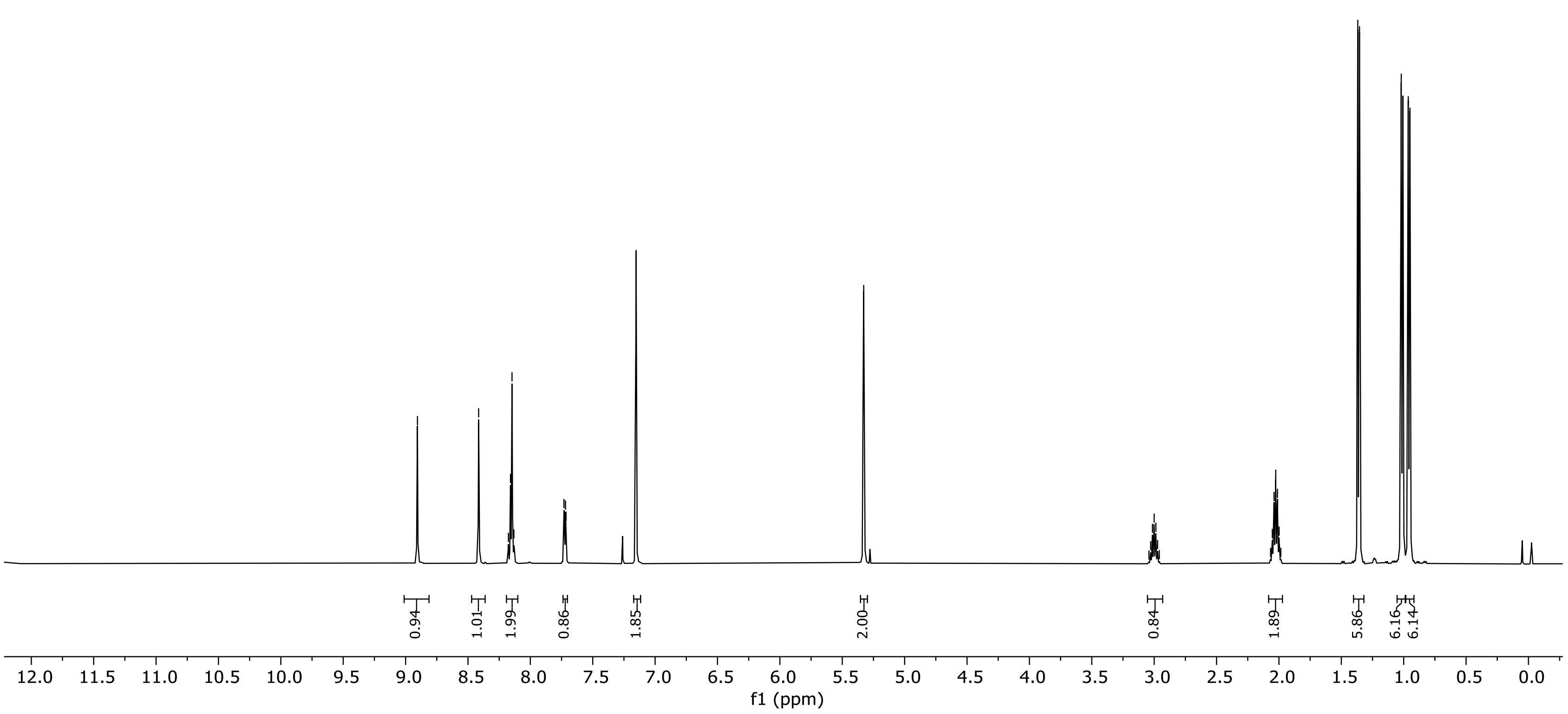

^{13}C-NMR spectrum of compound **12f**: (125 MHz, $CDCl_3$)

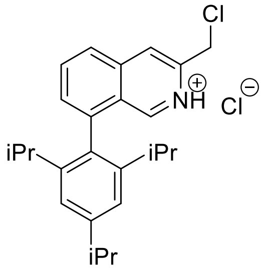

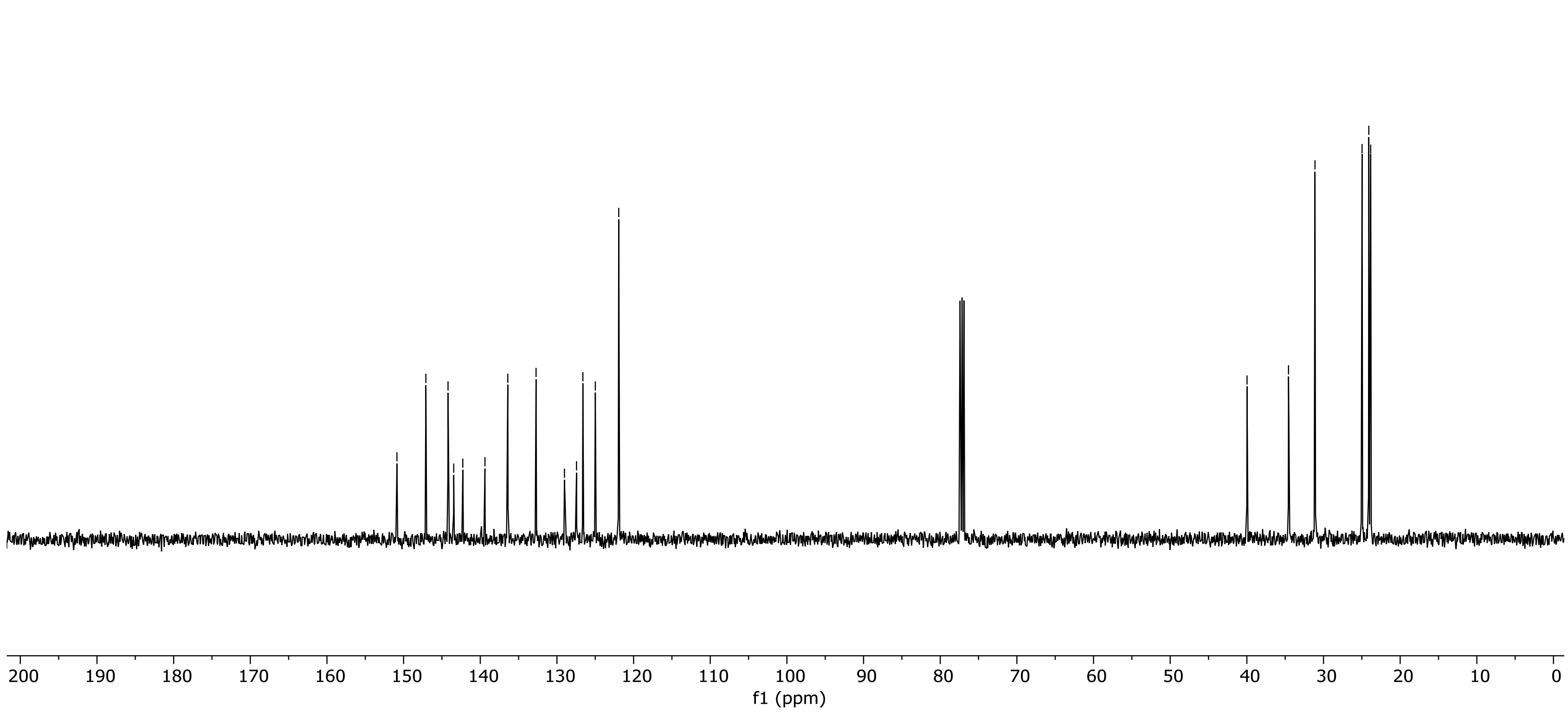

^{1}H-NMR spectrum of compound **12g**: (400 MHz, $CDCl_3$)

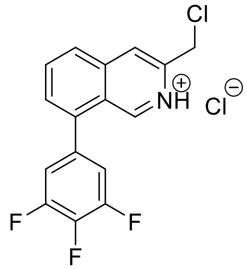

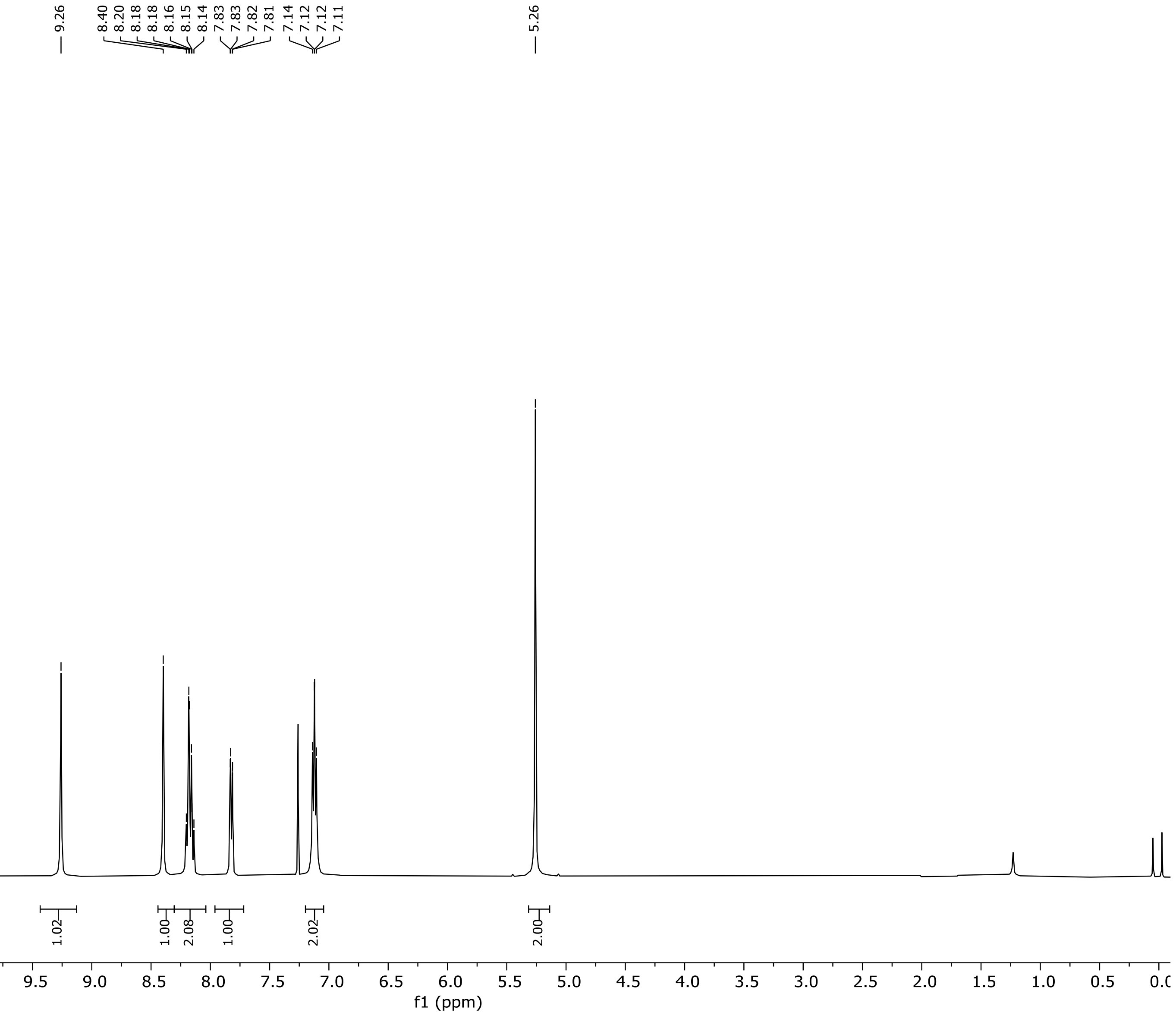

^{13}C-NMR spectrum of compound **12g**: (100 MHz, $CDCl_3$)

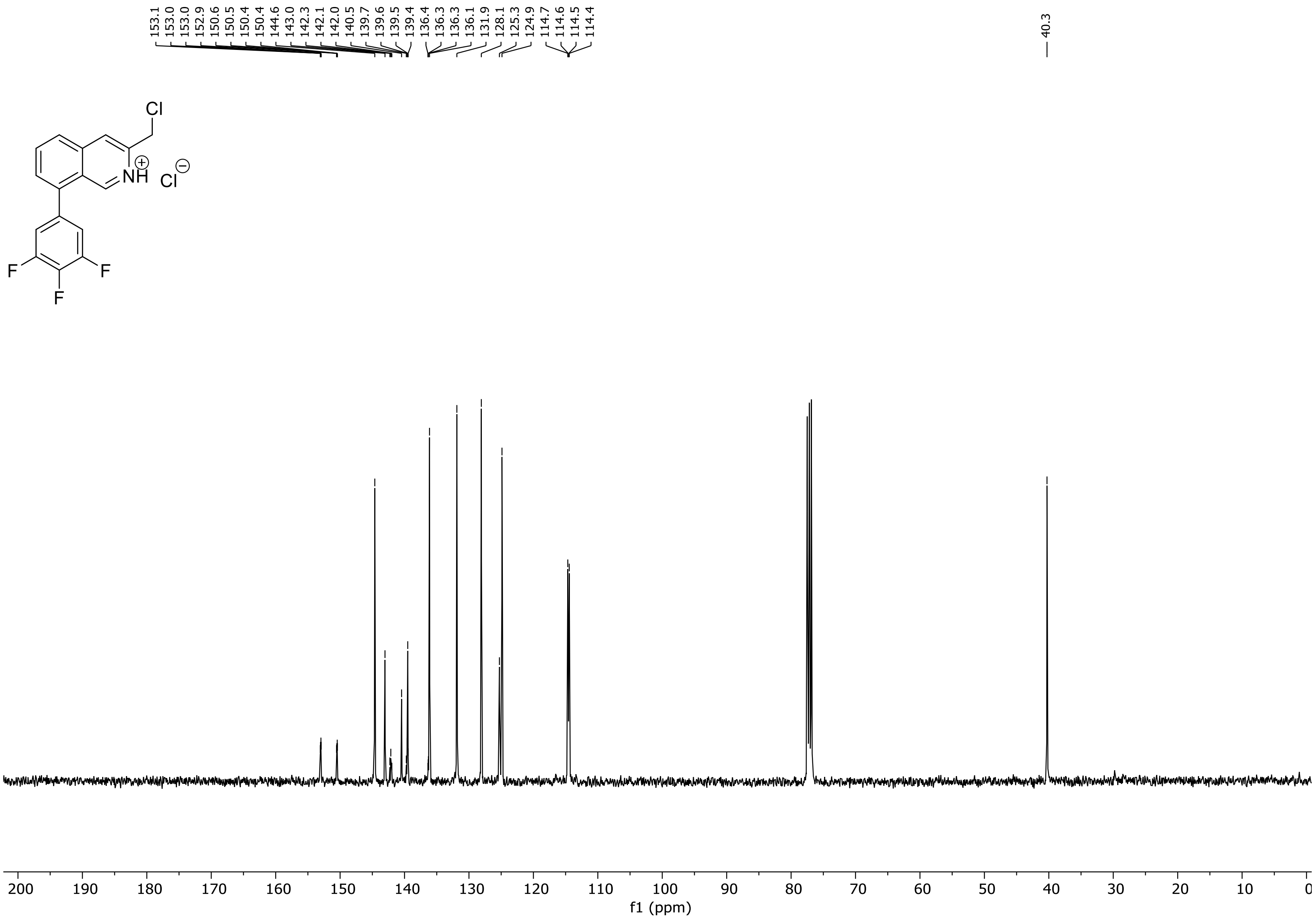

^{19}F-NMR spectrum of compound **12g**: (376 MHz, $CDCl_3$)

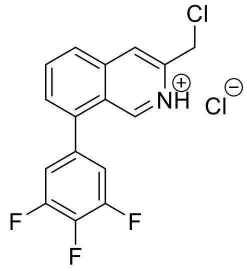

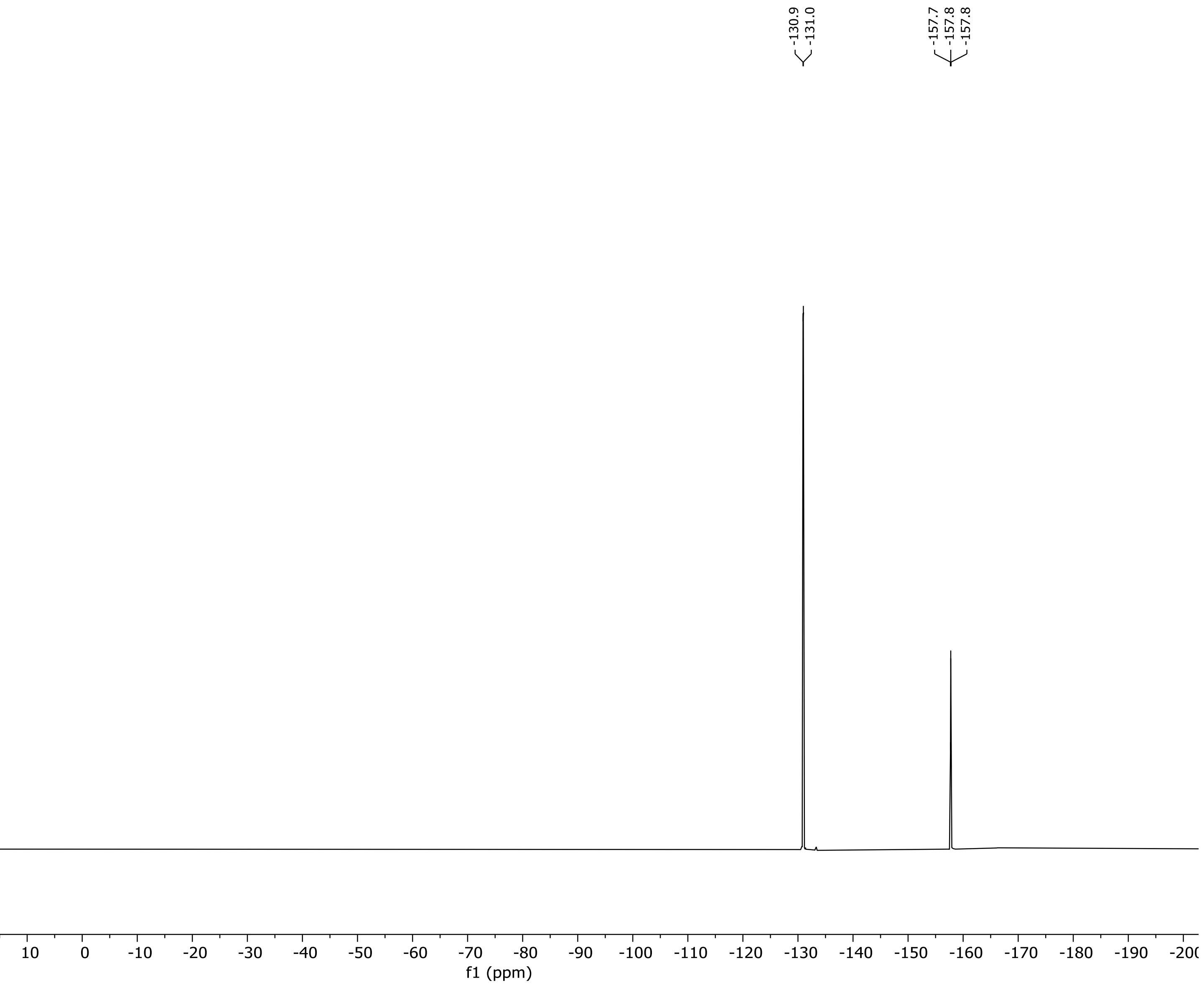

^{1}H-NMR spectrum of ligand (*S*,*S*)-$^{\mathbf{3,5\text{-}(di\text{-}CF3)}}$**iQ$_2$mc**: (400 MHz, $CDCl_3$)

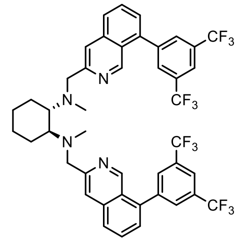

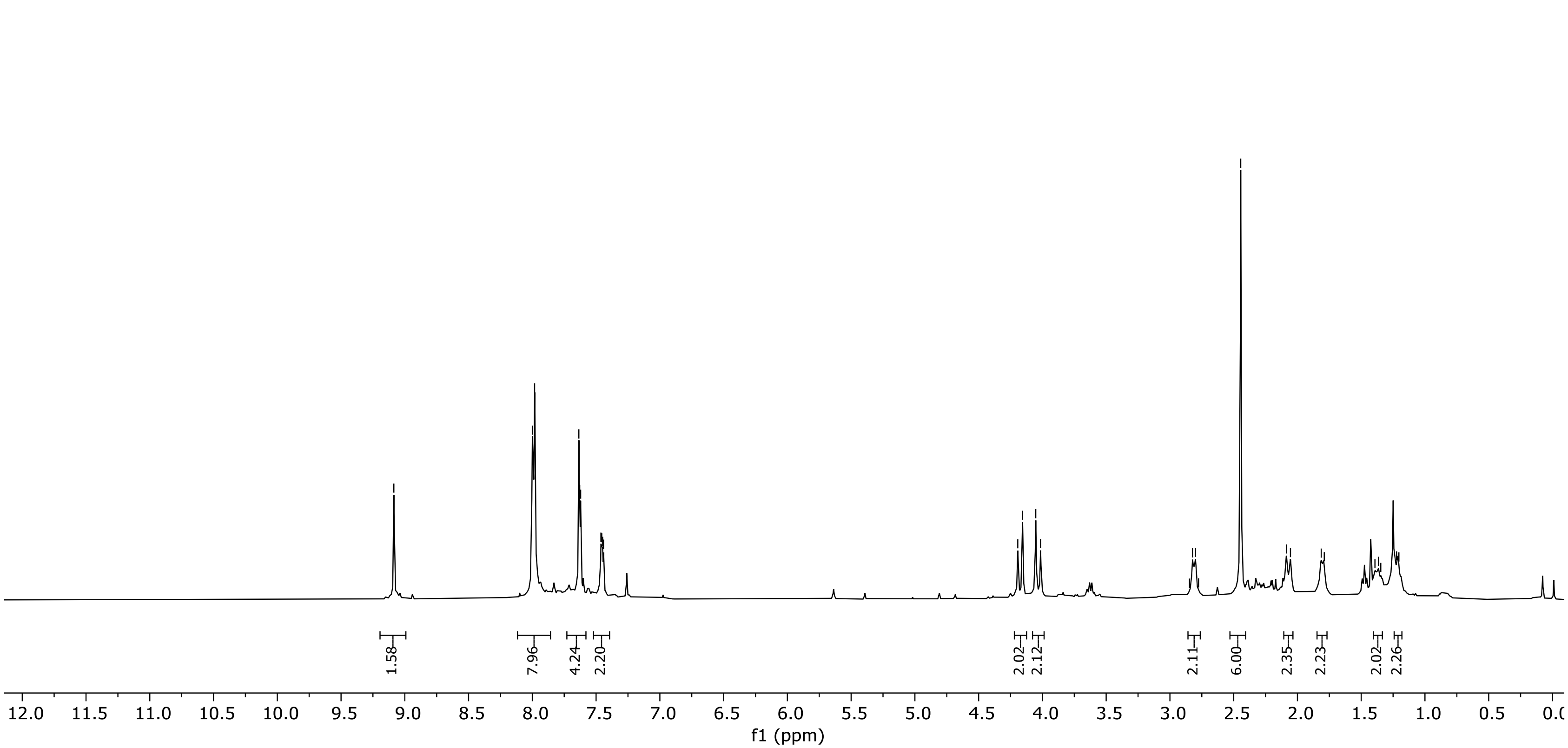

^{13}C-NMR spectrum of ligand (*S*,*S*)-$^{\mathbf{3,5\text{-}(di\text{-}CF3)}}$**iQ$_2$mc**: (100 MHz, $CDCl_3$)

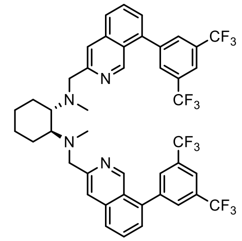

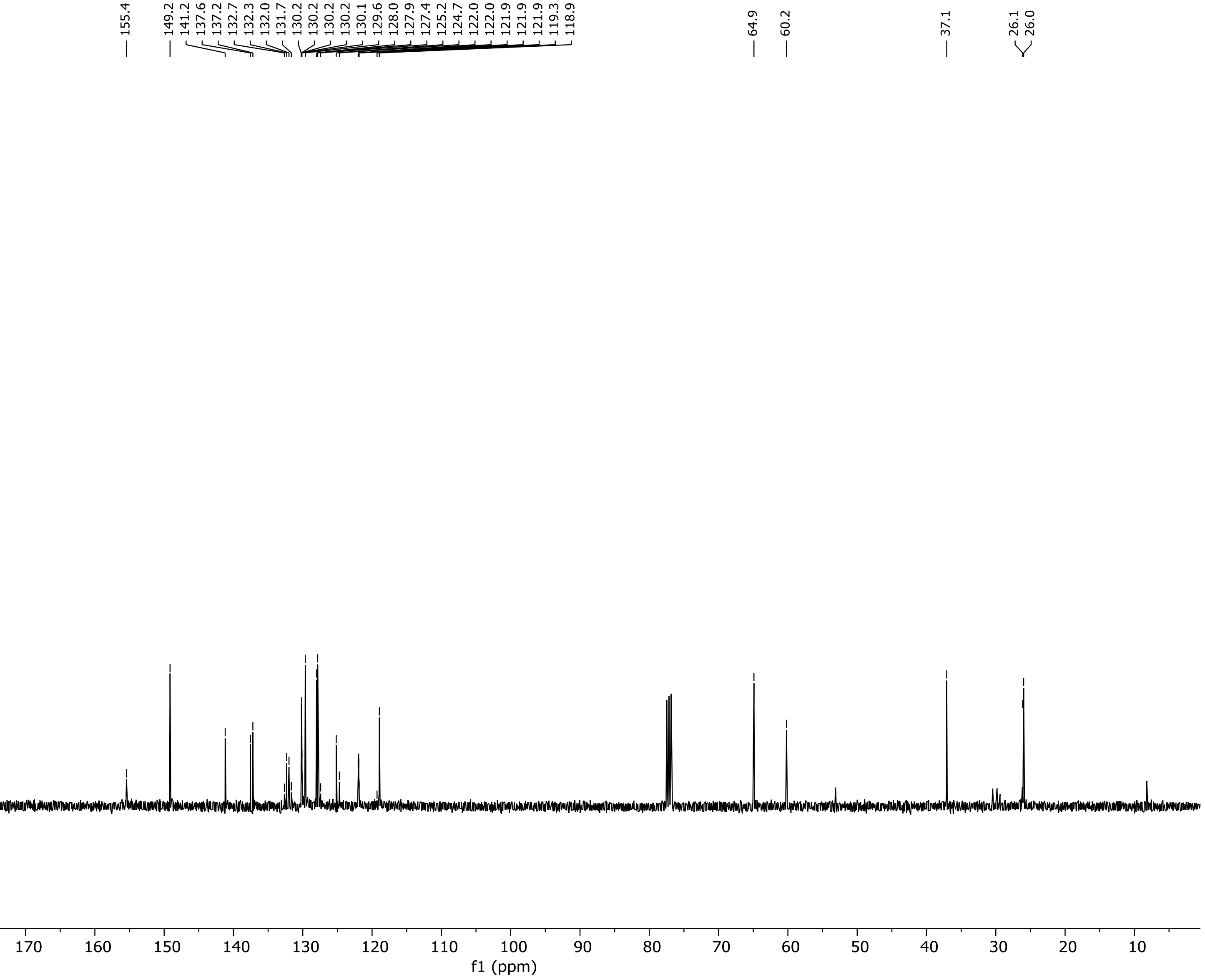

^{19}F-NMR spectrum of ligand (*S*,*S*)-$^{\mathbf{3,5\text{-}(di\text{-}CF3)}}$**iQ$_2$mc**: (376 MHz, $CDCl_3$)

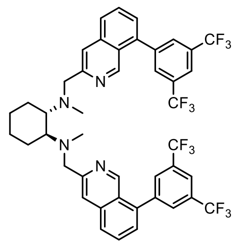

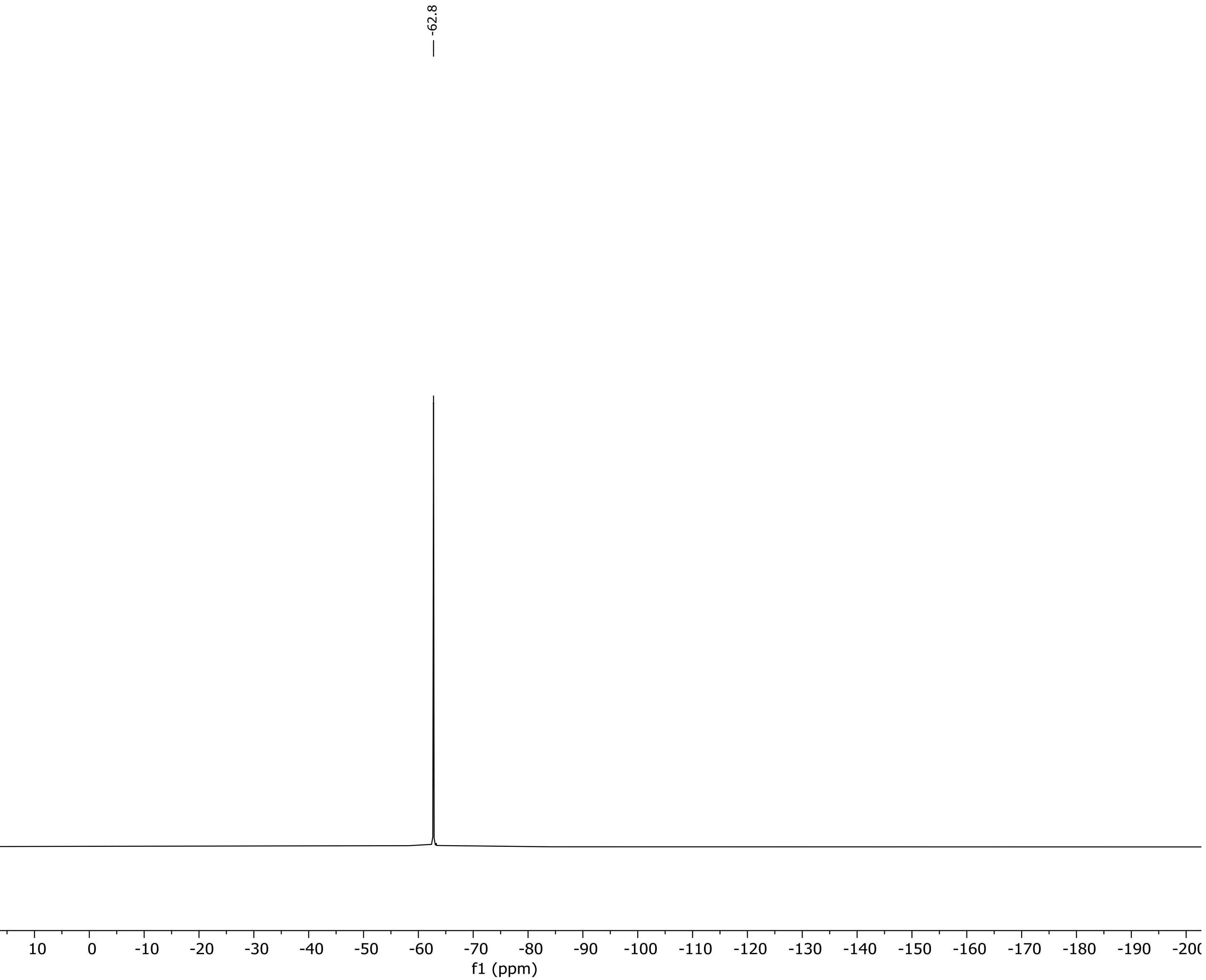

^{1}H-NMR spectrum of ligand (*S*,*S*)-$^{\mathbf{2,4,6\text{-}(tri\text{-}iPr)}}$**iQ$_2$mc**: (400 MHz, $CDCl_3$)

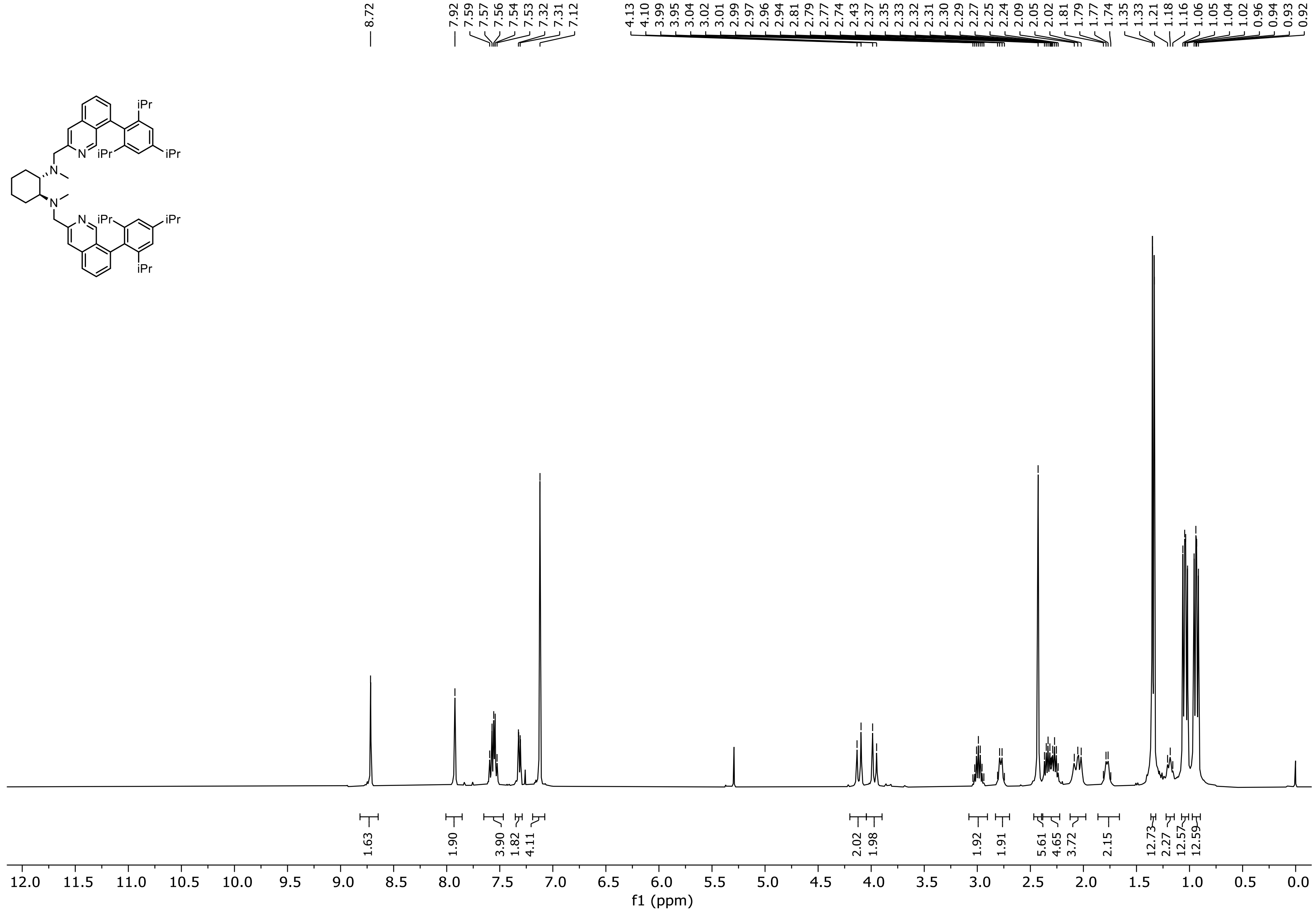

^{13}C-NMR spectrum of ligand (*S*,*S*)-$^{\mathbf{2,4,6\text{-}(tri\text{-}iPr)}}$**iQ$_2$mc**: (100 MHz, $CDCl_3$)

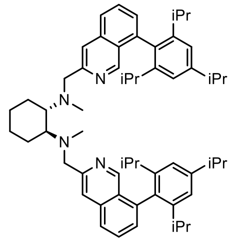

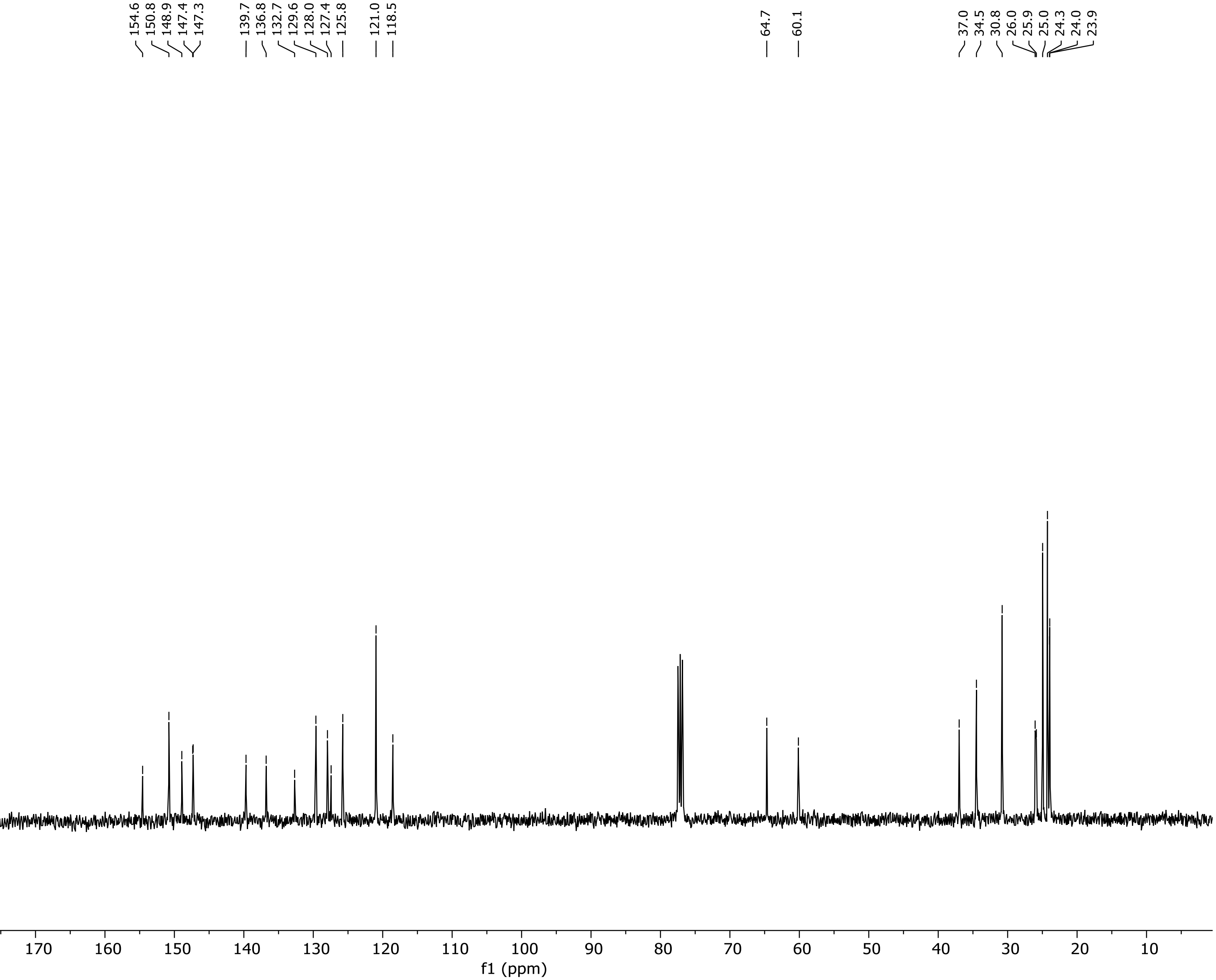

^{1}H-NMR spectrum of ligand (*S*,*S*)-[3,5-(di-*t*-Bu)]**iQ$_2$dp**: (500 MHz, $CDCl_3$)

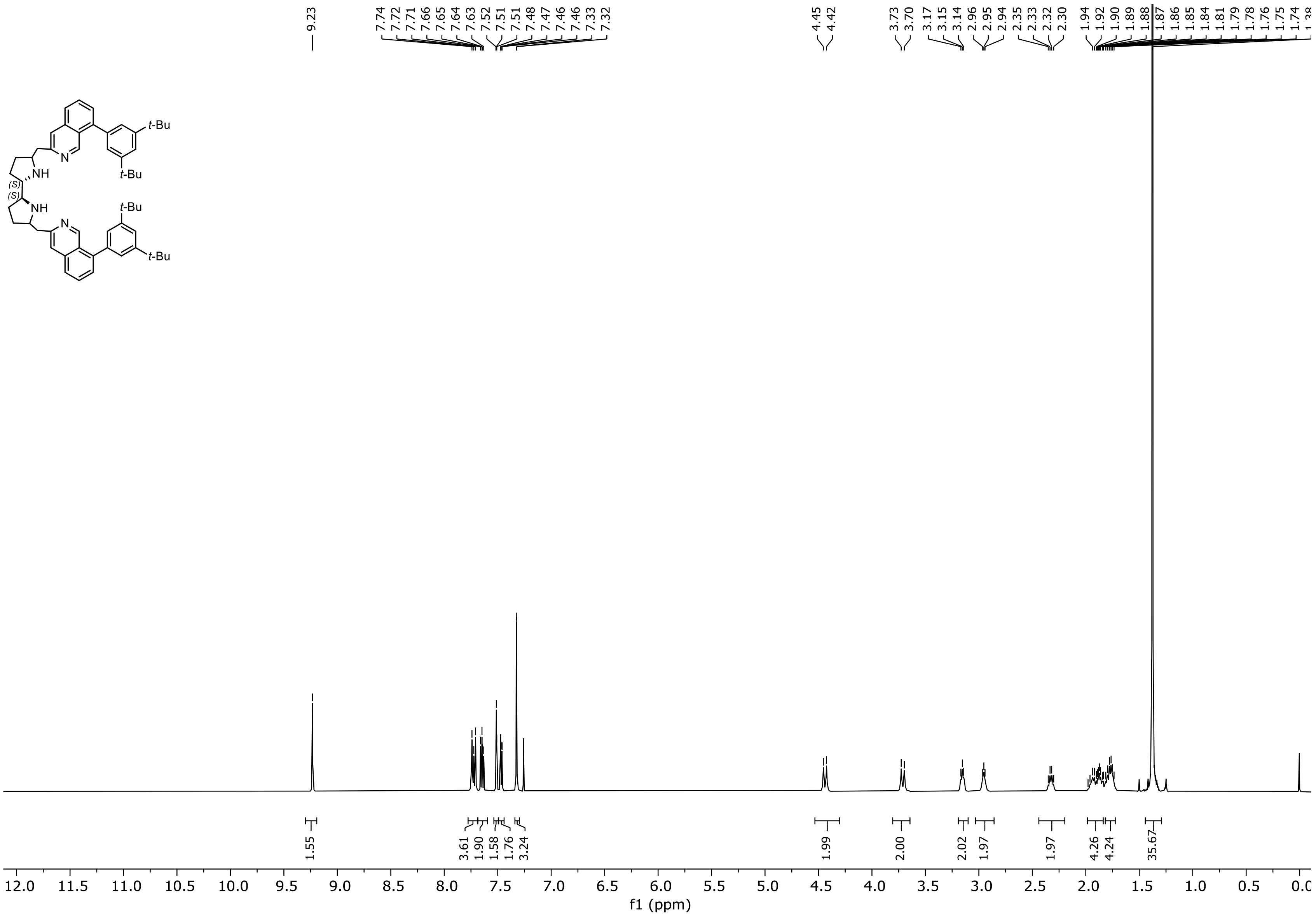

^{13}C-NMR spectrum of ligand (*S*,*S*)-$^{\mathbf{3,5\text{-}(di\text{-}\mathit{t}\text{-}Bu)}}$**iQ$_2$dp**: (126 MHz, $CDCl_3$)

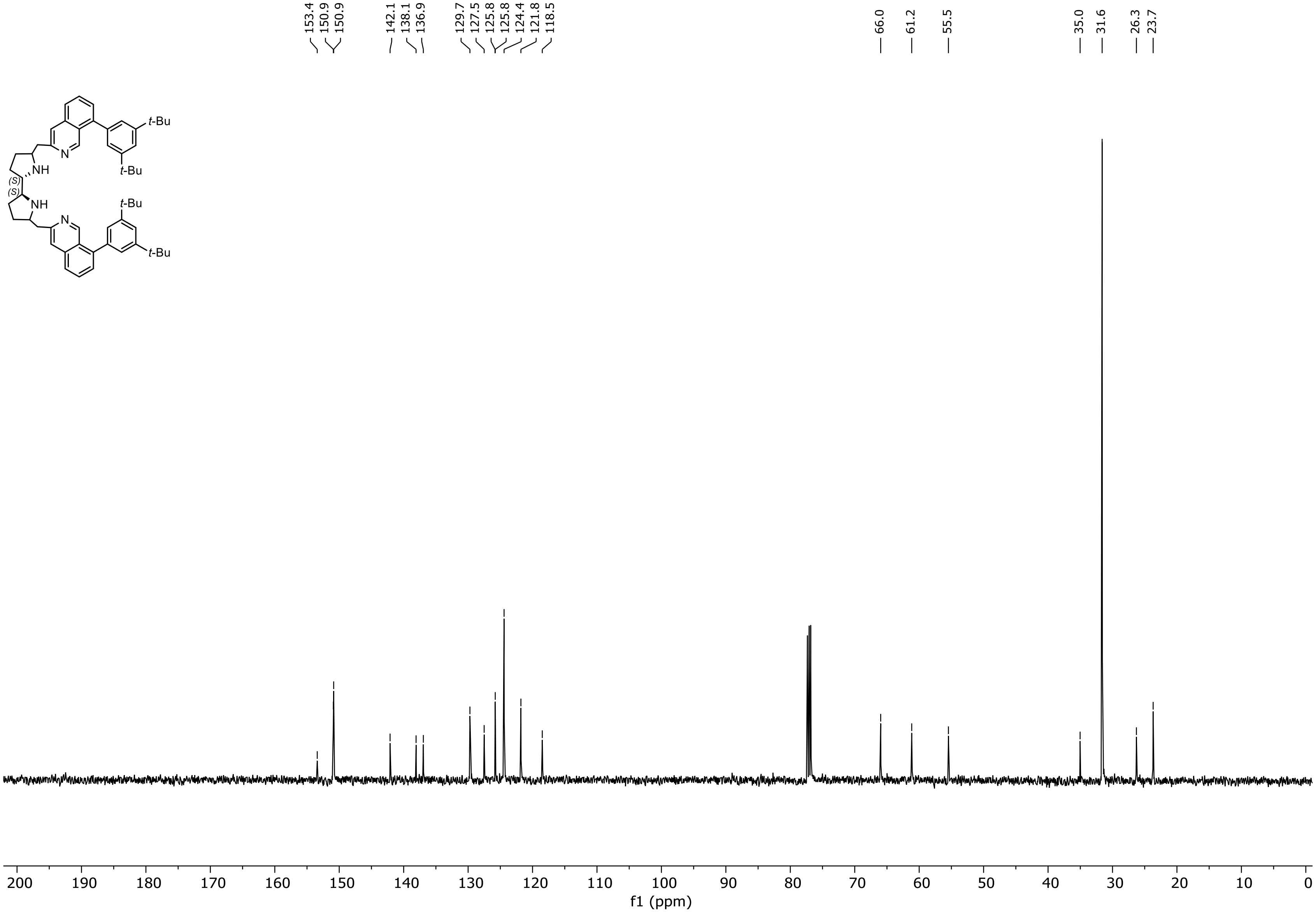

^{1}H-NMR spectrum of ligand (*S*,*S*)-$^{\mathbf{3,5\text{-}(di\text{-}CF3)}}$**iQ$_2$dp**: (400 MHz, $CDCl_3$)

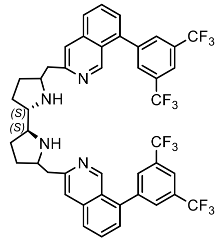

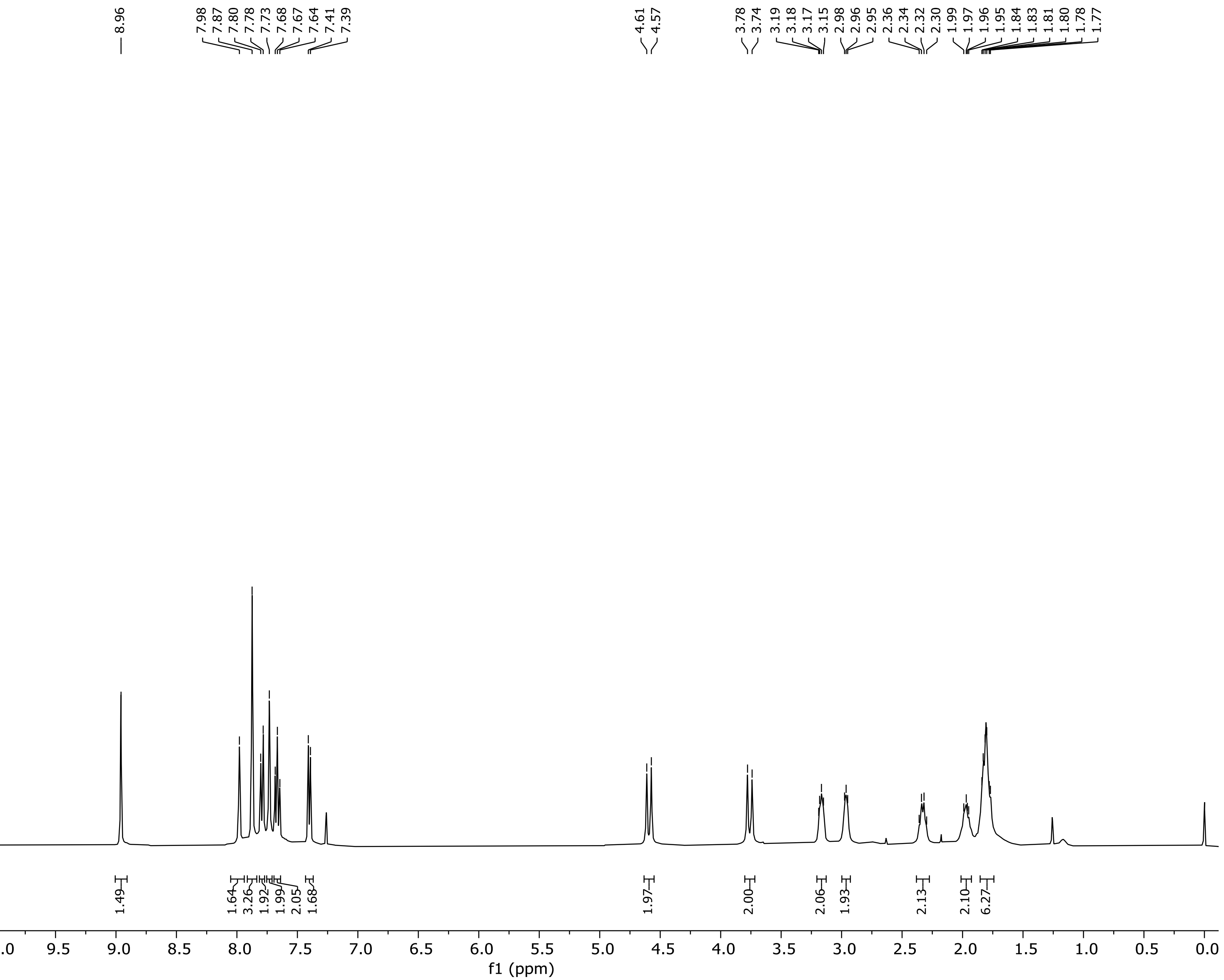

^{13}C-NMR spectrum of ligand (*S*,*S*)-$^{\mathbf{3,5\text{-}(di\text{-}CF3)}}$**iQ$_2$dp**: (100 MHz, $CDCl_3$)

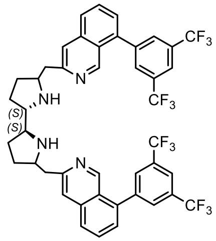

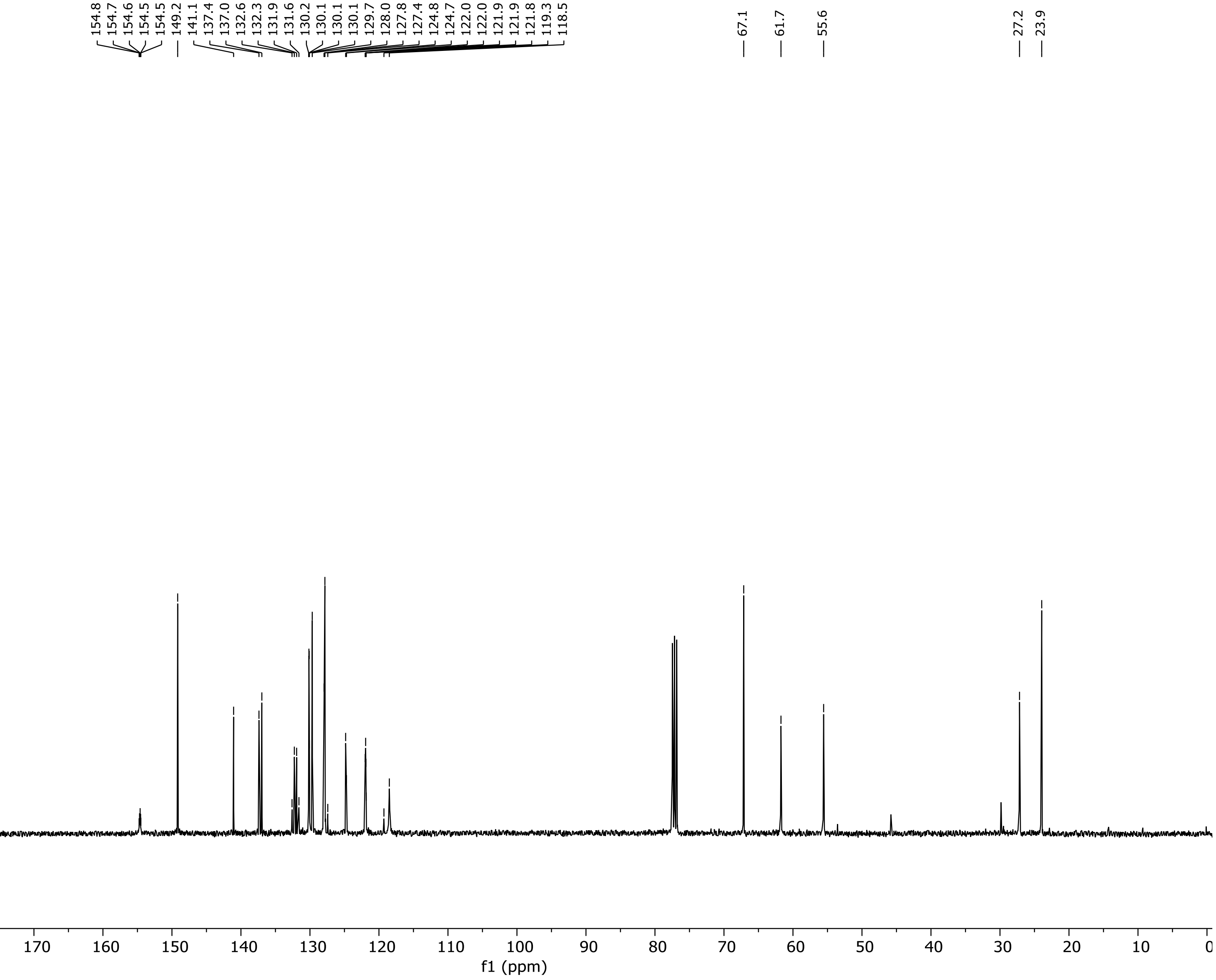

^{19}F-NMR spectrum of ligand (*S*,*S*)-**$^{3,5\text{-(di-CF3)}}$iQ$_2$dp**: (376 MHz, $CDCl_3$)

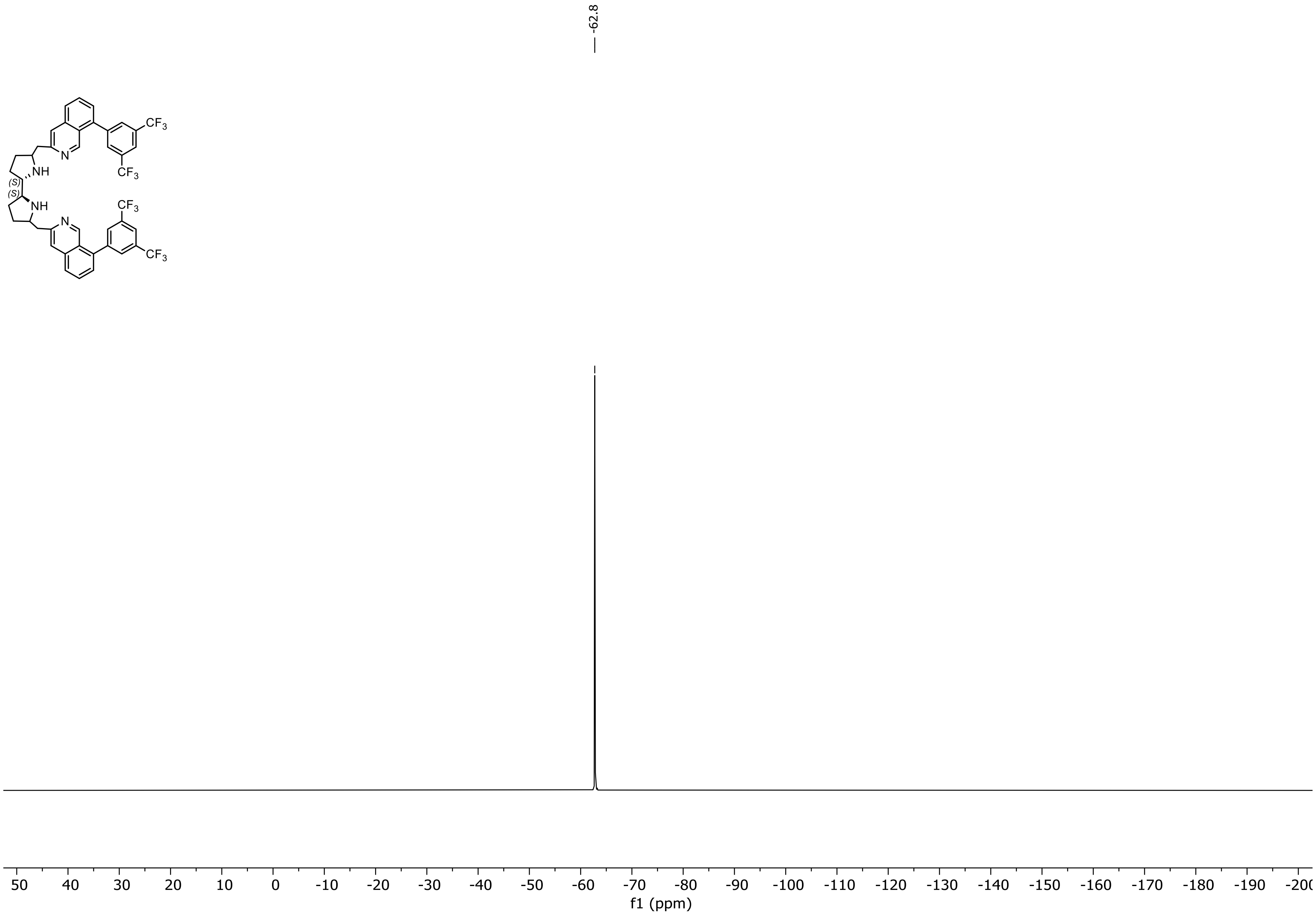

^{1}H-NMR spectrum of ligand (*R*,*R*)-$^{\mathbf{3,5\text{-(di-CF3)}}}$**iQ$_2$dp**: (400 MHz, $CDCl_3$)

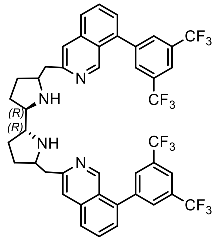

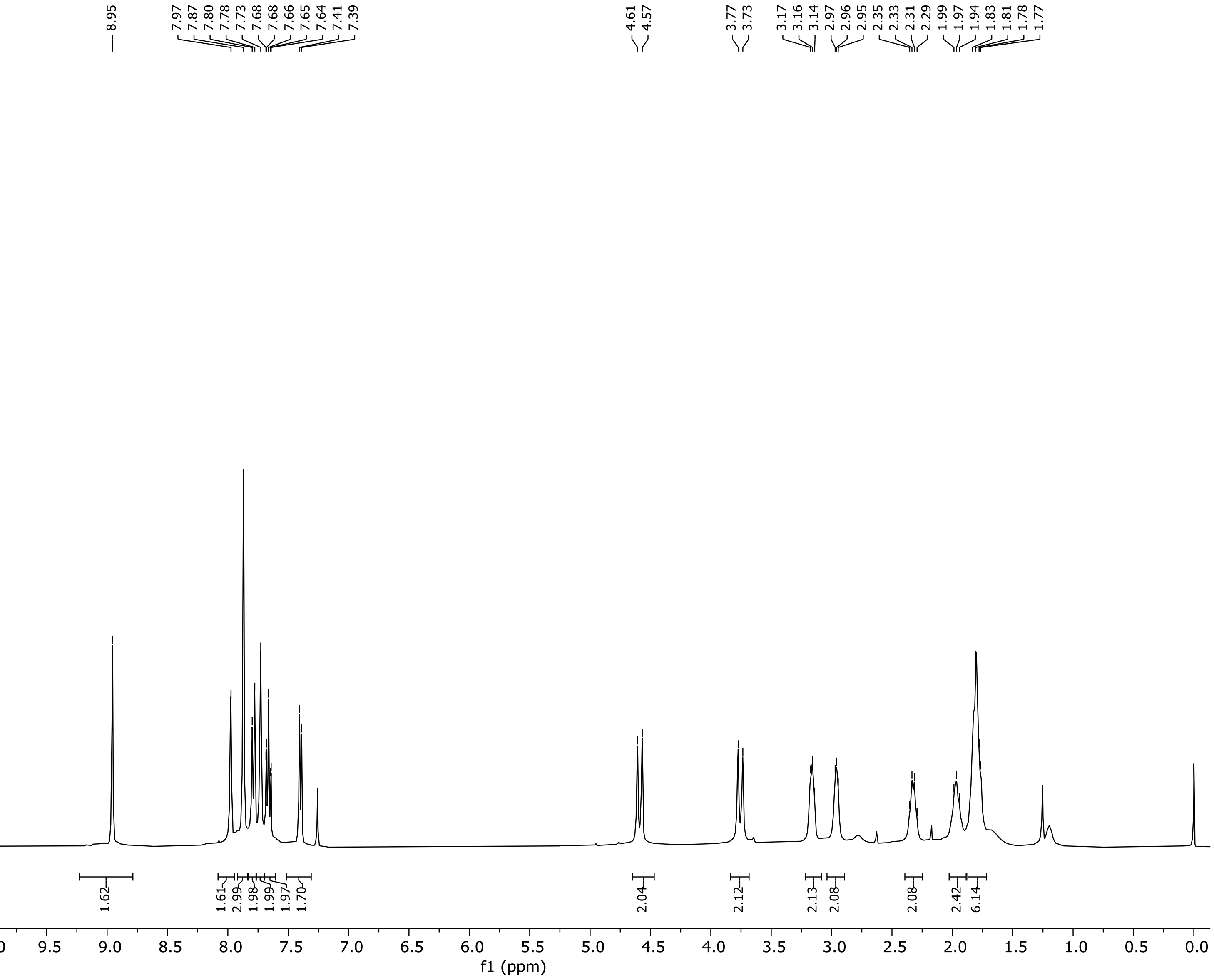

^{13}C-NMR spectrum of ligand (*R*,*R*)-**[3,5-(di-CF3)]iQ$_2$dp**: (100 MHz, $CDCl_3$)

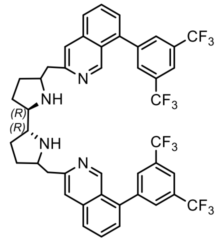

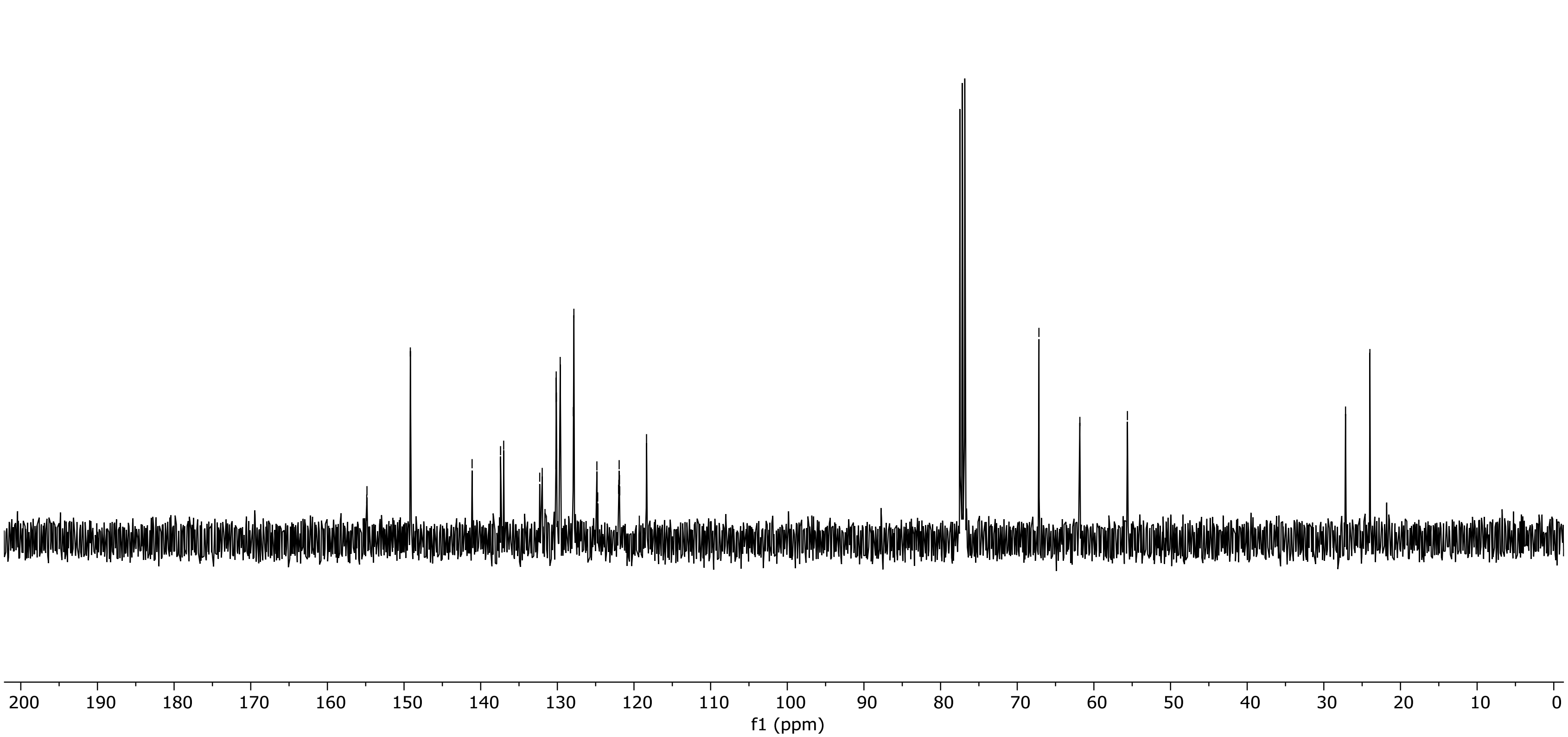

^{19}F-NMR spectrum of ligand (*R*,*R*)-**[3,5-(di-CF3)]iQ$_2$dp**: (376 MHz, CDCl$_3$)

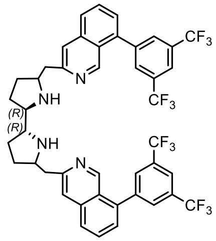

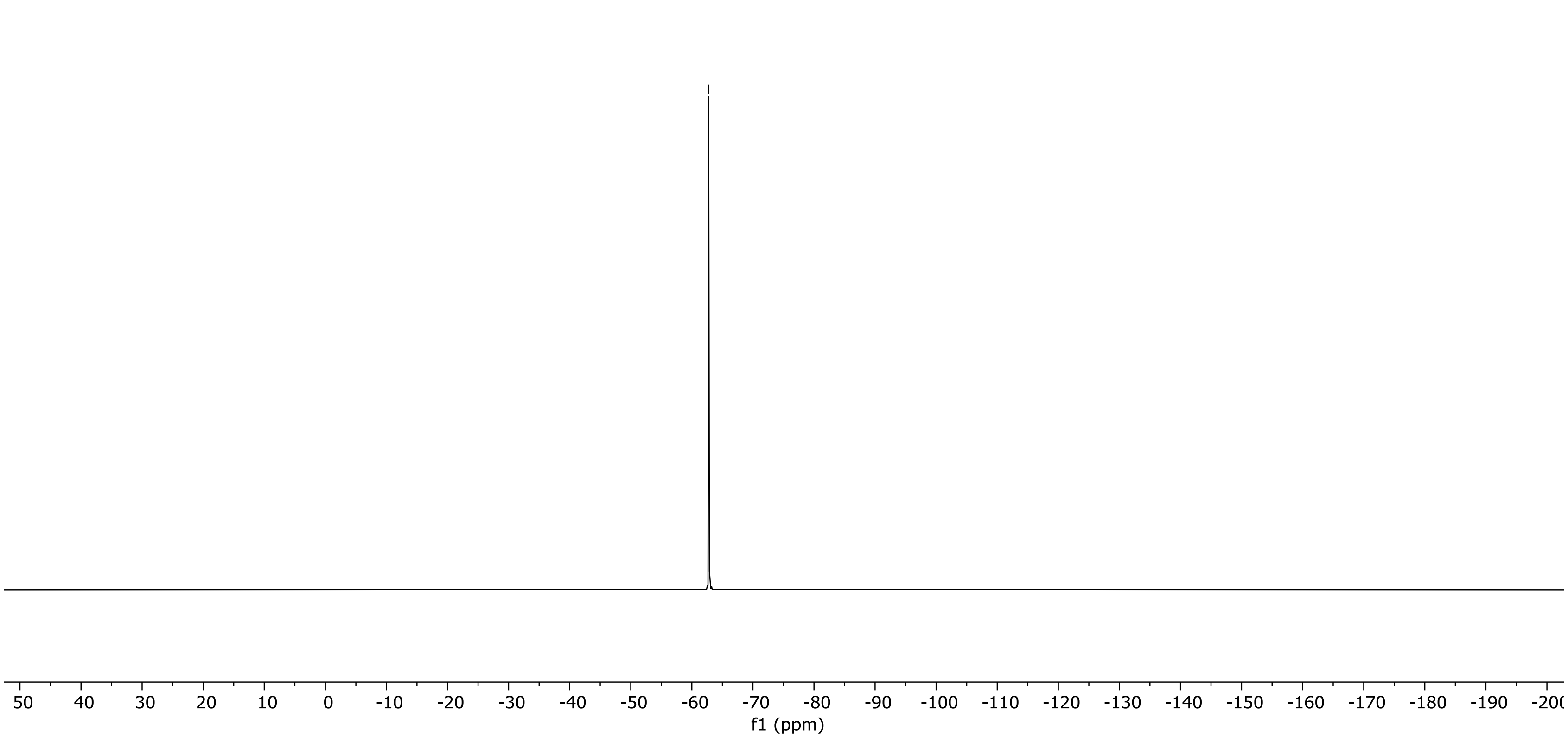

^{1}H-NMR spectrum of ligand (*S*,*S*)-**[2,4,6-(tri-iPr)]iQ$_2$dp**: (500 MHz, $CDCl_3$)

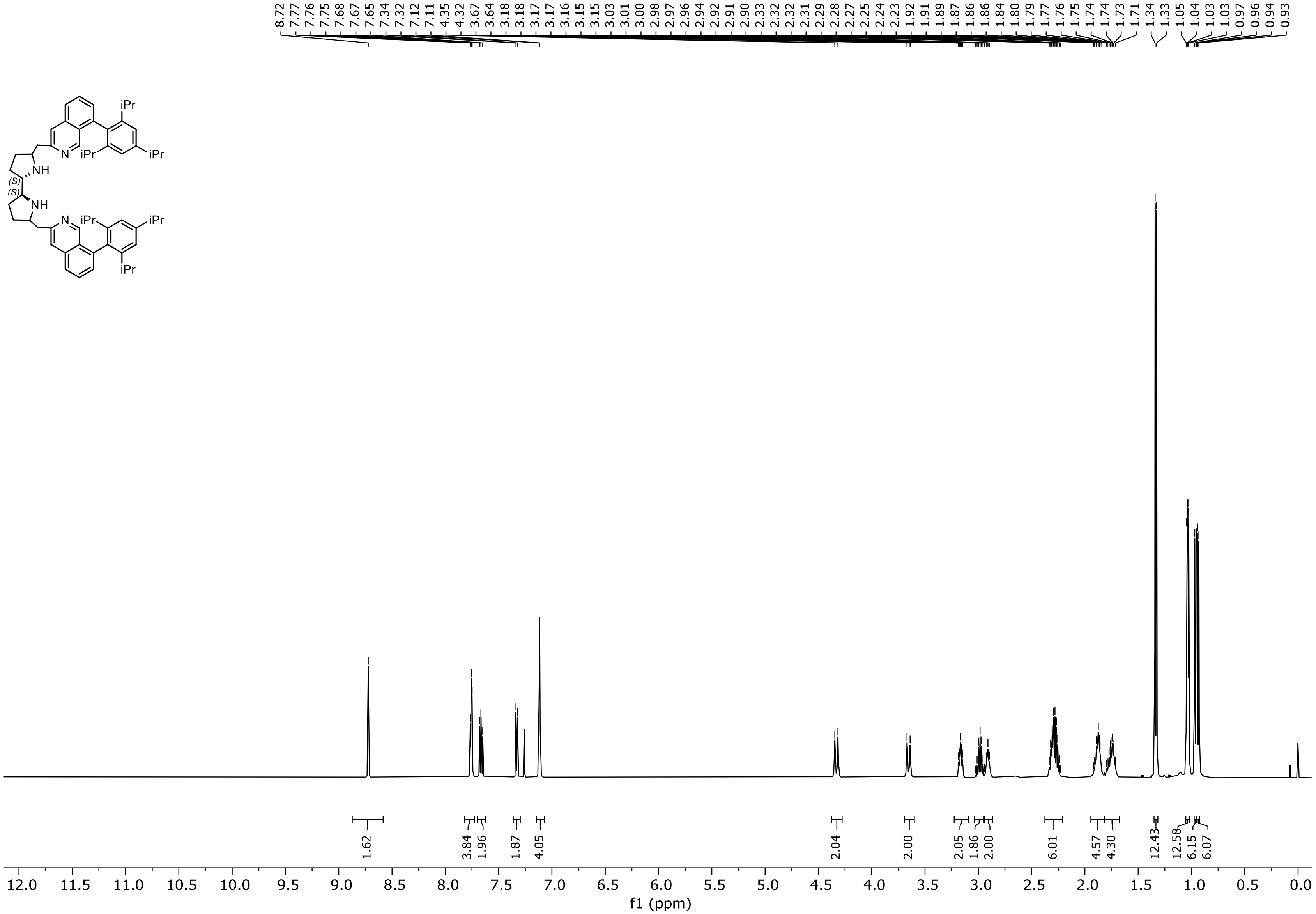

^{13}C-NMR spectrum of ligand (*S*,*S*)-$^{\mathbf{2,4,6\text{-}(tri\text{-}iPr)}}$**iQ$_2$dp**: (126 MHz, CDCl$_3$)

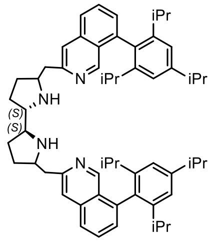

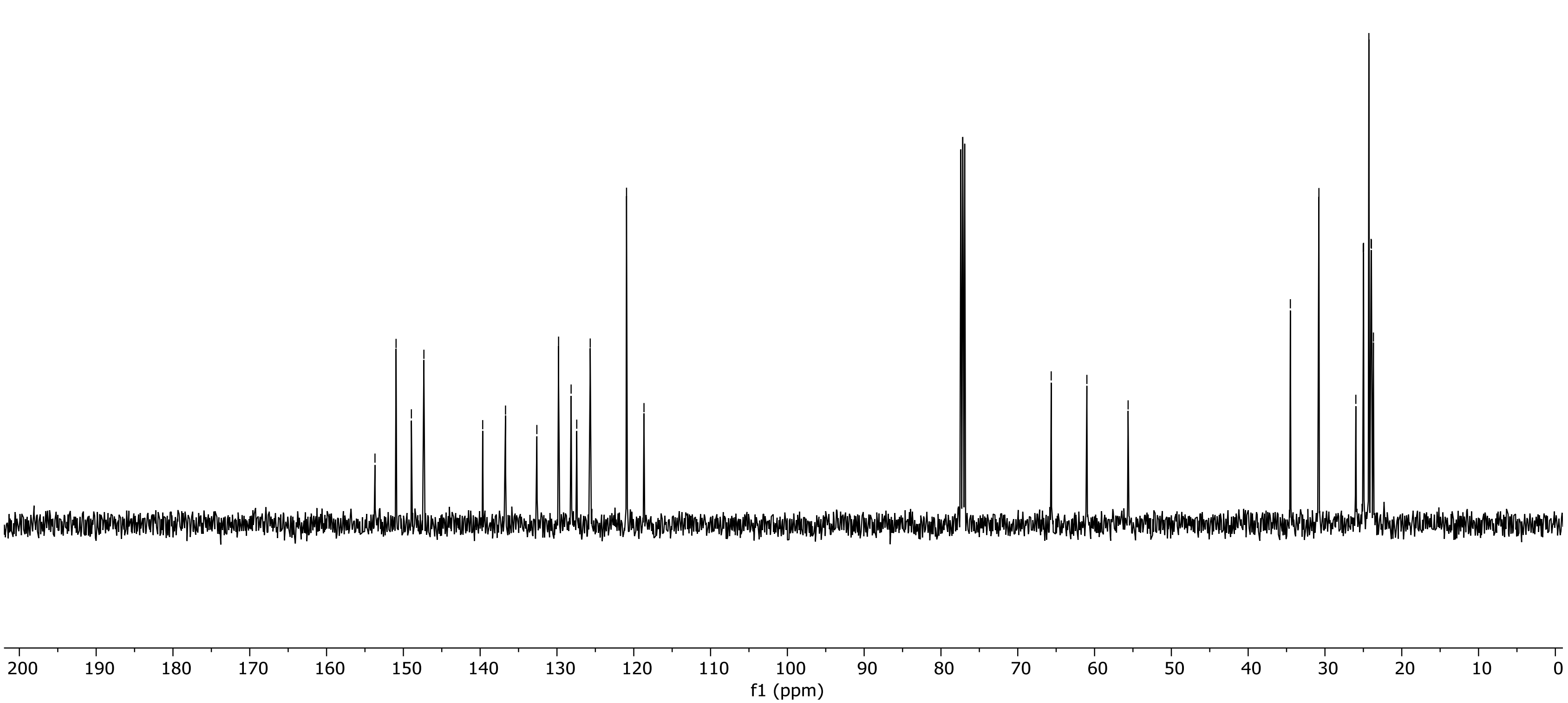

^{1}H-NMR spectrum of ligand (*S*,*S*)-**[3,4,5-(tri-F)]iQ₂dp**: (400 MHz, $CDCl_3$)

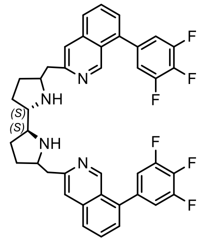

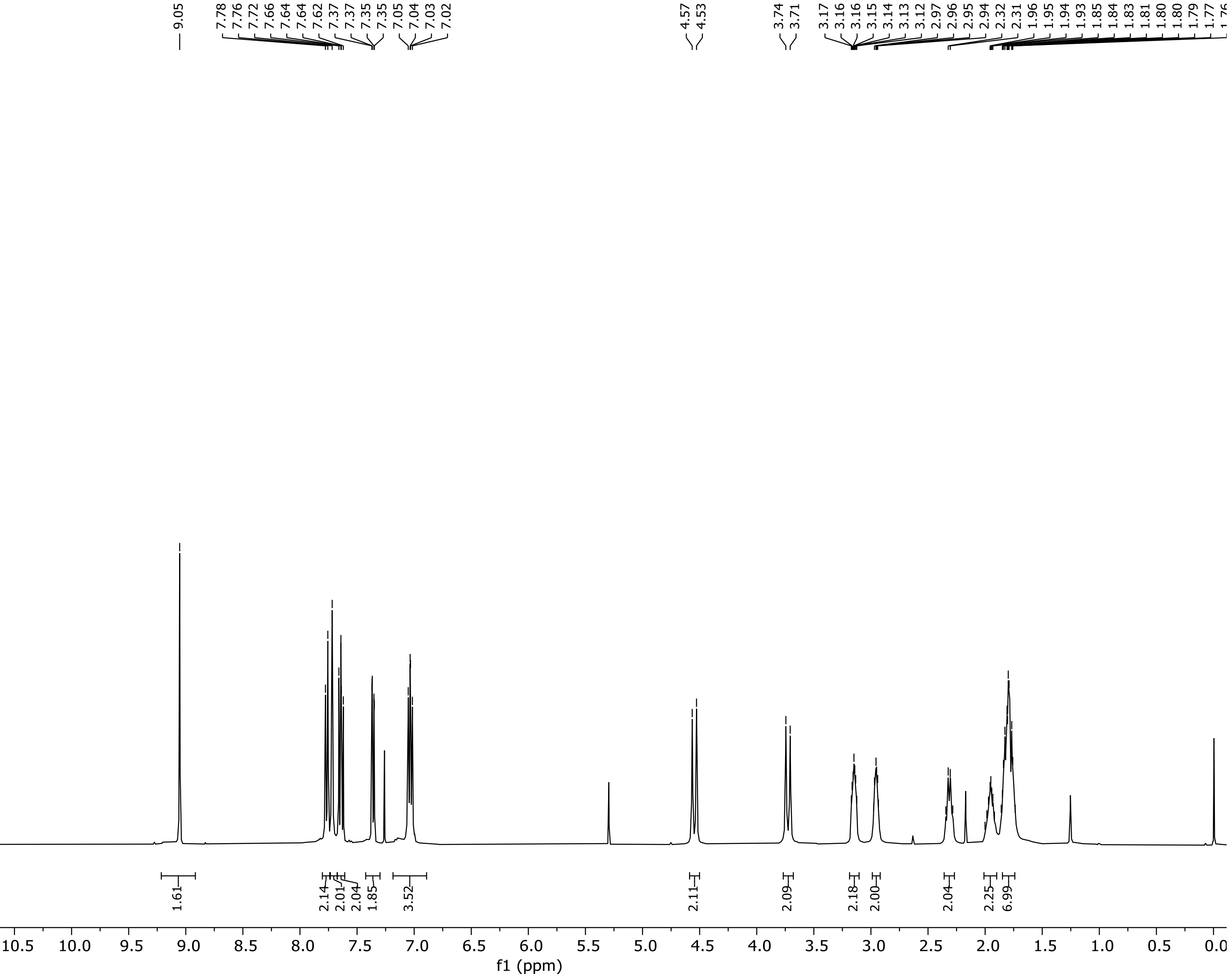

^{13}C-NMR spectrum of ligand (*S*,*S*)-$^{\mathbf{3,4,5\text{-}(tri\text{-}F)}}$**iQ$_2$dp**: (100 MHz, CDCl$_3$)

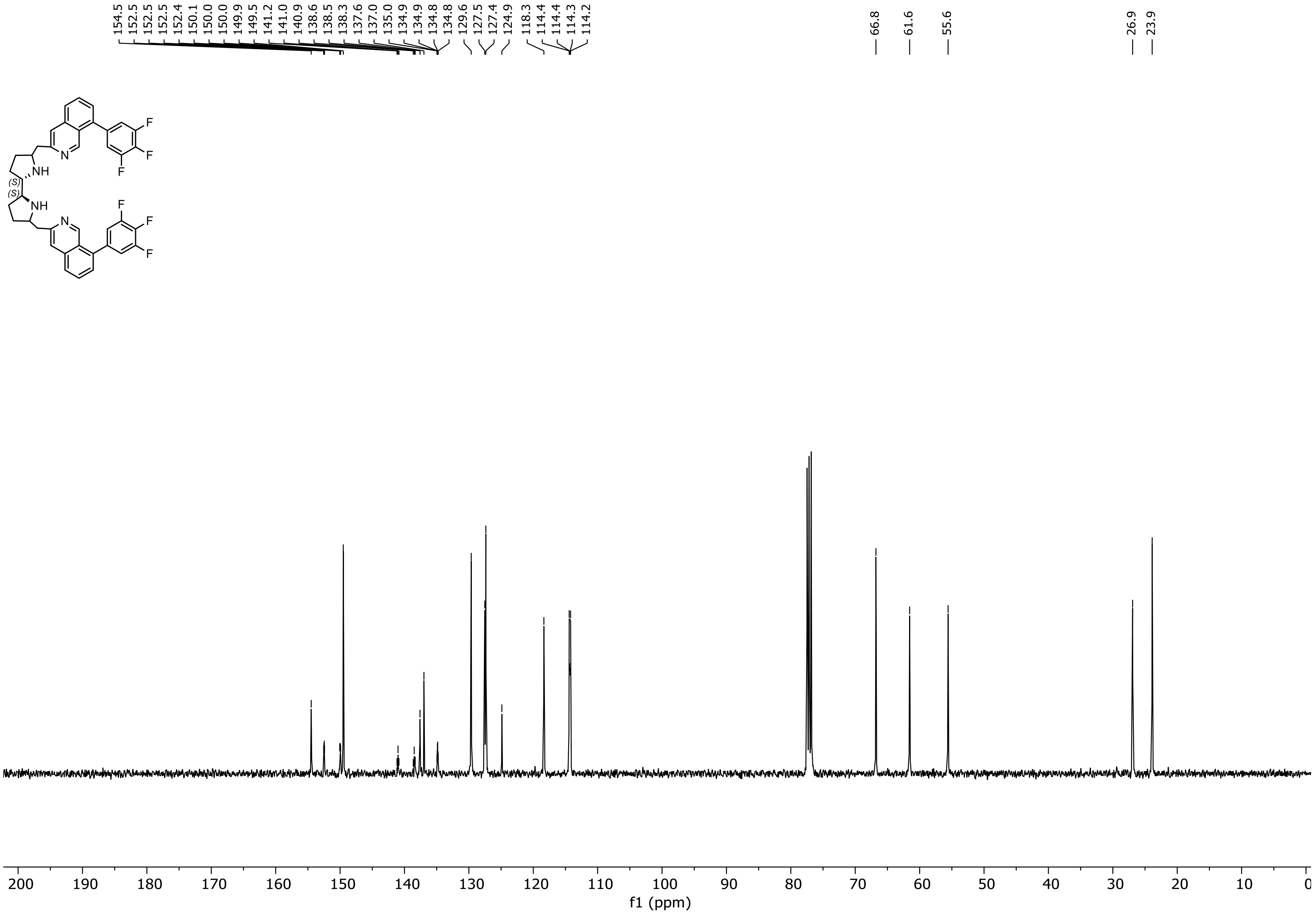

^{19}F-NMR spectrum of ligand (*S*,*S*)-**$^{3,4,5\text{-(tri-F)}}$iQ$_2$dp**: (376 MHz, $CDCl_3$)

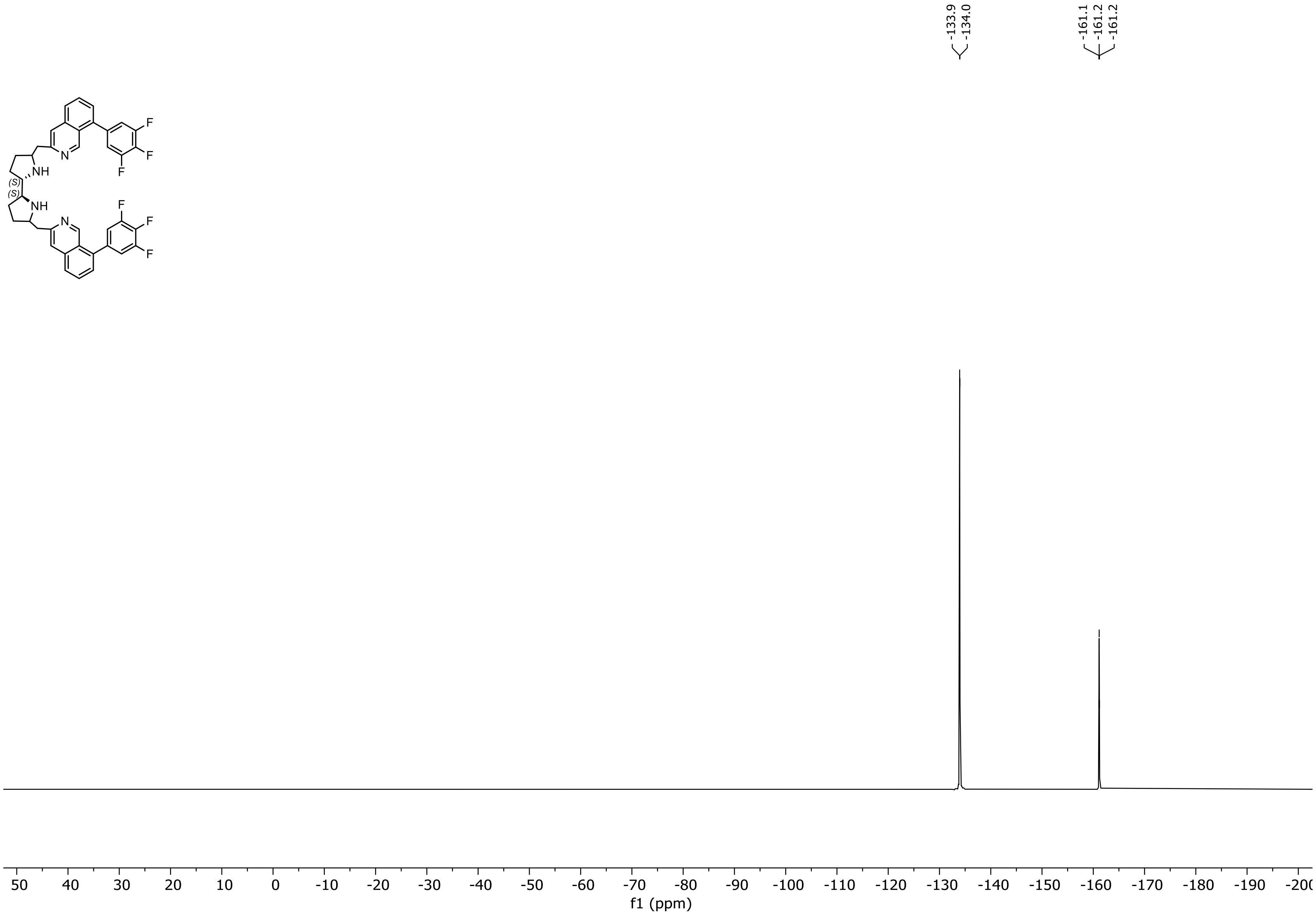

^{1}H-NMR spectrum of (*S*,*S*)-**Fe($^{3,5\text{-(di-CF3)}}$iQ$_2$mc)**: (500 MHz, CD_2Cl_2)

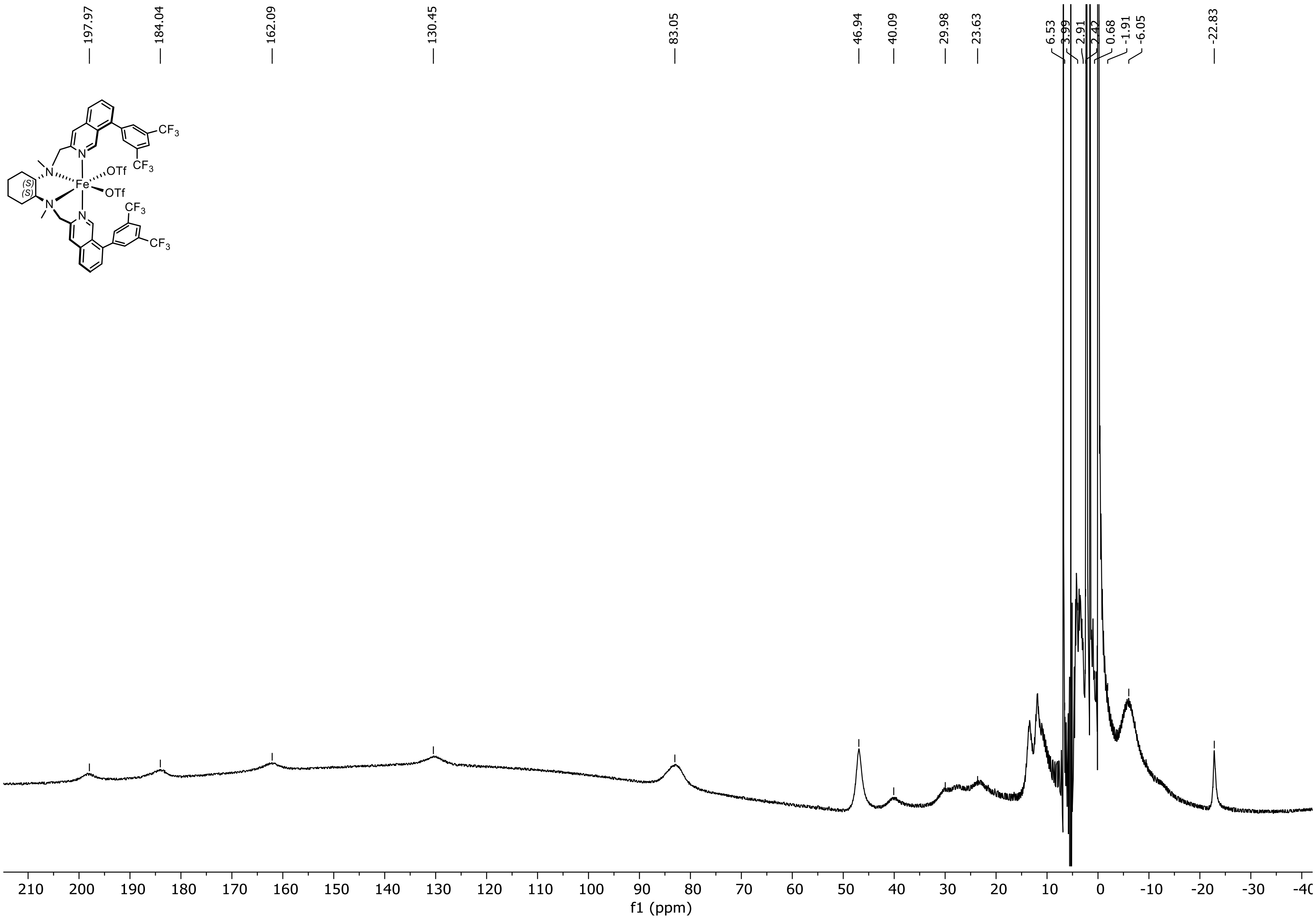

^{1}H-NMR spectrum of (*S*,*S*)-**Fe($^{2,4,6\text{-(tri-iPr)}}iQ_2$mc)**: (500 MHz, CD_2Cl_2)

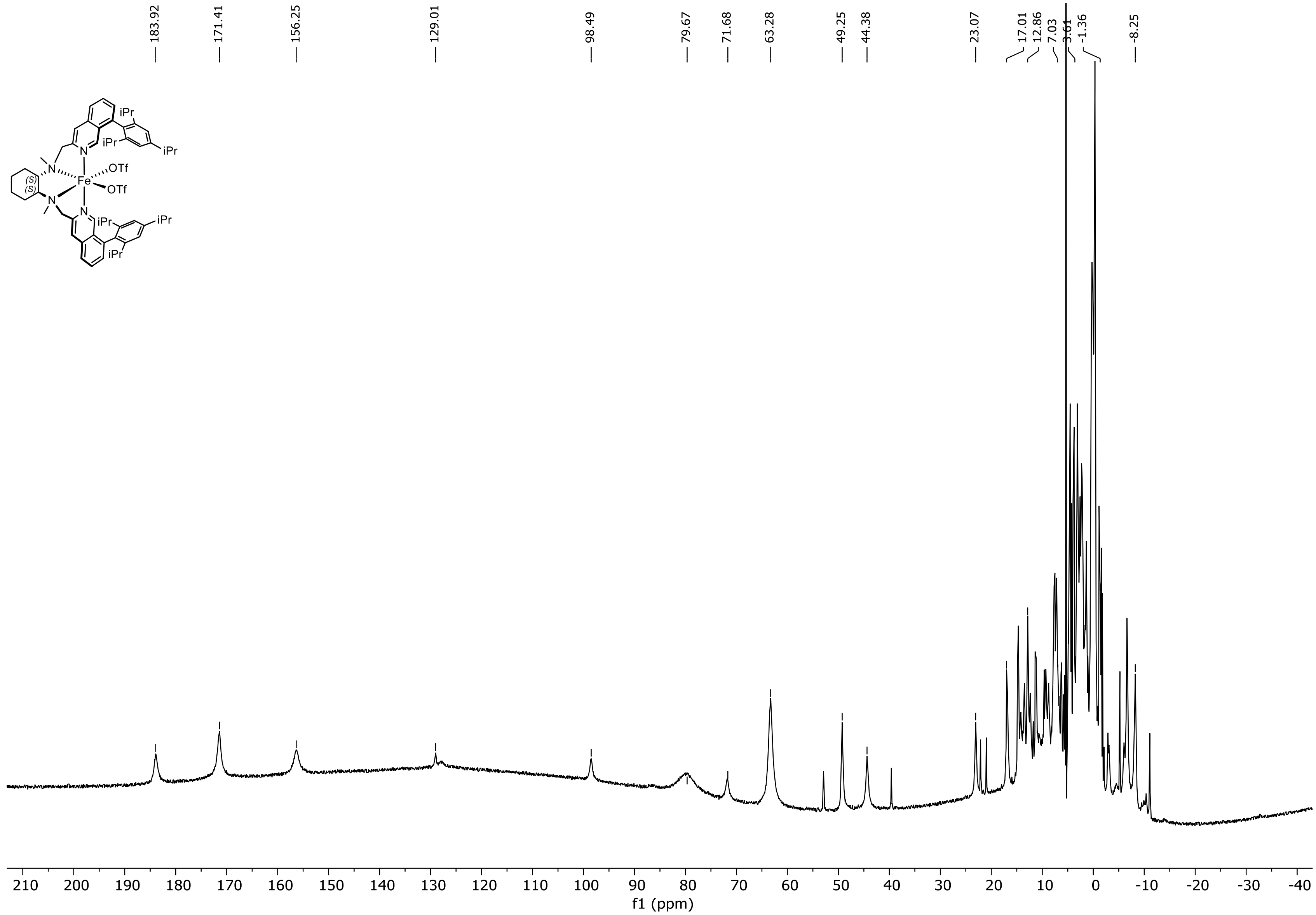

^{1}H-NMR spectrum of (*S*,*S*)-**Fe($^{3,5\text{-(di-}t\text{-Bu)}}iQ_2$dp)**: (500 MHz, CD_2Cl_2)

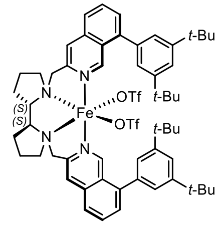

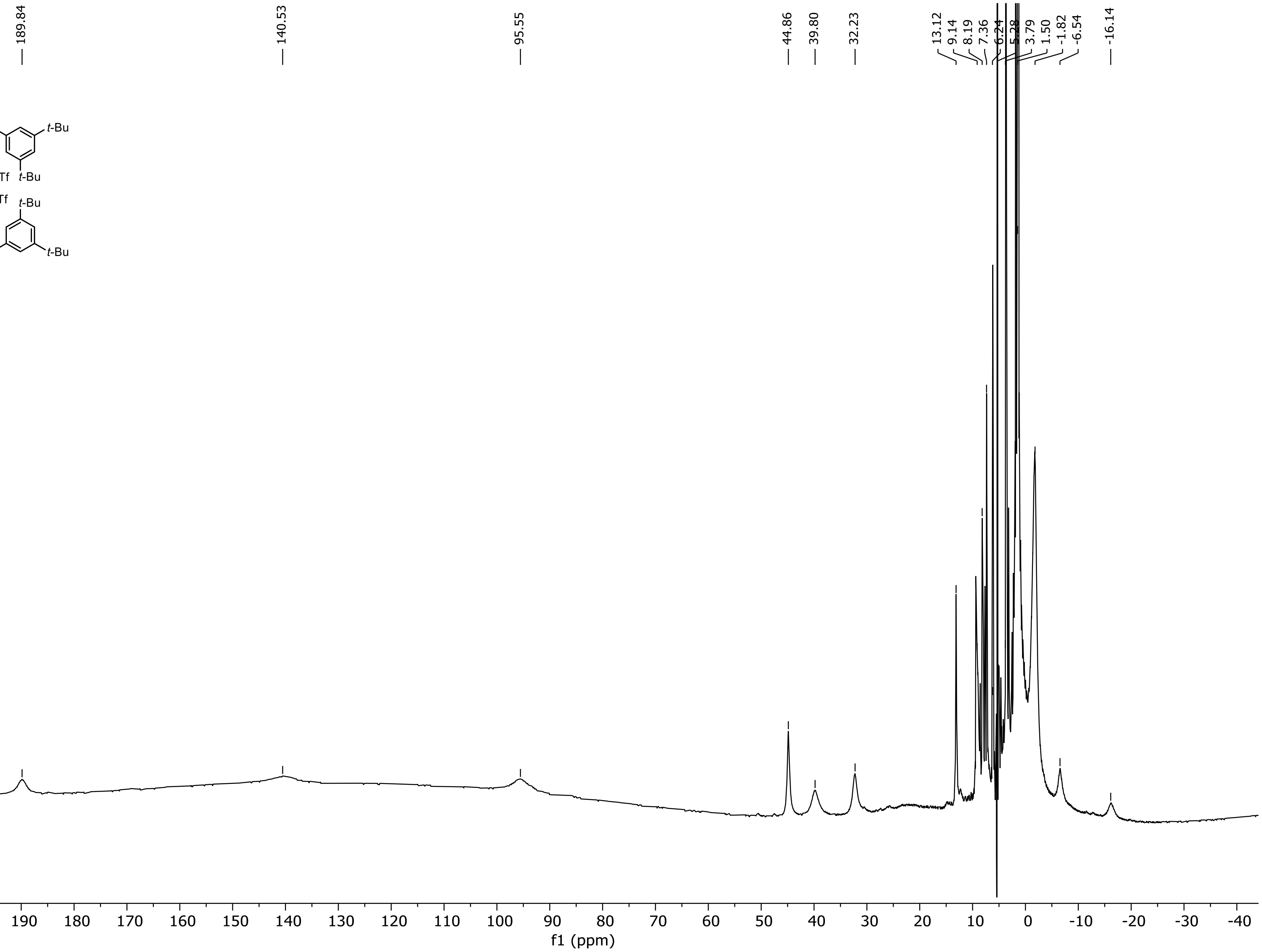

^{1}H-NMR spectrum of (*S*,*S*)-**Fe($^{3,5\text{-(di-CF3)}}$iQ$_2$dp)**: (400 MHz, CD_2Cl_2)

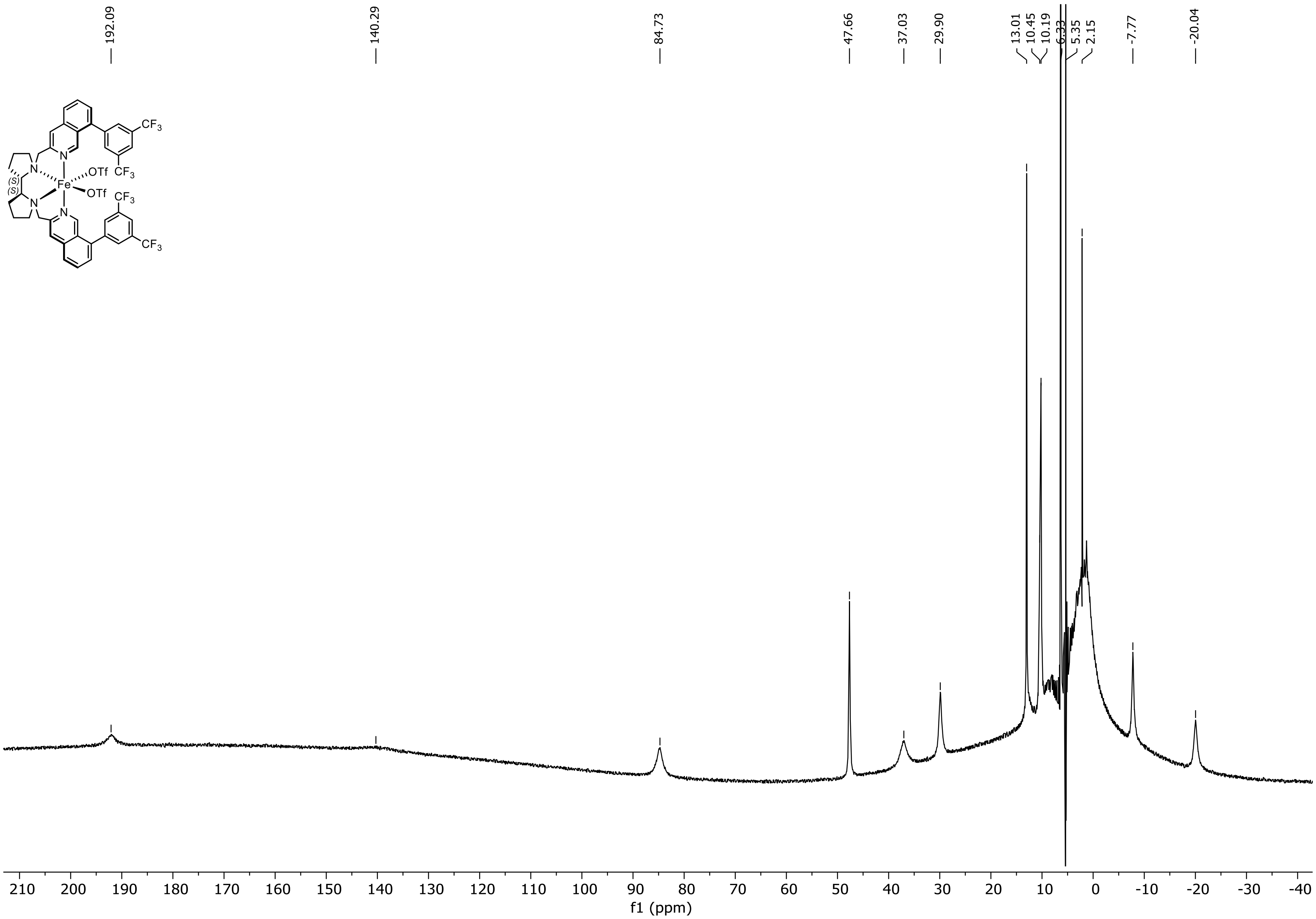

^{1}H-NMR spectrum of (*R*,*R*)-**Fe($^{3,5\text{-(di-CF3)}}$iQ$_2$dp)**: (500 MHz, CD_2Cl_2)

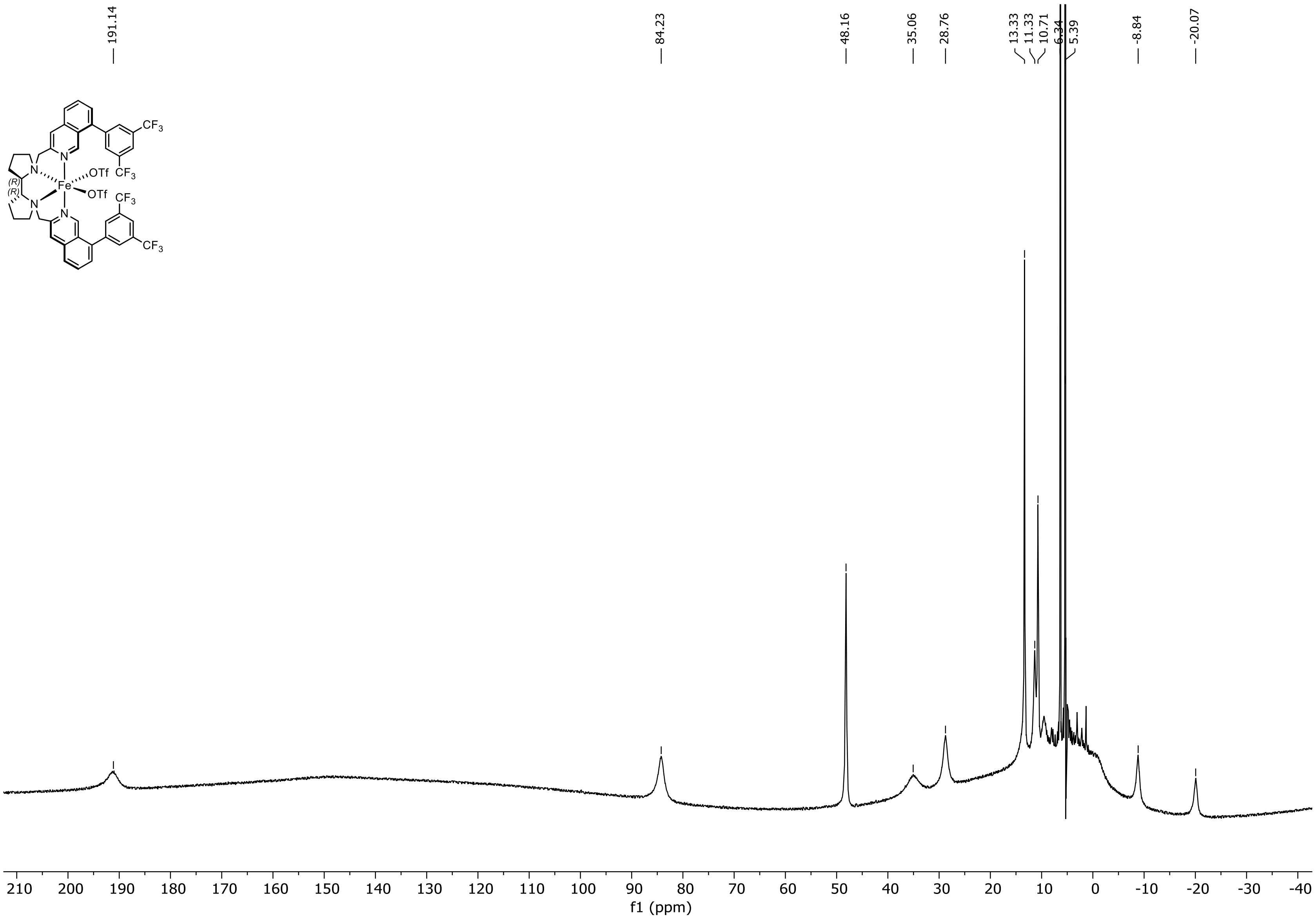

^{1}H-NMR spectrum of (*S*,*S*)-**Fe($^{2,4,6\text{-(tri-iPr)}}iQ_2$dp)**: (400 MHz, CD_2Cl_2)

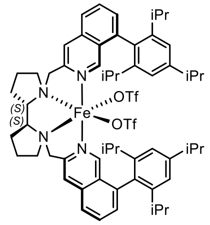

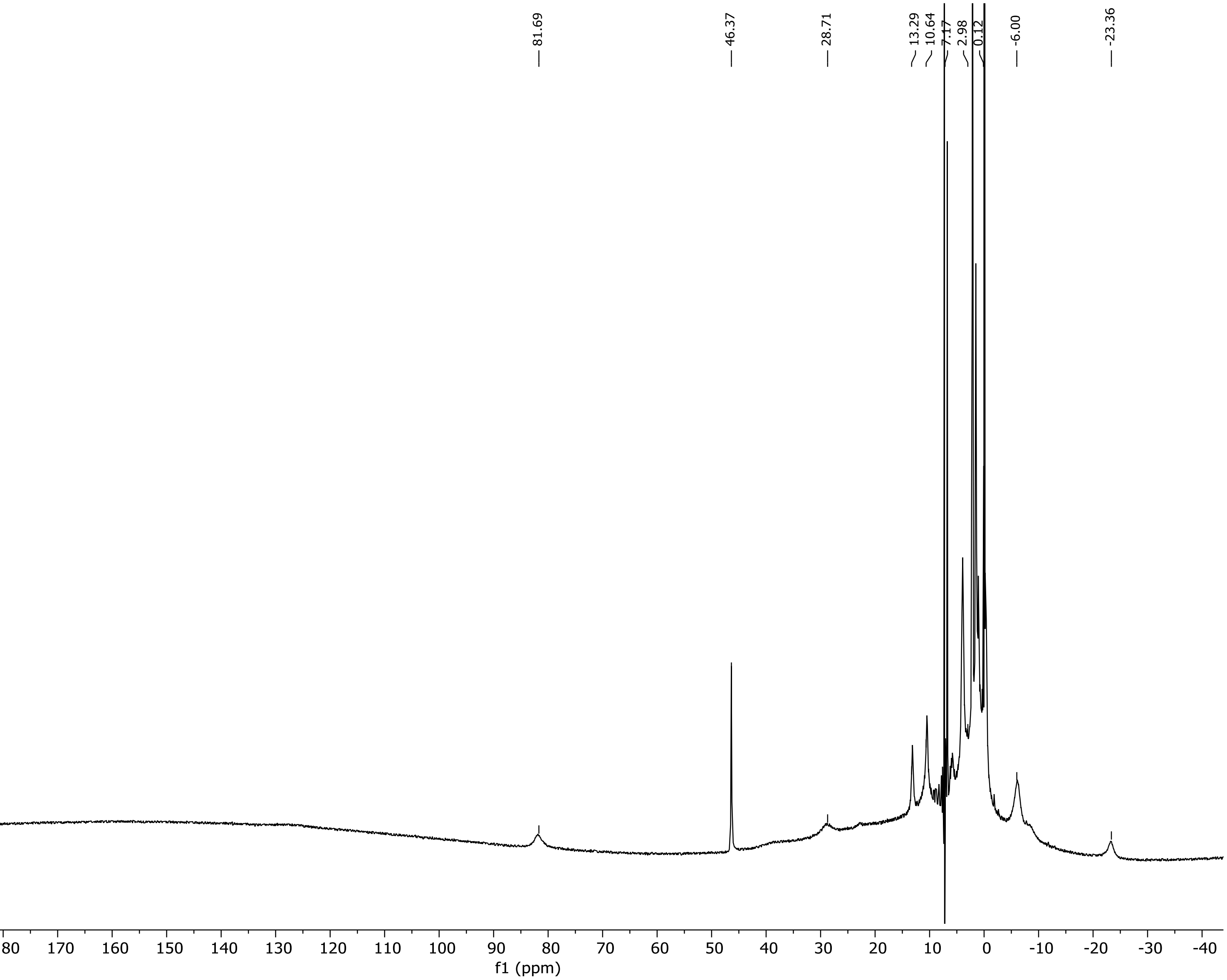

^{1}H-NMR spectrum of (*S*,*S*)-**Fe($^{3,4,5\text{-(tri-F)}}$iQ$_2$dp)**: (500 MHz, CD_2Cl_2)

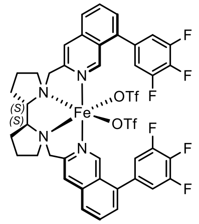

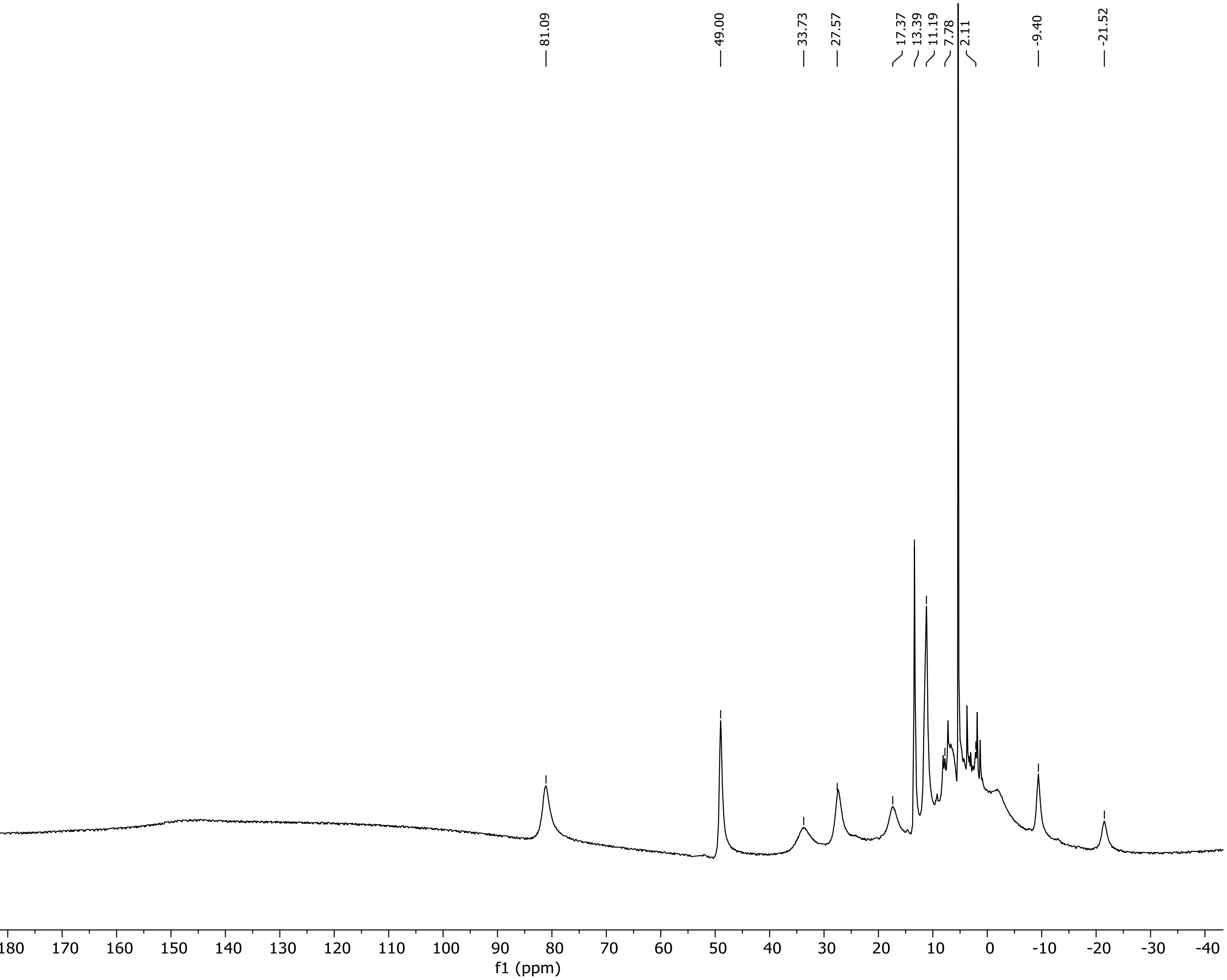

^{1}H-NMR spectrum of compound **11a**: (500 MHz, $CDCl_3$)

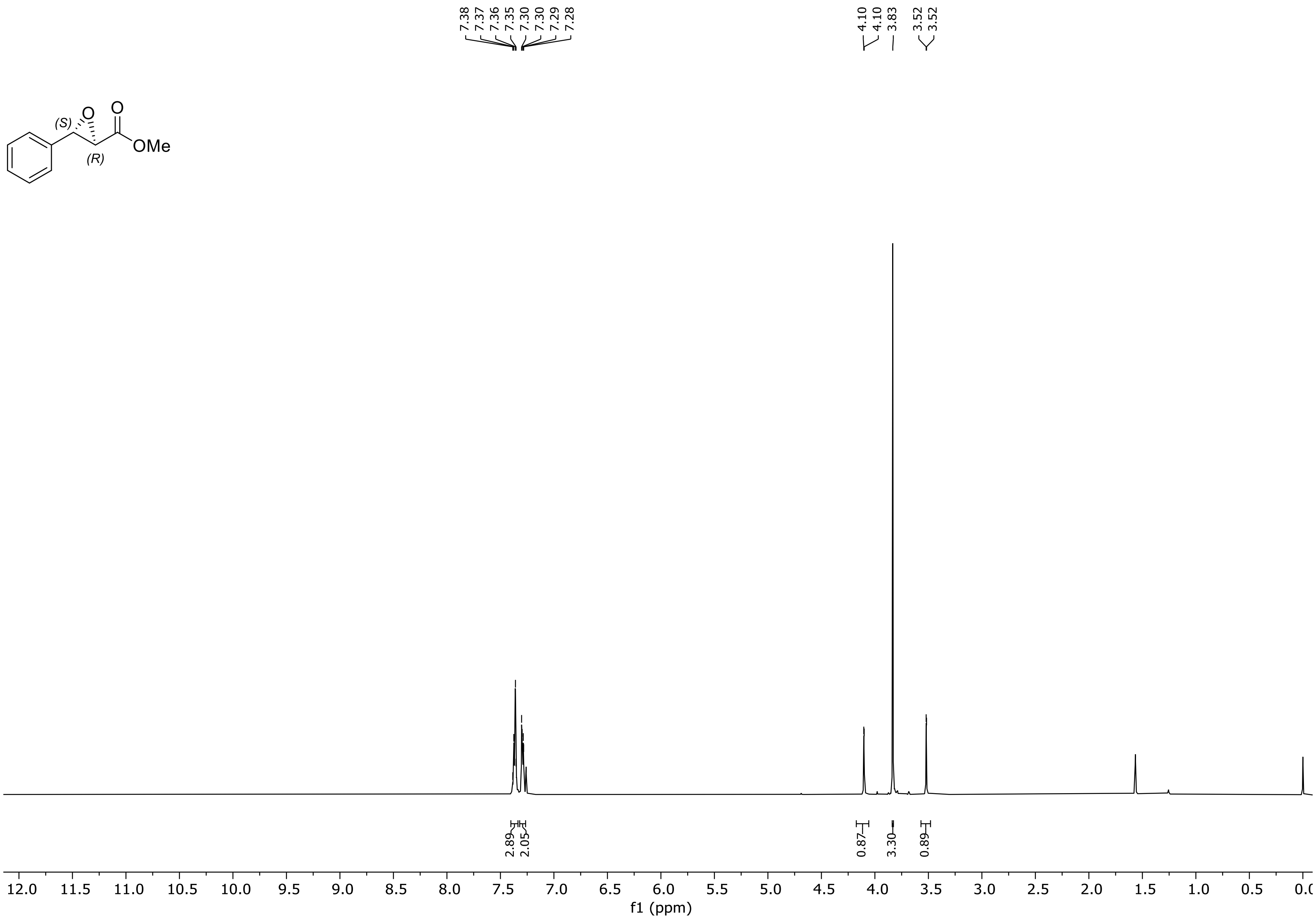

^{13}C-NMR spectrum of compound **11a**: (125 MHz, $CDCl_3$)

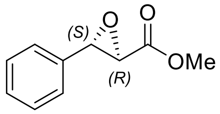

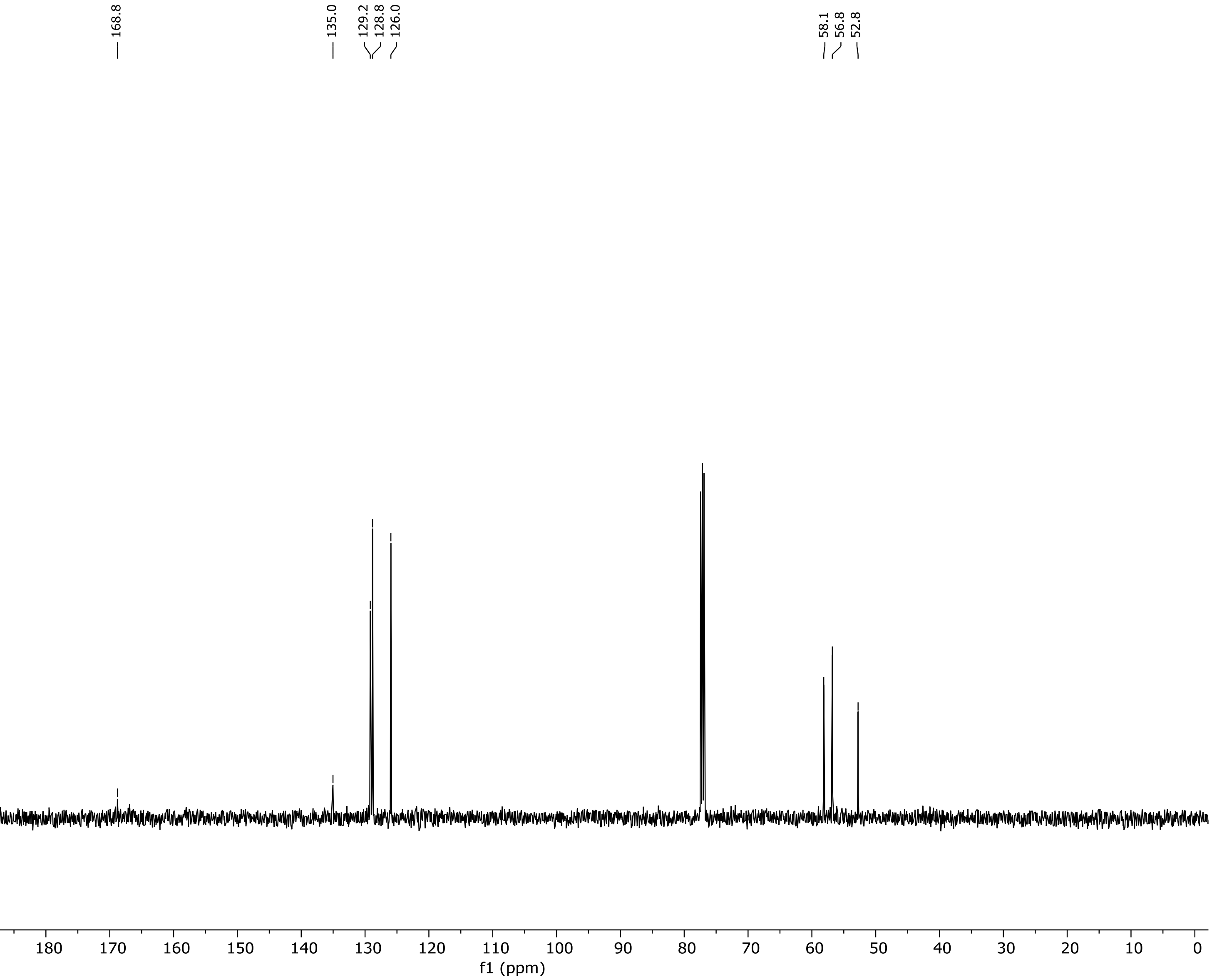

^{1}H-NMR spectrum of compound **11b**: (500 MHz, $CDCl_3$)

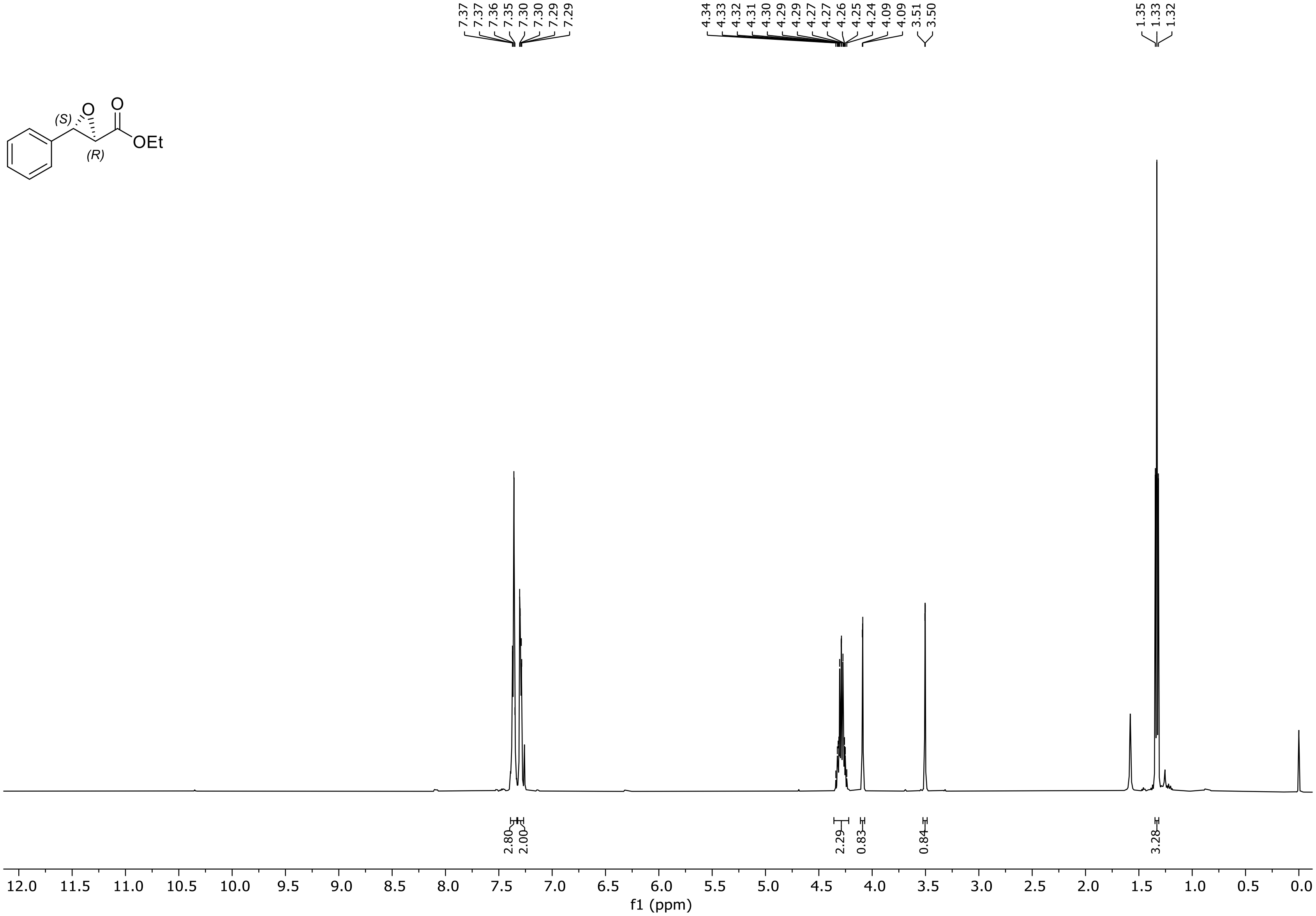

^{13}C-NMR spectrum of compound **11b**: (125 MHz, $CDCl_3$)

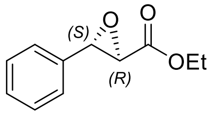

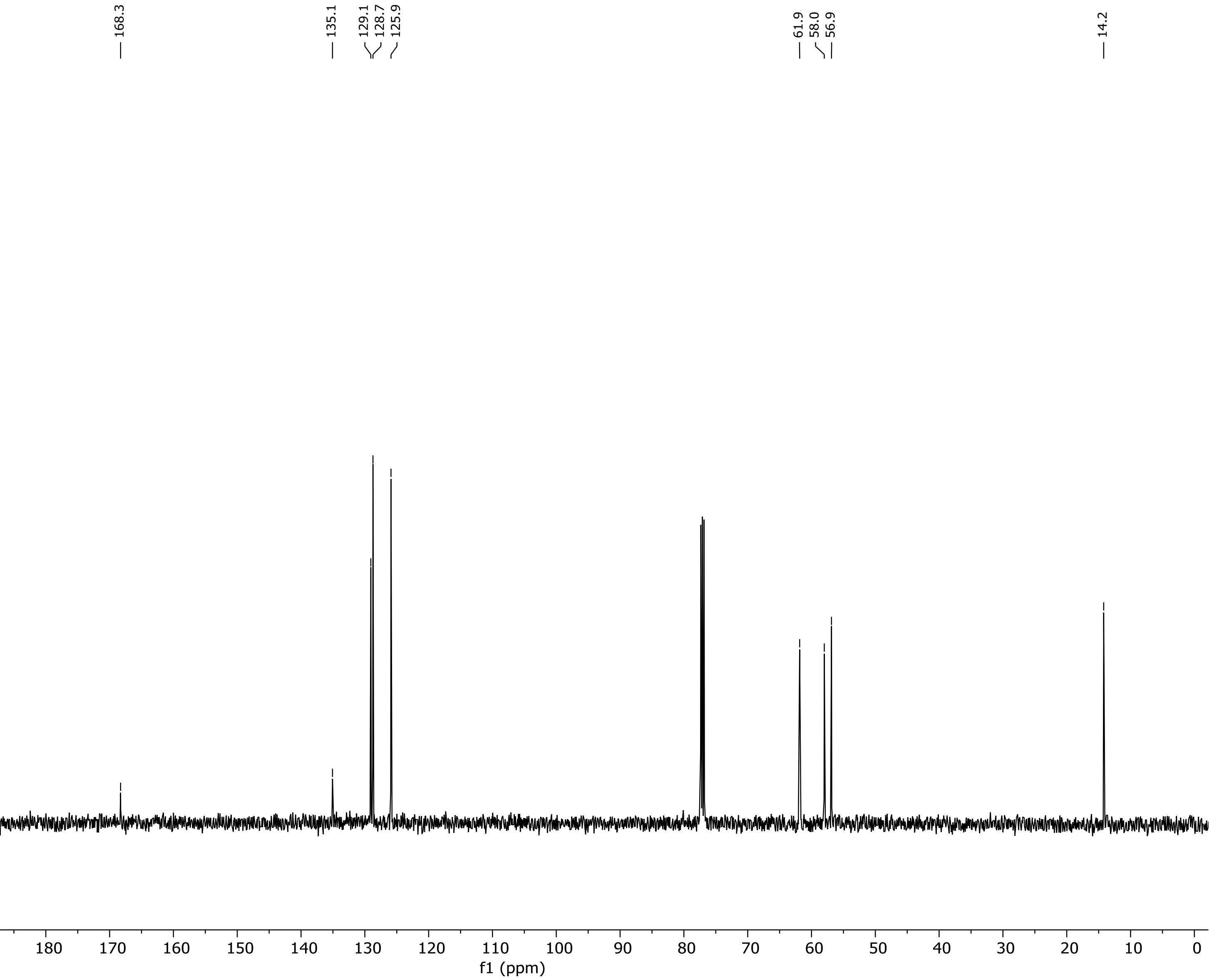

^{1}H-NMR spectrum of compound **11c**: (500 MHz, $CDCl_3$)

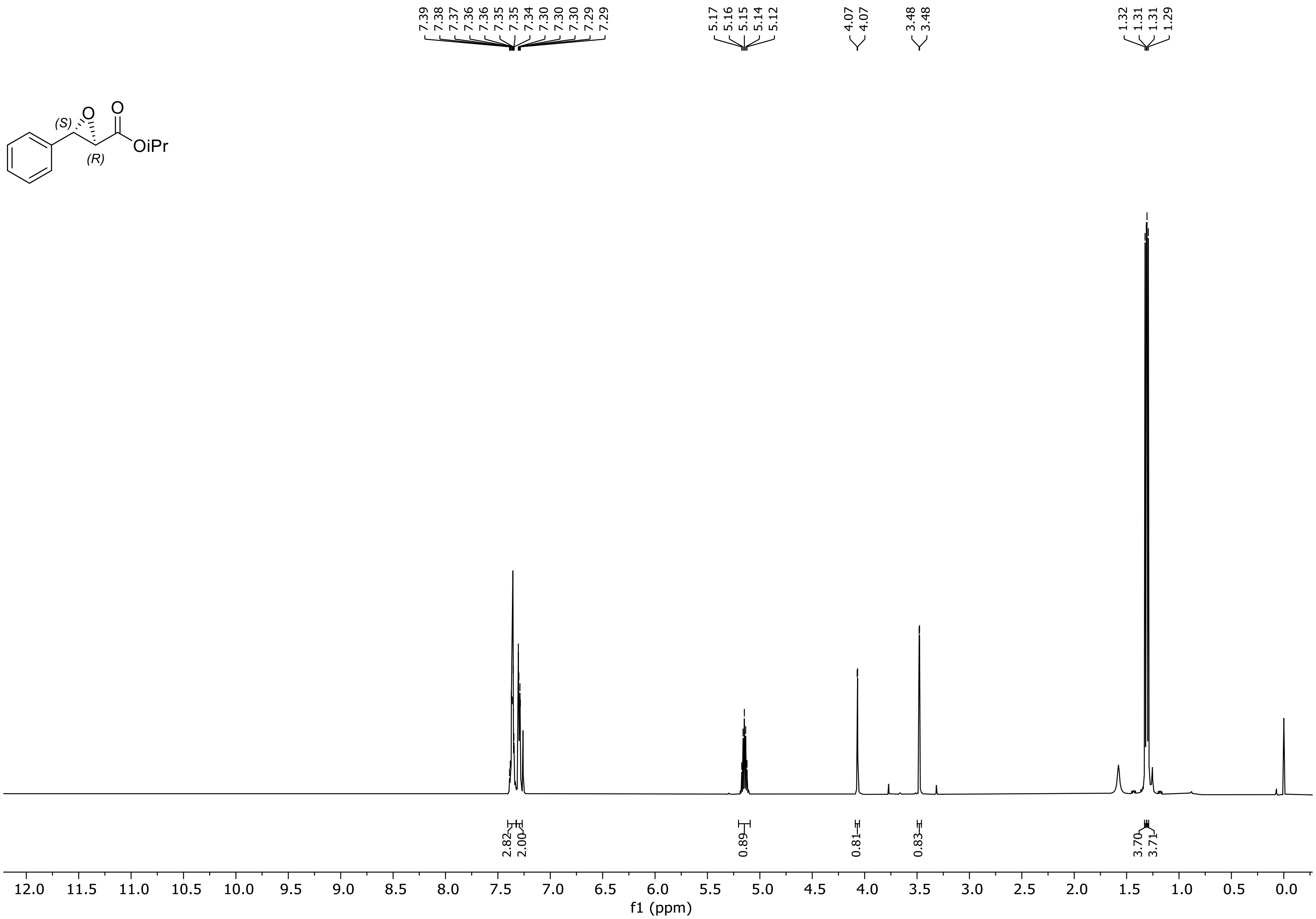

^{13}C-NMR spectrum of compound **11c**: (125 MHz, $CDCl_3$)

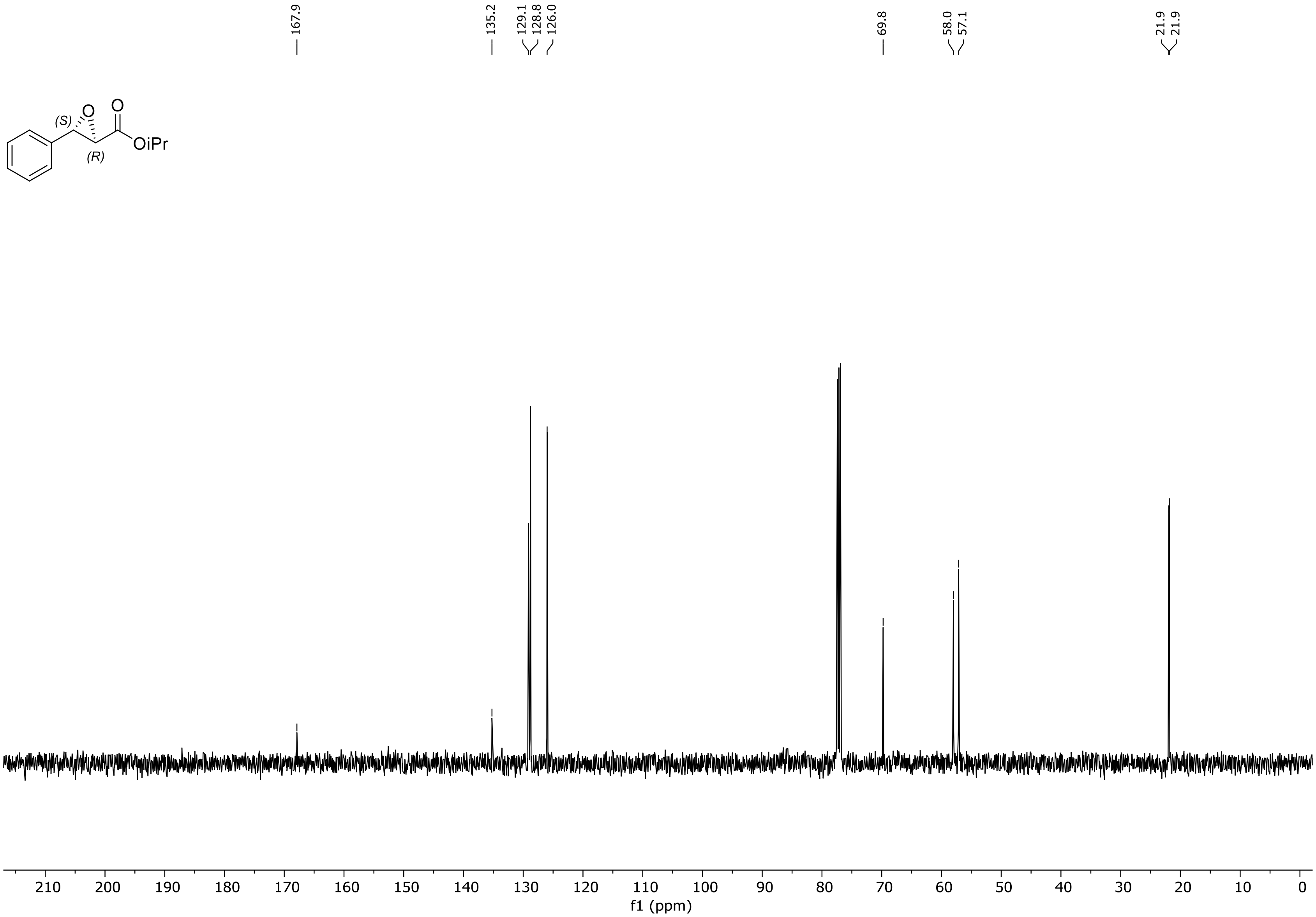

^{1}H-NMR spectrum of compound **11d**: (400 MHz, $CDCl_3$)

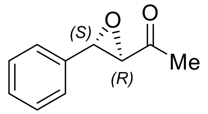

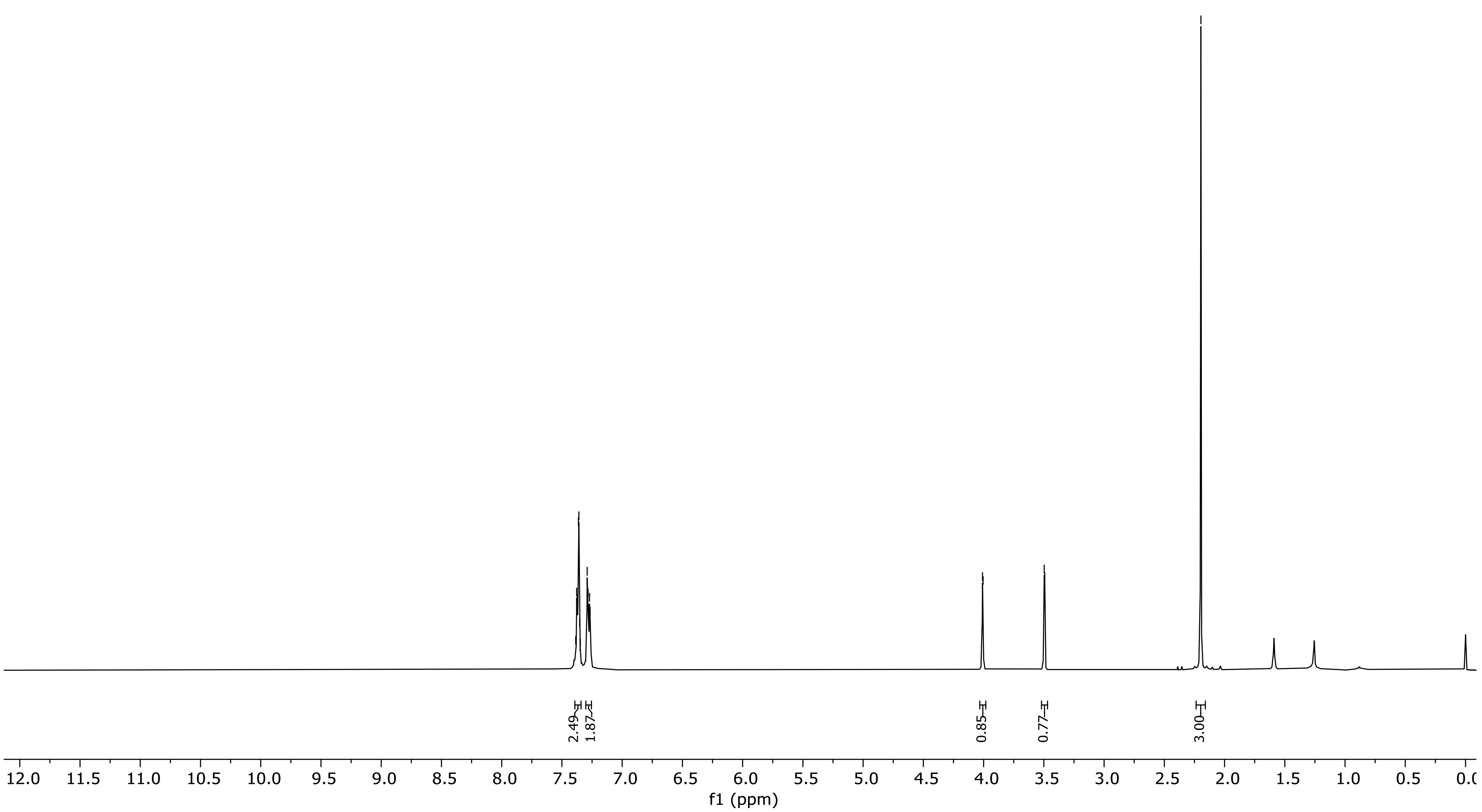

^{13}C-NMR spectrum of compound **11d**: (100 MHz, $CDCl_3$)

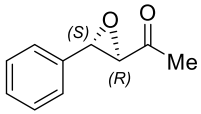

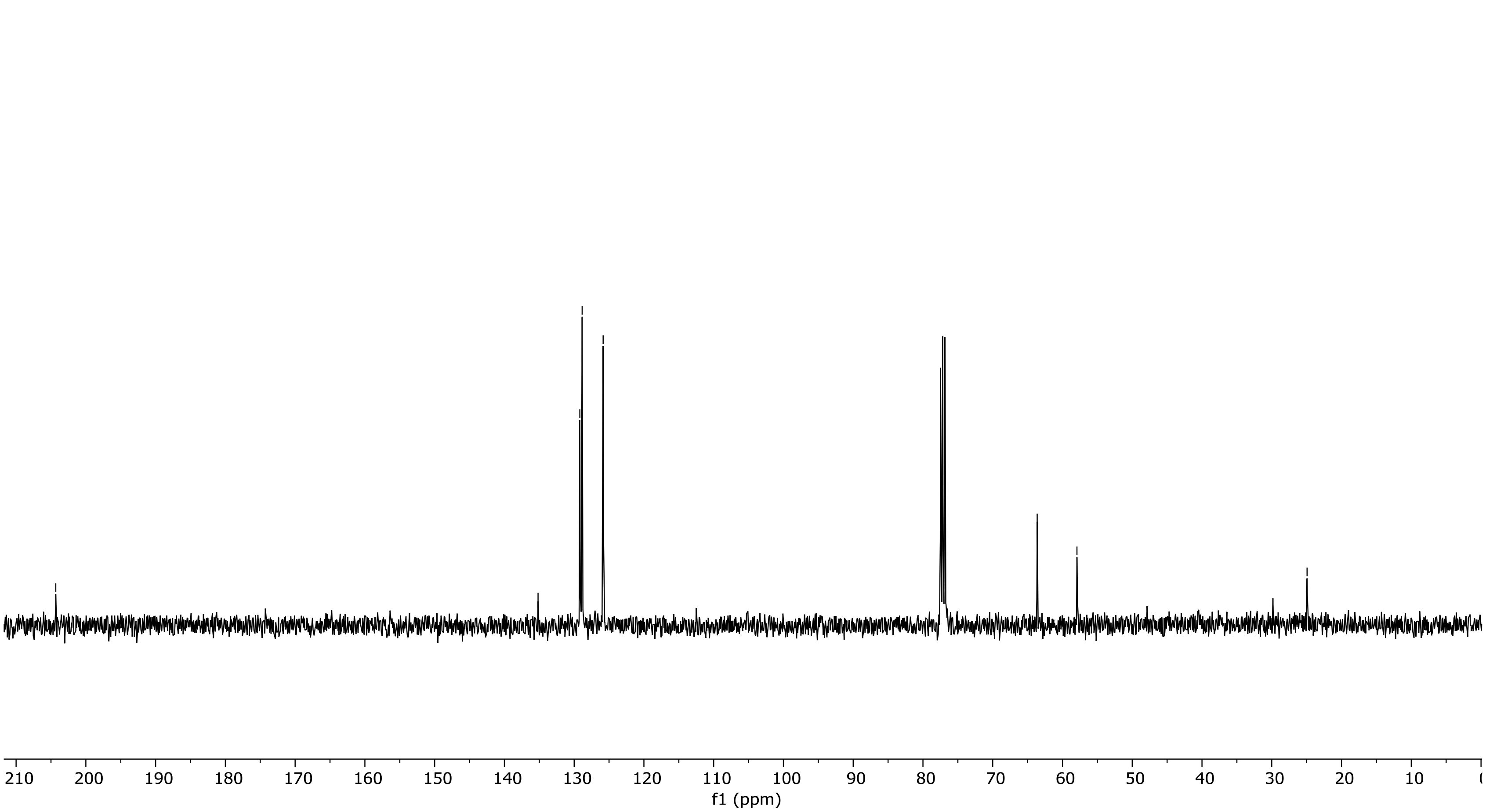

^{1}H-NMR spectrum of compound **11e**: (400 MHz, $CDCl_3$)

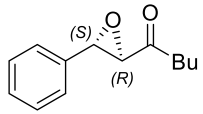

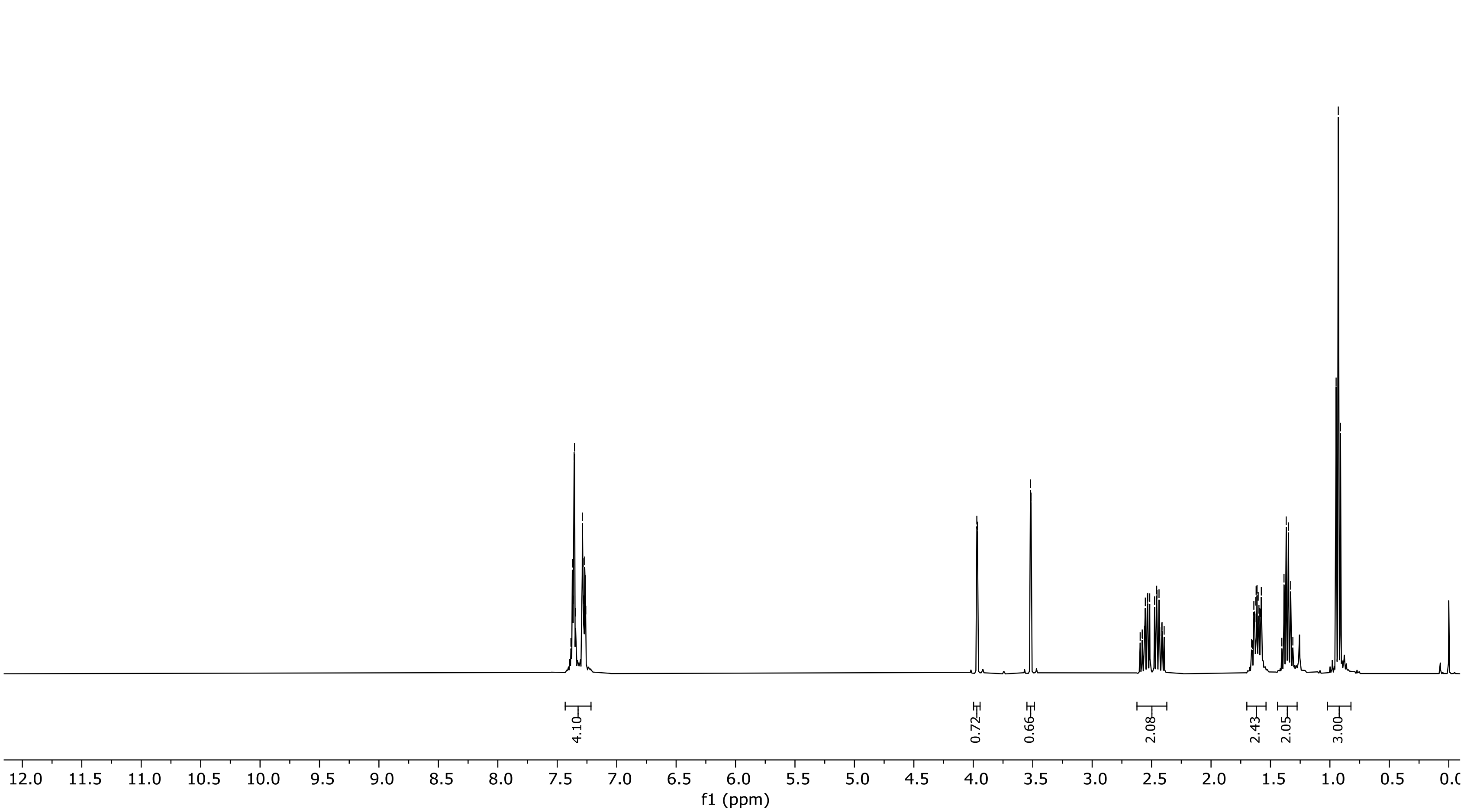

^{13}C-NMR spectrum of compound **11e**: (100 MHz, $CDCl_3$)

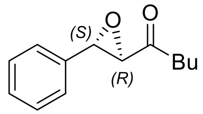

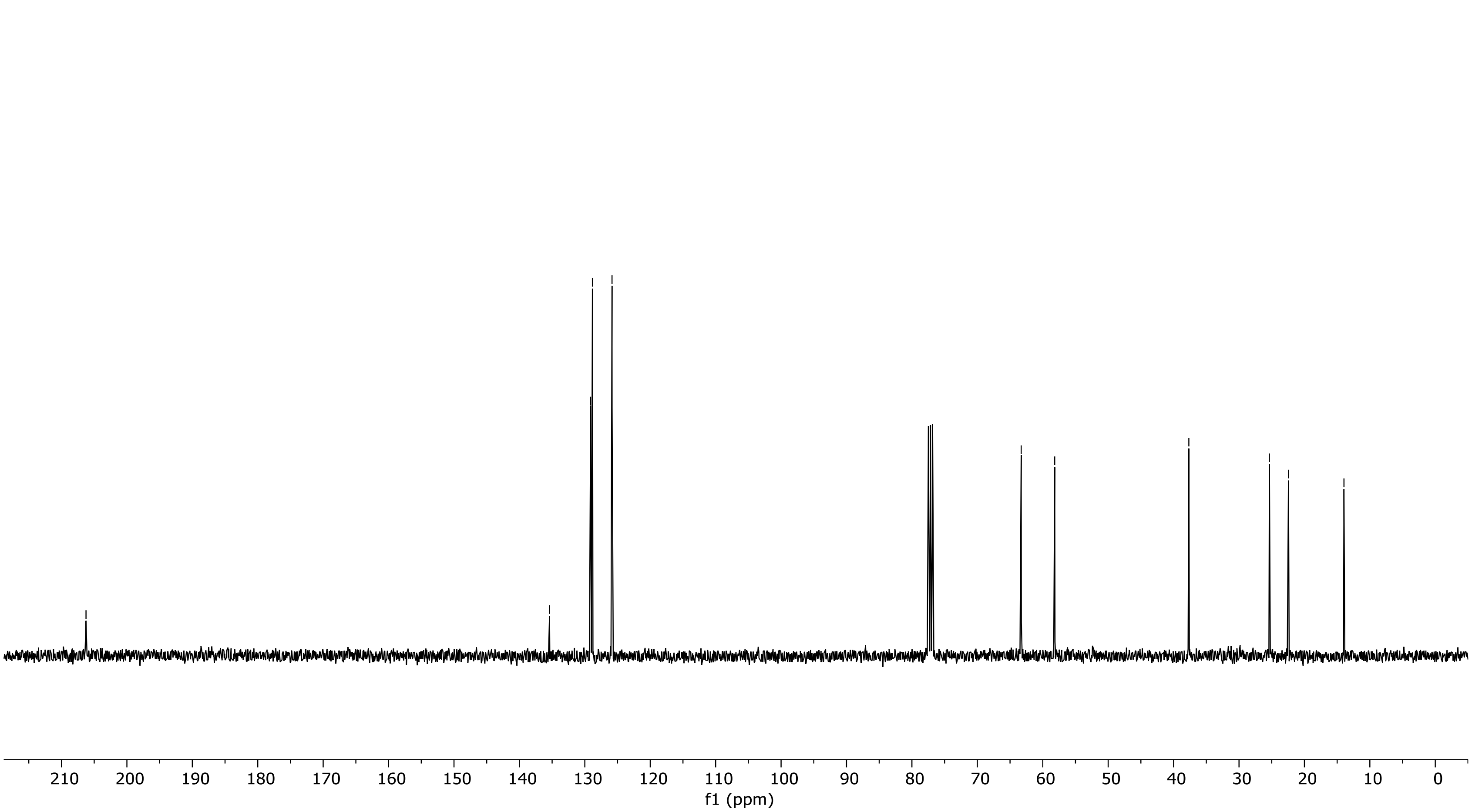

^{1}H-NMR spectrum of compound **11f**: (500 MHz, $CDCl_3$)

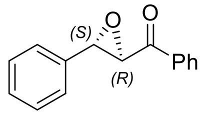

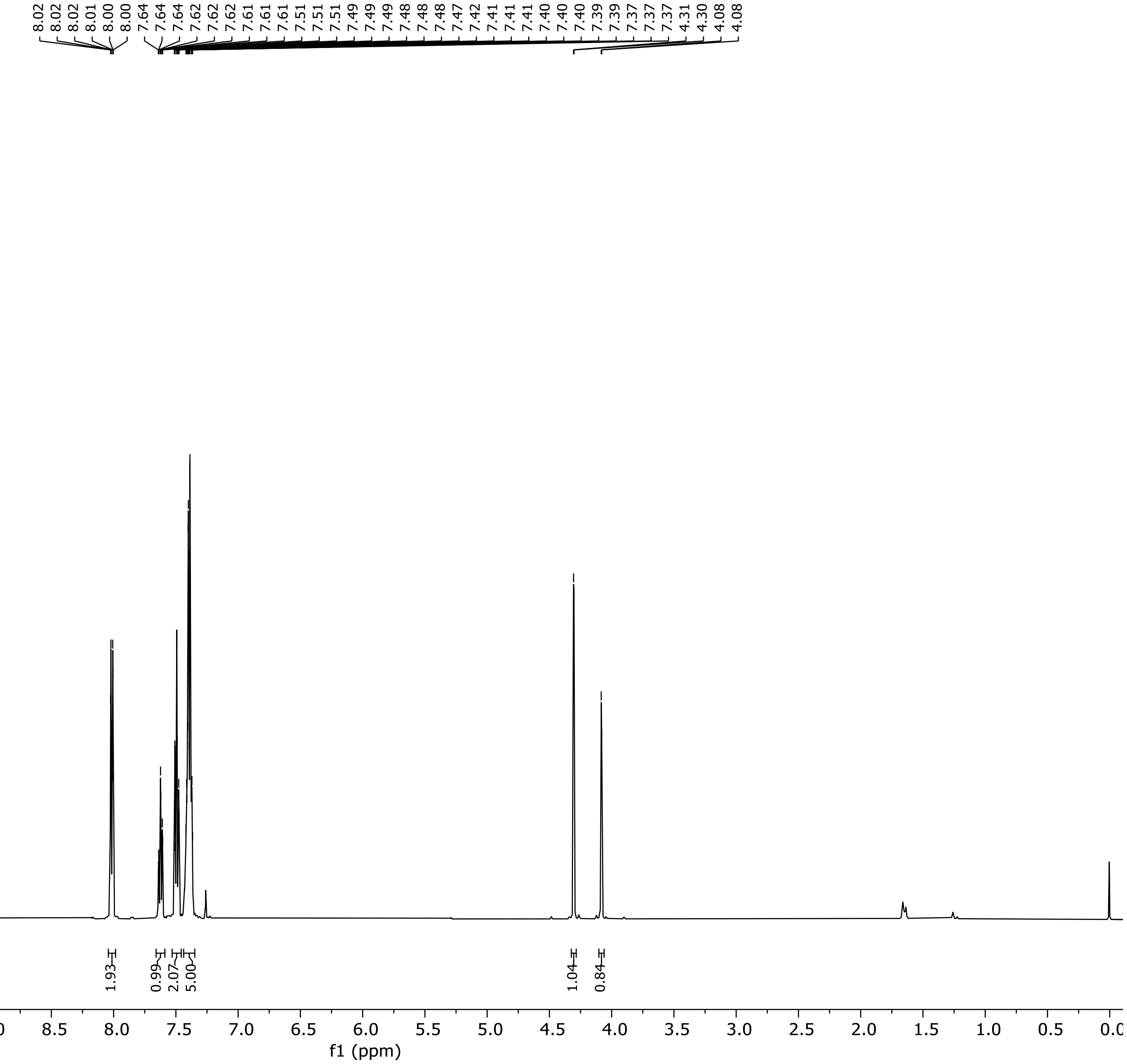

^{13}C-NMR spectrum of compound **11f**: (125 MHz, $CDCl_3$)

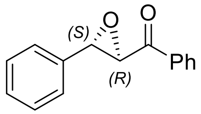

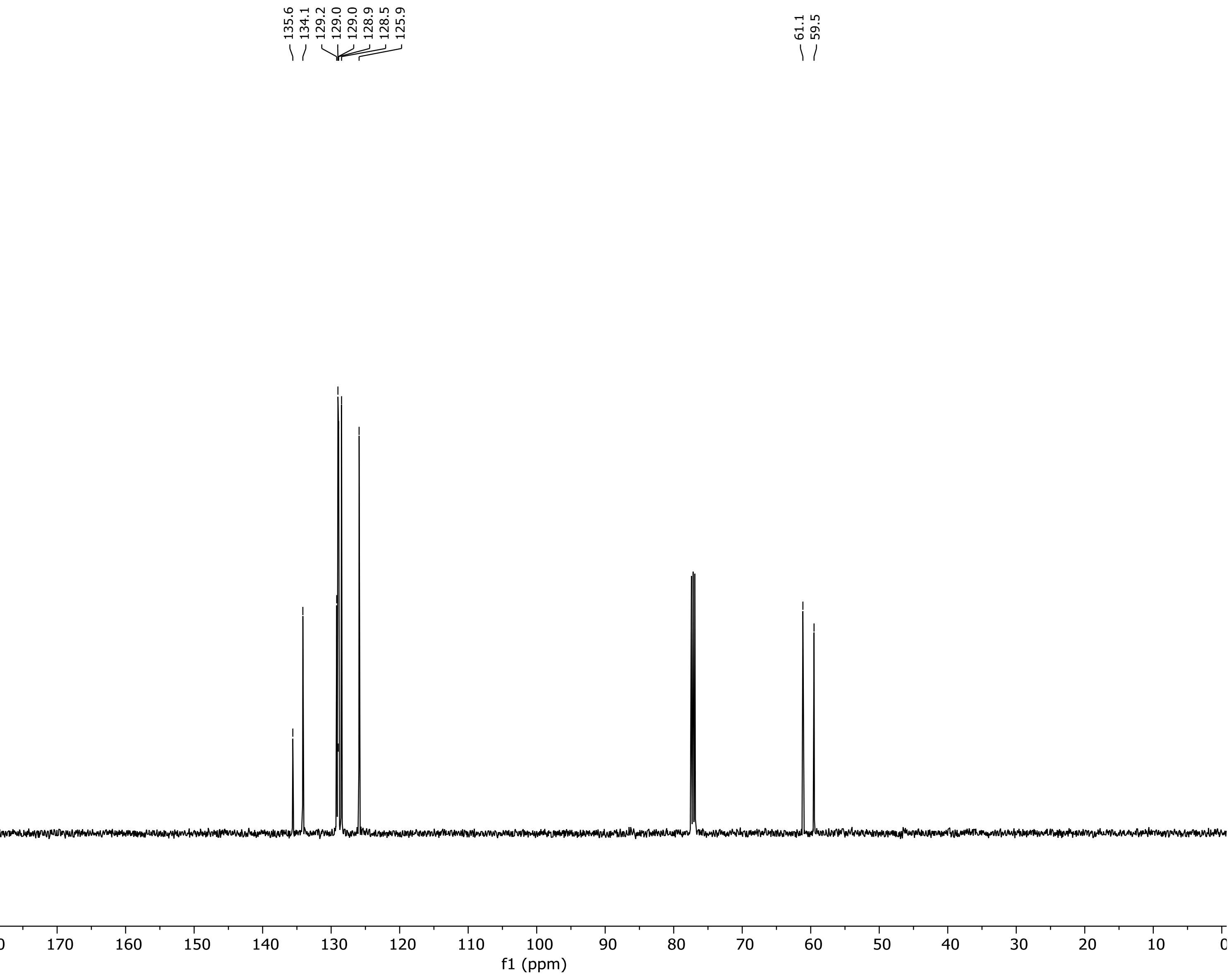

^{1}H-NMR spectrum of compound **11g**: (400 MHz, $CDCl_3$)

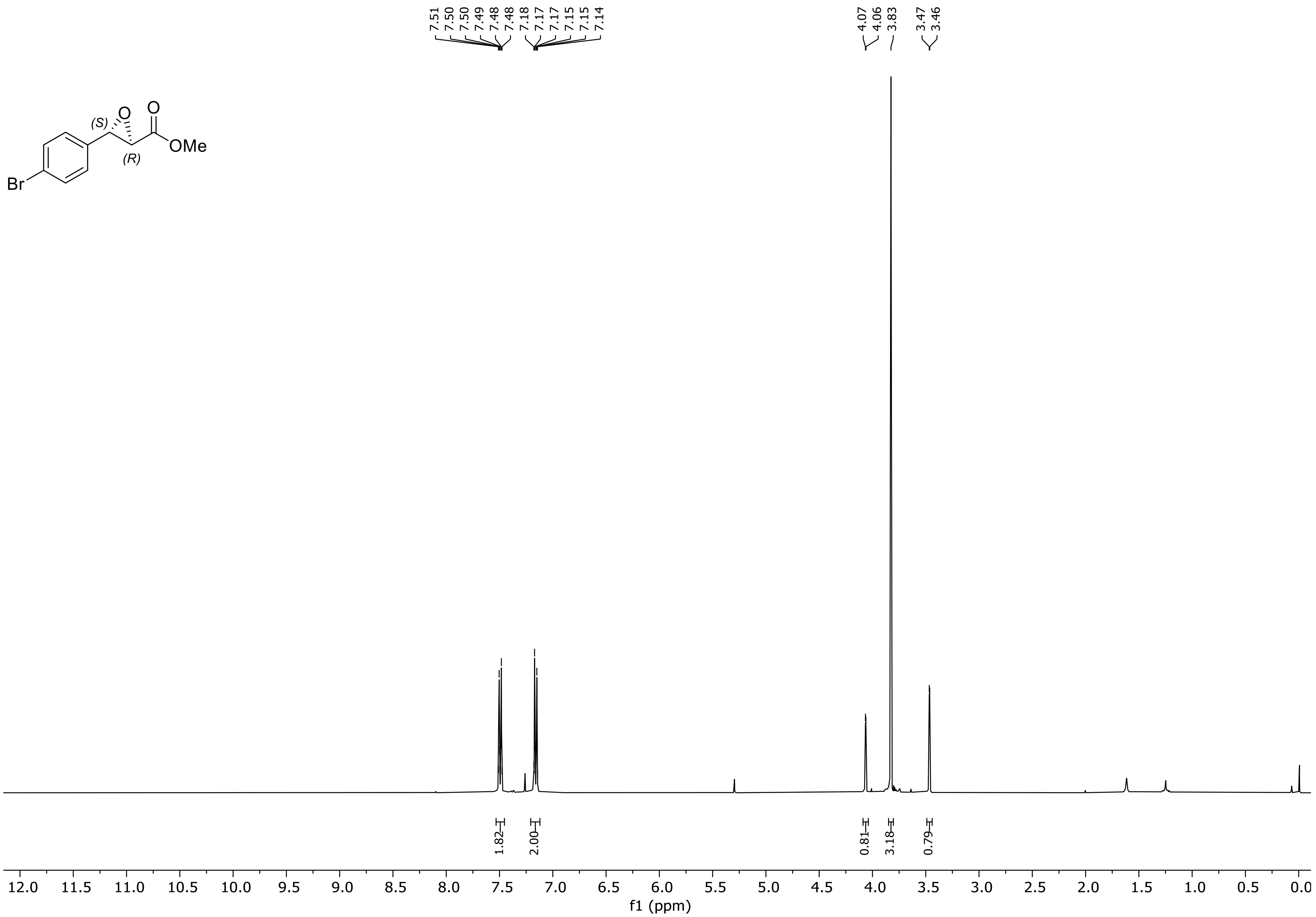

^{13}C-NMR spectrum of compound **11g**: (100 MHz, $CDCl_3$)

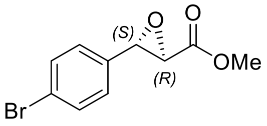

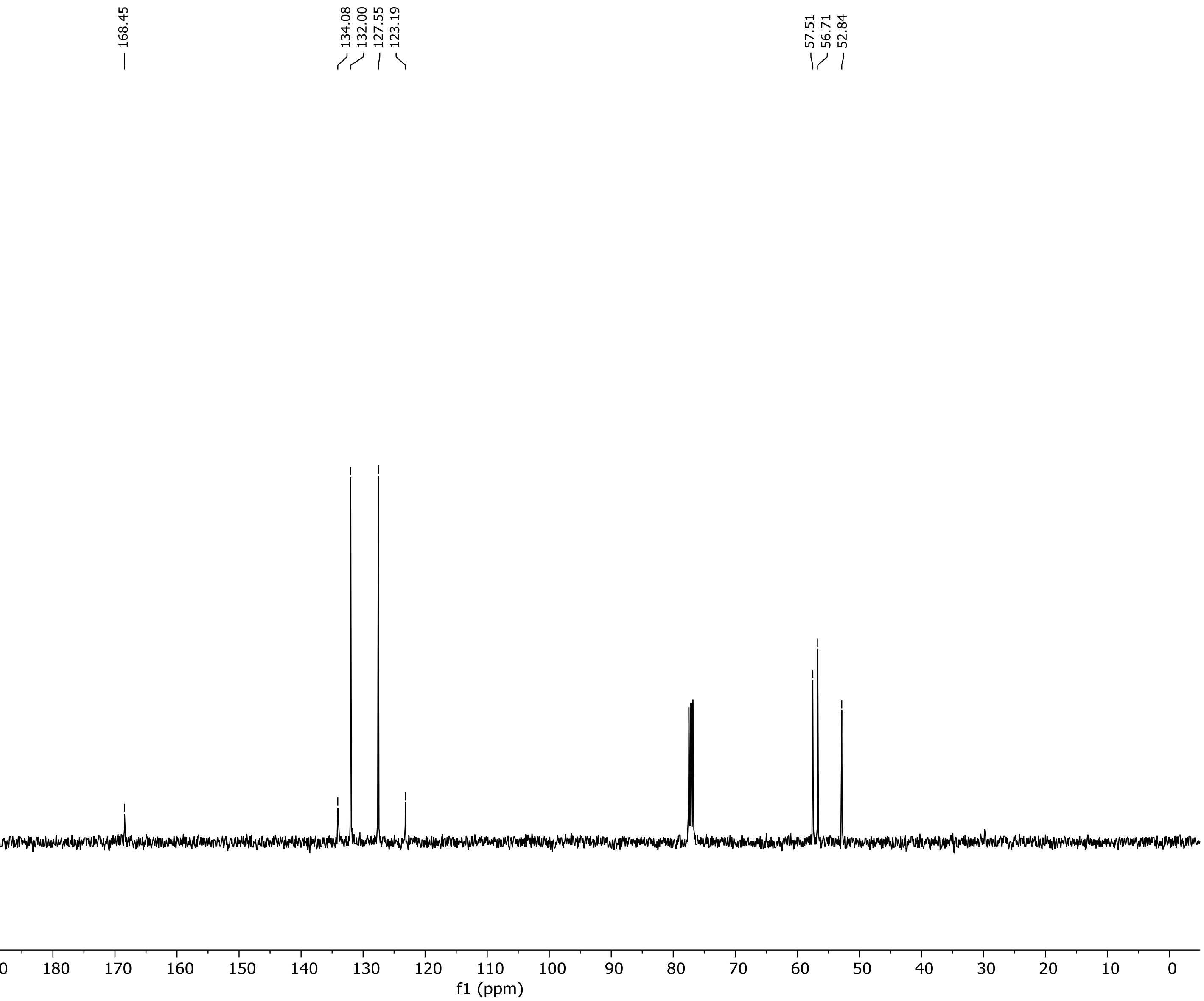

^{1}H-NMR spectrum of compound **11h**: (400 MHz, $CDCl_3$)

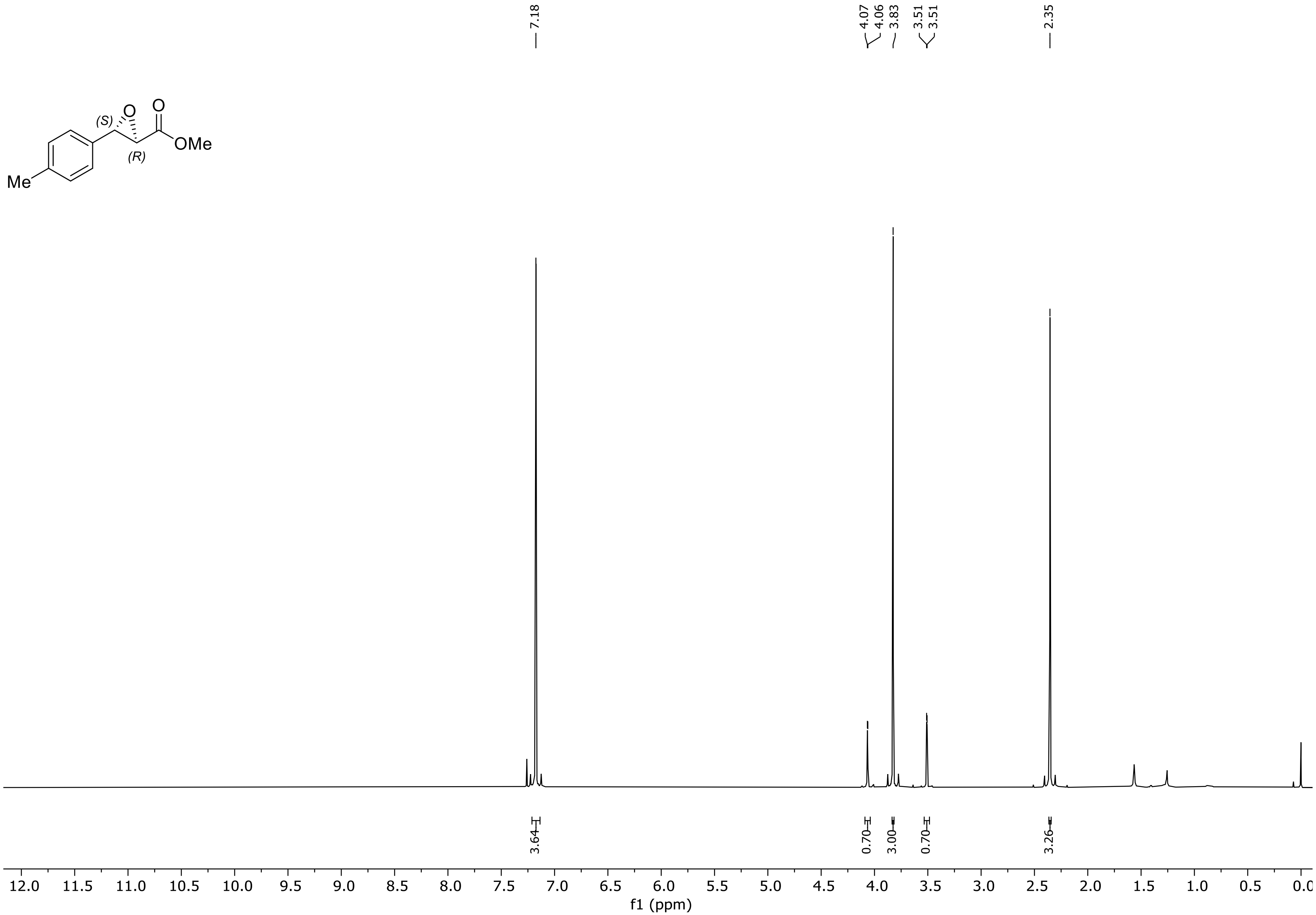

^{13}C-NMR spectrum of compound **11h**: (100 MHz, $CDCl_3$)

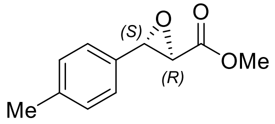

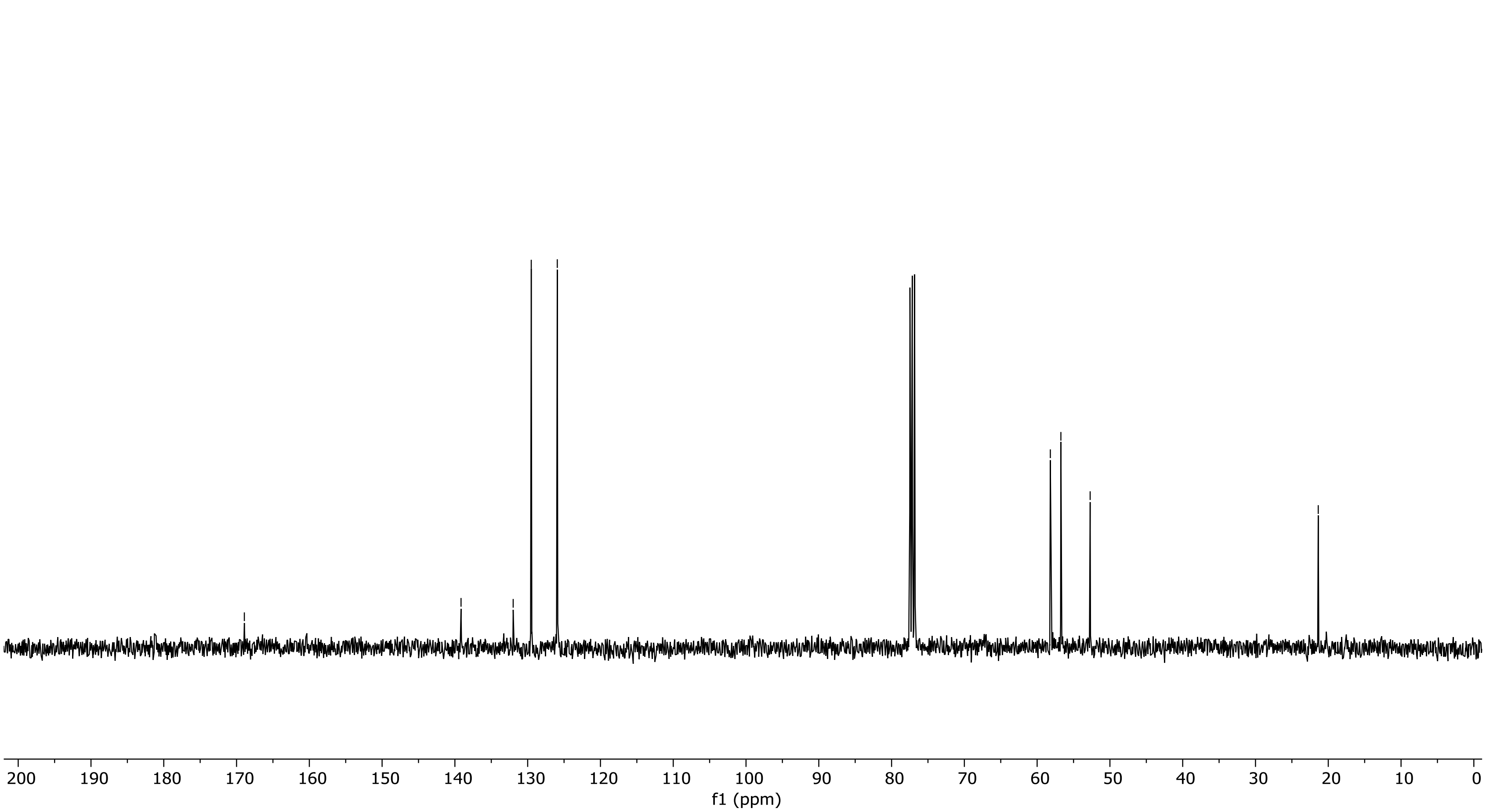

^{1}H-NMR spectrum of compound **12ia**: (400 MHz, $CDCl_3$)

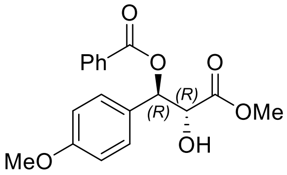

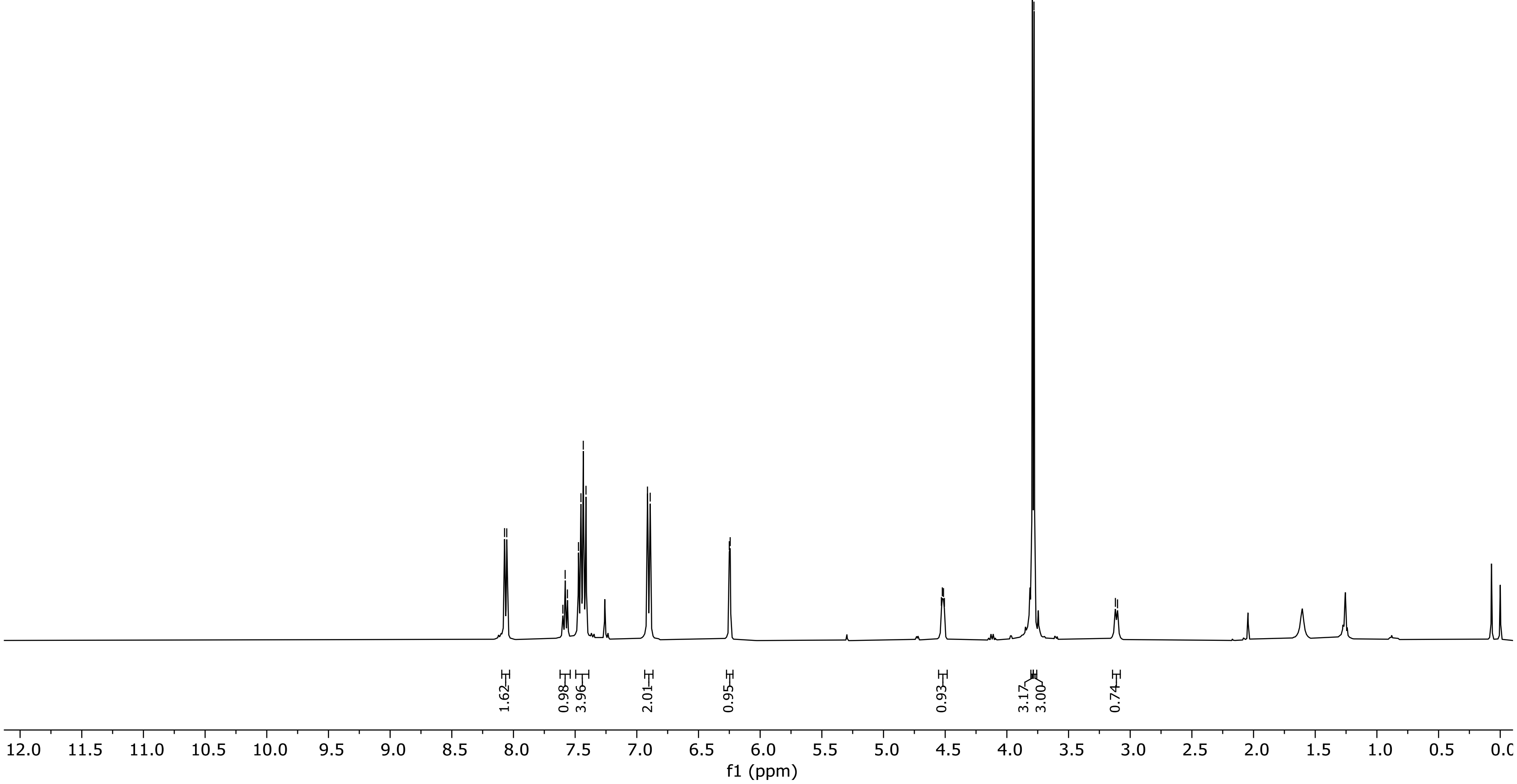

^{13}C-NMR spectrum of compound **12ia**: (100 MHz, $CDCl_3$)

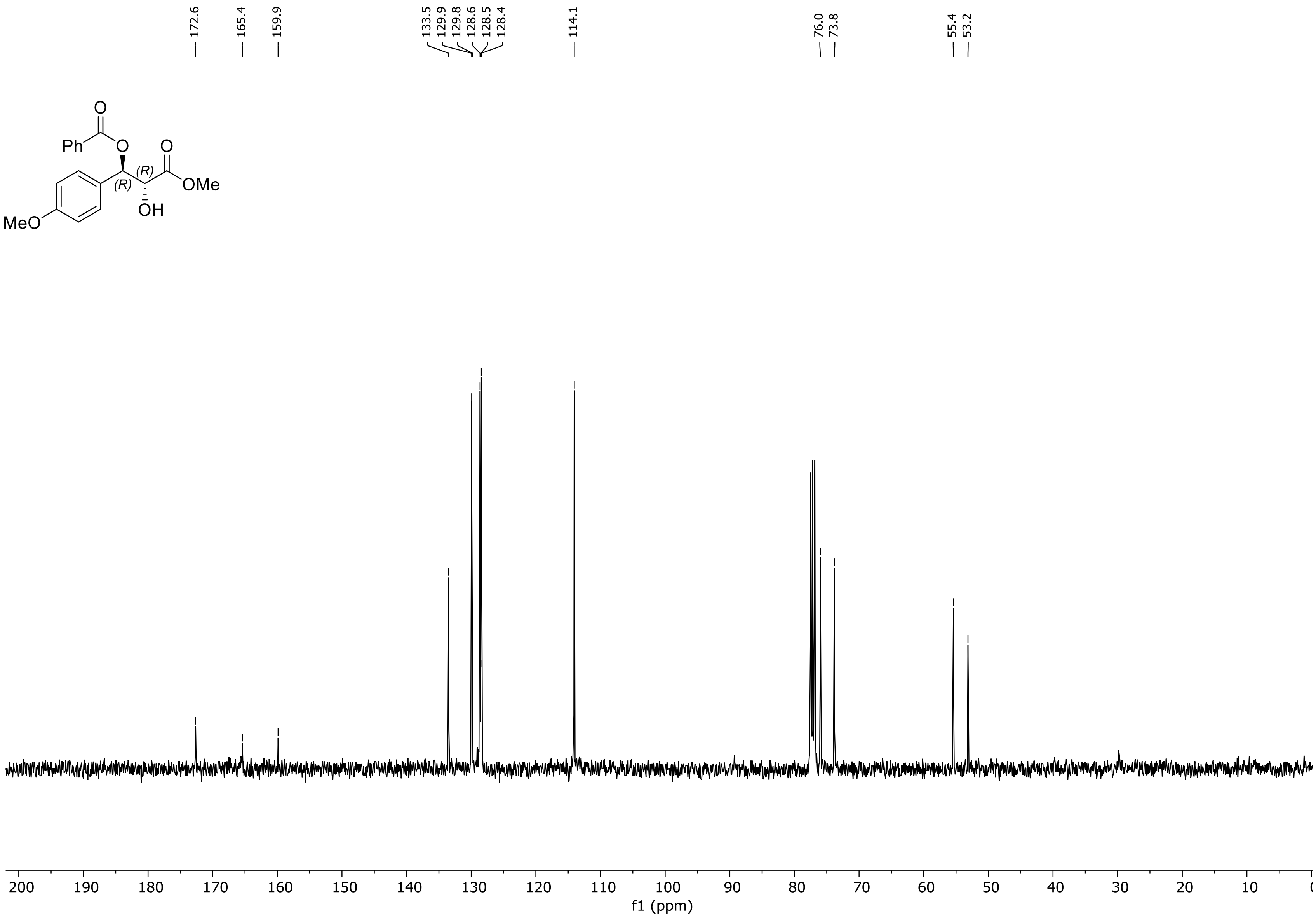

^{1}H-NMR spectrum of compound **12ib**: (400 MHz, $CDCl_3$)

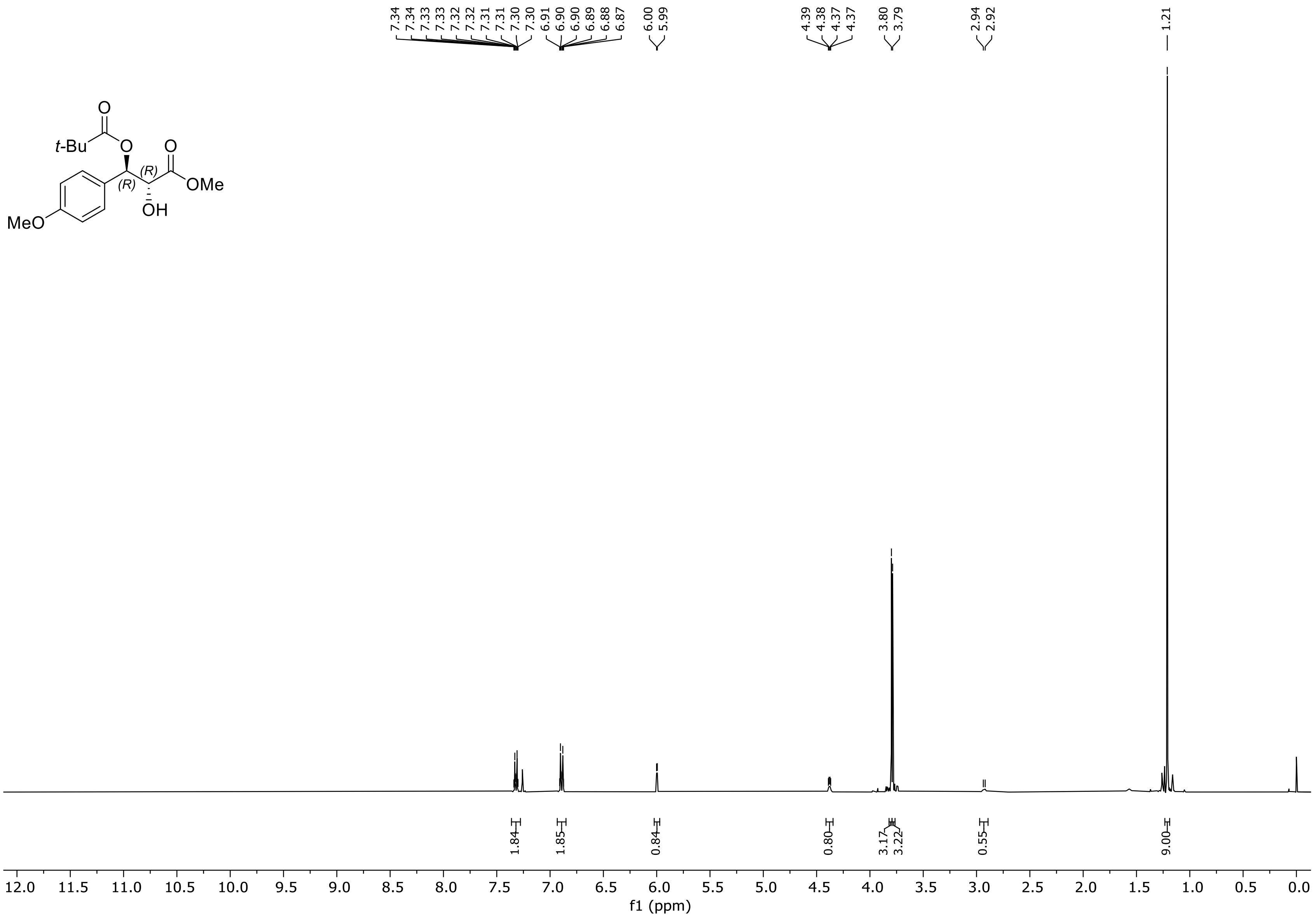

^{13}C-NMR spectrum of compound **12ib**: (100 MHz, $CDCl_3$)

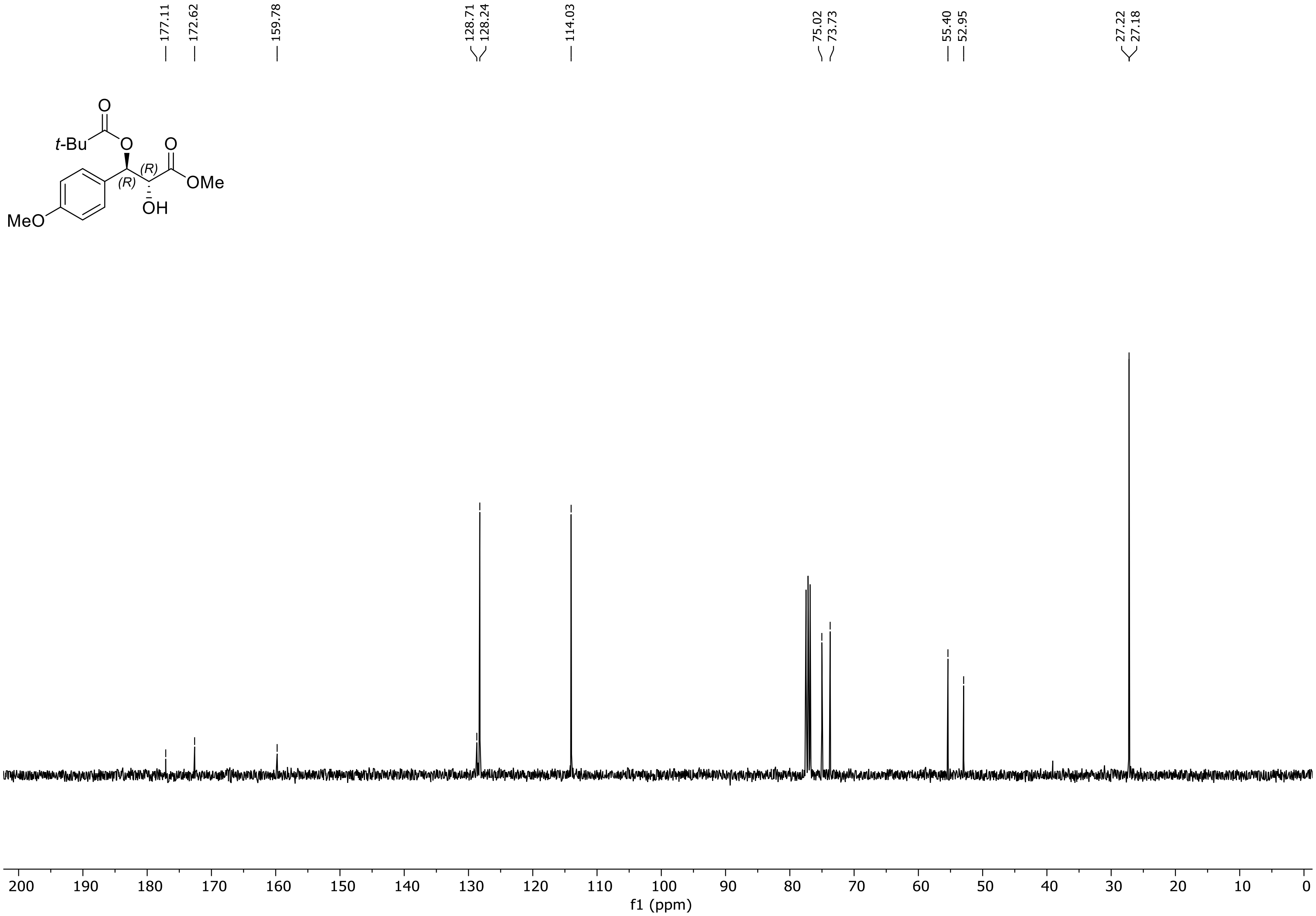

^{1}H-NMR spectrum of compound **12ic**: (500 MHz, $CDCl_3$)

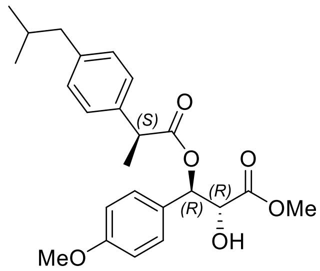

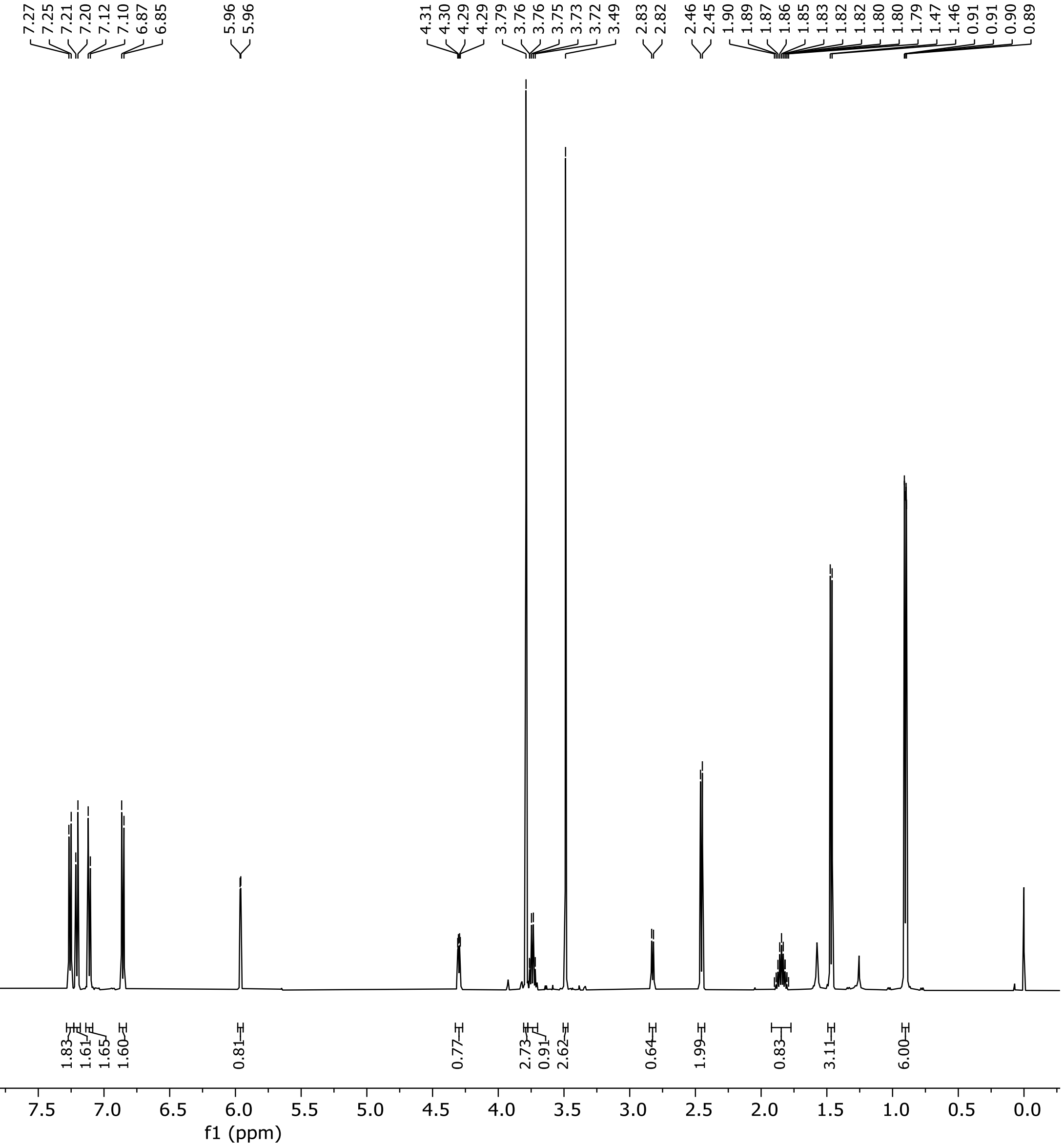

^{13}C-NMR spectrum of compound **12ic**: (125 MHz, $CDCl_3$)

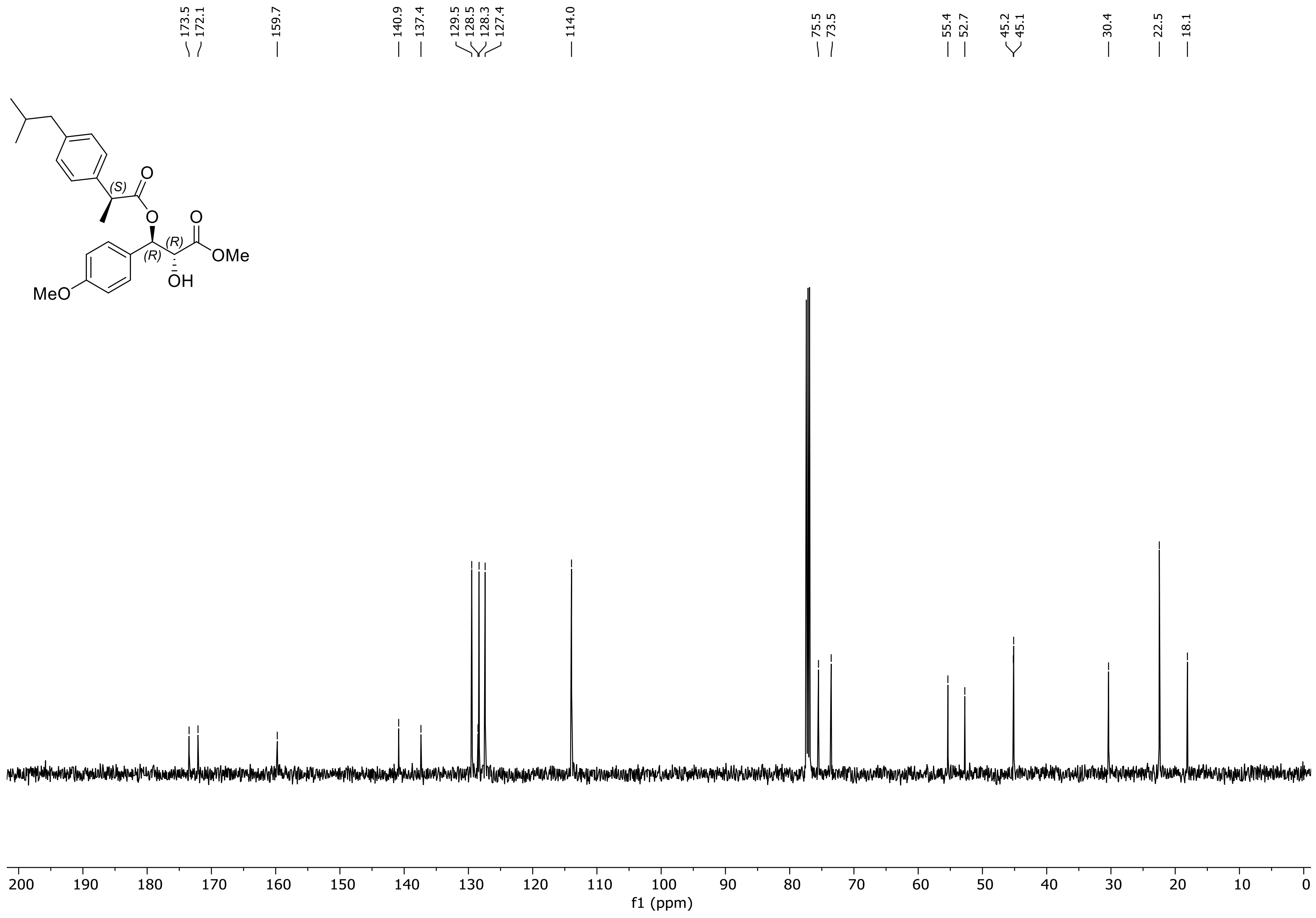

^{1}H-NMR spectrum of compound **12j**: (400 MHz, $CDCl_3$)

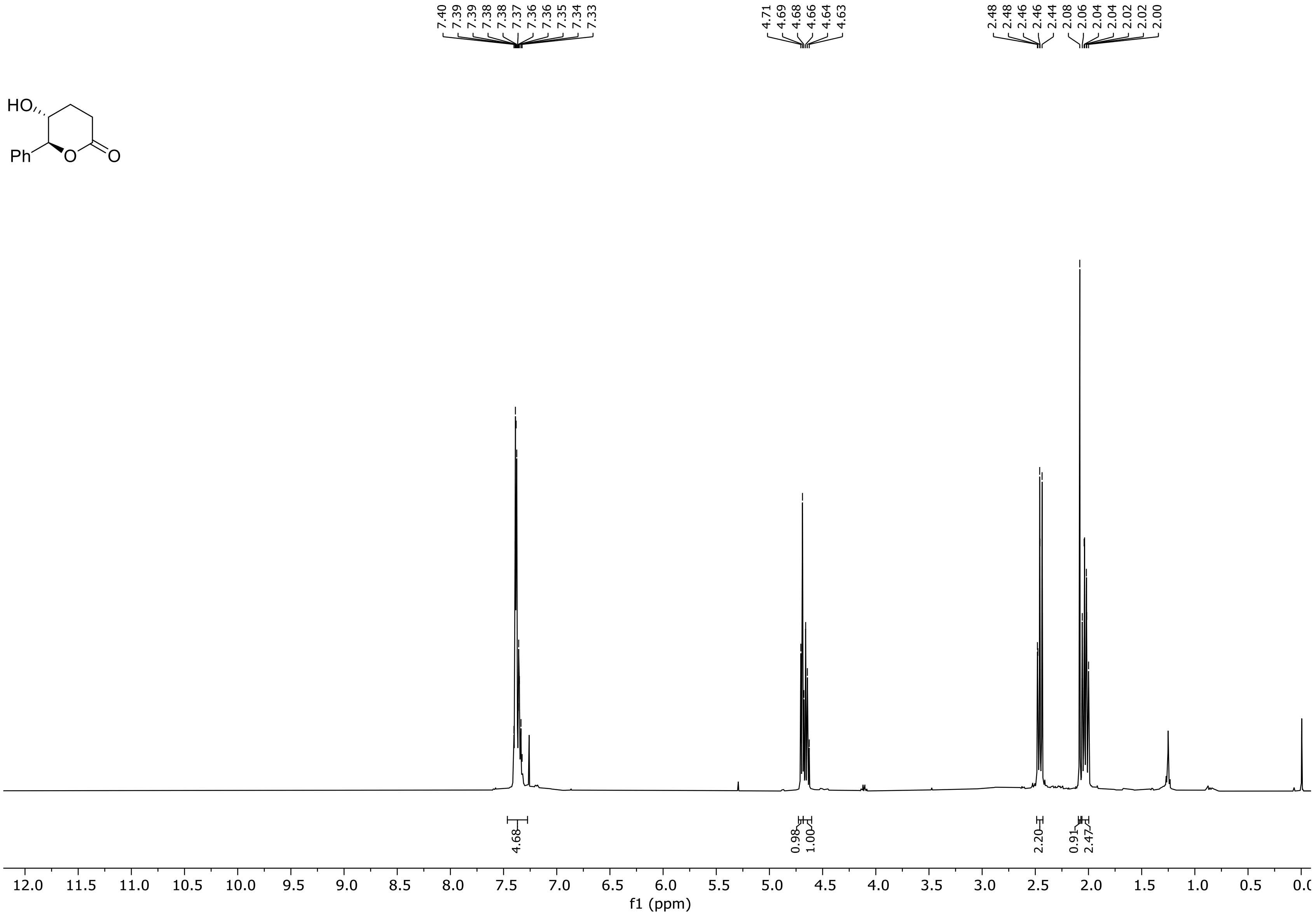

^{13}C-NMR spectrum of compound **12j**: (100 MHz, $CDCl_3$)

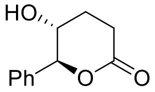

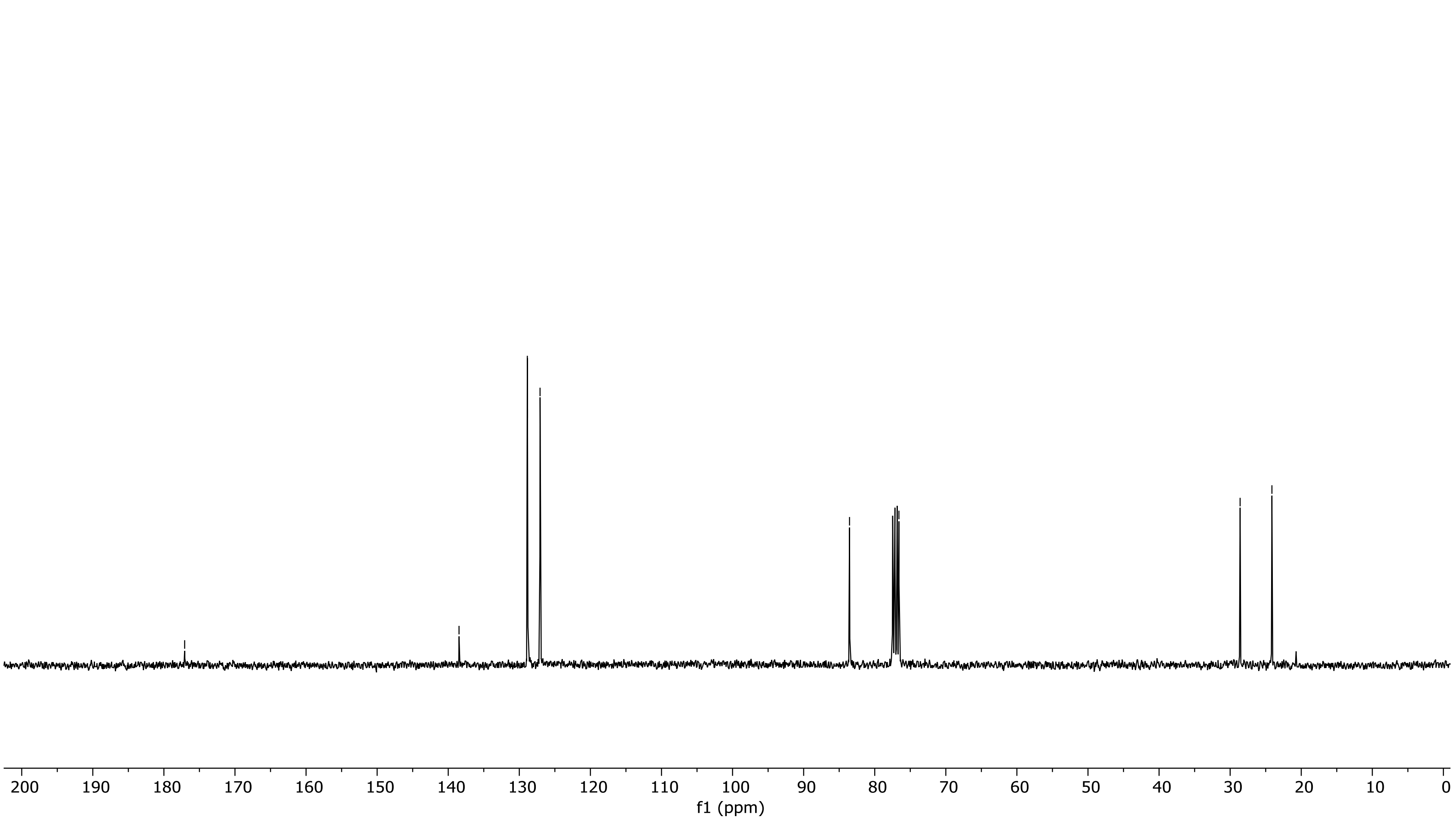

7. HPLC data analysis

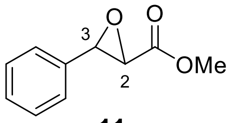

11a

Epoxidation reaction using (*S*,*S*)-Fe(**3,5-di-CF3iQ2dp**) affording methyl (*2R*,*3S*)-3-phenyloxirane-2-carboxylate **11a** in 95% *ee*

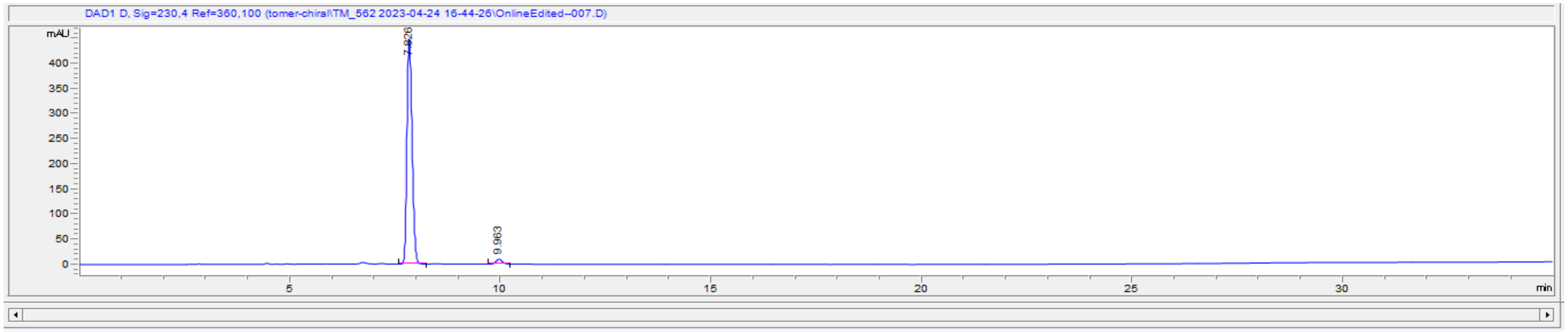

#	Time	Type	Area	Height	Width	Area%	Symmetry
1	7.826	BB	3814.5	447.4	0.1315	97.434	0.727
2	9.963	BB	100.5	9.8	0.1597	2.566	0.967

Epoxidation reaction using *ent*-Fe(**3,5-di-CF3iQ2dp**) affording methyl (*2S*,*3R*)-3-phenyloxirane-2-carboxylate ***ent*-11a** in 95% *ee*

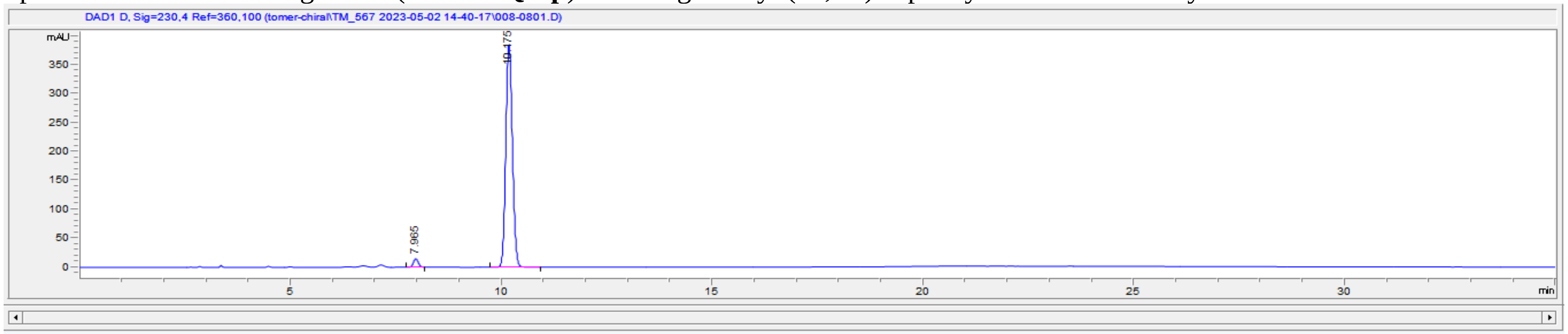

#	Time	Type	Area	Height	Width	Area%	Symmetry
1	7.965	BB	119.7	14.8	0.1267	2.748	0.954
2	10.175	BB	4236.7	388	0.1694	97.252	0.861

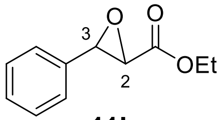

11b

Epoxidation reaction using (*S*,*S*)-Fe($^{3,5\text{-di-CF3}}$**iQ$_2$dp**) affording ethyl (2*R*,3*S*)-3-phenyloxirane-2-carboxylate **11b** in 92.5% *ee*

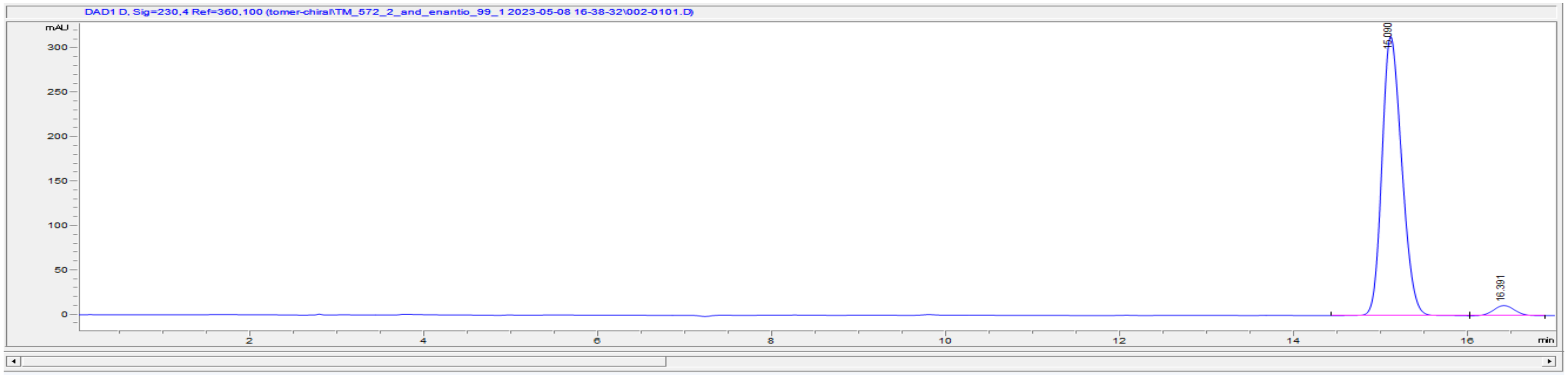

#	Time	Type	Area	Height	Width	Area%	Symmetry
1	15.09	BB	4948.3	313.3	0.2442	96.276	0.742
2	16.391	BB	191.4	11.4	0.2643	3.724	0.953

Epoxidation reaction using *ent*-Fe($^{3,5\text{-di-CF3}}$**iQ$_2$dp**) affording ethyl (2*S*,3*R*)-3-phenyloxirane-2-carboxylate ***ent*-11b** in 92.2% *ee*

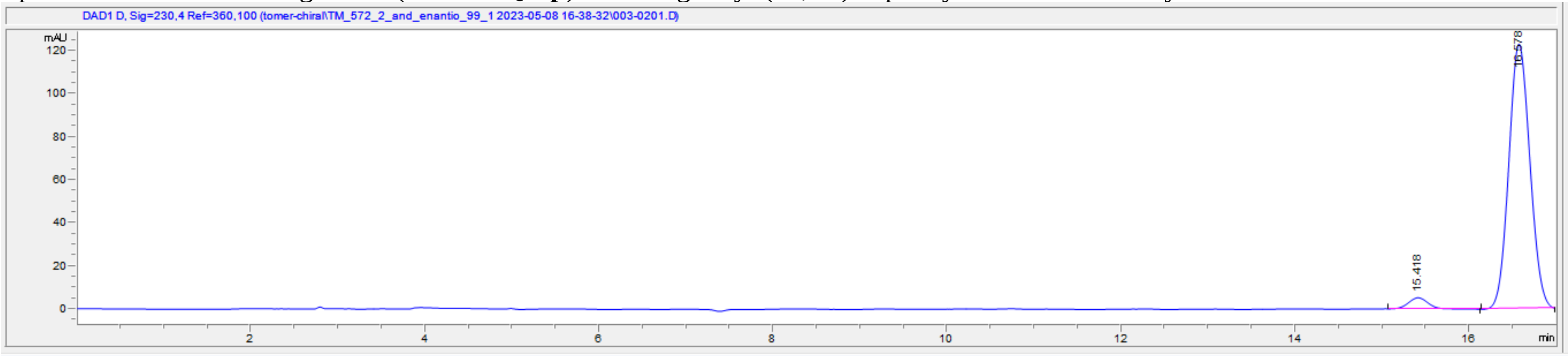

#	Time	Type	Area	Height	Width	Area%	Symmetry
1	15.418	BB	83.5	5.2	0.2453	3.883	0.861
2	16.578	BBA	2067.5	122.6	0.2647	96.117	0.894

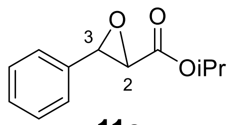

11c

Epoxidation reaction using (*S*,*S*)-Fe([3,5-di-CF3]**iQ$_2$dp**) affording isopropyl (2*R*,3*S*)-3-phenyloxirane-2-carboxylate **11c** in 86.4% *ee*

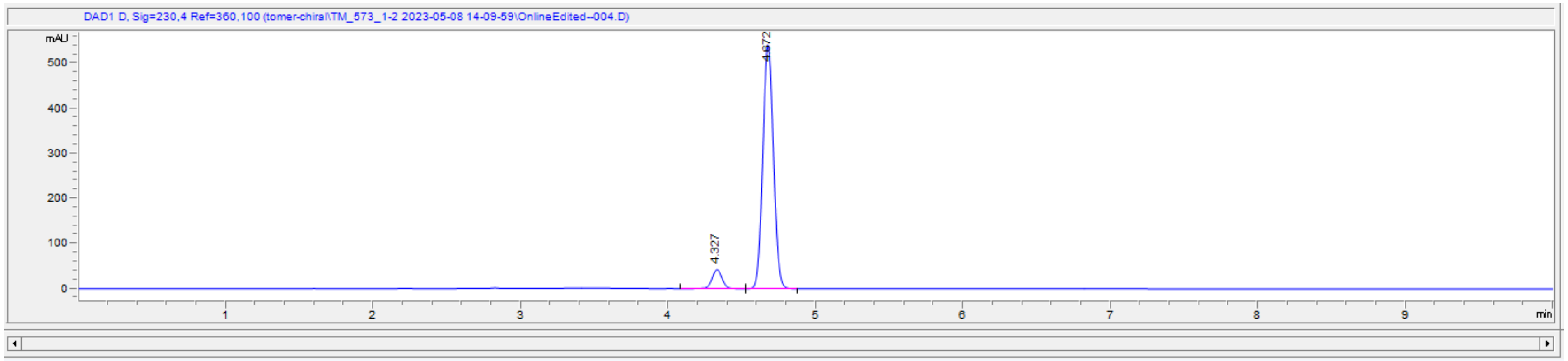

#	Time	Type	Area	Height	Width	Area%	Symmetry
1	4.327	BB	198	42.2	0.0727	6.800	0.967
2	4.672	BB	2714	543.2	0.0783	93.200	0.93

Epoxidation reaction using *ent*-Fe([3,5-di-CF3]**iQ$_2$dp**) affording isopropyl (2*S*,3*R*)-3-phenyloxirane-2-carboxylate ***ent*-11c** in 84% *ee*

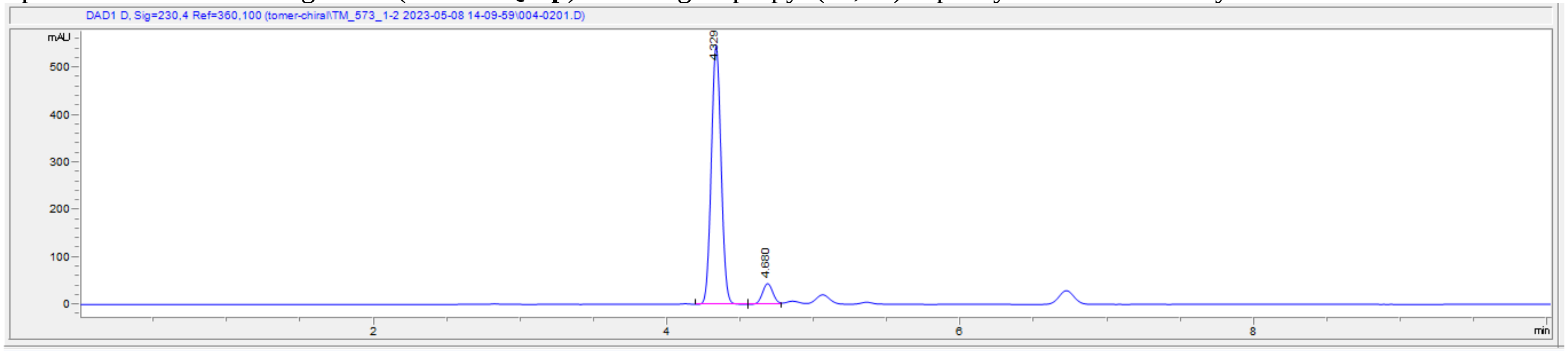

#	Time	Type	Area	Height	Width	Area%	Symmetry
1	4.329	VB	2542.9	548.5	0.0721	92.016	0.929
2	4.68	BV	220.7	44	0.0785	7.984	0.923

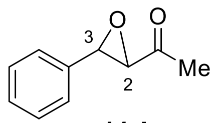

11d

Epoxidation reaction using (*S*,*S*)-Fe($^{3,5\text{-di-CF3}}$**iQ$_2$dp**) affording 1-(2*R*,3*S*)-(3-phenyloxiran-2-yl)ethan-1-one **11d** in 75% *ee.*

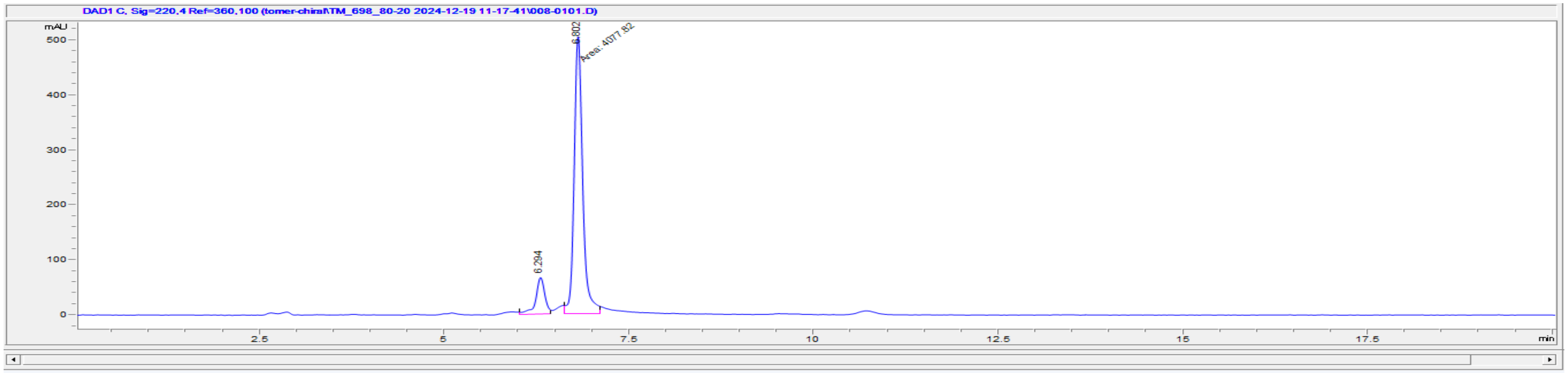

#	Time	Type	Area	Height	Width	Area%	Symmetry
1	6.294	VV	597.5	67.5	0.1313	12.781	1.114
2	6.802	MF	4077.8	506.3	0.1342	87.219	0.8

Epoxidation reaction using *ent*-Fe($^{3,5\text{-di-CF3}}$**iQ$_2$dp**) affording 1-(2*S*,3*R*)-(3-phenyloxiran-2-yl)ethan-1-one **11d** in 74% *ee.*

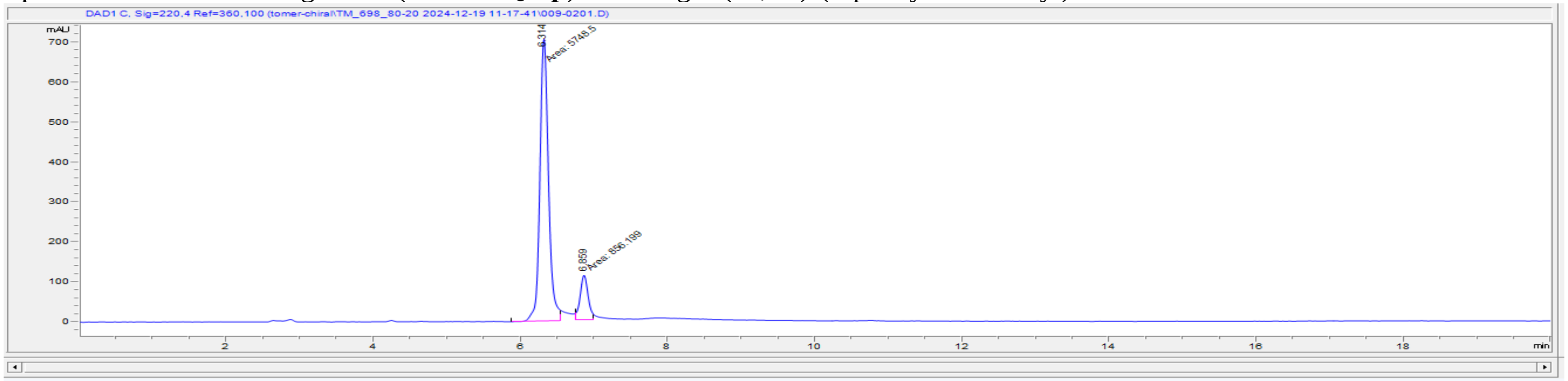

#	Time	Type	Area	Height	Width	Area%	Symmetry
1	6.314	MF	5748.5	704.5	0.136	87.037	0.847
2	6.859	FM	856.2	112.3	0.1271	12.963	0.891

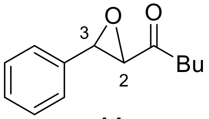

11e

Epoxidation reaction using (*S*,*S*)-Fe($^{3,5\text{-di-CF3}}$**iQ$_2$dp**) affording 1-((2*R*,3*S*)-3-phenyloxiran-2-yl)pentan-1-one **11e** in 81% *ee*.

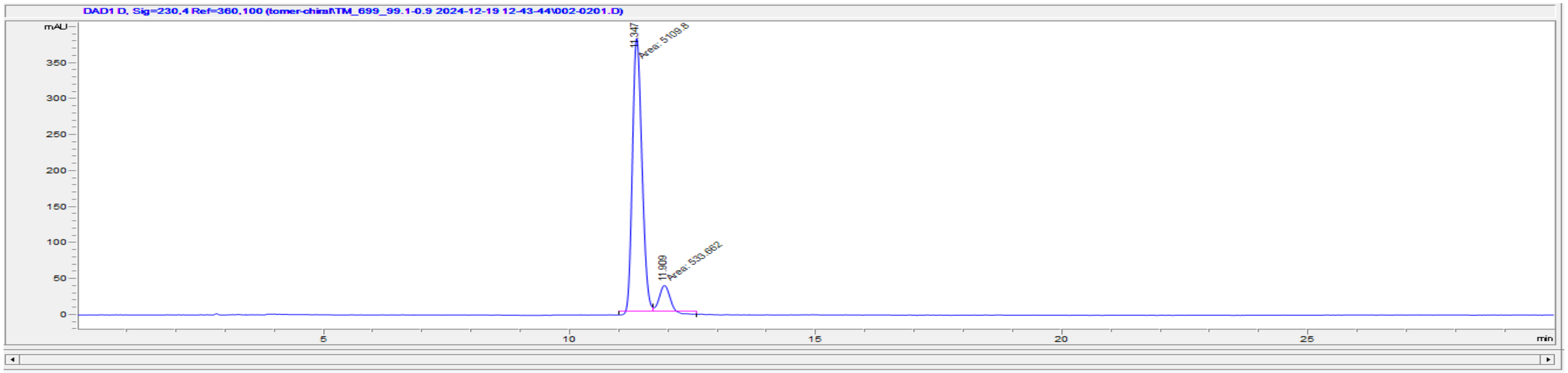

#	Time	Type	Area	Height	Width	Area%	Symmetry
1	11.347	MF	5109.8	381.2	0.2234	90.544	0.777
2	11.909	FM	533.7	36.6	0.2431	9.456	0.961

Epoxidation reaction using *ent*-Fe($^{3,5\text{-di-CF3}}$**iQ$_2$dp**) affording 1-((2*S*,3*R*)-3-phenyloxiran-2-yl)pentan-1-one **11e** in 85% *ee*.

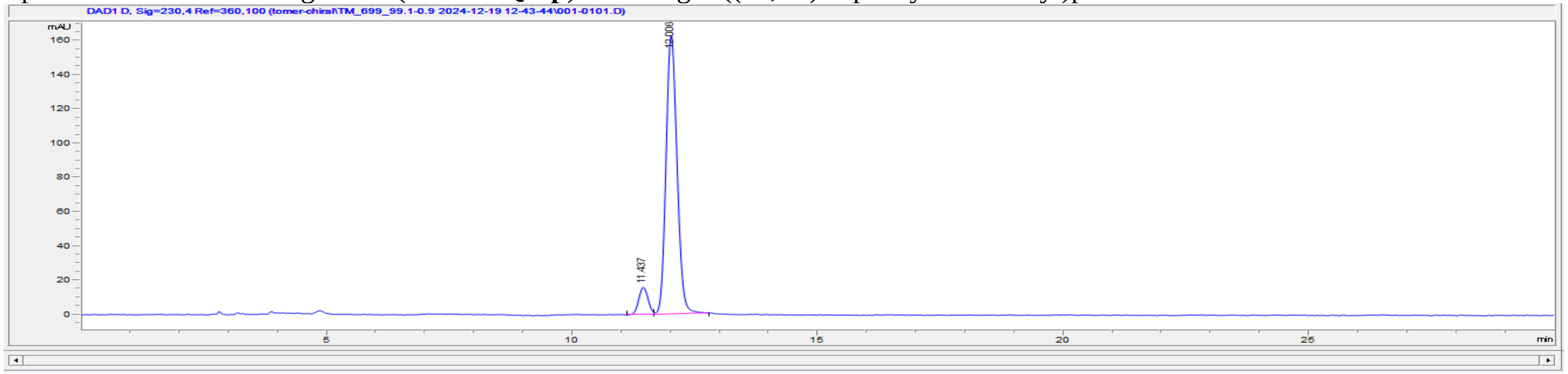

#	Time	Type	Area	Height	Width	Area%	Symmetry
1	11.437	BV	212.6	15.9	0.2159	7.745	0.915
2	12.006	VB	2532.2	162	0.2443	92.255	0.827

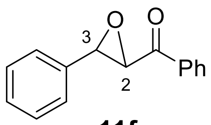

11f

Epoxidation reaction using (*S*,*S*)-Fe($^{\mathbf{3,5\text{-}di\text{-}CF3}}$**iQ$_2$dp**) affording phenyl((2*R*,3*S*)-3-phenyloxiran-2-yl)methanone **11f** in 4% *ee.*

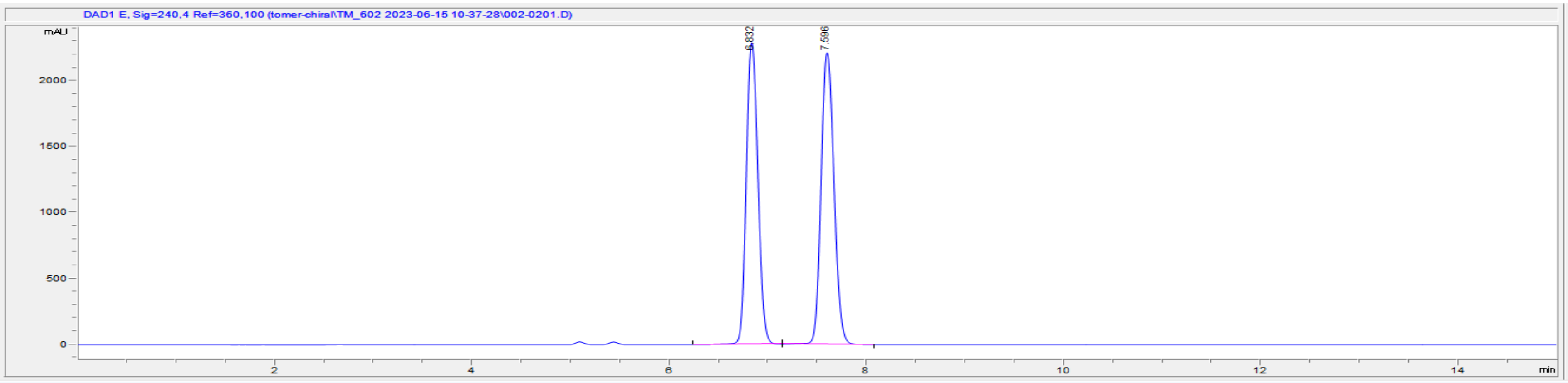

#	Time	Type	Area	Height	Width	Area%	Symmetry
1	6.832	BB	18732.2	2291.2	0.1294	48.191	0.841
2	7.596	BB	20138.3	2216.7	0.144	51.809	0.84

Epoxidation reaction using literature conditions[17] affording racemic phenyl(3-phenyloxiran-2-yl)methanone **11f.**

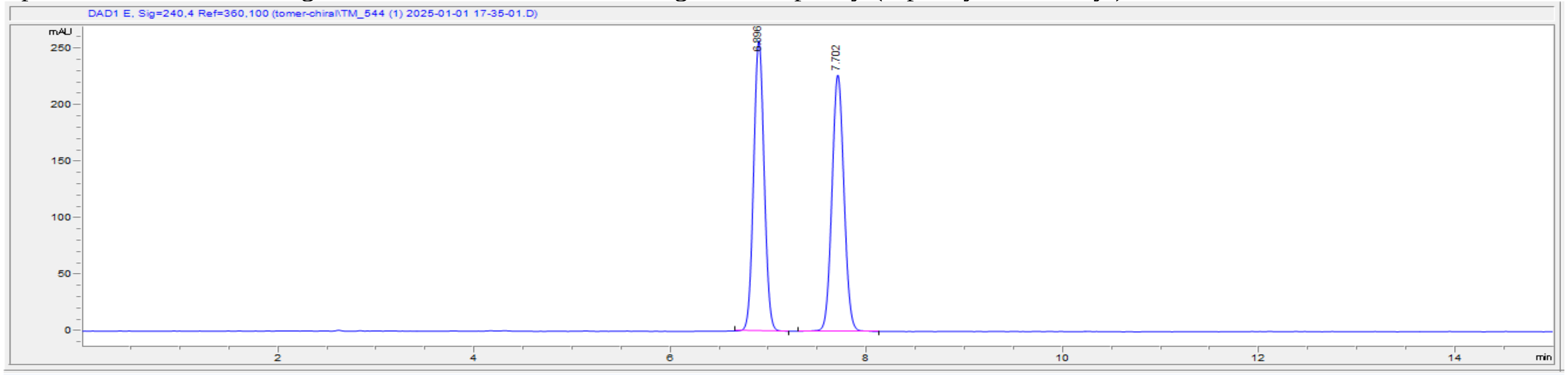

#	Time	Type	Area	Height	Width	Area%	Symmetry
1	6.896	BB	1914.9	256.6	0.1171	49.840	0.932
2	7.702	BB	1927.2	226.4	0.1313	50.160	0.93

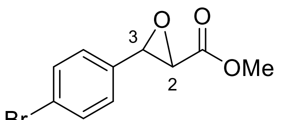

11g

Epoxidation reaction using (*S*,*S*)-Fe(**$^{3,5\text{-di-CF3}}$iQ$_2$dp**) affording methyl (2*R*,3*S*)-3-(4-bromophenyl)oxirane-2-carboxylate **11g** in 94% *ee*.

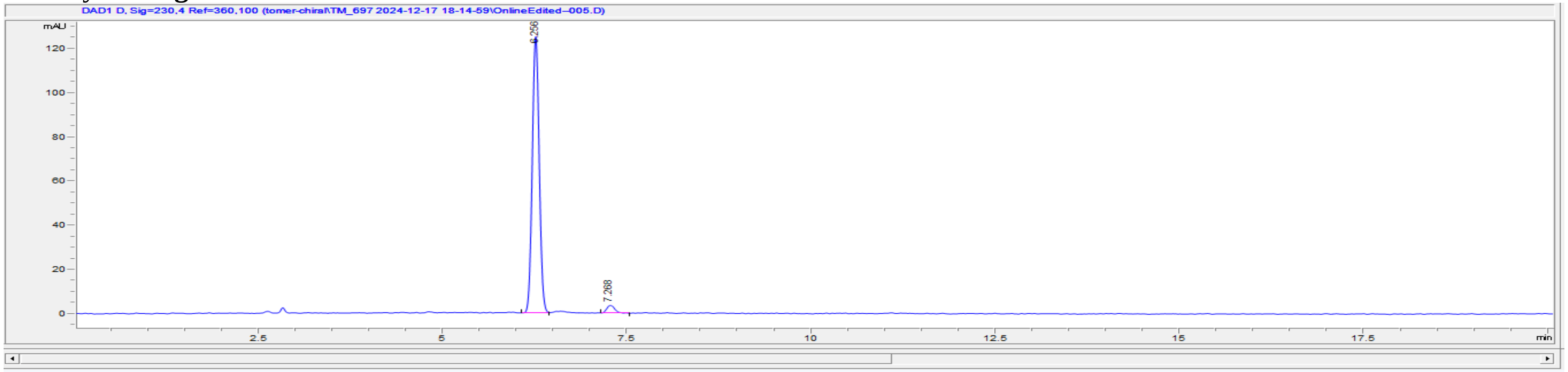

#	Time	Type	Area	Height	Width	Area%	Symmetry
1	6.256	BV	821	125.4	0.1024	96.817	0.935
2	7.268	BB	27	3.5	0.121	3.183	0.709

Epoxidation reaction using *ent*-Fe(**$^{3,5\text{-di-CF3}}$iQ$_2$dp**) affording methyl (2*S*,3*R*)-3-(4-bromophenyl)oxirane-2-carboxylate **11g** in 93% *ee*.

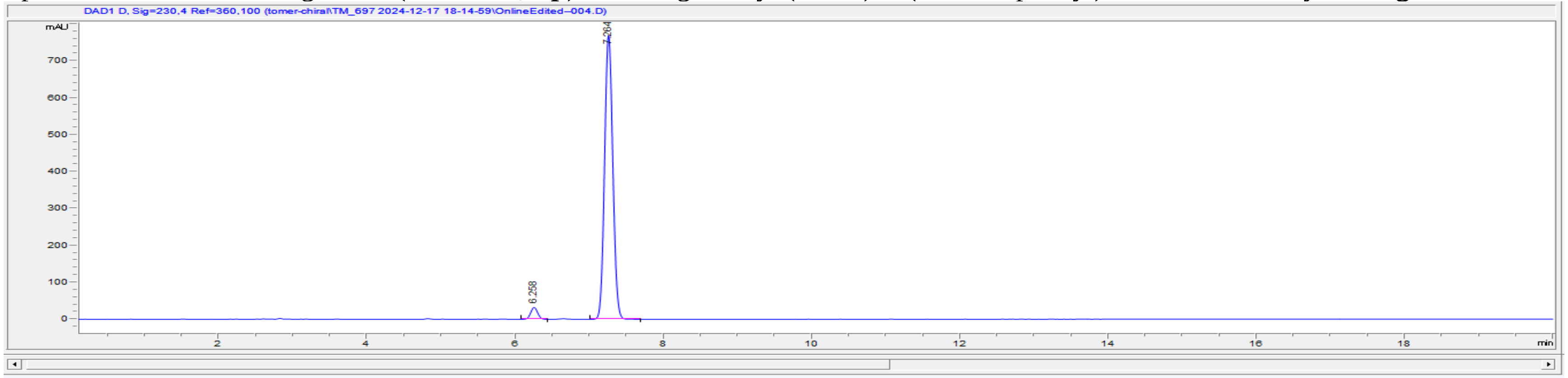

#	Time	Type	Area	Height	Width	Area%	Symmetry
1	6.258	BB	209.6	32.1	0.1001	3.411	0.945
2	7.264	BB	5935.3	767.8	0.1202	96.589	0.899

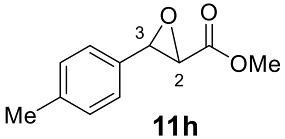

11h

Epoxidation reaction using (*S*,*S*)-Fe($^{3,5\text{-di-CF3}}$**iQ$_2$dp**) affording methyl (2*R*,3*S*)-3-(p-tolyl)oxirane-2-carboxylate **11h** in 76% *ee.*

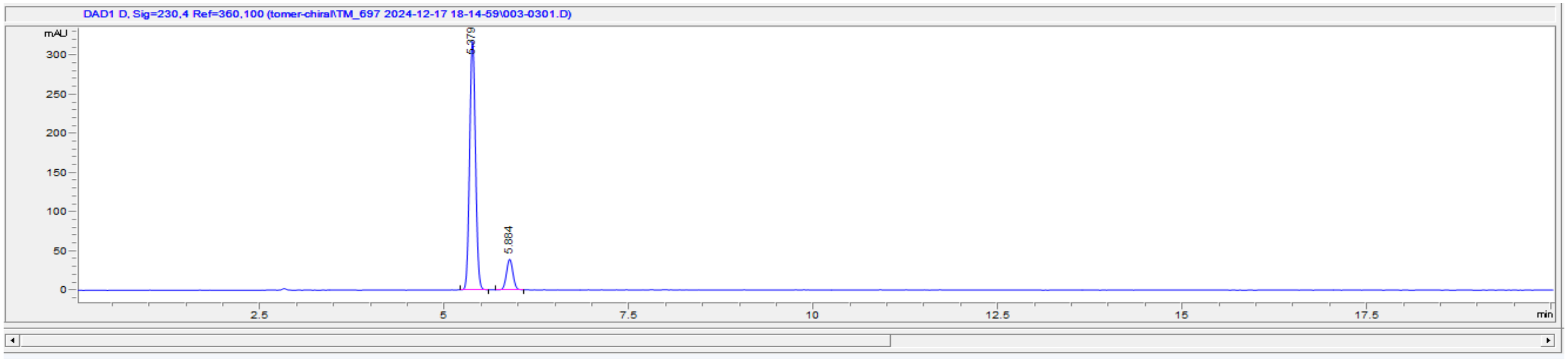

#	Time	Type	Area	Height	Width	Area%	Symmetry
1	5.379	BV	1769.8	319.2	0.0867	88.064	0.932
2	5.884	BB	239.9	39.3	0.0952	11.936	0.946

Epoxidation reaction using *ent*-Fe($^{3,5\text{-di-CF3}}$**iQ$_2$dp**) affording methyl (2*S*,3*R*)-3-(p-tolyl)oxirane-2-carboxylate **11h** in 78% *ee.*

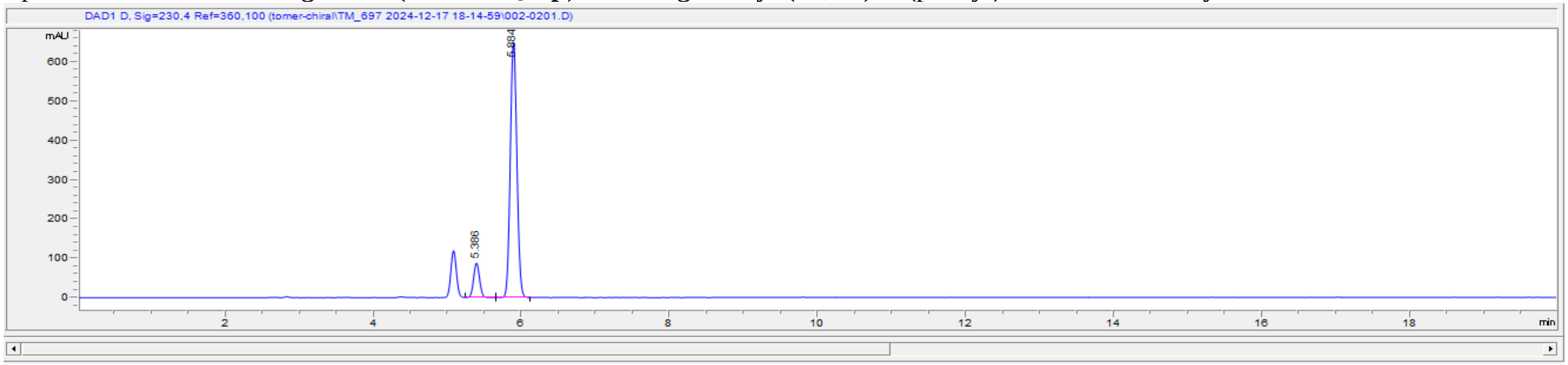

#	Time	Type	Area	Height	Width	Area%	Symmetry
1	5.386	VB	489.1	87.6	0.0871	10.885	0.933
2	5.884	BV	4004.3	648.7	0.096	89.115	0.909

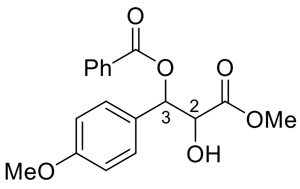

12ia

Hydroxy carboxylation reaction using (*S*,*S*)-Fe($^{3,5\text{-di-CF}_3}$**iQ$_2$dp**) affording (1*R*,2*R*)-2-hydroxy-3-methoxy-1-(4-methoxyphenyl)-3-oxopropyl benzoate **12ia** in 37.5% *ee*.

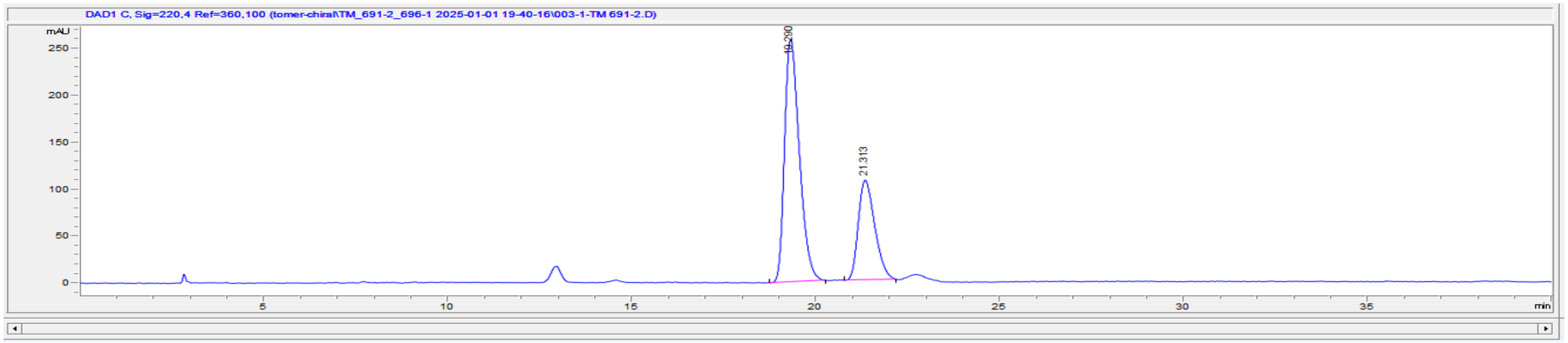

#	Time	Type	Area	Height	Width	Area%	Symmetry
1	19.29	BV	7258.1	259	0.4109	68.567	0.664
2	21.313	BV	3327.3	106.6	0.4442	31.433	0.678

Hydroxy carboxylation reaction using *ent*-Fe($^{3,5\text{-di-CF}_3}$**iQ$_2$dp**) affording (1*S*,2*S*)-2-hydroxy-3-methoxy-1-(4-methoxyphenyl)-3-oxopropyl benzoate **12ia** in 35% *ee*.

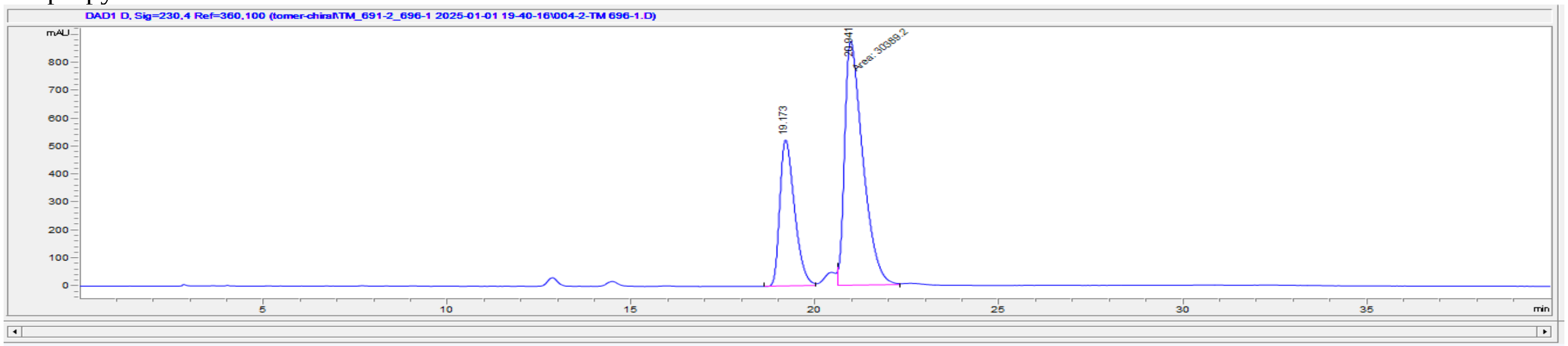

#	Time	Type	Area	Height	Width	Area%	Symmetry
1	19.173	BV	14701.3	525.6	0.4203	32.604	0.619
2	20.941	FM	30389.2	877.5	0.5772	67.396	0.472

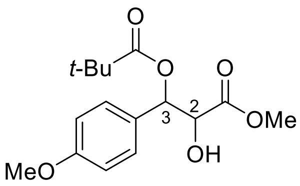

12ib

Hydroxy carboxylation reaction using (*S*,*S*)-Fe($^{3,5\text{-di-CF3}}$**iQ**$_2$**dp**) affording methyl (2*R*,3*R*)-2-hydroxy-3-(4-methoxyphenyl)-3-(pivaloyloxy)propanoate **12ib** in 82% *ee*.

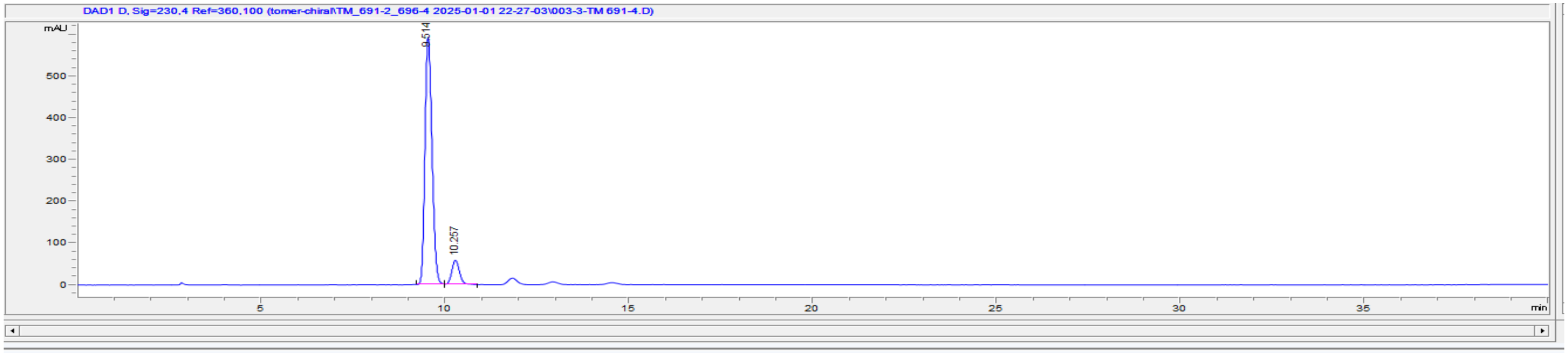

#	Time	Type	Area	Height	Width	Area%	Symmetry
1	9.514	BV	7948.6	595.6	0.2077	90.202	0.765
2	10.257	VB	863.4	58.5	0.2261	9.798	0.853

Hydroxy carboxylation reaction using *ent*-Fe($^{3,5\text{-di-CF3}}$**iQ**$_2$**dp**) affording methyl (2*S*,3*S*)-2-hydroxy-3-(4-methoxyphenyl)-3-(pivaloyloxy)propanoate **12ib** in 82% *ee*.

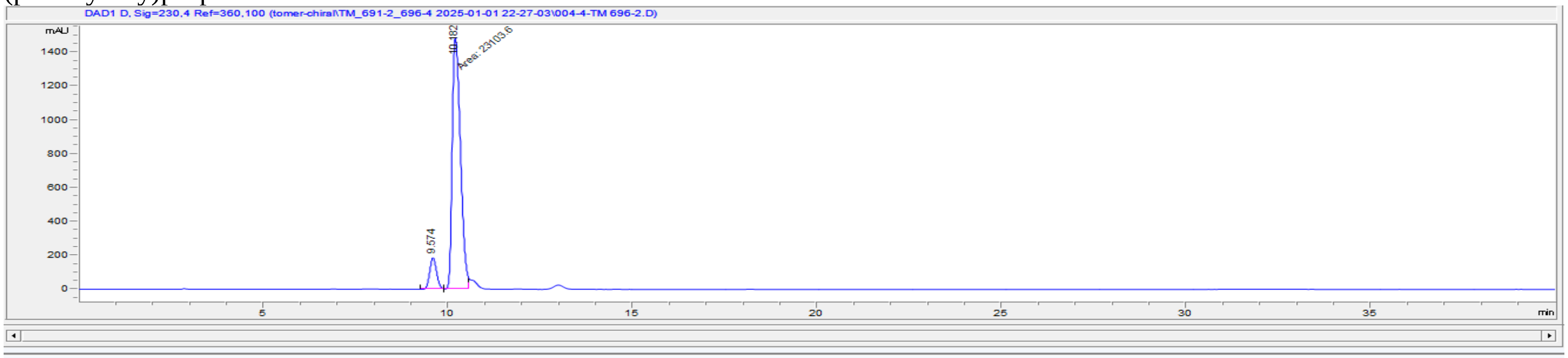

#	Time	Type	Area	Height	Width	Area%	Symmetry
1	9.574	BV	2423.1	186.2	0.2038	9.493	0.849
2	10.182	MF	23103.6	1481.3	0.26	90.507	0.593

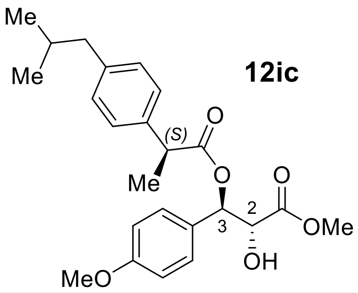

12ic

Hydroxy carboxylation reaction using (*S*,*S*)-Fe($^{\mathbf{3,5\text{-}di\text{-}CF3}}$**iQ$_2$dp**) affording methyl (2*R*,3*R*)-2-hydroxy-3-(((S)-2-(4-isobutylphenyl)propanoyl)oxy)-3-(4-methoxyphenyl)propanoate **12ic** in 81% *de*.

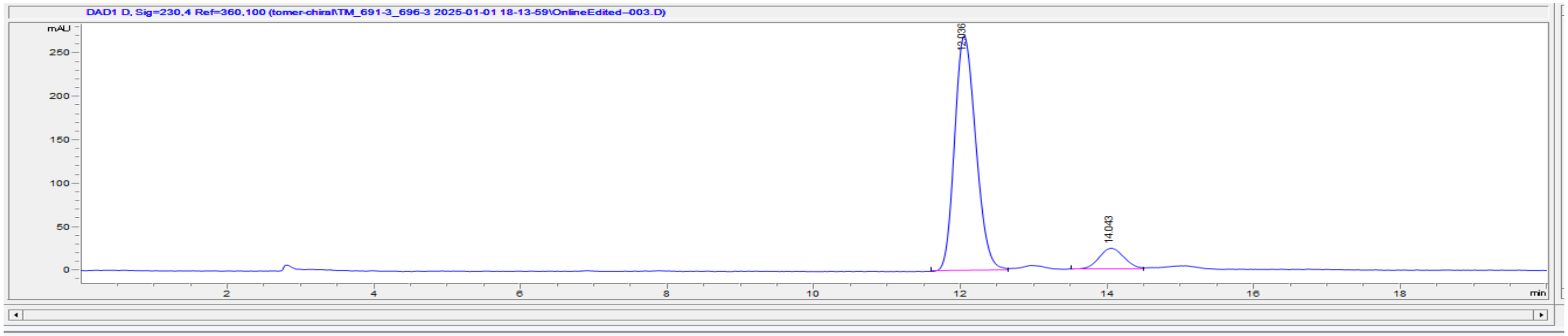

#	Time	Type	Area	Height	Width	Area%	Symmetry
1	12.036	BV	5447.8	270.5	0.315	90.208	0.771
2	14.043	VV	591.4	24.3	0.3338	9.792	0.927

Hydroxy carboxylation reaction using *ent*-Fe($^{\mathbf{3,5\text{-}di\text{-}CF3}}$**iQ$_2$dp**) affording methyl (2*S*,3*S*)-2-hydroxy-3-(((S)-2-(4-isobutylphenyl)propanoyl)oxy)-3-(4-methoxyphenyl)propanoate **12ic** in 63% *de*.

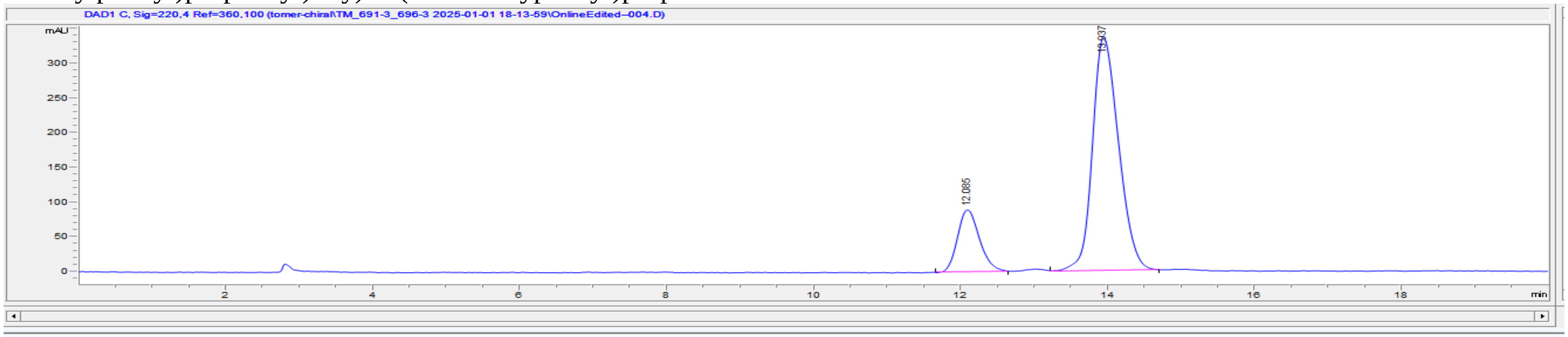

#	Time	Type	Area	Height	Width	Area%	Symmetry
1	12.085	BV	1883	89.6	0.323	18.532	0.778
2	13.937	VB	8277.9	334.9	0.3784	81.468	0.727

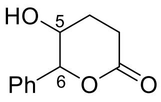

12j

Lactonization reaction using (*S*,*S*)-**Fe(**[**3,4,5-(tri-F)**]**iQ**$_{2}$**dp)** affording (5*R*,6*S*)-5-hydroxy-6-phenyltetrahydro-2H-pyran-2-one **12j** in 50% *ee.*

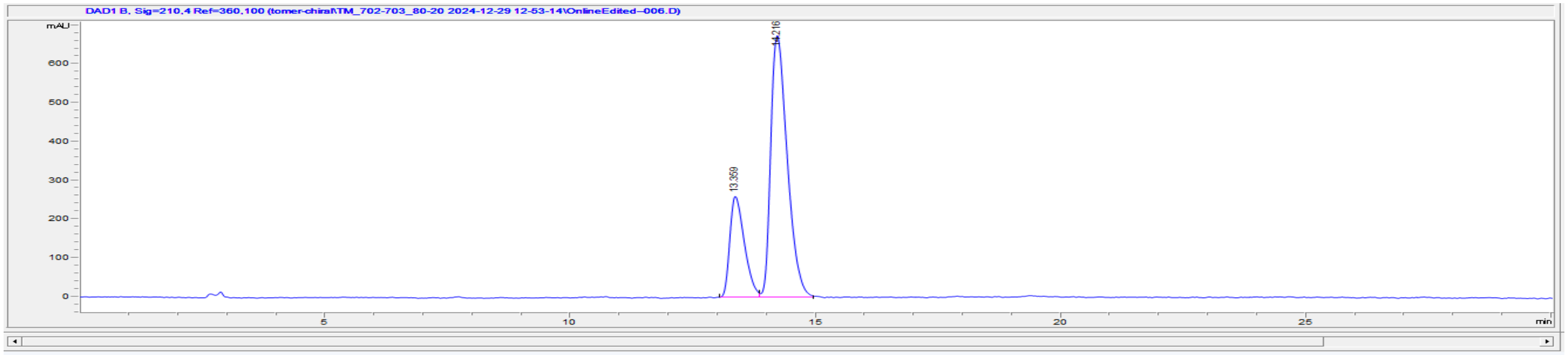

#	Time	Type	Area	Height	Width	Area%	Symmetry
1	13.359	BV	5427.6	261.1	0.3105	25.437	0.576
2	14.216	VV	15910.2	677.4	0.346	74.563	0.619

Lactonization reaction using *ent*-Fe([**3,5-di-CF3**]**iQ**$_{2}$**dp**) affording (5*S*,6*R*)-5-hydroxy-6-phenyltetrahydro-2H-pyran-2-one **12j** in 43% *ee.*

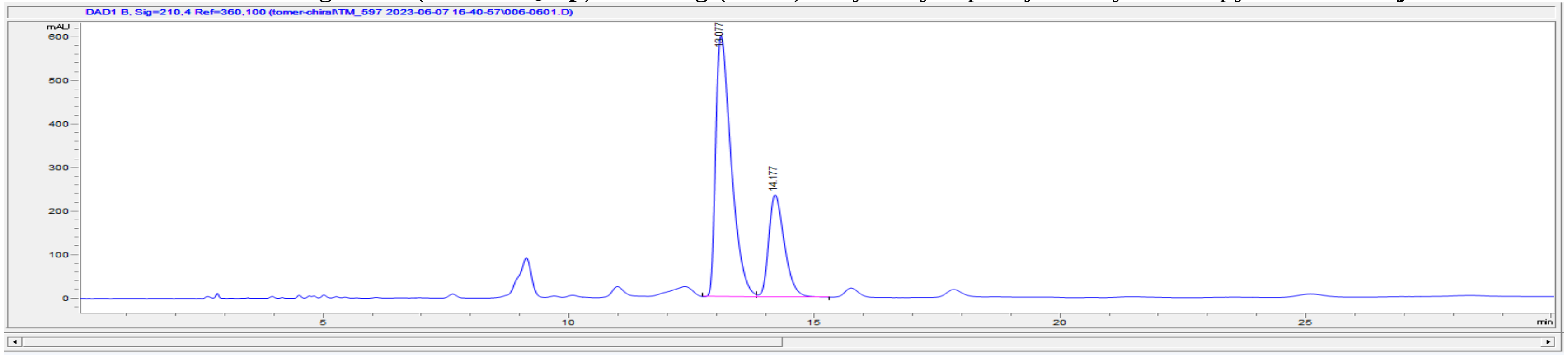

#	Time	Type	Area	Height	Width	Area%	Symmetry
1	13.077	BV	13125.6	598.6	0.3318	71.620	0.482
2	14.177	VB	5201.2	234.1	0.3432	28.380	0.645

8. References

(1) Goddard, T. D.; Huang, C. C.; Meng, E. C.; Pettersen, E. F.; Couch, G. S.; Morris, J. H.; Ferrin, T. E. UCSF ChimeraX: Meeting modern challenges in visualization and analysis. *Protein Sci.* **2018**, *27* (1), 14-25. DOI: 10.1002/pro.3235 From NLM.
(2) Schaefer, A. J.; Ingman, V. M.; Wheeler, S. E. SEQCROW: A ChimeraX bundle to facilitate quantum chemical applications to complex molecular systems. *J. Comput. Chem.* **2021**, *42* (24), 1750-1754. DOI: https://doi.org/10.1002/jcc.26700. Ingman, V. M.; Schaefer, A. J.; Andreola, L. R.; Wheeler, S. E. QChASM: Quantum chemistry automation and structure manipulation. *Wiley Interdiscip. Rev. Comput. Mol. Sci.* **2021**, *11* (4), e1510. DOI: https://doi.org/10.1002/wcms.1510.
(3) Gormisky, P. E.; White, M. C. Catalyst-Controlled Aliphatic C–H Oxidations with a Predictive Model for Site-Selectivity. *J. Am. Chem. Soc.* **2013**, *135* (38), 14052-14055. DOI: 10.1021/ja407388y.
(4) Grant, T. N.; West, F. G. A New Approach to the Nazarov Reaction via Sequential Electrocyclic Ring Opening and Ring Closure. *J. Am. Chem. Soc.* **2006**, *128* (29), 9348-9349. DOI: 10.1021/ja063421a.
(5) Sirinimal, H. S.; Hebert, S. P.; Samala, G.; Chen, H.; Rosenhauer, G. J.; Schlegel, H. B.; Stockdill, J. L. Synthetic and Computational Study of Tin-Free Reductive Tandem Cyclizations of Neutral Aminyl Radicals. *Org. Lett.* **2018**, *20* (20), 6340-6344. DOI: 10.1021/acs.orglett.8b02456.
(6) Cussó, O.; Garcia-Bosch, I.; Ribas, X.; Lloret-Fillol, J.; Costas, M. Asymmetric Epoxidation with H2O2 by Manipulating the Electronic Properties of Non-heme Iron Catalysts. *J. Am. Chem. Soc.* **2013**, *135* (39), 14871-14878. DOI: 10.1021/ja4078446.
(7) Murafuji, T.; Hafizur Rahman, A. F. M.; Magarifuchi, D.; Narita, M.; Miyakawa, I.; Ishiguro, K.; Kamijo, S. One-Pot Synthesis of Hypervalent Diaryl(iodo)bismuthanes from<i> o</i>-Carbonyl Iodoarenes by Zincation. *Heteroat. Chem.* **2019**, *2019*, 2385064. DOI: 10.1155/2019/2385064.
(8) Roesch, K. R.; Larock, R. C. Synthesis of isoquinolines and pyridines by the palladium-and copper-catalyzed coupling and cyclization of terminal acetylenes. *Org. Lett.* **1999**, *1* (4), 553-556.
(9) Cianfanelli, M.; Olivo, G.; Milan, M.; Klein Gebbink, R. J. M.; Ribas, X.; Bietti, M.; Costas, M. Enantioselective C–H Lactonization of Unactivated Methylenes Directed by Carboxylic Acids. *J. Am. Chem. Soc.* **2020**, *142* (3), 1584-1593. DOI: 10.1021/jacs.9b12239.
(10) Hagen, K. S. Iron(II) Triflate Salts as Convenient Substitutes for Perchlorate Salts: Crystal Structures of [Fe(H2O)6](CF3SO3)2 and Fe(MeCN)4(CF3SO3)2. *Inorg. Chem.* **2000**, *39* (25), 5867-5869. DOI: 10.1021/ic000444w.
(11) Moyna, G.; Williams, H. J.; Scott, A. I. An Improved Procedure for the Epoxidation of Methyl Cinnamate Derivatives and Production of Acid Sensitive Epoxides. *Synth. Commun.* **1996**, *26* (11), 2235-2239. DOI: 10.1080/00397919608003584.
(12) Xiang, M.; Ni, X.; Yi, X.; Zheng, A.; Wang, W.; He, M.; Xiong, J.; Liu, T.; Ma, Y.; Zhu, P.; et al. Preparation of Mesoporous Zeolite ETS-10 Catalysts for High-Yield Synthesis of α,β-Epoxy Ketones. *ChemCatChem* **2015**, *7* (3), 521-525. DOI: https://doi.org/10.1002/cctc.201402839.
(13) Mamedov, V. A.; Mamedova, V. L.; Syakaev, V. V.; Voronina, J. K.; Mahrous, E. M.; Korshin, D. E.; Latypov, S. K.; Sinyashin, O. G. Regioselective syntheses of 3-hydroxy-4-aryl-3, 4, 5-trihydro-2H-benzo [b][1, 4] diazepin-2 (1H)-ones and 3-benzylquinoxalin-2 (1H)-ones from arylglycidates when exposed to 1, 2-diaminobenzenes. *Tetrahedron* **2020**, *76* (41), 131478.
(14) Imashiro, R.; Seki, M. A Catalytic Asymmetric Synthesis of Chiral Glycidic Acid Derivatives through Chiral Dioxirane-Mediated Catalytic Asymmetric Epoxidation of Cinnamic Acid Derivatives. *J. Org. Chem.* **2004**, *69* (12), 4216-4226. DOI: 10.1021/jo049893u.

(15) Dolomanov, O. V.; Bourhis, L. J.; Gildea, R. J.; Howard, J. A. K.; Puschmann, H. OLEX2: A complete structure solution, refinement and analysis program *J. Appl. Cryst.* **2009**, *42* (2), 339-341.
(16) Sheldrick, G. M. Crystal structure refinement with SHELXL. *Acta Crystallographica Section C Structural Chemistry* **2015**, *71* (1), 3-8.
(17) dos Santos, A. A.; Wendler, E. P.; Marques, F. d. A.; Simonelli, F. Microwave-accelerated epoxidation of α, β-unsaturated ketones with urea-hydrogen peroxide. *Letters in Organic Chemistry* **2004**, *1* (1), 47-49.
